# Taxa and names in *Cynoglossum* sensu lato (*Boraginaceae*, *Cynoglosseae*): an annotated, synonymic inventory, with links to the protologues and mention of original material

**DOI:** 10.3897/BDJ.3.e4831

**Published:** 2015-04-22

**Authors:** Hartmut H. Hilger, Werner Greuter, Victoria Stier

**Affiliations:** ‡ Freie Universität Berlin - Biology, Berlin, Germany; § Botanischer Garten & Botanisches Museum Berlin-Dahlem, Freie Universität Berlin, Berlin, Germany

**Keywords:** *
Boraginales
*, *
Boraginaceae
*, *
Cynoglosseae
*, *Cynoglossum* sensu lato, taxonomy, nomenclature

## Abstract

**Background:**

An inventory is presented of all names so far validly published in ***Cynoglossum*** sensu lato and its segregate genera: ***Adelocaryum*, *Afrotysonia*, *Kuschakewiczia*, *Lindelofia*, *Mattiastrum*, *Paracaryum*, *Rindera*, *Solenanthus*, *Trachelanthus*,** and their synonyms. Names and designations that were not validly published in the cited place, and later isonyms, are accounted for when they have been included in the International Plant Name Index (IPNI). Problems with IPNI entries, including errors and omissions, are discussed, and the hope is expressed that the present inventory may be of use for fixing them.

**New information:**

The inventory, generated from a list of structured data, is presented in two Supplements, as a searchable HTML document comprising a sequence of entries with internal cross-links and links to external sources, in particular to protologues accessible online or, copyright restrictions permitting, made available as scanned documents via DOIs, and as machine-readible file. With minor exceptions, all names have been verified in their original place of publication, and all were nomenclaturally assessed. Colour coding is used to distinguish between names (in **green**) pertaining to ***Cynoglossum*** sensu lato, for which complete synonymies are provided; and names (in **orange**) pertaining to other genera but published under ***Cynoglossum*** or its segregates. They are listed together with their basionym and the corresponding correct name (if it exists), but without complete synonymy. Acceptable, potentially correct names appear in bold-face type, both under a broadly defined ***Cynoglossum*** (for which purpose validation of 81 new combinations and the name of 1 new species was necessary) and under one or more of its segregates. When a name was published for a new taxon, original material is indicated, usually by direct quotation from the protologue. New type designations are exceptional (two cases), whereas former type designations are cited whenever known. Furthermore, types and original specimens, especially when their digital images are available online, are mentioned with their locations and accession numbers. Comments are added whenever appropriate, especially to explain nomenclatural assessments that are not self-evident.

## Introduction

The cosmopolitan genus ***Cynoglossum*** (*Boraginaceae* sensu stricto) consists of more or less short-lived, predominantly biennial herbs. Anthers and stigma are usually included in the corolla tube, which at the throat is closed by hollow scales or folds called fornices. The fruit separates at maturity into more or less roundish, mostly glochid-bearing dry mericarpids (eremocarps, “nutlets”; German: Klausen).

Delimitation of the genus ***Cynoglossum*** (and thus of the main part of the *Cynoglosseae*) is controversial. Based on an unresolved relationship of taxa traditionally defined by morphological traits, mainly of the fruit, authors such as Greuter & Burdet (in Greuter 1981) plead for a very wide circumscription (with ***Cynoglossum*** sensu lato forming a single, large genus); at the other extreme, authors such as Riedl (1967) and many before and after him, subdivided that same assemblage into up to 10 genera, a position that is still favoured by one of the present authors (HHH). Popov (1953), among others, follows an intermediate path, e.g., concerning the possible merger of ***Mattiastrum*** and ***Paracaryum***.

In recent years, DNA sequencing studies (Selvi et al. 2011, Weigend et al. 2013) have tended at least in part to support the position of Greuter & Burdet (in Greuter 1981). In phylogenetic trees resulting from such studies, ***Cynoglossum*** sensu stricto, as traditionally defined (mainly by fruit characters), is clearly polyphyletic. Even when some discordant (New-World, African and South Asian) elements currently assigned to ***Cynoglossum*** are removed, ***Cynoglossum*** sensu stricto remains paraphyletic, as morphologically deviating genera such as ***Paracaryum*, *Paracynoglossum*, *Pardoglossum*** and ***Solenanthus*** are nested within it. This means that the characters traditionally used for delimiting genera are insufficient to define monophyletic groups. Only if and when additional, phylogenetically meaningful features are found will it be possible to define natural units, potentially of generic rank, within ***Cynoglossum*** sensu lato. This has not happened as yet but is an important task for the future, combined with DNA sequence analyses of those species (more than one half) that have not so far been investigated.

To facilitate future research, it appeared to be a worth-while task to establish a complete inventory of taxa so far described and validly named, at any rank, within ***Cynoglossum*** sensu lato. We decided to include into that inventory any and all combinations published under either ***Cynoglossum*** itself or the name of a genus pertaining to ***Cynoglossum*** sensu lato, with their respective basionyms, even when – on the basis of current phylogenetic understanding – we do not consider that the corresponding taxon belongs to the latter. In order to assess the nomenclatural status of every name, we had to verify the original publication of each, i.e. its protologue. Therefore,it appeared logical to include into our inventory the protologue information relating to the respective original material. As a corollary, we endeavoured to find and put on record as many subsequent type designations as possible. Needless to say, the tracing and incorporation of this additional information increased our self-set task manifold – but we believe that the added value conferred to the inventory justifies our effort.

Our initial approach was to establish a purely nomenclatural inventory, with cross-links between entries of homotypic names. However, we established two levels with different coverage. For all taxa falling within the unit that we call ***Cynoglossum*** sensu lato (and also for the members of *Afrotysonia*, which, while phylogenetically not close to *Cynoglossum*, are all but distinguishable from it morphologically) we give a complete synonymy. For other, non-***Cynoglossum*** taxa we only list names published under genera that pertain to ***Cynoglossum*** sensu lato, plus their basionym, if any. This distinction forced us to assess the appropriate placement of the named taxa, whether within or outside of ***Cynoglossum*** sensu lato, which is a taxonomic decision – and, as it turned out, not always a trivial one. Once we realised that we had lost our taxonomically virgin status we decided that we might just as well abandon our self-imposed restraint and introduce taxonomic assessment throughout, appreciating that users are keen to be given that kind of guidance. Since the present authors disagree as to the appropriate generic classification, we are in many cases, for the same taxon, listing more than one name as being potentially correct, i.e., as expressing possible generic placements.

The International Plant Name Index (IPNI: http://www.ipni.org) provided an ideal starting point and first basis for our inventory. Our task then consisted in a literature search for additional names, verification of the source of each and every entry found in IPNI, and nomenclatural (plus ultimately taxonomic) assessment of all names. Especially with respect to taxonomic assessment we made frequent use of the excellent facilities now provided by the Catalogue of Life dynamic checklist (CoL: http://www.catalogueoflife.org/col). By the way of feedback, our inventory will hopefully be used to improve the completeness and accuracy of the data in both IPNI and CoL.

## Geographic coverage

### Description

Worldwide

## Taxonomic coverage

### Description

*Cynoglossum* sensu lato, as defined for the purpose of the present inventory, relies heavily on the results of DNA sequencing studies, both published Selvi et al. 2011, Weigend et al. 2013, Selvi et al. 2011) and ongoing (Weigend, pers. comm.). In addition to *Cynoglossum* sensu lato as it appears in Weigend et al. 2013 (fig. 4), their PAR I (*Paracaryum* I) clade is here included.

Conversely, the genera *Microparacaryum* and *Bothriospermum*, even though close to *Cynoglossum* sensu lato according to DNA-based phylogeny, are considered sufficiently distinct to be left outside. The same applies to the not yet sequenced *Brandella*, a likely synonym of *Microparacaryum*.

Finally, there are two groups of species, or clades, that have not so far been challenged as members of *Cynoglossum* but, according not only to sequence data, but at least to some extent morphology, do not belong here. One comprises the indigenous New-World *“Cynoglossum”* species, the second a group of mainly African species, some of which had been erroneously placed in *Paracynoglossum*. These groups are here considered as non-*Cynoglossum* and must await their description as separate genera to be correctly placed. A similar case is that of *Afrotysonia*, a small African genus morphologically very close to *Cynoglossum*, but which on account of recent, unpublished sequence data (Weigend, pers. comm.) is akin to the afore-mentioned African group.

To make the above taxonomic distinction immediately clear for the reader, in the inventory the headline with a name of a taxon belonging to *Cynoglossum* sensu lato appears in green type, and that for a non- *Cynoglossum* taxon, in orange type. Furthermore a taxonomic distinction is made: for names that are potentially acceptable under either *Cynoglossum* in the wide sense or one of its constituent segregate genera the headline is set in **bold-face type**; for those of non- *Cynoglossum* taxa that are currently accepted it also appears in **bold-face print**; for all other names (whether legitimate or illegitimate) that we consider as synonyms under any currently acceptable option the headline is left in normal type.

The list of names of genera belonging in *Cynoglossum* sensu lato, at least with respect to their nomenclatural type (mentioned in parenthesis) is as follows (potentially correct generic names appear in ***bold-face italics***, currently accepted names, when heterotypic, are placed in square brackets):

***Adelocaryum*** Brand (*A. coelestinum* (Lindl.) Brand, *Cynoglossum coelestinum* Lindl.)

*Anchusopsis* Bisch. (*A. longiflora* (DC.) Bisch., *Cynoglossum longiflorum* Benth. (non Lehm.), *Omphalodes longiflora* DC. [= *C. grandiflorum* Benth.] (≡ ***Lindelofia*** Lehm.)

*Bilegnum* Brand (*B. bungei* (Boiss.) Brand, *Mattia bungei* Boiss., *Cynoglossum bungei* (Boiss.) Greuter & Stier)

*Cerinthopsis* Kotschy ex Paine (*C. foliosa* Paine, *Cynoglossum foliosum* (Paine) Greuter & Burdet)

***Cynoglossum*** L. (*C. officinale* L.)

*Cyphomattia* Boiss. (*C. lanata* (Lam.) Boiss., *Cynoglossum lanatum* Lam.)

***Kuschakewiczia*** Regel & Smirn. (*K. turkestanica* Regel & Smirn., *Cynoglossum turkestanicum* (Regel) Greuter & Stier)

***Lindelofia*** Lehm. (*L. spectabilis* Lehm., nom. illeg., *Cynoglossum longiflorum* Benth. (non Lehm.), *Omphalodes longiflora* DC., *L. longiflora* (DC.) Baill.) [= *C. grandiflorum* Benth.]

*Mattia* Schult., nom. illeg. (≡ ***Rindera*** Pall.) (*R. tetraspis* Pall., *Cynoglossum tetraspis* (Pall.) Greuter & Burdet)

*Mattia* Roem. & Schult., nom. illeg. (non *Mattia* Schult.) (*Paracaryum aucheri* (A. DC.) Boiss., *M. aucheri* A. DC., *Mattiastrum aucheri* (A. DC.) Brand, *Cynoglossum aucheri* (A. DC.) Greuter & Burdet)

***Mattiastrum*** (Boiss.) Brand (Paracaryum sect. Mattiastrum Boiss.) (type not designated)

*Paracaryopsis* (Riedl) R. R. Mill (Cynoglossum sect. Paracaryopsis Riedl, ***Adelocaryum*** Brand) (*C. coelestinum* Lindl., *P. coelestina* (Lindl.) R. R. Mill, *A. coelestinum* (Lindl.) Brand)

***Paracaryum*** (A. DC.) Boiss. (Omphalodes sect. Paracaryum A. DC.) (*O. rugulosa* DC., *P. rugulosum* (DC.) Boiss., *Cynoglossum rugulosum* (DC.) Greuter & Burdet)

*Paracynoglossum* Popov (*P. denticulatum* (A. DC.) Popov, *Cynoglossum denticulatum* A. DC.)

*Pardoglossum* Barbier & Mathez (*P. atlanticum* (Pit.) Barbier & Mathez, *Solenanthus atlanticus* Pit., *Cynoglossum pitardianum* Greuter & Burdet)

***Rindera*** Pall. (*R. tetraspis* Pall., *Cynoglossum tetraspis* (Pall.) Greuter & Burdet)

***Solenanthus*** Ledeb. (*S. circinnatus* Ledeb., *Cynoglossum circinnatum* (Ledeb.) Greuter & Burdet)

***Trachelanthus*** Kunze (*T. cerinthoides* (Boiss.) Kunze, *Solenanthus cerinthoides* Boiss., *Cynoglossum cerinthoides* (Boiss.) Greuter & Burdet

None of the following genera, with respect to their nomenclatural type (mentioned in parenthesis), is assigned by us to *Cynoglossum* sensu lato. However, each of them (except *Afrotysonia*) once included taxa that are currently placed, or still includes taxa that were at one time placed, in a genus belonging to *Cynoglossum* sensu lato (potentially correct generic names appear in ***bold-face italics***, currently accepted names, when heterotypic, are placed in square brackets):

***Afrotysonia*** Rauschert (*Tysonia* Bolus, nom. illeg., non *Tysonia* Fontaine) (*A. africana* (Bolus) Rauschert, *Tysonia africana* Bolus)

***Anchusa*** L. (*A. officinalis* L.)

***Antiotrema*** Hand.-Mazz. (*A. dunnianum* (Diels) Hand.-Mazz., *Cynoglossum dunnianum* Diels)

***Austrocynoglossum*** Popov ex R. R. Mill (*A. latifolium* (R. Br.) R. R. Mill, *Cynoglossum latifolium* R. Br.)

***Bothriospermum*** Bunge (*B. chinense* Bunge)

*Brandella* R. R. Mill (*B. erythraea* (Brand) R. R. Mill, *Adelocaryum erythraeum* Brand, *Cynoglossum erythraeum* (Brand) Riedl) [= ***Microparacaryum***]

***Cynoglossopsis*** Brand (*C. latifolia* (Hochst. ex A. Rich.) Brand, *Echinospermum latifolium* Hochst. ex A. Rich., *Cynoglossum hochstetteri* Vatke)

*Cynoglossospermum* Siegesb. ex Kuntze, nom. illeg. (≡ ***Lappula*** Moench) (*C. lappula* (L.) Kuntze, *Myosotis lappula* L.) [= *L. squarrosa* (Retz.) Dumort.]

*Echinospermum* Lehm., nom. illeg. (≡ ***Lappula*** Moench) (*E. lappula* (L.) Lehm., *Myosotis lappula* L.) [= *L. squarrosa* (Retz.) Dumort.]

***Echium*** L. (*E. vulgare* L.)

***Eritrichium*** Schrad. ex Gaudin (*E. nanum* (Vill.) Schrad. ex Gaudin, *Myosotis nana* Vill.)

***Hackelia*** Opiz (*H. deflexa* (Wahlenb.) Opiz, *Myosotis deflexa* Wahlenb., *Cynoglossum deflexum* (Wahlenb.) Roth)

***Lappula*** Moench (*L. myosotis* Moench, *Myosotis lappula* L., *Cynoglossum lappula* (L.) Scop.) [= *L. squarrosa* (Retz.) Dumort.]

***Lepechiniella*** Popov [type not designated]

***Lobostemon*** Lehm. (*L. echioides* Lehm.)

***Microparacaryum*** (Popov ex Riedl) Hilger & Podlech (Paracaryum sect. Microparacaryum Popov ex Riedl) (*P. intermedium* (Fresen.) Lipsky, *Cynoglossum intermedium* Fresen., *M. intermedium* (Fresen.) Hilger & Podlech

***Moltkia*** Lehm. (*M. coerulea* (Willd.) Lehm., *Onosma coerulea* Willd.)

***Myosotidium*** Hook. (*M. nobile* (Hook. f.) Hook., *Cynoglossum nobile* Hook. f.) [*M. hortensia* (Decne.) Baill.]

***Myosotis*** L. (*M. scorpioides* L.)

*Omphalium* (Wallr.) Roth (*Cynoglossum* [unranked] *Omphalium* Wallr. ≡ ***Omphalodes*** Mill.) (*C. omphaloides* L., *Omphalodes verna* Moench, nom. illeg., nom. cons. prop. (Greuter et al. 2014), *Omphalium vernum* Roth, nom. illeg.)

***Omphalodes*** Mill. (*O. verna* Moench, nom. illeg., nom. cons. prop., Greuter et al. 2014, *Cynoglossum omphaloides* L., *O. omphaloides* (L.) Voss, nom. rej. prop., Greuter et al. 2014)

***Oncaglossum*** Sutorý (O. *pringlei* (Greenm.) Sutorý, *Cynoglossum pringlei* Greenm.)

***Onosma*** L. (*O. echioides* L.)

***Oreocarya*** Greene (*O. suffruticosa* (Torrey) Greene, *Myosotis suffruticosa* Torrey)

***Pectocarya*** DC. ex Meisn. (*P. lateriflora* (Lam.) DC., *Cynoglossum lateriflorum* Lam.)

*Picotia* Roem. & Schult., nom. illeg. (≡ ***Omphalodes*** Mill.) (*O. verna* Moench, nom. illeg., nom. cons. prop., Greuter et al. 2014, *Cynoglossum omphaloides* L., *O. omphaloides* (L.) Voss, nom. rej. prop.Greuter et al. 2014)

***Pseudomertensia*** Riedl (Lithospermum subg. Oreocharis Decne., *Oreocharis* (Decne.) Lindl., nom. rej.) (*P. elongata* (Decne.) Riedl, *L. elongatum* Decne.)

***Rochelia*** Rchb., nom. cons. (*R. saccharata* Rchb., nom. illeg., *Lithospermum dispermum* L. f., *R. disperma* (L. f.) Wettst.)

***Selkirkia*** Hemsl. (*S. berteroi* (Colla) Hemsl. (*Cynoglossum berteroi* Colla)

***Suchtelenia*** Kar. ex Meisn. (*S. cerinthifolia* Kar. ex Meisn., nom. illeg., *Cynoglossum calycinum* C. A. Mey., *S. calycina* (C. A. Mey.) A. DC.)

***Symphytum*** L. (*S. officinale* L.)

*Tysonia* Bolus, nom. illeg. (non *Tysonia* Fontaine) (≡ ***Afrotysonia*** Rauschert) (*T. africana* Bolus, *A. africana* (Bolus) Rauschert)

## Traits coverage

Designations resembling names but that have not been validly published, being no names in the sense of the ICN (McNeill et al. 2012), have no type and no taxonomic identity (even though they may have been applied to a given taxon): therefore they appear in the database in **grey type**. No attempt at a complete coverage of such designations has been made. By and large, only those have been retained that have been registered in IPNI, the purpose being to document their actual status of non-names. Most often, they are junior isonyms without nomenclatural status of their own; or in some cases, phantom entries of names not appearing at all in their alleged place of publication. Several were deliberately proposed as new but failed to meet the requirements for valid publication; they were sometimes validly published subsequently – perhaps unnoticed by others, including ourselves, in which case they will have to be added in the future, with their proper source.

### The data entries

The contents of the nomenclatural database for *Cynoglossum* sensu lato are shown in the electronic appendices Suppl. materials 1, 2) to this paper as a structured, fully searchable Hypertext Markup Language (HTML) file and a text file for import into as a spreadsheet or database. The entries in both files consist of eight elements, in which the data are connected by internal links and, where appropriate, linked to external sources. The main data fields, in the order in which they appear in each entry, are:

1. Headline (mandatory), showing the name with author citation and reference to the place and year of valid publication. Colour (green, orange, grey) and type (**bold-face** or normal) used in the headline are those explained above and show taxonomic placement, acceptability, and nomenclatural status (or absence thereof). With minor exceptions, all places of publication cited here have been verified by us. Abbreviation of name authors and publication titles follows the standards of IPNI. Correctable orthographical errors, when they appear in the original publication, are cited parenthetically between single quotes.

2. Field “IPNI” (mandatory except for new names and combinations validated in this paper): a direct copy of the current (November 2014) entry in the International Plant Name Index, underlain by a link to the entry itself (which may and hopefully in many cases will have been modified since that date). When IPNI shows more than one entry, a choice has been made as follows: When a recent entry without stated source exists, it is preferred; otherwise, when the type or original material of the name is from the Americas the Gray Card Index (GCI) entry has been selected; when it is from Australia, the Australian Plant Name Index (APNI) entry shows up; when it is from elsewhere, the Index Kewensis (IK) entry has been chosen; in case of more than one entry from the same source, the more recent, more accurate or more complete was given preference. Names not listed in IPNI at all are marked “absent”.

3. Field ASSESSMENT (mandatory for validly published names): In this field, each name is taxonomically referred to the name considered as correct under the option of *Cynoglossum* sensu lato being treated as a single genus. (A reliable assessment under a different option cannot presently be given because the definition and naming of natural segregate genera, even though it remains an option for the future, currently is not possible.) The name in the headline is either declared “accepted” or referred to the name considered as correct, if it exists; if none exists (in particular, when a taxon hitherto placed in *Cynoglossum* has been found not to belong to *Cynoglossum* sensu lato), the existing combination is used but with *“Cynoglossum”* placed between quotation marks. Names in the rank of variety or below are not normally accepted but assigned to a species or subspecies. In case of heterotypic synonymy, i.e., when the accepted name has a type different from that of the name in the headline, the former is placed between square brackets. Names appearing in the ASSESSMENT field are cross-linked to their own entry. Names that are not validly published remain unassessed, as they have no type hence no taxonomic identity.

4. Field STATUS (mandatory): The nomenclatural status of each name is indicated as follows. A legitimate name published as a name of a new taxon is defined by its rank, if any (e.g., *sect. nov., spec. nov., var. nov.* – or *taxon nov.*). A legitimate name with a basionym, which results from transfer of a former name to a new rank (both ranks being mentioned) and/or from its use in a new combination, is termed *comb. nov., stat. nov., comb. & stat. nov., comb. in stat. nov.*, as the case may be. A replacement name, based on a replaced synonym, is designated as *nom. nov.* An illegitimate name (*nom. illeg.*) is qualified as nomenclaturally superfluous [*superfl.*] and/or as a junior homonym [*homonym*] to show the cause of illegitimacy. A name or designation that had been validly published earlier than in the place cited in the headline is termed *isonym*; when it was not validly published in that place, as *inval.* [with mention of the requirements it failed to comply with]; and when it does not appear there at all, as *no name*

5. Field REFERENCE (optional): In this field a link to the protologue is included whenever that text is freely available online. Open-access sources are preferred whenever they exist. Links to journals available through JSTOR (http://www.jstor.org/), to which limited free access is granted to individual scientists, or to items offered by journal or book publishers only upon subscription or against payment of a fee, are also provided – but only to the generally accessible level (e.g., the title and abstract of the corresponding paper).For protologues in publications not (not yet) available online, ad-hoc scans (“clippings” in pdf format) were uploaded to the Biodiversity Literature Repository (https://zenodo.org/collection/user-biosyslit). This solution potentially makes all the treatments of all the taxa citable and the digital representation directly accessible, while not violating the copyright, if any, of the publication (Agosti and Egloff 2009; Patterson et al. 2014). BLR provides – as part of Zenodo/CERN – the long term digital repository. Digital Object Identifiers (DOI's) issued and maintained by DataCite (https://www.datacite.org/) allow the treatments to be easily cited and retrieved using a familiar, standard, and persistent identifier, and discoverable through search tools such as Refindit (http://refindit.org).

6. Field SYNONYMY (not mandatory): Synonymy is given once for every group of names based on the same type. The synonyms, chronologically arranged, appear either in the entry of the accepted name, or its basionym if it has one, or a replaced synonym if that is older. For each entry with one or more homotypic synonyms, a clickable cross-link to the entry including the synonymy is provided in the field BASIS, under “basionym” or “replaced synonym”. Names appearing in a SYNONYMY field are cross-linked to their own entry. For technical reasons, heterotypic synonyms (if any) are not included in these synonymies; they can be found with the help of the taxonomic equivalences given in the ASSESSMENT field in square brackets, when appropriate.

7. Field BASIS (mandatory for validly published names): For names of new taxa, this field includes information on the nomenclatural type, or relevant for its establishment if it has not been designated so far. It starts with the indication of *Original material*, comprising the verbatim citation [*from protologue*] of any indication, in the protologue itself, relevant for establishing what elements have been used by the author of the validating description or diagnosis; when no original material is mentioned in the protologue, this citation is replaced by an explanatory statement. Thereafter, whenever possible, concrete type specimens (e.g., *holotype, syntype, lectotype*), or original specimens or illustrations, are enumerated, with their herbarium of deposit and accession number, followed by an asterisk (*) when a digital image of the specimen can be consulted online via the Internet. Whenever traced, places of lectotype designation are mentioned. – For new combinations, names at new rank and replacement names, the BASIS field mentions the basionym and/or replaced synonym, cross-linked to their own entry. – Names that are not validly published lack a nomenclatural basis.

8. Field COMMENTS (optional): Free text in which any particular circumstances related to the listed name are mentioned, e.g. those relevant for their status, adopted spelling and authorship, taxonomic assessment and typification, in particular in (but not limited to) those cases in which our conclusions differ from those currently prevailing in the literature and/or indexes.

### Sources of information

As mentioned before, our main initial source of names was the International Plant Name Index (http://www.ipni.org). However, it soon appeared that IPNI is neither complete nor consistently accurate – which is not really a surprise but does not detract from the general usefulness and value of IPNI as a first-rate means of information on names and nomenclatural data. Yet, IPNI’s shortcomings must be mentioned here as a positive fact, as they were our initial trigger. They encouraged us to embark on our self-set task: to complete and refine the inventory of names relevant in the taxonomic context of *Cynoglossum* sensu lato. Had we guessed beforehand the amount of work involved we might well have desisted, but when it dawned upon us it was too late to back out.

Our second-level sources can best be described as the entire botanical literature, both shelved and, increasingly, available online. Direct verification of the source works registered in IPNI led on to any number of further relevant publications, and through them, to a ramified and interconnected wealth of published data. Only when the snowball effect causing that avalanche started to subside and we discovered less and less new, unrecorded data did we feel that completion of our task (while never possible at 100 %) was in sight.

During the implementation of our plans, when tracking down every reference found within and outside the IPNI dataset, we were greatly assisted by the modern search mechanisms now available online. Literature directories such as WorldCat (http://www.worldcat.org) and the Karlsruhe Virtual Catalogue (KVK; http://www.ubka.uni-karlsruhe.de/kvk.html) for books, and for journals the German Zeitschriftendatenbank (ZDB; http://dispatch.opac.dnb.de/DB=1.1), are invaluable tools, registering both physical publications with their location and virtual, digitised media with their access links. Other directories, concerned with online-accessible publications only, were also regularly consulted, in particular Biological Heritage Library (BHL, http://www.biodiversitylibrary.org), Botanicus (http://www.botanicus.org/), Biblioteca digital del Real Jardín Botánico [Madrid] (http://bibdigital.rjb.csic.es/ing/index.php), Gallica, bibliothèque numérique de la Bibliothèque Nationale de France (http://gallica.bnf.fr), Europeana (http://www.europeana.eu/), and several others. [For European users, access through Google Books (http://books.google.com) or HathiTrust Digital Library (http://www.hathitrust.org/digital_library) is a frustrating experience, as many titles are not accessible due to (real or alleged) copyright restrictions.]

The ever increasing presence in the Internet of digitised images of herbarium specimens, particularly types, has enabled us to add a feature to our data that would have been unthinkable a few years back: direct reference to type specimens and elements of the original material, usually with herbarium location and accession numbers, plus an indication by means of an asterisk (*) that an image has been consulted by us. The most complete and useful gateway to type specimen illustrations is JSTOR’s Global Plants (http://plants.jstor.org), which unfortunately is not accessible for free. Other important (and free) sources of images are institute-based and must be accessed individually, as there is no global portal to that information yet. Many important herbarium holdings are presently accessible online, at least in part, including Virtual Herbaria (W, WU, JE, etc.; http://herbarium.univie.ac.at/database/search.php), Muséum national d’histoire naturelle (P, MPU; http://coldb.mnhn.fr/colweb/form.do?model=SONNERAT.wwwsonnerat.wwwsonnerat.wwwsonnerat), Botanical Museum Berlin-Dahlem (B; http://ww2.bgbm.org/herbarium), Conservatoire botanique de la Ville de Genève (G; http://www.ville-ge.ch/musinfo/bd/cjb/chg/advanced.php), Linnean Society of London (LINN; http://linnean-online.org/linnaean_herbarium.html), Botany Portal of Field Museum (F, MO, NY; http://emuweb.fieldmuseum.org/botany/BotanyPortal/Query.php), Harvard University Herbaria (GH, etc.; http://kiki.huh.harvard.edu/databases/specimen_index.html), Smithsonian Institution Washington (US; http://collections.nmnh.si.edu/search/botany), Herbarium Centrale Italicum, Museo di Storia Naturale dell'Università die Firenze (FI; http://parlatore.msn.unifi.it/types/) etc.

Finally, mention must be made of Species 2000 and ITIS’ Catalogue of Life (CoL; http://www.catalogueoflife.org/col). While less complete than IPNI as an inventory of names, it has one quality that is absent from the latter: it advises on synonymy. During the last stage of editing our inventory, when we introduced taxonomic assessment of names into it, we have repeatedly consulted CoL’s dynamic checklist and found it a useful and usually, though not invariably, accurate guide in matters of taxonomic synonymy. Especially when the correct identity of non-*Cynoglossum* names had to be assessed, we mostly found ourselves in full agreement with the choices in CoL.

## Usage rights

### Use license

Creative Commons CCZero

## Additional information

The nomenclatural database for *Cynoglossum* sensu lato comprises 1338 entries: 209 (in grey) of names not validly published in the place cited (these include isonym entries for names previously validated elsewhere); 268 (in orange) of names of which the types do not belong to our core or target group but have been nomenclaturally or taxonomically associated with it in the past, in some way or other; and 861 (in green) that do really pertain. The statistical digest in Table 1 is limited to names in the green group, as this is the only one for which coverage is supposed to be complete.

From the very beginning, one of our self-set targets was a comparison of the information found by us with what is currently in the IPNI database. We wanted, for one, to get an idea of how reliable IPNI is and to which extent, and in which respect, it might be inaccurate and/or incomplete. Also, we had and keep the hope that, subsequent to their publication, our data will be of use for updating the IPNI record, as we know and appreciate the hard work of the IPNI staff, both at Kew and Harvard, to continually improve their entries.

We will not dwell on minor errors, including wrong numbers, some of which may well go back to weaknesses of the optical character recognition (OCR) software used when the original *Index Kewensis* volumes were scanned; nor on the failure to adopt IPNI’s own standards in many of the old entries, as we know that standardising them is among the ongoing concerns of the IPNI staff. Apart from such minor, trivial aspects, we found that IPNI currently has shortcomings principally in three fields.

1. Missing names. These are in their great majority found in ranks between genus and species and below species, which is explained by the fact that *Index Kewensis*, from which the bulk of IPNI entries originates, did not cover these ranks (contrary to the *Gray Card Index*). Only in recent years have infraspecific names and those of subdivisions of genera been registered consistently, and if it is judged worth while to fill that gap retroactively, this will constitute a gigantic task that will take decades to complete. Currently, for infraspecific names in particular, information in IPNI is fragmentary only. On the other hand, at the level of genera (94.4 %) and species (95.8 %) the coverage is reasonably good.

2. Inclusion of non-names. In a nomenclatural database, designations that are not validly published (some of which do not appear at all in the indicated place) should be either eliminated or – if maintained to avoid loss of information – prominently flagged. Current IPNI policy is to record them when they are intended as a nomenclatural novelty, but the author fails to comply with the conditions for valid publication, a fact that is then duly noted. But of old, such “names” were accepted at face value, and if and when validation finally occurred no note was taken. Worse, the early volumes of *Index Kewensis* are full of records of what appear to be junior homonyms, but are in fact mere misapplications of earlier names – a distinction that, while nomenclaturally relevant now, was not made at that time. This ballast, of what in terms of current nomenclature are junior isonyms without standing, lost whatever relevance it may once have had when it was decided to eliminate from the IPNI database the original reason of being of such entries: the sense in which the names had been misapplied, carefully recorded in *Index Kewensis.* As they are listed now, devoid of their rationale, all these isonyms are just only confusing, and a real nuisance. We hope that sooner or later they will be thrown over board.

3. Wrong assessment of the status of names, wrong author citations. For newly added names, IPNI is careful to distinguish legitimate from illegitimate ones, and new combinations or replacement names are referred to their basionym or replaced synonym – additionally, with increasing frequence, they are linked to outside sources available online. Old entries, however, have been updated only in part. The early volumes of *Index Kewensis* did not cite basionyms nor mention parenthetical authors, and illegitimacy at that time was an unheard-of concept. Updating such imperfect information is a big, demanding task for the future, requiring verification of the original source if it is to be properly done; we hope that the present inventory will be used for that purpose. Verification of author citations is a particular aspect of status assessment and is also necessary for former entries, because the relevant rules have changed substantially after the Tokyo Congress in 1993.

The problems just highlighted with respect to IPNI, the main existing nomenclatural data source for vascular plants, strengthen our belief in the usefulness of improved nomenclatural inventories such as the present one. We are conscious of the fact that no such list will ever be perfect, but we also believe that, beyond a certain point, aiming at an ever greater level of reliability becomes a futile exercise, an investment of time and money for little if any useful return. Once an inventory of this kind exists, it should be possible to confer official sanction to its basic contents, meaning certain parameters of the listed names (such as status, authorship, date and place of publication, spelling, etc.). We strongly advocate the creation of such a stabilising option under the rules governing nomenclature.

### Conclusions

As stated in the introduction, the authors of this paper are divided as to their preferred generic concept for *Cynoglossum* sensu lato. This is no disadvantage. On the contrary, it permits us to present our readers with a dual approach, allowing them to choose between alternative classifications.

The problem with narrowly defined genera, the option endorsed by HHH, is that it would be premature to present them in a firm classificatory frame. Traditional generic definitions, based mainly on fruit morphology, are clearly unnatural in some cases. A reassessment leading to the establishment of natural segregate units requires further in-depth morphological and expanded molecular studies. The solution presented here – the best that is currently feasible – is to declare as acceptable, or potentially correct, names that exist under any of the currently adopted genera. This does not of course preclude the treatment of some names as synonyms when they belong to a taxon (species or subspecies) for which an older name exists, but for the time being it prevents the validation of new, potentially correct combinations in cases when they are likely needed under the segregate-genera concept.

The option of treating *Cynoglossum* sensu lato as a single genus, favoured by WG and VS, is less prone to future change. It is certainly possible, indeed likely, that some species currently assigned to that genus will have to be excluded from it in the future, but in general terms the unit that has emerged as monophyletic (Selvi et al. 2011, Weigend et al. 2013) is well circumscribed. We have therefore decided to facilitate the practical implementation of this scenario by creating the required combinations that do not so far exist, as follows:

***Cynoglossum alaicum*** (Lazkov) Greuter & Stier, **comb. nov. [BDJ 2015, hoc loco]** ≡ *Rindera alaica* Lazkov in Bot. Zhurn. (Moskva & Leningrad) 85(8): 116. 2000. (IPNI urn:lsid:ipni.org:names:77146588-1)

***Cynoglossum albiflorum*** (Czukav. & Meling) Greuter & Stier, **comb. nov. [BDJ 2015, hoc loco]** ≡ *Solenanthus albiflorus* Czukav. & Meling in Novosti Sist. Vyssh. Rast. 19: 161. 1982. (IPNI urn:lsid:ipni.org:names:77146589-1)

***Cynoglossum anchusoides*** subsp. ***asperum*** (Rech. f.) Greuter & Stier, **comb. nov. [BDJ 2015, hoc loco]** ≡ *Lindelofia aspera* Rech. f. in Ann. Naturhist. Mus. Wien 58: 48. 1951 ≡ Lindelofia anchusoides subsp. aspera (Rech. f.) Sadat in Mitt. Bot. Staatssamml. München 28: 104. 1989. (IPNI urn:lsid:ipni.org:names:77146590-1)

***Cynoglossum asperum*** (Stocks) Greuter & Stier, **comb. nov. [BDJ 2015, hoc loco]** ≡ *Paracaryum asperum* Stocks in Hooker’s J. Bot. Kew Gard. Misc. 4: 175. 1852. (IPNI urn:lsid:ipni.org:names:77146591-1)

***Cynoglossum austroechinatum*** (Popov) Greuter & Stier, **comb. nov. [BDJ 2015, hoc loco]** ≡ *Rindera austroechinata* Popov in Bot. Mater. Gerb. Bot. Inst. Komarova Akad. Nauk S.S.S.R. 13: 222. 1950. (IPNI urn:lsid:ipni.org:names:77146592-1)

***Cynoglossum badghysii*** (Sadat) Greuter & Stier, **comb. nov. [BDJ 2015, hoc loco]** ≡ *Mattiastrum badghysii* Sadat in Mitt. Bot. Staatssamml. München 28: 67. 1989. (IPNI urn:lsid:ipni.org:names:77146593-1)

***Cynoglossum bakhtiaricum*** (Khat.) Greuter & Stier, **comb. nov. [BDJ 2015, hoc loco]** ≡*Solenanthus bakhtiaricus* Khat. in Iranian J. Bot. 8: 6. 1999. (IPNI urn:lsid:ipni.org:names:77146594-1)

***Cynoglossum brachystemon*** (Fisch. & C. A. Mey.) Greuter & Stier, **comb. nov. [BDJ 2015, hoc loco]** ≡*Solenanthus brachystemon* Fisch. & C. A. Mey. in Bull. Soc. Imp. Naturalistes Moscou 11: 306. 1838. (IPNI urn:lsid:ipni.org:names:77146595-1)

***Cynoglossum bungei*** (Boiss.) Greuter & Stier, **comb. nov. [BDJ 2015, hoc loco]** ≡*Mattia bungei* Boiss., Fl. Orient. 4: 274. 1875. (IPNI urn:lsid:ipni.org:names:77146596-1)

***Cynoglossum campanulatum*** (Riedl) Greuter & Stier, **comb. nov. [BDJ 2015, hoc loco]** ≡*Lindelofia campanulata* Riedl in Biol. Skr. 13: 198. 1963. (IPNI urn:lsid:ipni.org:names:77146597-1)

***Cynoglossum coechinatum*** (Popov) Greuter & Stier, **comb. nov. [BDJ 2015, hoc loco]** ≡*Rindera coechinata* Popov in Bot. Mater. Gerb. Bot. Inst. Komarova Akad. Nauk S.S.S.R. 13: 223. 1950. (IPNI urn:lsid:ipni.org:names:77146598-1)

***Cynoglossum crista-galli*** (Rech. f. & Riedl) Greuter & Stier, **comb. nov. [BDJ 2015, hoc loco]** ≡*Mattiastrum crista-galli* Rech. f. & Riedl in Biol. Skr. 13: 207. 1963. (IPNI urn:lsid:ipni.org:names:77146599-1)

***Cynoglossum cristulatum*** (Lipsky) Greuter & Stier, **comb. nov. [BDJ 2015, hoc loco]** ≡*Rindera cristulata* Lipsky in Trudy Imp. S.-Peterburgsk. Bot. Sada 26: 570. 1910. (IPNI urn:lsid:ipni.org:names:77146600-1)

***Cynoglossum cyclhymenium*** (Boiss.) Greuter & Stier, **comb. nov. [BDJ 2015, hoc loco]** ≡Paracaryum rugulosum var. cyclhymenium Boiss., Fl. Orient. 4: 256. 1875 ≡ *Paracaryum cyclhymenium* (Boiss.) Riedl in Rechinger, Fl. Iranica 48: 102. 1967. (IPNI urn:lsid:ipni.org:names:77146602-1)

***Cynoglossum cynoglossoides*** (Rech. f. & Riedl) Greuter & Stier, **comb. nov. [BDJ 2015, hoc loco]** ≡*Mattiastrum cynoglossoides* Rech. f. & Riedl in Rechinger, Fl. Iranica 48: 123. 1967. (IPNI urn:lsid:ipni.org:names:77146603-1)

***Cynoglossum densum*** (Rech. f. & Riedl) Greuter & Stier, **comb. nov. [BDJ 2015, hoc loco]** ≡*Mattiastrum densum* Rech. f. & Riedl in Oesterr. Bot. Z. 110: 519. 1963. (IPNI urn:lsid:ipni.org:names:77146604-1)

***Cynoglossum dielsii*** (Bornm.) Greuter & Stier, **comb. nov.** ≡ *Mattiastrum dielsii* Bornm. in Bot. Jahrb. Syst. 66: 236. 1934. (IPNI urn:lsid:ipni.org:names:77146607-1)

***Cynoglossum dieterlei*** (Sadat) Greuter & Stier, **comb. nov.** ≡ *Mattiastrum dieterlei* Sadat in Mitt. Bot. Staatssamml. München 28: 88. 1989. (IPNI urn:lsid:ipni.org:names:77146611-1)

***Cynoglossum dubium*** (Fisch. & C. A. Mey.) Greuter & Stier, **comb. nov.** ≡ *Solenanthus dubius* Fisch. & C. A. Mey. in Bull. Soc. Imp. Naturalistes Moscou 11: 306. 1838. (IPNI urn:lsid:ipni.org:names:77146616-1)

***Cynoglossum dumanii*** (Aytaç & R. R. Mill) Greuter & Stier, **comb. nov.** ≡ *Rindera dumanii* Aytaç & R. R. Mill in Edinburgh J. Bot. 61: 113. 2005. (IPNI urn:lsid:ipni.org:names:77146617-1)

***Cynoglossum eriocalycinum*** (Boiss. & Buhse) Greuter & Stier, **comb. nov.** ≡ *Solenanthus eriocalycinus* Boiss. & Buhse in Nouv. Mém. Soc. Imp. Naturalistes Moscou 12: 156. 1860. (IPNI urn:lsid:ipni.org:names:77146618-1)

***Cynoglossum ferganicum*** (Popov) Greuter & Stier, **comb. nov. [BDJ 2015, hoc loco]** ≡*Rindera ferganica* Popov in Bot. Mater. Gerb. Bot. Inst. Komarova Akad. Nauk S.S.S.R. 13: 219. 1950. (IPNI urn:lsid:ipni.org:names:77146619-1)

***Cynoglossum flaviflorum*** (Rech. f. & Riedl) Greuter & Stier, **comb. nov. [BDJ 2015, hoc loco]** ≡*Mattiastrum flaviflorum* Rech. f. & Riedl in Rechinger, Fl. Iranica 48: 122. 1967. (IPNI urn:lsid:ipni.org:names:77146620-1)

***Cynoglossum fornicatum*** (Pazij) Greuter & Stier, **comb. nov. [BDJ 2015, hoc loco]** ≡*Rindera fornicata* Pazij in Bot. Mater. Gerb. Inst. Bot. Akad. Nauk Uzbeksk. S.S.R. 15: 24. 1959. (IPNI urn:lsid:ipni.org:names:77146621-1)

***Cynoglossum glabratum*** (Pazij) Greuter & Stier, **comb. nov. [BDJ 2015, hoc loco]** ≡*Rindera glabrata* Pazij in Bot. Mater. Gerb. Inst. Bot. Akad. Nauk Uzbeksk. S.S.R. 15: 26. 1959. (IPNI urn:lsid:ipni.org:names:77146622-1)

***Cynoglossum glandulosum*** (Khat.) Greuter & Stier, **comb. nov. [BDJ 2015, hoc loco]** ≡*Paracaryum glandulosum* Khat. in Iranian J. Bot. 6: 228. 1994. (IPNI urn:lsid:ipni.org:names:77146623-1)

***Cynoglossum hedgei*** (Aytaç & R. R. Mill) Greuter & Stier, **comb. nov. [BDJ 2015, hoc loco]** ≡*Paracaryum hedgei* Aytaç & R. R. Mill in Edinburgh J. Bot. 61: 109. 2005. (IPNI urn:lsid:ipni.org:names:77146624-1)

***Cynoglossum heratense*** (Rech. f. & Riedl) Greuter & Stier, **comb. nov. [BDJ 2015, hoc loco]** ≡*Mattiastrum heratense* Rech. f. & Riedl in Biol. Skr. 13: 210. 1963. (IPNI urn:lsid:ipni.org:names:77146625-1)

***Cynoglossum himalayense*** (Klotzsch) Greuter & Stier, **comb. nov. [BDJ 2015, hoc loco]** ≡*Mattia himalayensis* Klotzsch in Klotzsch & Garcke, Bot. Ergebn. Reise Waldemar: 94. 1862. (IPNI urn:lsid:ipni.org:names:77146627-1)

***Cynoglossum hissaricum*** (Lipsky) Greuter & Stier, **comb. nov. [BDJ 2015, hoc loco]** ≡*Trachelanthus hissaricus* Lipsky in Trudy Imp. S.-Peterburgsk. Bot. Sada 23: 202. 1904. (IPNI urn:lsid:ipni.org:names:77146629-1)

***Cynoglossum holochiton*** (Popov) Greuter & Stier, **comb. nov. [BDJ 2015, hoc loco]** ≡ *Rindera holochiton* Popov in Komarov, Fl. SSSR 19: 716. 1953. (IPNI urn:lsid:ipni.org:names:77146631-1)

***Cynoglossum hupehense*** (R. R. Mill) Greuter & Stier, **comb. nov. [BDJ 2015, hoc loco]** ≡ *Solenanthus hupehensis* R. R. Mill in Notes Roy. Bot. Gard. Edinburgh 44: 271. 1987. (IPNI urn:lsid:ipni.org:names:77146633-1)

***Cynoglossum hystrix*** Greuter & Stier, **nom. nov. [BDJ 2015, hoc loco]** ≡ *Rindera echinata* Regel in Bull. Soc. Imp. Naturalistes Moscou 41(1): 92. 1868 (non *Cynoglossum echinatum* Thunb.). (IPNI urn:lsid:ipni.org:names:77146637-1)

***Cynoglossum indecorum*** Greuter & Stier, **nom. nov. [BDJ 2015, hoc loco]** ≡ *Solenanthus micranthus* Riedl in Ann. Naturhist. Mus. Wien 76: 635. 1972 (non *Cynoglossum micranthum* Poir.). (IPNI urn:lsid:ipni.org:names:77146640-1)

***Cynoglossum integerrimum*** (P. Myrzakulov) Greuter & Stier, **comb. nov. [BDJ 2015, hoc loco]** ≡ *Paracaryum integerrimum* P. Myrzakulov in Bot. Mater. Gerb. Bot. Inst. Bot. Acad. Nauk Kazakhsk. S.S.R. 9: 30. 1975. (IPNI urn:lsid:ipni.org:names:77146641-1)

***Cynoglossum karakoricum*** (Podlech & Sadat) Greuter & Stier, **comb. nov. [BDJ 2015, hoc loco]** ≡ *Mattiastrum karakoricum* Podlech & Sadat in Mitt. Bot. Staatssamml. München 27: 65. 1988. (IPNI urn:lsid:ipni.org:names:77146647-1)

***Cynoglossum karataviense*** (Pavlov ex Popov) Greuter & Stier, **comb. nov. [BDJ 2015, hoc loco]** ≡ *Paracaryum karataviense* Pavlov ex Popov in Komarov, Fl. URSS 19: 715. 1953. (IPNI urn:lsid:ipni.org:names:77146648-1)

***Cynoglossum karateginum*** (Lipsky) Greuter & Stier, **comb. nov. [BDJ 2015, hoc loco]** ≡ *Solenanthus karateginus* Lipsky in Trudy Imp. S.-Peterburgsk. Bot. Sada 23: 196. 1904. (IPNI urn:lsid:ipni.org:names:77146649-1)

***Cynoglossum khorassanicum*** (Khat.) Greuter & Stier, **comb. nov. [BDJ 2015, hoc loco]** ≡ *Paracaryum khorassanicum* Khat. in Iranian J. Bot. 8: 4. 1999. (IPNI urn:lsid:ipni.org:names:77146650-1)

***Cynoglossum kokanicum*** (Regel) Greuter & Stier, **comb. nov. [BDJ 2015, hoc loco]** ≡ *Solenanthus kokanicus* Regel in Izv. Imp. Obshch. Lyubit. Estestv. Moskovsk. Univ. 34(2) [Putesh. Turkest. 3(18)]: 60 (‘*olgae*’), 89. 1882. (IPNI urn:lsid:ipni.org:names:77146651-1)

***Cynoglossum korolkowii*** (Lipsky) Greuter & Stier, **comb. nov. [BDJ 2015, hoc loco]** ≡ *Trachelanthus korolkowii* Lipsky in Trudy Imp. S.-Peterburgsk. Bot. Sada 23: 199. 1904. (IPNI urn:lsid:ipni.org:names:77146652-1)

***Cynoglossum korshinskyi*** (Lipsky) Greuter & Stier, **comb. nov. [BDJ 2015, hoc loco]** ≡ *Cyphomattia korshinskyi* Lipsky in Trudy Imp. S.-Peterburgsk. Bot. Sada 26: 511. 1910. (IPNI urn:lsid:ipni.org:names:77146653-1)

***Cynoglossum krasniqii*** Wraber, **spec. nov. [BDJ 2015, hoc loco] –** Descr.: Wraber in Candollea 41: 145. 1986. – Holotype: Srbija, Kosovo: Paštrik supra vicum Gorožup prope oppidum Prizren, 1520 m s.m., 30.4.1983 (flor.), *T. Wraber* (BEO). (IPNI urn:lsid:ipni.org:names:77146654-1)

***Cynoglossum kuhitangicum*** (Raenko) Greuter & Stier, **comb. nov. [BDJ 2015, hoc loco]** ≡ *Rindera kuhitangica* Raenko in Novosti Sist. Vyssh. Rast. 34: 151. 2002. (IPNI urn:lsid:ipni.org:names:77146655-1)

***Cynoglossum kuramense*** (Turak.) Greuter & Stier, **comb. nov. [BDJ 2015, hoc loco]** ≡ *Rindera kuramensis* Turak. in Novosti Sist. Vyssh. Rast. 28: 131. 1991. (IPNI urn:lsid:ipni.org:names:77146656-1)

***Cynoglossum lambertianum*** (C. B. Clarke) Greuter & Stier, **comb. nov. [BDJ 2015, hoc loco]** ≡ *Paracaryum lambertianum* C. B. Clarke in Hooker, Fl. Brit. India 4: 161. 1883. (IPNI urn:lsid:ipni.org:names:77146657-1)

***Cynoglossum longifolium*** (Leichtlin ex Beck & F. Abel) Greuter & Stier, **comb. nov. [BDJ 2015, hoc loco]** ≡ *Lindelofia longifolia* Leichtlin ex Beck & F. Abel in Wiener Ill. Gart.-Zeitung 13: 326. 1888. (IPNI urn:lsid:ipni.org:names:77146658-1)

***Cynoglossum longipedicellatum*** (Riedl) Greuter & Stier, **comb. nov. [BDJ 2015, hoc loco]** ≡ *Lindelofia longipedicellata* Riedl in Biol. Skr. 13: 199. 1963. (IPNI urn:lsid:ipni.org:names:77146659-1)

***Cynoglossum luristanicum*** (Nábělek) Greuter & Stier, **comb. nov. [BDJ 2015, hoc loco]** ≡ *Paracaryum luristanicum* Nábělek in Spisy Přír. Fak. Masarykovy Univ. 70: 23. 1926. (IPNI urn:lsid:ipni.org:names:77146660-1)

***Cynoglossum medium*** (Turrill) Greuter & Stier, **comb. nov. [BDJ 2015, hoc loco]** ≡ *Bilegnum medium* Turrill in Bull. Misc. Inform. Kew 1929: 232. 1929. (IPNI urn:lsid:ipni.org:names:77146661-1)

***Cynoglossum minimum*** (Brand) Greuter & Stier, **comb. nov. [BDJ 2015, hoc loco]** ≡ *Solenanthus minimus* Brand in Repert. Spec. Nov. Regni Veg. 13: 547. 1915. (IPNI urn:lsid:ipni.org:names:77146662-1)

***Cynoglossum minutiflorum*** Greuter & Stier, **nom. nov. [BDJ 2015, hoc loco]** ≡ *Lindelofia micrantha* Rech. f. & Riedl in Rechinger, Fl. Iranica 48: 139. 1967 (non *Cynoglossum micranthum* Poir.). (IPNI urn:lsid:ipni.org:names:77146663-1)

***Cynoglossum modestum*** (Boiss. & Hausskn.) Greuter & Stier, **comb. nov. [BDJ 2015, hoc loco]** ≡ *Paracaryum modestum* Boiss. & Hausskn. in Boissier, Pl. Or. Nov. Dec. 2: 5. 1875. (IPNI urn:lsid:ipni.org:names:77146664-1)

***Cynoglossum multicaule*** (Rech. f. & Riedl) Greuter & Stier, **comb. nov. [BDJ 2015, hoc loco]** ≡ *Mattiastrum multicaule* Rech. f. & Riedl in Biol. Skr. 13: 216. 1963. (IPNI urn:lsid:ipni.org:names:77146665-1)

***Cynoglossum nebulicola*** (R. R. Mill) Greuter & Stier, **comb. nov. [BDJ 2015, hoc loco]** ≡ *Adelocaryum nebulicola* R. R. Mill in Edinburgh J. Bot. 67: 148. 2010. (IPNI urn:lsid:ipni.org:names:77146666-1)

***Cynoglossum neubaueri*** (Rech. f.) Greuter & Stier, **comb. nov. [BDJ 2015, hoc loco]** ≡ *Moltkia neubaueri* Rech. f. in Ann. Naturhist. Mus. Wien 58: 57. 1951. (IPNI urn:lsid:ipni.org:names:77146667-1)

***Cynoglossum nigrum*** (Riedl) Greuter & Stier, **comb. nov. [BDJ 2015, hoc loco]** ≡ *Mattiastrum nigrum* Riedl in Rechinger, Fl. Iranica 48: 118. 1967. (IPNI urn:lsid:ipni.org:names:77146668-1)

***Cynoglossum oblongifolium*** (Popov) Greuter & Stier, **comb. nov. [BDJ 2015, hoc loco]** ≡ *Rindera oblongifolia* Popov in Bot. Mater. Gerb. Bot. Inst. Komarova Akad. Nauk S.S.S.R. 13: 224. 1950. (IPNI urn:lsid:ipni.org:names:77146669-1)

***Cynoglossum ochroleucum*** (Kar. & Kir.) Greuter & Stier, **comb. nov. [BDJ 2015, hoc loco]** ≡ *Rindera ochroleuca* Kar. & Kir. in Bull. Soc. Imp. Naturalistes Moscou 15: 408. 1842. (IPNI urn:lsid:ipni.org:names:77146670-1)

***Cynoglossum olgae*** (Regel & Smirn.) Greuter & Stier, **comb. nov. [BDJ 2015, hoc loco]** ≡ *Solenanthus olgae* Regel & Smirn. in Izv. Imp. Obshch. Lyubit. Estestv. Moskovsk. Univ. 34(2) [Putesh. Turkest. 3(18)]: 59. 1882. (IPNI urn:lsid:ipni.org:names:77146671-1)

***Cynoglossum oschense*** (Popov) Greuter & Stier, **comb. nov. [BDJ 2015, hoc loco]** ≡ *Rindera oschensis* Popov in Bot. Mater. Gerb. Bot. Inst. Komarova Akad. Nauk S.S.S.R. 13: 220. 1950. (IPNI urn:lsid:ipni.org:names:77146672-1)

***Cynoglossum persicum*** (Boiss.) Greuter & Stier, **comb. nov. [BDJ 2015, hoc loco]** ≡ *Omphalodes persica* Boiss., Diagn. Pl. Orient., ser. 1, 7: 30. 1846. (IPNI urn:lsid:ipni.org:names:77146673-1)

***Cynoglossum phardycalyx*** Greuter & Stier, **nom. nov. [BDJ 2015, hoc loco]** ≡ *Paracaryum platycalyx* Riedl in Oesterr. Bot. Z. 110: 535. 1963 (non *Cynoglossum platycalyx* (Riedl) Greuter & Stier). (IPNI urn:lsid:ipni.org:names:77146674-1)

***Cynoglossum plantaginifolium*** (Lipsky) Greuter & Stier, **comb. nov. [BDJ 2015, hoc loco]** ≡ *Solenanthus plantaginifolius* Lipsky in Trudy Imp. S.-Peterburgsk. Bot. Sada 23: 198. 1904. (IPNI urn:lsid:ipni.org:names:77146675-1)

***Cynoglossum platycalyx*** (Riedl) Greuter & Stier, **comb. nov. [BDJ 2015, hoc loco]** ≡ *Lindelofia platycalyx* Riedl in Biol. Skr. 13: 202. 1963. (IPNI urn:lsid:ipni.org:names:77146676-1)

***Cynoglossum polyanthum*** (Rech. f. & Riedl) Greuter & Stier, **comb. nov. [BDJ 2015, hoc loco]** ≡ *Mattiastrum polyanthum* Rech. f. & Riedl in Rechinger, Fl. Iranica 48: 123. 1967. (IPNI urn:lsid:ipni.org:names:77146677-1)

***Cynoglossum pygmaeum*** (Rech. f.) Greuter & Stier, **comb. nov. [BDJ 2015, hoc loco]** ≡ *Mattiastrum pygmaeum* Rech. f. in Ann. Naturhist. Mus. Wien 58: 52. 1951. (IPNI urn:lsid:ipni.org:names:77146678-1)

***Cynoglossum rechingeri*** Greuter & Stier, **nom. nov. [BDJ 2015, hoc loco]** ≡ *Mattiastrum formosum* Rech. f. & Riedl in Rechinger, Fl. Iranica 48: 119. 1967 (non *Cynoglossum formosum* (R. R. Mill) Greuter & Burdet). (IPNI urn:lsid:ipni.org:names:77146679-1)

***Cynoglossum regelii*** Greuter & Stier, **nom. nov. [BDJ 2015, hoc loco]** ≡ *Solenanthus hirsutus* Regel in Izv. Imp. Obshch. Lyubit. Estestv. Moskovsk. Univ. 34(2) [Putesh. Turkest. 3(18)]: 60. 1882 (non *Cynoglossum hirsutum* Thunb.). (IPNI urn:lsid:ipni.org:names:77146680-1)

***Cynoglossum regium*** (S. G. Gmel.) Greuter & Stier, **comb. nov. [BDJ 2015, hoc loco]** ≡ *Symphytum regium* S. G. Gmel., Reise Russland 3: 363, t. 36. f. 1. 1774. (IPNI urn:lsid:ipni.org:names:77146684-1)

***Cynoglossum sessiliflorum*** (Rech. f. & Riedl) Greuter & Stier, **comb. nov. [BDJ 2015, hoc loco]** ≡ *Mattiastrum sessiliflorum* Rech. f. & Riedl in Rechinger, Fl. Iranica 48: 125. 1967. (IPNI urn:lsid:ipni.org:names:77146685-1)

***Cynoglossum strictissimum*** (Brand) Greuter & Stier, **comb. nov. [BDJ 2015, hoc loco]** ≡ *Solenanthus strictissimus* Brand in Repert. Spec. Nov. Regni Veg. 13: 546. 1915. (IPNI urn:lsid:ipni.org:names:77146686-1)

***Cynoglossum stylosum*** subsp. ***pterocarpum*** (Rupr.) Greuter & Stier, **comb. nov. [BDJ 2015, hoc loco]** ≡ Solenanthus nigricans var. pterocarpus Rupr. in Mém. Acad. Imp. Sci. St.-Pétersbourg, ser. 7, 14: 62. 1869 ≡ Lindelofia stylosa subsp. pterocarpa (Rupr.) Kamelin in Novon 3: 263. 1993. (IPNI urn:lsid:ipni.org:names:77146687-1)

***Cynoglossum subscaposum*** (Rech. f. & Riedl) Greuter & Stier, **comb. nov. [BDJ 2015, hoc loco]** ≡ *Mattiastrum subscaposum* Rech. f. & Riedl in Rechinger, Fl. Iranica 48: 120. 1967. (IPNI urn:lsid:ipni.org:names:77146688-1)

***Cynoglossum tenerum*** (Bornm.) Greuter & Stier, **comb. nov. [BDJ 2015, hoc loco]** ≡ *Paracaryum tenerum* Bornm. in Beih. Bot. Centralbl., Abt. 2, 33: 175. 1915. (IPNI urn:lsid:ipni.org:names:77146689-1)

***Cynoglossum trinervium*** (Duthie) Greuter & Stier, **comb. nov. [BDJ 2015, hoc loco]** ≡ *Paracaryum trinervium* Duthie in Bull. Misc. Inform. Kew 1912: 39. 1912. (IPNI urn:lsid:ipni.org:names:77146690-1)

***Cynoglossum tschotkalense*** (Popov) Greuter & Stier, **comb. nov. [BDJ 2015, hoc loco]** ≡ *Rindera tschotkalensis* Popov in Bot. Mater. Gerb. Bot. Inst. Komarova Akad. Nauk S.S.S.R. 13: 217. 1950. (IPNI urn:lsid:ipni.org:names:77146691-1)

***Cynoglossum turcomanicum*** (Bornm. & Sint.) Greuter & Stier, **comb. nov. [BDJ 2015, hoc loco]** ≡ *Paracaryum turcomanicum* Bornm. & Sint. in Beih. Bot. Centralbl., Abt. 2, 20: 193. 1906. (IPNI urn:lsid:ipni.org:names:77146692-1)

***Cynoglossum turkestanicum*** (Regel) Greuter & Stier, **comb. nov. [BDJ 2015, hoc loco]** ≡ *Kuschakewiczia turkestanica* Regel in Trudy Imp. S.-Peterburgsk. Bot. Sada 5: 626. 1877. (IPNI urn:lsid:ipni.org:names:77146693-1)

Concerning nomenclatural types, we have as a rule limited ourselves to (a) record lectotype designations that we noted during our searches (well aware of the fact that such a search can never be complete and fully reliable), or (b), when no such designation is known, to provide data (as explained under “Data entries”, Field BASIS) on which future specialists may base their choice. In the following exceptional cases, new type designations are being effected here:

***Cynoglossum officinale*** var. ***corsicum*** E. Rev. ex Brand – Lectotype (designated here): Fl. Corsica: Evisa, [6 July] 1885, *Reverchon* in Baenitz, Herbarium europaeum, FR (FR00360781!).

***Paracaryum*** sect. ***Mattiastrum*** Boiss. – Type (designated here): *P. aucheri* (A. DC.) Boiss. (*Mattia aucheri* A. DC., *Mattiastrum aucheri* (A. DC.) Brand, *Cynoglossum aucheri* (A. DC.) Greuter & Burdet).

Additionally we correct an erroneous name:

***Microparacaryum intermedium*** f. ***stellatum*** (Riedl) Hilger & Podlech, **stat. nov**. **[BDJ 2015, hoc loco]** ≡ *Paracaryum stellatum* Riedl in Rechinger, Fl. Iranica 48: 104. 1967 ≡ Microparacaryum intermedium var. stellatum (Riedl) Hilger & Podlech in Pl. Syst. Evol. 148: 304. 1985 ≡ Microparacaryum intermedium f. paracaryoides Hilger & Podlech in Pl. Syst. Evol. 148: 305. 1985 (non Microparacaryum intermedium f. paracaryoides Hilger & Podlech in Pl. Syst. Evol. 148: 303. 1985). (IPNI urn:lsid:ipni.org:names:77146694-1)

## Supplementary Material

Supplementary material 1 Nomenclatural database for Cynoglossum s.l. Data type: list
Brief description: Nomenclatural database for *Cynoglossum* sensu lato - a structured, fully searchable Hypertext Markup Language (HTML) file with names connected by internal links and, where appropriate, linked to external sources.
File: oo_42064.html Hilger, H.H.; Greuter, W.; Stier, V.

Supplementary material 2 Machine readable nomenclatural database for Cynoglossum s.l. Data type: list
Brief description: Nomenclatural database for *Cynoglossum* sensu lato - a structured text file for import, e.g., in MS Excel.
File: oo_42065.txt Hilger, H.H.; Greuter, W.; Stier, V.

## Figures and Tables

**Table 1.** Numbers of taxa and names in *Cynoglossum* sensu lato.

	**accepted** **taxa**	**potentially** **accepted** **names**	**synonyms**	**(illegitimate)**	**validly** **published** **names**	**not in IPNI**
Genus	1	9	9	2	18	1
Subgenus	0	0	10	0	10	5
Section	0	0	27	0	27	26
Subsection	0	0	3	0	3	3
Series	0	0	0	0	0	0
Unranked (supraspecific)	0	0	1	0	1	1
Species (incl. nothospecies)	204	407	205	26	612	26
Subspecies	10	16	9	0	25	7
Variety	0	0	138	5	138	124
Subvariety	0	0	5	0	5	5
Forma	0	0	20	1	20	16
Unranked (infraspecific)	0	0	5	0	5	5
**Total**	**215**	**432**	**432**	**34**	**864**	**219**

## References

[B1237309] Agosti D., Egloff W. (2009). Taxonomic information exchange and copyright: the Plazi approach. BMC Research Notes.

[B1214038] Greuter W. (1981). Med-checklist notulae, 3. Willdenowia.

[B1214078] Greuter Werner, Stier Victoria, Hilger Hartmut H. (2014). (2280) Proposal to conserve the name *Omphalodes
verna* against *Omphalodes
omphaloides* (*Boraginaceae*). Taxon.

[B1213895] McNeill J, Barrie FR, Buck WR, Demoulin V, Greuter W, Hawksworth DL, Herendeen PS, Knapp S, Marhold K, Prado J, Prud'homme van Reine WF, Smith GF, Wiersema JH, Turland NJ (2012). International Code of Nomenclature for algae, fungi, and plants (Melbourne Code) adopted by the Eighteenth International Botanical Congress Melbourne, Australia, July 2011..

[B1237319] Patterson David J, Egloff Willi, Agosti Donat, Eades David, Franz Nico, Hagedorn Gregor, Rees Jonathan A, Remsen David P (2014). Scientific names of organisms: attribution, rights, and licensing. BMC Research Notes.

[B1213914] Popov M G, Komarov VL (1953). Boraginaceae. Flora SSSR.

[B1214527] Riedl H., Rechinger K H (1967). Boraginaceae. Flora iranica.

[B1213864] Selvi F., Coppi A., Cecchi L. (2011). High epizoochorous specialization and low DNA sequence divergence in Mediterranean Cynoglossum (Boraginaceae): Evidence from fruit traits and ITS region. Taxon.

[B1213874] Weigend M., Luebert F., Selvi F., G. Brokamp, Hilger H. H. (2013). Multiple origins for hound’s tongues (*Cynoglossum* L.) and navel seeds (*Omphalodes* Mill.) – the phylogeny of the borage family (*Boraginaceae* s. str.). Molec. Phylog. Evol..

