# Supplementary material for: Taxa and names in Cynoglossum sensu lato (Boraginaceae, Cynoglosseae): an annotated, synonymic inventory, with links to the protologues and mention of original material
Source: Biodivers Data J. 2015 Apr 22;(3):e4831. doi: 10.3897/BDJ.3.e4831 (PMC4411491; doi:10.3897/BDJ.3.e4831)
Supplement: Supplementary material 1 — Nomenclatural database for Cynoglossum s.l. [file biodiversity_data_journal-3-e4831-s001.html]

Beschreibung der Seite


*Adelocaryum* Brand in Repert. Spec. Nov. Regni Veg. 13: 547. 1915

- IPNI: Adelocaryum Brand -- Repert. Spec. Nov. Regni Veg. 13: 547. 1915 (IK)
- ASSESSMENT: [*Cynoglossum* L.]
- STATUS: gen. nov.
- REFERENCE: Link
- BASIS: Type (Riedl in Linzer Biol. Beitr. 24: 21. 1992): *Adelocaryum coelestinum* (Lindl.) Brand
- COMMENTS: See type discussion by Mill in Edinburgh J. Bot. 67: 141—154. 2010.

---

*Adelocaryum anchusoides* (Lindl.) Brand in Repert. Spec. Nov. Regni Veg. 13: 548. 1915

- IPNI: Adelocaryum anchusoides (Lindl.) Brand -- Repert. Spec. Nov. Regni Veg. 13: 548. 1915 (IK)
- ASSESSMENT: *Cynoglossum anchusoides* Lindl.
- STATUS: comb. nov.
- REFERENCE: Link
- BASIS: Basionym: *Cynoglossum anchusoides* Lindl.

---

*Adelocaryum capusii* (Franch.) Brand in Repert. Spec. Nov. Regni Veg. 13: 548. 1915

- IPNI: Adelocaryum capusii Brand -- Repert. Spec. Nov. Regni Veg. 13: 548. 1915 (IK)
- ASSESSMENT: *Cynoglossum capusii* (Franch.) Pazij
- STATUS: comb. nov.
- REFERENCE: Link
- BASIS: Basionym: *Paracaryum capusii* Franch.

---

*Adelocaryum coelestinum* (Lindl.) Brand in Repert. Spec. Nov. Regni Veg. 13: 549. 1915

- IPNI: Adelocaryum coelestinum Brand -- Repert. Spec. Nov. Regni Veg. 13: 549. 1915 (IK)
- ASSESSMENT: *Cynoglossum coelestinum* Lindl.
- STATUS: comb. nov.
- REFERENCE: Link
- BASIS: Basionym: *Cynoglossum coelestinum* Lindl.

---

*Adelocaryum erythraeum* Brand in Engler, Pflanzenr. 78 (IV.252): 78. 1921

- IPNI: Adelocaryum erythraeum Brand -- Pflanzenr. (Engler) Borrag. Cynogloss. 78. 1921 (IK)
- ASSESSMENT: provisionally: *Brandella erythraea* (Brand) R. R. Mill [combination under *Microparacaryum* not yet published]
- STATUS: spec. nov.
- REFERENCE: Link
- SYNONYMY:   
  ≡ *Cynoglossum erythraeum* (Brand) Riedl 1971
    
  ≡ *Brandella erythraea* (Brand) R. R. Mill 1986
- BASIS: Original material: Nordostafrikanische Hochland- und Steppenprovinz (Colonia Eritrea): östlich von Amba-Tokhân, 398 m, 27.2.1892 (Schweinfurth und Riva n. 615 Herb. Boissier); oberer Teil des Tales Mogod, 1400 m, 8.4.1892 (Schweinfurth und Riva n. 1594, Herb. Boissier) [from protologue]. Lectotype (Riedl in Linzer Biol. Beitr. 24: 26. 1992): Schweinfurth & Riva 1594 (G). \*

---

*Adelocaryum erythraeum* f. *subexalatum* Riedl in Linzer Biol. Beitr. 24: 26. 1992 *(‘subexalata’)*

- IPNI: Adelocaryum erythraeum Brand f. subexalatum Riedl -- Linzer Biol. Beitr. 24(1): 26. 1992 [17 Jul 1992]
- ASSESSMENT: provisionally: [*Brandella erythraea* (Brand) R. R. Mill] [combination under *Microparacaryum* not yet published]
- STATUS: f. nov.
- REFERENCE: Link
- BASIS: Holotype: Abyssinia, Dschadscha (6000'), 29.10.1854, Schimper 362 (FI) [from protologue]

---

*Adelocaryum flexuosum* Brand in Repert. Spec. Nov. Regni Veg. 14: 149. 1915

- IPNI: Adelocaryum flexuosum Brand -- Repert. Spec. Nov. Regni Veg. 14: 149. 1915 (IK)
- ASSESSMENT: *Cynoglossum flexuosum* (Brand) Riedl
- STATUS: spec. nov.
- REFERENCE: Link
- SYNONYMY:   
  ≡ *Cynoglossum flexuosum* (Brand) Riedl 1971 [Feb]
- BASIS: Holotype: Kashmir: Gurés (nördlich von Srinágar, der Hauptstadt von Kaschmir), 3. und 4.10.1856, Schlagintweit 7612 (Breslau [WRCL]) [from protologue]

---

*Adelocaryum lambertianum* (C. B. Clarke) R. R. Mill in Edinburgh J. Bot. 67: 152. 2010

- IPNI: Adelocaryum lambertianum (C.B.Clarke) R.R.Mill -- Edinburgh J. Bot. 67(1): 152. 2010 [16 Feb 2010]
- ASSESSMENT: *Cynoglossum lambertianum* (C. B. Clarke) Greuter & Stier
- STATUS: comb. nov.
- REFERENCE: Link
- BASIS: Basionym: *Paracaryum lambertianum* C. B. Clarke

---

*Adelocaryum malabaricum* (C. B. Clarke) Brand in Repert. Spec. Nov. Regni Veg. 13: 549. 1915

- IPNI: Adelocaryum malabaricum Brand -- Repert. Spec. Nov. Regni Veg. 13: 549. 1915 (IK)
- ASSESSMENT: *Cynoglossum malabaricum* (C. B. Clarke) Riedl
- STATUS: comb. nov.
- REFERENCE: Link
- BASIS: Basionym: *Paracaryum malabaricum* C. B. Clarke

---

*Adelocaryum nebulicola*  R. R. Mill in Edinburgh J. Bot. 67: 148. 2010

- IPNI: Adelocaryum nebulicola R.R.Mill -- Edinburgh J. Bot. 67(1): 148 (-152; figs. 1-2). 2010 [16 Feb 2010]
- ASSESSMENT: *Cynoglossum nebulicola* (R. R. Mill) Greuter & Stier
- STATUS: spec. nov.
- REFERENCE: Link
- SYNONYMY:   
  ≡ *Cynoglossum nebulicola* (R. R. Mill) Greuter & Stier 2015
- BASIS: Holotype: Sultanate of Oman, Dhofar, Jebel Qara, Salallah to Ashanhaib road, c. 10km S of Ashanhaib, 800m, 6.9.1989, A.G. Miller & J.A. Nyberg M. 9131 (E; isotypes: K, KTUH, ON) [from protologue] \*

---

*Adelocaryum schlagintweitii* Brand in Repert. Spec. Nov. Regni Veg. 13: 548. 1915

- IPNI: Adelocaryum schlagintweitii Brand -- Repert. Spec. Nov. Regni Veg. 13: 548. 1915 (IK)
- ASSESSMENT: *Cynoglossum schlagintweitii* (Brand) Riedl
- STATUS: spec. nov.
- REFERENCE: Link
- SYNONYMY:   
  ≡ *Cynoglossum schlagintweitii* (Brand) Riedl 1971 [Feb]
- BASIS: Original material: Tibet: Provinz Dras: Kargil, zwischen Suru und Tsringmat (Schlagintweit no. 7211) [from protologue]. Lectotype (Kazmi in J. Arnold Arbor. 52: 352. 1971): Schlagintweit 7211 (WRCL)

---

*Afrotysonia* Rauschert in Taxon 31: 558. 1982

- IPNI: Afrotysonia Rauschert -- Taxon 31(3): 558 (1982), nom. nov. (IK)
- ASSESSMENT: accepted
- STATUS: nom. nov.
- REFERENCE: Link
- BASIS: *Tysonia* Bolus (1890), non *Tysonia* Fontaine (1889)
    
  TYPE: *Afrotysonia africana* (Bolus) Rauschert (*Tysonia africana* Bolus)

---

*Afrotysonia africana* (Bolus) Rauschert in Taxon 31: 558. 1982

- IPNI: Afrotysonia africana (Bolus) Rauschert -- Taxon 31(3): 558 (1982):. (IK)
- ASSESSMENT: accepted
- STATUS: comb. nov.
- REFERENCE: Link
- BASIS: Basionym: *Tysonia africana* Bolus

---

*Afrotysonia glochidiata* (R. R. Mill) R. R. Mill in Notes Roy. Bot. Gard. Edinburgh 43: 470. 1986

- IPNI: Afrotysonia glochidiata (R.Mill) R.R.Mill -- Notes Roy. Bot. Gard. Edinburgh 43(3): 470. 1986 (IK)
- ASSESSMENT: accepted
- STATUS: comb. nov.
- REFERENCE: Link
- BASIS: Basionym: *Tysonia glochidiata* R. R. Mill

---

*Afrotysonia pilosicaulis* R. R. Mill in Notes Roy. Bot. Gard. Edinburgh 43: 472. 1986

- IPNI: Afrotysonia pilosicaulis R.R.Mill -- Notes Roy. Bot. Gard. Edinburgh 43(3): 472. 1986 (IK)
- ASSESSMENT: accepted
- STATUS: spec. nov.
- REFERENCE: Link
- BASIS: Holotype: Tanzania: Ufipa distr., Sumbawanga, Mbesi forest, in very rough grassland, stem leaves broad, calyx greyish, hairy, flowers white, 2400m, 13.3.1957, H.M.Richards 8679 (K) [from protologue] \*

---

*Anchusa lanata* L., Syst. Nat., ed. 10: 914. 1759

- IPNI: Anchusa lanata L. -- Syst. Nat., ed. 10. 2: 914. 1759 [7 Jun 1759] (IK)
- ASSESSMENT: *Cynoglossum mathezii* Greuter & Burdet
- STATUS: spec. nov.
- REFERENCE: Link
- SYNONYMY:   
  ≡ *Cynoglossum cheirifolium* var. *lanatum* (L.) Lehm. 1818
    
  ≡ *Solenanthus lanatus* (L.) A. DC. 1846
    
  ≡ *Lindelofia lanata* (L.) Brand 1921
    
  ≡ *Pardoglossum lanatum* (L.) Barbier & Mathez 1973
    
  ≡ *Cynoglossum mathezii* Greuter & Burdet 1981
- BASIS: Original material: *Brander* [Original]. Lectotype (Mill in Taxon 53: 800. 2004): Algir., Herb. Original material: Linn. No. 182.6 (LINN\*)
- COMMENTS: Non *Cynoglossum lanatum* Lam. (1786).

---

*Anchusa petiolata* Hook. in Bot. Mag.: ad t. 3858. 1841

- IPNI: Anchusa petiolata Hook. -- Bot. Mag. 67: t. 3858. 1841 (IK)
- ASSESSMENT: [*Cynoglossum microglochin* Royle ex Benth.]
- STATUS: spec. nov.
- REFERENCE: Link
- SYNONYMY:   
  ≡ *Cynoglossum petiolatum* (Hook.) A. DC. 1846
    
  ≡ *Cynoglossum nervosum* var. *petiolatum* (Hook.) Kazmi 1971
- BASIS: Original material: raised by Mr. Murray, in the Glasgow Botanic Garden, from seeds sent from Nepal by Colonel Colvin … flowered in October 1840 [from protologue]

---

*Anchusa zeylanica* J. Jacq., Ecl. Pl. Rar. 1: 47. 1812

- IPNI: Anchusa zeylanica J.Jacq. -- Ecl. Pl. Rar. i. 47. t. 29. (IK)
- ASSESSMENT: *Bothriospermum zeylanicum* (J. Jacq.) Druce
- STATUS: spec. nov.
- REFERENCE: Link
- SYNONYMY:   
  ≡ *Bothriospermum zeylanicum* (J. Jacq.) Druce 1917
- BASIS: Original material: der hiesige Universitäts-Garten erhielt sie (1811) durch Samen, von Herrn Professor Bernhardi in Erfurt [from protologue]

---

*Anchusa zeylanica* Vahl ex Hornem., Hort. Bot. Hafn. 1: 176. 1813

- IPNI: Anchusa zeylanica Vahl ex Hornem. -- Hort. Bot. Hafn. i. 176. 1813 (IK)
- ASSESSMENT: *Cynoglossum zeylanicum* (Lehm.) Brand
- STATUS: nom. illeg. [homonym]
- REFERENCE: Link
- SYNONYMY:   
  ≡ *Myosotis zeylanica*  Lehm.1817
    
  ≡ *Echinospermum zeylanicum* (Lehm.) Lehm. 1818
    
  ≡ *Cynoglossum denticulatum* var. *zeylanicum* (Lehm.) Clarke 1883
    
  ≡ *Cynoglossum zeylanicum* (Lehm.) Brand 1915
- BASIS: Original material: in Herb. Vahl asservatur sub nomine *Anch. zeylanicae* [from protologue]. Holotype: C (foto in Kew Bull. 43: 344. 1988); probable isotype: [Sri Lanka] ad littora maris solo arenoso inter Gale et Mature, *Koenig*, L (foto in Kew Bull.43: 346. 1988)
- COMMENTS: First published as a nom. nud. in Hornem., Enum. Pl. Hort. Hafn. 1807 (not seen; see Verdcourt in Kew Bull. 43: 343. 1988); then with a minimal description in 1813. *Anchusa zeylanica* J. Jacq., Ecl. Pl. Rar.: 47. 1812, might perhaps be considered as an earlier isonym rather than homonym, as its protologue includes reference to the holotype of the to-be *A. zeylanica* Hornem.; but as Jacquin’s correlated illustration (l.c.: t. 29), the currently accepted lectotype, is an entirely different plant (*Bothriospermum zeylanicum* (J. Jacq.) Druce), treating the two names as independent is the better answer. Two specimens in C (C10008678\*, C10008679\*) declared to be types of *Anchusa zeylanica* are not from the Vahl Herbarium and belong to *Bothriospermum*. They are original material for *A. tenella* Hornem. (*B. tenellum* (Hornem.) Fisch. & C. A. Mey.), a taxonomic synonym of *B. zeylanicum*.

---

*Anchusopsis* Bisch., Del. Sem. Hort. Bot. Heidelb. Coll. 1852: 8. 1852

- IPNI: Anchusopsis Bisch. -- Del. Sem. Hort. Heidelb. (1852) 8. (IK)
- ASSESSMENT: [*Cynoglossum* L.]
- STATUS: gen. nov.
- REFERENCE: Link
- BASIS: Original type: *Anchusopsis longiflora* (DC.) Bisch.

---

*Anchusopsis longiflora* (A. DC.) Bisch., Del. Sem. Hort. Bot. Heidelb. Coll. 1852: 8. 1852

- IPNI: Anchusopsis longiflora Bisch. -- Del. Sem. Hort. Heidelb. (1852) 8. (IK)
- ASSESSMENT: [*Cynoglossum longifolium* (Leichtlin ex Beck & F. Abel) Greuter & Stier]
- STATUS: comb. nov.
- REFERENCE: Link
- BASIS: Basionym: *Omphalodes longiflora*  A. DC.; Replaced synonym: *Cynoglossum longiflorum* Royle ex Benth., non *Cynoglossum longiflorum* Lehm.

---

*Antiotrema dunnianum* (Diels) Hand.-Mazz. in Anz. Akad. Wiss. Wien 57: 240. 1920

- IPNI: Antiotrema dunnianum Hand.-Mazz. -- Anz. Akad. Wiss. Wien 1920, lvii. 240. (IK)
- ASSESSMENT: accepted
- STATUS: comb. nov.
- REFERENCE: Link
- BASIS: Basionym: *Cynoglossum dunnianum* Diels

---

*Austrocynoglossum* Popov ex R. R. Mill in Notes Roy. Bot. Gard. Edinburgh 46: 43. 1989

- IPNI: Austrocynoglossum M.Popov ex R.R.Mill -- Notes Roy. Bot. Gard. Edinburgh 46(1): 43. 1989 (IK)
- ASSESSMENT: accepted
- STATUS: nom. nov.
- REFERENCE: Link
- BASIS: Replaced synonym: *Cynoglossum* sect. *Axillaria* Riedl

---

*Austrocynoglossum latifolium* (R. Br.) R. R. Mill in Notes Roy. Bot. Gard. Edinburgh 46: 44. 1989

- IPNI: Austrocynoglossum latifolium (R.Br.) R.R.Mill -- Notes Roy. Bot. Gard. Edinburgh 46(1): 44. 1989 (IK)
- ASSESSMENT: accepted
- STATUS: comb. nov.
- REFERENCE: Link
- BASIS: Basionym: *Cynoglossum latifolium* R. Br.

---

*Bilegnum* Brand in Repert. Spec. Nov. Regni Veg. 13: 549. 1915

- IPNI: Bilegnum Brand -- Repert. Spec. Nov. Regni Veg. 13: 549. 1915 (IK)
- ASSESSMENT: [*Cynoglossum* L.]
- STATUS: gen. nov.
- REFERENCE: Link
- SYNONYMY:   
  ≡ *Rindera* sect. *Bilegnum* (Brand) Riedl 1967
    
  ≡ *Rindera* subg. *Bilegnum* (Brand) R. R. Mill 2005
- BASIS: Original type: *Bilegnum bungei* (Boiss.) Brand (≡ *Mattia bungei* Boiss.)

---

*Bilegnum bungei* (Boiss.) Brand in Repert. Spec. Nov. Regni Veg. 13: 550. 1915

- IPNI: Bilegnum bungei Brand -- Repert. Spec. Nov. Regni Veg. 13: 550. 1915 (IK)
- ASSESSMENT: *Cynoglossum bungei* (Boiss.) Greuter & Stier
- STATUS: comb. nov.
- REFERENCE: Link
- BASIS: Basionym: *Mattia bungei* Boiss.

---

*Bilegnum medium* Turrill in Bull. Misc. Inform. Kew 1929: 232. 1929

- IPNI: Bilegnum medium Turrill -- Bull. Misc. Inform. Kew 1929(7): 232. [28 Aug 1929] (IK)
- ASSESSMENT: *Cynoglossum medium* (Turrill) Greuter & Stier
- STATUS: spec. nov.
- REFERENCE: Link
- SYNONYMY:   
  ≡ *Rindera media* (Turrill) Parsa 1949
    
  ≡ *Cynoglossum medium* (Turrill) Greuter & Stier 2015
- BASIS: Originalmaterial: N. Persia: Urmia district, May 1926, Gilliat-Smith 1542 [from protologue]. Holotype: presumably at K

---

*Borago macranthera* Banks & Sol. in Russel, Nat. Hist. Aleppo, ed. 2. 2: 246. 1794

- IPNI: Borago macranthera Russell -- Aleppo, ed. II. ii. 246. (IK)
- ASSESSMENT: *Caccinia macranthera* (Banks & Sol.) Brand
- STATUS: spec. nov.
- REFERENCE: Link
- SYNONYMY:   
  ≡ *Caccinia macranthera* (Banks & Sol.) Brand 1921
- BASIS: Original material: None indicated [Syria, Aleppo area]. Original specimen (holotype?): Russell, BM (BM001014440\*)

---

*Bothriospermum marifolium* (Roxb.) DC. & A. DC. in Candolle, Prodr. 10: 116. 1846

- IPNI: Bothriospermum marifolium A.DC. -- Prodr. [A. P. de Candolle] 10: 116. 1846 [8 Apr 1846] (IK)
- ASSESSMENT: accepted
- STATUS: comb. nov.
- REFERENCE: Link
- BASIS: Basionym: *Cynoglossum marifolium* Roxb.

---

*Bothriospermum zeylanicum* (J. Jacq.) Druce in Rep. Bot. Soc. Exch. Club Brit. Isles 4: 610. 1917

- IPNI: Bothriospermum zeylanicum Druce -- Rep. Bot. Exch. Cl. Brit. Isles 1916, 610 (1917). (IK)
- ASSESSMENT: accepted
- STATUS: comb. nov.
- REFERENCE: Link
- BASIS: Basionym: *Anchusa zeylanica* J. Jacq.
- COMMENTS: Considered the correct name of two heterotypic synonyms: *Cynoglossum diffusum* Roxb. ex Lehm. and *Cynoglossum prostratum* Buch.-Ham. ex D. Don.

---

*Brandella erythraea* (Brand) R. R. Mill in Notes Roy. Bot. Gard. Edinburgh 43: 478. 1986

- IPNI: Brandella erythraea (Brand) R.R.Mill -- Notes Roy. Bot. Gard. Edinburgh 43(3): 478. 1986 (IK)
- ASSESSMENT: Non *Cynoglossum* s.l.; *Microparacaryum* sp. [combination not yet published]
- STATUS: comb. nov.
- REFERENCE: Link
- BASIS: Basionym: *Adelocaryum erythraeum* Brand

---

*Caccinia macranthera* (Banks & Sol.) Brand in Engler, Pflanzenr. 78 (IV.252): 90. 1921

- IPNI: Caccinia macranthera Brand -- Pflanzenr. (Engler) Borrag. Cynogloss. 90. 1921 (IK)
- ASSESSMENT: accepted
- STATUS: comb. nov.
- REFERENCE: Link
- BASIS: Basionym: *Borago macranthera* Banks & Sol.
- COMMENTS: Considered the correct name of the heterotypic synonym *Cynoglossum longiflorum* Lehm..

---

*Cerinthopsis* Kotschy ex Paine in Palestine Explor. Soc. Statem. 3: 114. 1875

- IPNI: Cerinthopsis Kotschy ex Paine -- Palestine Explor. Soc. Third Statem. 114. 1875 [Jan 1875] (IK)
- ASSESSMENT: [*Cynoglossum* L.]
- STATUS: gen. nov.
- REFERENCE: Link
- BASIS: Type [probably first designated on ING card, date 1996-02-09]: *Cerinthopsis foliosa* Paine
- COMMENTS: *Cerinthopsis* originally comprised two species, one described at length, the second briefly diagnosed; but the only descriptive statement referring to the genus reads: “By the addition of this species *Cerinthopsis* is confirmed as a good genus, differing from *Solenanthus* chiefly in the stamens.” The stamens are not explicitly mentioned in the description of *Cerinthopsis foliosa*, but the anthers are (“antheris linearibus fauci corollae insertis subsessilibus lobis aequilongis”), so that by a generous interpretation of the rules one can accept the generic name (and with it the two binomials) as validly published.

---

*Cerinthopsis* Kotschy ex Benth. & Hook. f., Gen. Pl. 2: 849. 1876

- IPNI: Cerinthopsis Kotschy ex Benth. & Hook.f. -- Gen. Pl. [Bentham & Hooker f.] 2(2): 849. 1876 [May 1876] (IK)
- STATUS: nom. inval. [pro syn. sub *Solenanthus*]
- REFERENCE: Link

---

*Cerinthopsis foliosa* Paine in Palestine Explor. Soc. Statem. 3: 114. 1875

- IPNI: Cerinthopsis foliosa Paine -- Palestine Explor. Soc. Third Statem. 114. 1875 [Jan 1875] (IK)
- ASSESSMENT: *Cynoglossum foliosum* (Paine) Greuter & Burdet
- STATUS: spec. nov.
- REFERENCE: Link
- SYNONYMY:   
  ≡ *Trachelanthus foliosus* (Paine) Tristram 1884
- BASIS: Original material: Gathered in el-Wâdî el-Harâmîyeh at the edge of forests along the open valley-ground, April 10th, 1873 [from protologue]

---

*Cerinthopsis kurdica* Kotschy, in schedis impr. Iter cilicio-kurdicum 1859, Nr. 463

- IPNI: absent (2014-11-05)
- STATUS: nom. inval. [nom. nud.]
- COMMENTS: For additional information see *Cerinthopsis kurdica* Kotschy ex Paine.

---

*Cerinthopsis kurdica* of Kotschy [Boiss. Fl. Orient. 4: 271. 1875]

- IPNI: Cerinthopsis kurdica Kotschy ex Boiss. -- Fl. Orient. [Boissier] 4(1): 271. 1875 [Sep-Oct 1875] (IK)
- STATUS: [isonym]
- REFERENCE: Link

---

*Cerinthopsis kurdica* Kotschy ex Paine in Palestine Explor. Soc. Statem. 3: 115. 1875

- IPNI: Cerinthopsis kurdica Kotschy ex Paine -- Palestine Explor. Soc. Third Statem. 115. 1875 [Jan 1875] in obs. (IK)
- ASSESSMENT: [*Cynoglossum cerinthoides* (Boiss.) Greuter & Burdet]
- STATUS: spec. nov.
- REFERENCE: Link
- SYNONYMY:   
  ≡ *Trachelanthus kurdicus* (Kotschy ex Paine) Boiss. 1875 [Sep-Oct]
    
  ≡ *Solenanthus kurdicus* (Kotschy ex Paine) Gürke 1893
    
  ≡ *Trachelanthus cerinthoides* var. *kurdicus* (Kotschy ex Paine) Post 1896
    
  ≡ *Lindelofia kurdica* (Kotschy ex Paine) Brand 1921
- BASIS: Original material: Iter cilicico-kurdicum, No. 463 [from protologue]. Text of printed label: Plantae ex schistosis in alpibus prope Musch lectae: In angustiis vallis Teng alt. 6500'. Die 9. Sept. [from sheet in B] \*

---

*Craniospermum subvillosum* Lehm., Pl. Asperif. Nucif.: 337. 1818

- IPNI: Craniospermum subvillosum Lehm. -- Pl. Asperif. Nucif. 2: 337. 1818 [Sep-Oct 1818] (IK)
- ASSESSMENT: accepted
- STATUS: spec. nov.
- REFERENCE: Link
- BASIS: Original material: locis glareosis ad lacum Baical in Sibiria (v. s.) [from protologue]. Original specimens: MEL?; S (S12-8154\*)
- COMMENTS: Considered the correct name of the heterotypic synonym *Cynoglossum baicalense* Pall. ex Roem. & Schult. (*Solenanthus baicalensis* (Pall. ex Roem. & Schult.) DC.).

---

*Cynoglossopsis latifolia*  (Hochst. ex A. Rich.) Brand in Engler, Pflanzenr. 97 (IV.252): 22. 1931

- IPNI: Cynoglossopsis latifolia (Hochst. ex A.Rich.) Brand -- Pflanzenr. (Engler) Borrag.-Borraginoid.-Cryptanth. 22 (1931). (IK)
- ASSESSMENT: accepted
- STATUS: comb. nov.
- REFERENCE: Link
- BASIS: Basionym: *Echinospermum latifolium*  Hochst. ex A. Rich.

---

*Cynoglossum* L., Sp. Pl.: 134. 1753

- IPNI: Cynoglossum L. – Sp. Pl. 1: 134. 1753 [1 May 1753] (IK)
- ASSESSMENT: accepted
- STATUS: gen. nov.
- REFERENCE: Link
- BASIS: Type (Britton & Brown, Ill. Fl. N. U.S., ed. 2, 3: 75. 1913, confirmed by Hitchcock in Prop. Brit. Bot.: 127. 1929): *Cynoglossum officinale* L.

---

*Cynoglossum* sect. *Axillaria* Riedl in Oesterr. Bot. Z. 109: 393. 1962

- IPNI: absent (2014-11-05)
- ASSESSMENT: *Austrocynoglossum* Popov ex R. R. Mill
- STATUS: sect. nov.
- REFERENCE: Link
- SYNONYMY:   
  ≡ *Austrocynoglossum* Popov ex R. R. Mill 1989
- BASIS: Original type: *Cynoglossum latifolium* R. Br.

---

*Cynoglossum* sect. *Bracteata Riedl* in Oesterr. Bot. Z. 109: 391. 1962

- IPNI: absent (2014-11-05)
- ASSESSMENT: [*Cynoglossum* L.]
- STATUS: sect. nov.
- REFERENCE: Link
- BASIS: Original type: *Cynoglossum cheirifolium* L.

---

*Cynoglossum* sect. *Eleutherostylum* Brand in Engler, Pflanzenr. 78 (IV.252): 115,140. 1921

- IPNI: Cynoglossum sect. Eleutherostylum Brand – Engler's Das Pflanzenreich Heft 78 1921 (APNI)
- ASSESSMENT: Not *Cynoglossum* s. l.; genus uncertain
- STATUS: sect. nov.
- REFERENCE: Link
- SYNONYMY:   
  ≡ *Cynoglossum* subg. *Eleutherostylum* (Brand) Riedl 1962
- BASIS: Type (Riedl in Oesterr. Bot. Z. 109: 393. 1962): *Cynoglossum grande* Dougl. ex Lehm.

---

*Cynoglossum* subg. *Eleutherostylum* (Brand) Riedl in Oesterr. Bot. Z. 109: 393. 1962

- IPNI: absent (2014-11-05)
- ASSESSMENT: Not *Cynoglossum* s. l.; genus uncertain
- STATUS: stat. nov. [section to subgenus]
- REFERENCE: Link
- BASIS: Basionym: *Cynoglossum* sect. *Eleutherostylum* Brand

---

*Cynoglossum* sect. *Eucynoglossum* of Brand in Engler, Pflanzenr. 78 (IV.252): 115. 1921

- IPNI: Cynoglossum sect. Eucynoglossum Brand – Engler's Das Pflanzenreich Heft 78 1921 (APNI)
- STATUS: nom. inval. [ICN Art. 21.3 & 22.2]
- REFERENCE: Link

---

*Cynoglossum* sect. *Foliata* Riedl in Oesterr. Bot. Z. 109: 392. 1962

- IPNI: absent (2014-11-05)
- ASSESSMENT: [*Cynoglossum* L.]
- STATUS: sect. nov.
- REFERENCE: Link
- BASIS: Original type: *Cynoglossum divaricatum* Steph. ex Lehm.

---

*Cynoglossum* [unranked] *Lappula* (Moench) Wallr., Sched. Crit.: 76. 1822

- IPNI: absent (2014-11-05)
- ASSESSMENT: *Lappula* Moench
- STATUS: stat. nov. [genus to subdivison of genus]
- REFERENCE: Link
- BASIS: not designatedBasionym: *Lappula* Moench
- COMMENTS: Published without stated rank as one of four “subdivisiones” (a term here used colloquially, not as rank designator). No reference is made to the basionym, but the type of both names is the same (see ICN Art. 22.6) and the subdivisional epithet coincides with the generic name, indicating Wallroth’s intent (ICN Art. 41.4).

---

*Cynoglossum* sect. *Lindelofia* of Lehmann [Regel & Smirnov in Trudy Imp. S.-Peterburgsk. Bot. Sada 5: 623. 1877]

- IPNI: absent (2014-11-05)
- STATUS: no name [ICN Art. 35.2]
- REFERENCE: Link
- COMMENTS: Regel & Smirn. described an unnamed “sectio II” with *Lindelofia* Lehm. as synonym.

---

*Cynoglossum* subg. *Mattiaria* (Coss.) Greuter in Willdenowia 11: 33. 1981

- IPNI: Cynoglossum subgen. Mattiaria (Coss.) Greuter – Willdenowia 11(1): 33. 1981 (IK)
- ASSESSMENT: [*Cynoglossum* L.]
- STATUS: comb. nov.
- REFERENCE: Link
- BASIS: Basionym: *Mattia* subg. *Mattiaria* Coss.

---

*Cynoglossum* [unranked] *Omphalium* Wallr., Sched. Crit.: 77. 1822

- IPNI: absent (2014-11-05)
- ASSESSMENT: *Omphalodes* Mill.
- STATUS: taxon nov.
- REFERENCE: Link
- BASIS: Original type (ICN Art. 22.6): *Cynoglossum omphaloides* L. (*Omphalodes verna* Moench, nom. illeg., nom. cons. prop.)
- COMMENTS: Published without stated rank as one of four “subdivisiones” (a term here used colloquially, not as rank designator) of *Cynoglossum*. Validated by its own description. Might be regarded as a replacement name (nom. nov.) for the homotypic *Omphalodes* Mill. (1754), except for the fact that the latter was not then typified as yet. Raised to generic rank as *Omphalium* (Wallr.) Roth (1827).

---

*Cynoglossum* subg. *Papilligera* Riedl in Oesterr. Bot. Z. 109: 392. 1962 *(‘Papilligerum’)*

- IPNI: absent (2014-11-05)
- ASSESSMENT: [*Cynoglossum* L.]
- STATUS: subg. nov.
- REFERENCE: Link
- BASIS: Original type: *Cynoglossum viridiflorum* Pall.

---

*Cynoglossum* sect. *Paracaryopsis* Riedl in Oesterr. Bot. Z. 109: 393. 1962

- IPNI: absent (2014-11-05)
- ASSESSMENT: [*Cynoglossum* L.]
- STATUS: sect. nov.
- REFERENCE: Link
- SYNONYMY:   
  ≡ *Paracaryopsis* (Riedl) R. R. Mill 1991
- BASIS: Original type: *Cynoglossum coelestinum* Lindl.

---

*Cynoglossum* sect. *Paracaryum* (A. DC.) Kern. in Ber. Naturwiss.-Med. Vereins Innsbruck 1: 109. 1870

- IPNI: absent (2014-11-05)
- ASSESSMENT: [*Cynoglossum* L.]
- STATUS: comb. nov.
- REFERENCE: Link
- BASIS: Basionym [implicit]: *Omphalodes* sect. *Paracaryum* A. DC.
- COMMENTS: Kerner's opinion to reduce *Paracaryum* to *Cynoglossum* sect. *Paracaryum*, but apparently no formal combination. Sentence in subjunctive.

---

*Cynoglossum* subg. *Paracynoglossum* (Popov) Riedl in Oesterr. Bot. Z. 109: 392. 1962

- IPNI: absent (2014-11-05)
- ASSESSMENT: [*Cynoglossum* L.]
- STATUS: stat. nov. [genus to subgenus]
- REFERENCE: Link
- BASIS: Basionym: *Paracynoglossum* Popov

---

*Cynoglossum* [unranked] *Tetraspis* Wallr., Sched. Crit.: 77. 1822

- IPNI: absent (2014-11-05)
- ASSESSMENT: [*Cynoglossum* L.]
- STATUS: nom. nov.
- REFERENCE: Link
- BASIS: Replaced synonym: *Rindera* Pall.
- COMMENTS: Published without stated rank as one of four “subdivisiones” (a term here used colloquially, not as rank designator). Best regarded as a replacement name (nom. nov.) for the homotypic *Rindera* Pall., the types being the same (see ICN Art. 22.6).

---

*Cynoglossum abyssinicum* of Hochstetter [Engler in Abh. Königl. Akad. Wiss. Berlin 1891: 353. 1892]

- IPNI: Cynoglossum abyssinicum Hochst. ex Engl. -- Abh. Preuss. Akad. Wiss. 1891. ii. (1892) 353. (IK)
- STATUS: nom. inval. [nom. nud.]
- REFERENCE: Link

---

*Cynoglossum aequinoctiale* T. C. E. Fr. in Notizbl. Bot. Gart. Berlin-Dahlem 8: 416. 1923

- IPNI: Cynoglossum aequinoctiale T.C.E.Fr. -- Notizbl. Bot. Gart. Berlin-Dahlem 8: 416. 1923 (IK)
- ASSESSMENT: accepted
- STATUS: spec. nov.
- REFERENCE: Link
- BASIS: Original material: Specimen originale: Rob. E. et Th. C. E. Fries n. 303 in museo botanico Upsaliensi [fig. 6] … Britisch Ost-Afrika: W.-Lenya Forest Station auf der *Themeda*-Steppe ca 2300 m. ü. d. M. Mit Frucht am 25. Dezember 1921 [+ 1 paratype] [from protologue]— [Fig. 6.]. Holotype: UPS \*; isotypes: BR (BR0000008865335\*), S (S11-34494\*)

---

*Cynoglossum afrocaeruleum* Riedl in Linzer Biol. Beitr. 17: 320. 1985

- IPNI: Cynoglossum afrocaeruleum (R.R.Mill) Riedl -- Linzer Biol. Beitr. 17(2): 320 (1985). (IK)
- ASSESSMENT: Not *Cynoglossum* s. l.; genus uncertain [*“Cynoglossum” coeruleum* Hochst. ex A. DC.]
- STATUS: nom. illeg. [superfl.]
- REFERENCE: Link
- BASIS: *Cynoglossum coeruleum* Hochst. ex A. DC.
- COMMENTS: Published on the erroneous assumption that *Cynoglossum coeruleum* would be a later parahomonym of the [not validly published] *Cynoglossum caeruleum* of Buchanan-Hamilton.

---

*Cynoglossum alaicum* (Lazkov) Greuter & Stier in Biodivers. Data J. [hoc loco]. 2015

- ASSESSMENT: accepted
- STATUS: comb. nov.
- BASIS: Basionym: *Rindera alaica* Lazkov

---

*Cynoglossum alatum* Molina, Sag. Stor. Nat. Chili, ed. 2.: 280. 1810

- IPNI: absent (2014-11-05)
- ASSESSMENT: Not *Cynoglossum* s. l.; genus uncertain [*“Cynoglossum” limense* Willd.]
- STATUS: nom. illeg. [superfl.]
- REFERENCE: Link
- BASIS: *Cynoglossum decurrens* Ruiz & Pav.

---

*Cynoglossum albanicum* Degen & Bald. in Nuovo Giorn. Bot. Ital. 6: 337. 1899

- IPNI: Cynoglossum albanicum Degen & Bald. -- Nuovo Giorn. Bot. Ital. vi. 337. (IK)
- ASSESSMENT: accepted
- STATUS: spec. nov.
- REFERENCE: Link
- SYNONYMY:   
  ≡ *Solenanthus albanicus* (Degen & Bald.) Degen & Bald. 1903
- BASIS: Original material: In aridis alpestribus ad fontem Bocikopoulon distr. Pogoni, ubi fructiferam die 8 Julii detexi! Num. collect. 188. [from protologue]. Hab. In Epiro boreali. In aridis alpestribus ad fontem Bocikipoulon district. Pogoni die 8 Juli 1896 fructiferum detexit amiciss. Dr. Ant. Baldacci (exsicc, ex itinere albanico – [epirotico] quarto 1896 No. 188.) [Degen in Magyar Bot. Lapok 2: 315. 1903]. Syntypes : BM (BM000752673), FI?, WU (WU0043275) \*

---

*Cynoglossum albidum* (Wettst.) Greuter & Burdet in Willdenowia 11: 34. 1981

- IPNI: Cynoglossum albidum (Wettst.) Greuter & Burdet -- Willdenowia 11(1): 34. 1981 (IK)
- ASSESSMENT: accepted
- STATUS: comb. nov.
- REFERENCE: Link
- BASIS: Basionym: *Mattia albida*  Wettst.

---

*Cynoglossum albiflorum* (Czukav. & Meling) Greuter & Stier in Biodivers. Data J. [hoc loco]. 2015

- ASSESSMENT: accepted
- STATUS: comb. nov.
- BASIS: Basionym: *Solenanthus albiflorus* Czukav. & Meling

---

*Cynoglossum album* of Gueldenstaedt, Reis. Russland 1: 79. 1787

- IPNI: absent (2014-11-05)
- STATUS: nom. inval. [nom. nud.]
- REFERENCE: Link

---

*Cynoglossum album* of Gueldenstaedt [Ledebour, Fl. Ross. 3: 166. 1847]

- IPNI: Cynoglossum album Gueldenst. ex Ledeb. -- Fl. Ross. (Ledeb.) 3(1,8): 166. 1847 [Oct 1847] (IK)
- STATUS: nom. inval. [pro syn. sub *Cynoglossum officinale* var. *bicolor*]
- REFERENCE: Link

---

*Cynoglossum alpestre* Ohwi in Acta Phytotax. Geobot. 2: 150. 1933

- IPNI: Cynoglossum alpestre Ohwi -- Acta Phytotax. Geobot. 1933, ii. 150. (IK)
- ASSESSMENT: accepted
- STATUS: spec. nov.
- REFERENCE: Link
- BASIS: Original material: Formosa: Bunakkei in m. Nankotaisan in Taichushu (J. Ohwi n. 4076) [from protologue]. Type probably in KYO; isotype in TNS (TNS-VS-43024; see Ebihara & Miyashita in Bull. Natl. Mus. Nat. Sci., Tokyo, B 34: 107. 2008)

---

*Cynoglossum alpinum* (Brand) Riedl in Linzer Biol. Beitr. 17: 317. 1985

- IPNI: Cynoglossum alpinum (Brand) Riedl -- Linzer Biol. Beitr. 17(2): 317 (1985), as 'sp. nov.'. (IK)
- ASSESSMENT: accepted
- STATUS: comb. & stat. nov. [variety to species]
- REFERENCE: Link
- BASIS: Basionym: *Cynoglossum montanum* var. *alpinum*  Brand

---

*Cynoglossum alpinum* of Brand [B. L. Burtt in Notes Roy. Bot. Gard. Edinburgh 43: 345. 1986]

- IPNI: Cynoglossum alpinum (Brand) B.L.Burtt -- Notes Roy. Bot. Gard. Edinburgh 43(3): 345. 1986 (IK)
- STATUS: [isonym]
- REFERENCE: Link

---

*Cynoglossum alticola* Hilliard & B. L. Burtt in Notes Roy. Bot. Gard. Edinburgh 43: 346. 1986

- IPNI: Cynoglossum alticola Hilliard & B.L.Burtt -- Notes Roy. Bot. Gard. Edinburgh 43(3): 346. 1986 (IK)
- ASSESSMENT: accepted
- STATUS: spec. nov.
- REFERENCE: Link
- BASIS: Holotype: E Cape, Barkly East distr., 3027 DB, Ben McDhui, 8600 ft, 5 ii 1983, Hilliard & Burtt 16468, cult. at RBG Edinb. Under 831306 (E holo.) + 2 Paratypes [from protologue]. Holotype: E (E00193310), isotype: NU (NU0016516-0) \*

---

*Cynoglossum amabile* Stapf & J. R. Drumm. in Bull. Misc. Inform. Kew 1906: 202. 1906

- IPNI: Cynoglossum amabile Stapf & J.R.Drumm. -- Bull. Misc. Inform. Kew 1906(6): 202. (IK)
- ASSESSMENT: accepted
- STATUS: spec. nov.
- REFERENCE: Link
- BASIS: Original material: China. Yunnan, Maengtsze, Hancock 133; Szemao, 1350 m., Henry, 9365; Szecuan, Tatsienlu, Soulié, 861; 2700–4050 m., Pratt, 887; without precise locality, cultivated and communicated by Max Leichtlin [from protologue]. Lectotype (Verdcourt in Polhill, Fl. Trop. E. Africa, Boragin.: 104. 1991): Hancock 133 (K); isotypes: BM, C, GH, HK, LE, NY, OXF, P, US

---

*Cynoglossum amabile* f. *leucanthum* X. D. Dong in Bull. Bot. Res., Harbin 17: 141. 1997

- IPNI: Cynoglossum amabile Stapf & J.R.Drumm. f. leucanthum X.D.Dong -- Bull. Bot. Res., Harbin 17(2): 141. 1997 (IK)
- ASSESSMENT: [*Cynoglossum amabile* Stapf & J. R. Drumm.]
- STATUS: f. nov.
- REFERENCE: Link
- BASIS: Holotype: Yunnan: Dali, Cangshan, alt. 2200m, 15 Jun. 1994, Dong Xiao–dong, No. 0079 (Typus in Herb. Department of Biology, Dali Teacher's college, Yunnan) [from protologue]

---

*Cynoglossum amabile* var. *parviflorum* Turrill in Bot. Mag. 177: ad tab. 82. 1949

- IPNI: absent (2014-11-05)
- ASSESSMENT: [*Cynoglossum amabile* Stapf & J. R. Drumm.]
- STATUS: var. nov.
- REFERENCE: Link
- BASIS: Original material: Cultivated at Kew … grown as an annual … sown in April 1947 [from protologue]. Original specimens: A (A00096678\*), E (E00288407\*, E00288408\*), G (G00177266\*), K, US (US00110903\*, US01013752\*). Lectotype (to be designated elsewhere by König et al.): Herbarium experimental grounds, K. 2492, seed from Ceylon, 15 July 1947, anon. (K)

---

*Cynoglossum amabile* var. *pauciglochidiatum* Y. L. Liu in Acta Phytotax. Sin. 19: 520. 1981

- IPNI: Cynoglossum amabile Stapf & J.R.Drumm. var. pauciglochidiatum Y.L.Liu -- Acta Phytotax. Sin. 19(4): 520 (1981). (IK)
- ASSESSMENT: [*Cynoglossum amabile* Stapf & J. R. Drumm.]
- STATUS: var. nov.
- REFERENCE: Link
- BASIS: Holotype: loco dicto [Lijang], alt. 2700 m, IX. 1958, W. T. Wang 186 (Typus in Herb. Kumming. [KUN] Inst. Bot. Acad. Sin. conserv.) [from protologue]

---

*Cynoglossum amabile* f. *rubrum* X. D. Dong in Bull. Bot. Res., Harbin 17: 141. 1997 *(‘ruberum’)*

- IPNI: Cynoglossum amabile Stapf & J.R.Drumm. f. ruberum X.D.Dong -- Bull. Bot. Res., Harbin 17(2): 141. 1997 (IK)
- ASSESSMENT: [*Cynoglossum amabile* Stapf & J. R. Drumm.]
- STATUS: f. nov.
- REFERENCE: Link
- BASIS: Holotype gathering: Yunnan: Dali, Cangshan, alt. 2100m, 20 Jun. 1994, Dong Xiao–dong, No.[0089], 0090 (Typus in Herb. Department of Biology, Dali Teacher's college, Yunnan) [from protologue]
- COMMENTS: Two specimens are designated as holotype, but as they are obviously part of the same gathering (same taxon, same locality, same date, same collector) and are kept in the same institution, the name is validly published (ICN Art. 40.2 & 6–7). The diagnosis refers to “floribus rubris” (not “ruberis”), so that the original spelling of the epithet is considered a correctable (orthographical or typographical) error.

---

*Cynoglossum amani* (Rech. f.) Greuter & Burdet in Willdenowia 11: 34. 1981

- IPNI: Cynoglossum amani (Rech.f.) Greuter & Burdet -- Willdenowia 11(1): 34. 1981 (IK)
- ASSESSMENT: accepted
- STATUS: comb. nov.
- REFERENCE: Link
- BASIS: Basionym: *Mattiastrum amani* Rech. f.

---

*Cynoglossum amplexicaule* Lam., Tabl. Encycl. 1: 399. 1792

- IPNI: Cynoglossum amplexicaule Lam. -- Tabl. Encycl. i. 399. (IK)
- ASSESSMENT: [*Cynoglossum creticum* Mill.]
- STATUS: spec. nov.
- REFERENCE: Link
- BASIS: Original material: [Cult. in Paris?] Ex Oriente? ♂ Cynogl. creticum. 2. Clus. an C. pictum. Hort. Kew [from protologue]. Type: P-LAM?

---

*Cynoglossum amplexicaule* Michx., Fl. Bor.-Amer. 1: 132. 1803

- IPNI: Cynoglossum amplexicaule Michx. -- Fl. Bor.-Amer. (Michaux) 1: 132. 1803 [19 Mar 1803] (IK)
- ASSESSMENT: Not *Cynoglossum* s. l.; genus uncertain [*“Cynoglossum” virginianum* L.]
- STATUS: nom. illeg. [homonym]
- REFERENCE: Link
- BASIS: Original material: In montibus Alléghanis [from protologue]

---

*Cynoglossum amplifolium* Hochst. ex A. DC. in Candolle, Prodr. 10: 149. 1846

- IPNI: Cynoglossum amplifolium Hochst. ex DC. -- Prodr. [A. P. de Candolle] 10: 149. 1846 [8 Apr 1846] (IK)
- ASSESSMENT: Not *Cynoglossum* s. l.; genus uncertain (species accepted)
- STATUS: spec. nov.
- REFERENCE: Link
- BASIS: Original material: (Hochst.! pl. Schimp. abyss. sect. 2. n. 564) in mediâ regione montium Abyssiniae [from protologue]. Holotype: Ethiopia, between Endschetcap and Schoata, Schimper 564 (G-DC); isotypes: BM, BR\*, HBG\*, K\*, L\*, M\*, MPU\*, S\*, UPS, W\*

---

*Cynoglossum amplifolium* f. *macrocarpum* Brand in Engler, Pflanzenr. 78 (IV.252): 141. 1921

- IPNI: absent (2014-11-05)
- ASSESSMENT: Not *Cynoglossum* s. l.; genus uncertain [*“Cynoglossum” amplifolium* Hochst. ex A. DC.]
- STATUS: nom. nov.
- REFERENCE: Link
- BASIS: Replaced synonym: *Cynoglossum lancifolium* Hook. f.
- COMMENTS: For additional information see *Cynoglossum lancifolium* Hook. f..

---

*Cynoglossum amplifolium* var. *subalpinum* (T. C. E. Fr.) Verdc. in Polhill, Fl. Trop. E. Africa, Boragin.: 106. 1991

- IPNI: Cynoglossum amplifolium Hochst. ex DC. var. subalpinum (T.C.E.Fr.) Verdc. -- Fl. Trop. E. Africa, Boragin. 106. 1991 (IK)
- ASSESSMENT: Not *Cynoglossum* s. l.; genus uncertain [*“Cynoglossum” amplifolium* Hochst. ex A. DC.]
- STATUS: comb. & stat. nov. [species to variety]
- REFERENCE: Link
- BASIS: Basionym: *Cynoglossum subalpinum* T. C. E. Fr.

---

*Cynoglossum anchusoides* Lindl. in Edwards’s Bot. Reg. 28: t. 14. 1842

- IPNI: Cynoglossum anchusoides Lindl. -- Edwards's Bot. Reg. 28: t. 14. 1842 (IK)
- ASSESSMENT: accepted
- STATUS: spec. nov.
- REFERENCE: Link
- SYNONYMY:   
  ≡ *Lindelofia anchusoides* (Lindl.) Lehm. 1850
    
  ≡ *Adelocaryum anchusoides* (Lindl.) Brand 1915
- BASIS: Original material: Raised from seeds received from the East India Company, said to have been collected either in Cashmere or Tibet [from protologue]. Presumed type: Herb. East India Company, without collector, s.n. (K) [Kazmi in J. Arnold Arb. 52: 335. 1971]

---

*Cynoglossum anchusoides* subsp. *asperum* (Rech. f.) Greuter & Stier in Biodivers. Data J. [hoc loco]. 2015

- ASSESSMENT: accepted
- STATUS: comb. nov.
- BASIS: Basionym: *Lindelofia aspera* Rech. f.

---

*Cynoglossum ancyritanum* (Boiss.) Greuter & Burdet in Willdenowia 11: 34. 1981

- IPNI: Cynoglossum ancyritanum (Boiss.) Greuter & Burdet -- Willdenowia 11(1): 34. 1981 (IK)
- ASSESSMENT: accepted
- STATUS: comb. nov.
- REFERENCE: Link
- BASIS: Basionym: *Paracaryum ancyritanum* Boiss.

---

*Cynoglossum andicola* K. Krause in Bot. Jahrb. Syst. 37: 635. 1906 *(‘andicolum’)*

- IPNI: Cynoglossum andicola K.Krause -- Bot. Jahrb. Syst. 37(5): 635. 1906 [30 Oct 1906] (IK)
- ASSESSMENT: *Hackelia andicola* (K. Krause) Brand
- STATUS: spec. nov.
- REFERENCE: Link
- SYNONYMY:   
  ≡ *Hackelia andicola* (K. Krause) Brand 1931
- BASIS: Peru: Inter Cajamarca et Hualgayoc, in saxosis, 4100–4200 m s.m. (Weberbauer n. 4230 –- fl. Mense Junio) [from protologue]
- COMMENTS: Correction of the epithet’s original spelling mandated by ICN Art. 23.5.

---

*Cynoglossum angustifolium* Willd., Sp. Pl. 1: 763. 1798

- IPNI: Cynoglossum angustifolium Willd. -- Sp. Pl., ed. 4 [Willdenow] 1(2): 763. 1798 [Jul 1798] (IK)
- ASSESSMENT: [*Cynoglossum racemosum* Schreb.]
- STATUS: spec. nov.
- REFERENCE: Link
- SYNONYMY:   
  ≡ *Mattia angustifolia* (Willd.) G. Don 1837-1838
    
  ≡ *Paracaryum angustifolium* (Willd.) Boiss. 1849
- BASIS: Original material: in Armenia (v. s.) [from protologue]. Holotype: B-W (B-W03337\*)
- COMMENTS: Tournefort’s phrase (1707) “*Cynoglossum orientale minus, flore campanulato caeruleo*” is the basis of three *Cynoglossum* names: *Cynoglossum racemosum* Schreb. (1767), *Cynoglossum emarginatum* Lam. (1791) and *Cynoglossum angustifolium* Willd. (1789), all with different type specimens. Therefore we treat them as three heterotypic, legitimate names.

---

*Cynoglossum angustifolium* of gardeners, ascribed to Steudel, Nomencl. Bot.: 251. 1821

- IPNI: Cynoglossum angustifolium Hort. ex Steud. -- Nomencl. Bot. [Steudel] 251. 1821 (IK)
- STATUS: nom. inval. [pro syn. sub *Cynoglossum officinale*]
- REFERENCE: Link

---

*Cynoglossum angustifolium* of Aucher-Eloy [A. DC. in Candolle, Prodr. 10: 170. 1846]

- IPNI: Cynoglossum angustifolium Aucher ex A.DC. -- Prodr. [A. P. de Candolle] 10: 170. 1846 [8 Apr 1846] (IK)
- STATUS: nom. inval. [pro syn. sub *Mattia leptophylla*]
- REFERENCE: Link

---

*Cynoglossum apenninum* L., Sp. Pl.: 134. 1753

- IPNI: Cynoglossum apenninum L. -- Sp. Pl. 1: 134. 1753 [1 May 1753] (IK)
- ASSESSMENT: accepted
- STATUS: spec. nov.
- REFERENCE: Link
- SYNONYMY:   
  ≡ *Solenanthus apenninus* (L.) Hohen. 1838
- BASIS: Original material: in alpibus Apenninis, Campoclarensibus, umbrosis [from protologue]. Lectotype (Selvi in Taxon 53: 801. 2004): illustration ”Cynoglossa mont. maxima“ in Colonna, Ekphr.: t. 170. 1606

---

*Cynoglossum apenninum* of Gouan, Fl. Monsp.: 20. 1765

- IPNI: Cynoglossum apenninum Gouan -- Fl. Monsp. (Gouan) 20. 1764 [Dec 1764] (IK)
- STATUS: [isonym]
- REFERENCE: Link

---

*Cynoglossum apenninum* of Roth [A. DC. in Candolle, Prodr. 10: 147. 1846]

- IPNI: Cynoglossum apenninum Roth ex A.DC. -- Prodr. [A. P. de Candolle] 10: 147. 1846 [8 Apr 1846] (IK)
- STATUS: nom. inval. [pro syn. sub *Cynoglossum montanum*]
- REFERENCE: Link

---

*Cynoglossum argaeum* Greuter & Burdet in Willdenowia 11: 34. 1981

- IPNI: Cynoglossum argaeum Greuter & Burdet -- Willdenowia 11(1): 34, nom. nov. 1981 (IK)
- ASSESSMENT: accepted
- STATUS: nom. nov.
- REFERENCE: Link
- BASIS: Replaced synonym: *Paracaryum calycinum* Boiss. & Balansa, non *Cynoglossum calycinum* C. A. Mey.

---

*Cynoglossum argenteum* Lam., Fl. Franç. 2: 277. 1779

- IPNI: Cynoglossum argenteum Lam. -- Fl. Franç. (Lamarck) 2: 277. 1779 [1778 publ. after 21 Mar 1779] (IK)
- ASSESSMENT: *Cynoglossum cheirifolium* L.
- STATUS: nom. illeg. [superfl.]
- REFERENCE: Link
- BASIS: Replaced synonym: *Cynoglossum cheirifolium* L.

---

*Cynoglossum armenum* of Gundelsheimer [K. Koch in Linnaea 22: 647. 1849]

- IPNI: absent (2014-11-05)
- STATUS: nom. inval. [pro syn. sub *Mattia punctata*]
- REFERENCE: Link

---

*Cynoglossum artvinense* (R. R. Mill) Greuter & Burdet in Willdenowia 11: 34. 1981

- IPNI: Cynoglossum artvinense (R.R.Mill) Greuter & Burdet -- Willdenowia 11(1): 34. 1981 (IK)
- ASSESSMENT: accepted
- STATUS: comb. nov.
- REFERENCE: Link
- BASIS: Basionym: *Paracaryum artvinense* R. R. Mill

---

*Cynoglossum arundanum* Coss., Notes Pl. Crit.: 41. 1849

- IPNI: Cynoglossum arundanum Coss. -- Notes Pl. Crit. 41. (IK)
- ASSESSMENT: [*Cynoglossum cheirifolium* subsp. *heterocarpum* (Kunze) Font Quer]
- STATUS: spec. nov.
- REFERENCE: Link
- SYNONYMY:   
  ≡ *Cynoglossum cheirifolium* var. *arundanum* (Coss.) Maire 1936
    
  ≡ *Pardoglossum cheirifolium* var. *arundanum* (Coss.) Mathez 1975
- BASIS: Original material: Fructiferam legit 4a die Julii E. Bourgeau. In regione montana regni Granatensis, Sierra de las Nieves prope Ronda (E. Bourgeau, 1849) [from protologue]. Syntypes: P (P00616503, P00616504)\*

---

*Cynoglossum arundanum* var. *mariolense* Rouy in Bull. Soc. Bot. France 29: 123. 1882.

- IPNI: absent (2014-11-05)
- ASSESSMENT: [*Cynoglossum cheirifolium* subsp. *heterocarpum* (Kunze) Font Quer]
- STATUS: var. nov.
- REFERENCE: Link
- SYNONYMY:   
  ≡ *Cynoglossum heterocarpum* var. *mariolense* (Rouy) Willk. 1893
    
  ≡ *Cynoglossum cheirifolium* subvar. *mariolense* (Rouy) Brand 1921
- BASIS: Original material: Sierra Mariola: éboulis/rocailles du cerro de Agres [from protologue]
- COMMENTS: The name already appears, without description, in Bull. Soc. Bot. France 28: 162, 168. 1881.

---

*Cynoglossum asperrimum* Nakai in Bot. Mag. (Tokyo) 37: 6. 1923

- IPNI: Cynoglossum asperrimum Nakai -- Bot. Mag. (Tokyo) 1923, xxxvii. 6. (IK)
- ASSESSMENT: accepted
- STATUS: spec. nov.
- REFERENCE: Link
- SYNONYMY:   
  ≡ *Paracynoglossum asperrimum* (Nakai) Popov 1953
- BASIS: Original material: Quelpaert: Hallasan (T. Nakai); Corea. In insula Hokitsuto (T. Nakai); Kiusiu: Wakasugiyama prov. Chikuzen (K.Nagano); Shikoku: Koyabiramura prov. Awa (J.Nikai n. 1331); Hondo: Nikko prov. Shimotsuke (J. Matsumura). Aidzu prov. Iwashiro (R.Yatabe et J. Matsamura). Usuitoge prov. Shinano (J. Matsamura). Sone prov. Rikuzen (Y. Yabe). ontake prov. Shinano (G. Koidzumi); Yeso: Hakodate prov. Oshima (Baehmer) [from protologue]

---

*Cynoglossum asperrimum* var. *tosaense* (Nakai) H. Hara, Enum. Spermatophytarum Japon. 1: 172. 1948

- IPNI: absent (2014-11-05)
- ASSESSMENT: [*Cynoglossum asperrimum* Nakai]
- STATUS: comb. & stat. nov. [species to variety]
- REFERENCE: Link
- BASIS: Basionym: *Cynoglossum tosaense* Nakai

---

*Cynoglossum asperrimum* var. *yesoense* Nakai in Bot. Mag. (Tokyo) 37: 7, 69. 1923

- IPNI: absent (2014-11-05)
- ASSESSMENT: [*Cynoglossum asperrimum* Nakai]
- STATUS: var. nov.
- REFERENCE: Link
- BASIS: Original material: Yeso: Moiwa prov. Ishikari (T.Nakai). Sapporo (K. Miyabe) Yubarisan (G. Koidzumi). Jyozankei (J. Matsamura). Asahigawa (G. Koidzumi) [from protologue]

---

*Cynoglossum asperum* (Stocks) Greuter & Stier in Biodivers. Data J. [hoc loco]. 2015

- ASSESSMENT: accepted
- STATUS: comb. nov.
- BASIS: Basionym: *Paracaryum asperum* Stocks

---

*Cynoglossum atlanticum* Murb. in Bot. Not. 1922: 275. 1922

- IPNI: Cynoglossum atlanticum Murb. -- Bot. Not. 1922, 275. (IK)
- ASSESSMENT: [*Cynoglossum creticum* Mill.]
- STATUS: spec. nov.
- REFERENCE: Link
- SYNONYMY:   
  ≡ *Cynoglossum creticum* var. *atlanticum* (Murb.) Maire 1934
- BASIS: Original material: In reg. infer. Atlantis Majoris: Amismiz [from protologue]. Holotype: 5 May 1921, Murbeck, LD (LD1226779) \*

---

*Cynoglossum aucheri* (A. DC.) Greuter & Burdet in Willdenowia 11: 34. 1981

- IPNI: Cynoglossum aucheri (A.DC.) Greuter & Burdet -- Willdenowia 11(1): 34. 1981 (IK)
- ASSESSMENT: accepted
- STATUS: comb. nov.
- REFERENCE: Link
- BASIS: Basionym: *Mattia aucheri* A. DC.

---

*Cynoglossum austiniae* Eastw. in Bull. Torrey Bot. Club 32: 203. 1905 *(‘austinae’)*

- IPNI: Cynoglossum austinae Eastw. -- Bull. Torrey Bot. Club 32: 203. 1905 (GCI)
- ASSESSMENT: Not *Cynoglossum* s. l.; genus uncertain [*“Cynoglossum” grande* Douglas ex Lehm.]
- STATUS: spec. nov.
- REFERENCE: Link
- BASIS: Original material: Collected by Mrs. C. C. Bruce, the daughter of Mrs. R. M. Austin, in whose honor it is named, at Butte Creek, Calfornia (sic!), March and June, 1897, being 2092 of her collection [from protologue]. Holotype: CAS \*

---

*Cynoglossum australe* R. Br., Prodr. Fl. Nov. Holland.: 495. 1810

- IPNI: Cynoglossum australe R.Br. -- Prodr. Fl. Nov. Holland. 495. 1810 [27 Mar 1810] (IK)
- ASSESSMENT: accepted
- STATUS: spec. nov.
- REFERENCE: Link
- BASIS: Original material: J. D. *(v. v.)* [Port Jackson area and Van Diemen Island (vidi vivum = seen living)] [from protologue]. Specimens: BM (BM001040566, BM001040567)\*,GH (GH00092801)

---

*Cynoglossum australe* var. *australe* R. Br. in Engler, Pflanzenr. 78 (IV.252): 132. 1921

- IPNI: Cynoglossum australe var. australe R.Br. -- Engler's Das Pflanzenreich Heft 78 1921 (APNI)
- STATUS: [autonym]
- REFERENCE: Link

---

*Cynoglossum australe* var. *drummondii* (Benth.) Brand in Engler, Pflanzenr. 78 (IV.252): 133. 1921

- IPNI: Cynoglossum australe var. drummondii (Benth.) Brand -- Engler's Das Pflanzenreich Heft 78 1921 (APNI)
- ASSESSMENT: [*Cynoglossum australe* R. Br.]
- STATUS: comb. & stat. nov. [species to variety]
- REFERENCE: Link
- BASIS: Basionym: *Cynoglossum drummondii* Benth.

---

*Cynoglossum austriacum* Rech. in Ann. Naturhist. Mus. Wien 38: 151. 1925, pro hybr.

- IPNI: Cynoglossum × austriacum Rech. -- Ann. Naturhist. Mus. Wien 38: 151. 1925 (IK)
- ASSESSMENT: accepted as hybrid
- STATUS: hybr. nov. [*C. hungaricum* × *C. officinale*]
- REFERENCE: Link
- BASIS: Original material: Austria inferior: Marchfeld. In pinetis sabulosis “Weikendorfer Kemise” (1924) inter parentes [from protologue]. Syntype: 19 Jun 1924, *Rechinger*, U (U0000806\*)

---

*Cynoglossum austroafricanum* of Weimarck [Jacot Guillarmot, Fl. Lesotho: 233. 1971] *(‘austro-africanum’)*

- IPNI: absent (2014-11-05)
- STATUS: nom. inval. [nom. nud.]
- REFERENCE: Link

---

*Cynoglossum austroafricanum* Weim. ex Hilliard & B. L. Burtt in Notes Roy. Bot. Gard. Edinburgh 43: 347. 1986

- IPNI: Cynoglossum austroafricanum Hilliard & B.L.Burtt -- Notes Roy. Bot. Gard. Edinburgh 43(3): 347. 1986 (IK)
- ASSESSMENT: [*Cynoglossum hirsutissimum* Lehm.]
- STATUS: spec. nov.
- REFERENCE: Link
- BASIS: Original material: 2929 CB, Cobham Forest Reserve, Sipongweni, c. 6500 ft, 21 ii 1981, Hilliard & Burtt 14072 (E holo., NU iso.). [+ many paratypes] [from protologue]. Holotype: E; Isotype: NU

---

*Cynoglossum austroechinatum* (Popov) Greuter & Stier in Biodivers. Data J. [hoc loco]. 2015

- ASSESSMENT: accepted
- STATUS: comb. nov.
- BASIS: Basionym: *Rindera austroechinata* Popov

---

*Cynoglossum azocartii* Phil. in Anales Univ. Chile 65: 62. 1884 *(‘azocarti’)*

- IPNI: Cynoglossum azocarti Phil. -- Anales Univ. Chile (1884), reimp. 8. (IK)
- ASSESSMENT: Not *Cynoglossum* s. l.; genus uncertain [*“Cynoglossum” paniculatum* Hook. & Arn.]
- STATUS: spec. nov.
- REFERENCE: Link
- SYNONYMY:   
  ≡ *Cynoglossum paniculatum* var. *azocartii* (Phil.) Reiche 1907
    
  ≡ *Cynoglossum paniculatum* f. *azocartii* (Phil.) Brand 1921
- BASIS: Original material: Prope Constitucion invenit ornatissimus Raphael Azocart [from protologue]. Syntypes: (SGO000004028\*, SGO000004029\*)
- COMMENTS: Correction of the epithet’s original spelling mandated by ICN Art. 60.12.

---

*Cynoglossum badghysii* (F. Sadat) Greuter & Stier in Biodivers. Data J. [hoc loco]. 2015

- ASSESSMENT: accepted
- STATUS: comb. nov.
- BASIS: Basionym: *Mattiastrum badghysii* F. Sadat

---

*Cynoglossum baeticum* Sutorý in Willdenowia 36: 137. 2006

- IPNI: Cynoglossum baeticum Sutorý -- Willdenowia 36(1): 137 (-144; figs. 1-6). 2006 [27 Feb 2006]
- ASSESSMENT: [*Cynoglossum melananthum* Pau]
- STATUS: spec. nov.
- REFERENCE: Link
- BASIS: Holotype: Hispania, provincia de Jaén, Sierra de Segura, in valle supra fontes fluminis Quadalquivir dictis, 1400 m, 1.6.1996, K. Sutorý (BRNM 694 870) [from protologue]

---

*Cynoglossum baicalense* Pall. ex Roem. & Schult., Syst. Veg. 4: 764. 1819

- IPNI: Cynoglossum baicalense Pall. ex Roem. & Schult. -- Syst. Veg., ed. 15 bis [Roemer & Schultes] 4: 764. 1819 (IK)
- ASSESSMENT: [*Craniospermum subvillosum* Lehm.]
- STATUS: spec. nov.
- REFERENCE: Link
- SYNONYMY:   
  ≡ *Solenanthus baicalensis* (Pall. ex Roem. & Schult.) DC. 1846
- BASIS: Original material: Ad lacum Baikal. Pall. [from protologue]. Probable holotype: HAL (HAL\_0115150)\*

---

*Cynoglossum bakhtiaricum* (Khat.) Greuter & Stier in Biodivers. Data J. [hoc loco]. 2015

- ASSESSMENT: accepted
- STATUS: comb. nov.
- BASIS: Basionym: *Solenanthus bakhtiaricus* Khat.

---

*Cynoglossum barbaricinum* Arrigoni & Selvi in Webbia 66: 40. 2011

- IPNI: Cynoglossum barbaricinum Arrigoni & Selvi -- Webbia 66(1): 40 (39-43; fig. 1, map). 2011 [6 Jun 2011]
- ASSESSMENT: accepted
- STATUS: spec. nov.
- REFERENCE: Link
- BASIS: Holotype: Orgosolo, Supramonte, radure nella lecceta di Sas Baddes, 22 Jun 1972, Arrigoni & Nardi (FI) [from protologue]

---

*Cynoglossum basuticum* of Weimarck [Jacot Guillarmot, Fl. Lesotho: 234. 1971]

- IPNI: absent (2014-11-05)
- STATUS: nom. inval. [nom. nud.]
- REFERENCE: Link

---

*Cynoglossum bequaertii* De Wild. in Rev. Zool. Africaine 8: B18. 1920 *(‘bequaerti’)*

- IPNI: Cynoglossum bequaertii De Wild. -- Rev. Zool. Bot. Africaines viii. Suppl. Bot 18 (1920). (IK)
- ASSESSMENT: Not *Cynoglossum* s. l.; genus uncertain [*“Cynoglossum” amplifolium* Hochst. ex A. DC.]
- STATUS: spec. nov.
- REFERENCE: Link
- BASIS: Original material: [Zaire] Ruwenzori, 12 avril 1914 (J. Bequaert, no. 3590. – Vers 2,000 à 1,200 m. d’altitude) [from protologue]. Syntypes: BR (BR0000008865311\*, BR0000008865328\*) (BR, holo.)
- COMMENTS: Correction of the epithet’s original spelling mandated by ICN Art. 60.12.

---

*Cynoglossum berteroi* Colla, Herb. Pedem. 4: 256. 1835 [Aug]; & in Mem. Reale Accad. Sci. Torino 38: 129. 1835 [Nov-Dec] *(‘berteri’)*

- IPNI: Cynoglossum berteroi Colla -- Herb. Pedem. iv. 256 (1835). (IK)
- ASSESSMENT: *Selkirkia berteroi*  (Colla) Hemsl.
- STATUS: spec. nov.
- REFERENCE: Link
- SYNONYMY:   
  ≡ *Selkirkia berteroi*  (Colla) Hemsl. 1884
- BASIS: Original material: In praeruptis sylvaticis montium editiorum ins: Juan-Fermandez Berter: … Colla pl: rar: chil: fasc: III. tab. XLII. ined: [from protologue]. Original specimen: TO [foto!], duplicate:– Bertero 35, G (G00236137\*), Bertero 1449, G-DC (G00205848\*)
- COMMENTS: Correction of the epithet’s original spelling mandated by ICN Art. 60.7). Name subsequently published in Mem. Reale Accad. Sci. Torino 38: 132, t. 43. 1835 [Nov-Dec].

---

*Cynoglossum berteroi* of Colla [DC., Prodr. 10: 153. 1846] *(‘berteri’)*

- IPNI: Cynoglossum berteroi Colla ex DC. -- Prodr. [A. P. de Candolle] 10: 153. 1846 [8 Apr 1846] (IK)
- STATUS: [isonym]
- REFERENCE: Link

---

*Cynoglossum bicolor* Willd., Enum. Pl.: 180. 1809

- IPNI: Cynoglossum bicolor Willd. -- Enum. Pl. [Willdenow] 1: 180. 1809 [Apr 1809] (IK)
- ASSESSMENT: [*Cynoglossum officinale* L.]
- STATUS: spec. nov.
- REFERENCE: Link
- SYNONYMY:   
  ≡ *Cynoglossum officinale* var. *bicolor* (Willd.) Lehm. 1821
    
  ≡ *Cynoglossum officinale* f. *bicolor* (Willd.) Asch. & Graebn. 1899
    
  ≡ *Cynoglossum officinale* subvar. *bicolor* (Willd.) Rouy 1908
- BASIS: Original material: in Germania. ♂. [biennial] D. [cultivated out of doors] [from protologue]

---

*Cynoglossum biebersteinii* Greuter & Burdet in Willdenowia 11: 34. 1981

- IPNI: Cynoglossum biebersteinii (DC.) Greuter & Burdet -- Willdenowia 11(1): 34. 1981 (IK)
- ASSESSMENT: *Cynoglossum dubium* (Fisch. & C. A. Mey.) Greuter & Stier
- STATUS: nom. illeg. [superfl.]
- REFERENCE: Link
- BASIS: Replaced synonym: *Solenanthus dubius* Fisch. & C. A. Mey.

---

*Cynoglossum birkinshawii* J. S. Mill. in Adansonia, ser. 3, 27: 115. 2005

- IPNI: Cynoglossum birkinshawii J.S.Mill. -- Adansonia ser. 3, 27(1): 115 (-118; figs. 1-2). 2005 [30 Jun 2005]
- ASSESSMENT: accepted
- STATUS: spec. nov.
- REFERENCE: Link
- BASIS: Original material: Birkinshaw 915, Madagascar, Prov. Antsiranana, Tsaratanana Massif, Mahatsabory Mica, 12 km N of Mangindrano, around dried-up lake and adjacent mid-elevation humid evergreen forest, 2050 m, 14°09’09”S, 48°57’21”E, fl., 15 Oct. 2001 (holo-, MO!, iso-, TAN), + several paratypes [from protologue]. Holotype: MO (MO5789723\*); isotype: TAN

---

*Cynoglossum borbonicum* Bory, Voy. Iles Afrique 2: 382. 1804

- IPNI: Cynoglossum borbonicum Bory -- Voy. Iles Afrique 2: 382. 1804 (IK)
- ASSESSMENT: accepted
- STATUS: spec. nov.
- REFERENCE: Link
- BASIS: Original material: La Réunion, Plaine des Cafres, Bory. [G? Fl. Masc.]
- COMMENTS: Prevents the transfer to *Cynoglossum* of its taxonomic synonym *Myosotis borbonica*  Lam. (1791), earlier and independently described.

---

*Cynoglossum borbonicum* var. *adscendens* A. DC. in Candolle, Prodr. 10: 152. 1846

- IPNI: absent (2014-11-05)
- ASSESSMENT: [*Cynoglossum borbonicum* Bory]
- STATUS: var. nov.
- REFERENCE: Link
- BASIS: Original material: in Mauritio aut Borbonia (A. DC. v. s. comm. a Mus. par.) [from protologue]. Holotype: G-DC (G00205655)
- COMMENTS: The reference to Mauritius is incorrect: the species is endemic to La Réunion (Borbonia).

---

*Cynoglossum borbonicum* var. *angustifolium* Bory ex DC., Prodr. 10: 151. 1846

- IPNI: absent (2014-11-05)
- ASSESSMENT: [*Cynoglossum borbonicum* Bory]
- STATUS: var. nov.
- REFERENCE: Link
- BASIS: Original material: La Réunion, Plaine des Cafres [from Bory, Voy. Iles Afrique 2: 382. 1804]; (v. s. cum fl. et fr.) [from protologue]. Type specimens in G-DC\*

---

*Cynoglossum borbonicum* var. *latifolium* Bory ex DC., Prodr. 10: 151. 1846

- IPNI: absent (2014-11-05)
- ASSESSMENT: [*Cynoglossum borbonicum* Bory]
- STATUS: var. nov.
- REFERENCE: Link
- BASIS: Original material: La Réunion, Plaine des Cafres [from Bory, Voy. Iles Afrique 2: 382. 1804].(v. s. sine fr. maturo) [from protologue]. Type specimens in G-DC\*

---

*Cynoglossum boreale* Fernald in Rhodora 7: 250. 1905

- IPNI: Cynoglossum boreale Fernald -- Rhodora 7: 250. 1905 (GCI)
- ASSESSMENT: Not *Cynoglossum* s. l.; genus uncertain (*“Cynoglossum” virginianum* subsp. *boreale* (Fernald) A. Haines)
- STATUS: spec. nov.
- REFERENCE: Link
- SYNONYMY:   
  ≡ *Cynoglossum virginianum* var. *boreale* (Fernald) Cooperr. 1984
    
  ≡ *Cynoglossum virginianum* subsp. *boreale* (Fernald) A. Haines 2010
- BASIS: Original material: Quebec, beneath Larix in sandy alluvial woods. Little Cascapedia River, July 17, 1905 Williams, Collins & Fernald): New Brunswick, gorge of Aroostook River, July 17, 1902 (Williams, Collins & Fernald): Maine, woods, Orono, June 17, 1873 (F. Lamson-Scribner), June 5, 1898 (E. D. Merrill); open woods. South Chesterville, June 23, 1902 (Z. O. Eaton); Harrison (A. P. Chute): New Hampshire, Franconia, June 16, 1886, June 14, 1887 (E. & C. E. Faxon): Vermont, Bristol, May 26, 1898, and woods near Lake Dunmore, Salisbury, June 2, 1898 (E. Brainerd): New York, Salamanca (G. W. Clinton): Michigan, Sault Ste. Marie, June, 1831 (Houghton); Copper Harbor, August, 1885 (O. A. Farwell, no. 295): Ontario, sandy woods, Nipigon River, July 3, 1884 (J. Macoun): British Columbia, Donald, Columbia Valley, July 3, 1885 (J. Macoun) [from protologue]. Syntypes: GH (GH00096670\*, GH00096671\*), etc.

---

*Cynoglossum boscoi* of Sennen & Mauricio, Cat. Fl. Rif Orient.: 82. 1933

- IPNI: Cynoglossum boscoi Sennen & Mauricio -- Cat. Fl. Rif Orient. 82, nomen. 1933 (IK)
- STATUS: nom. inval. [nom. nud.]
- REFERENCE: Link

---

*Cynoglossum bottae* Deflers, Voyage Yemen: 173. 1889

- IPNI: Cynoglossum bottae Deflers -- Voyage Yemen 173. 1889 (IK)
- ASSESSMENT: Not *Cynoglossum* s. l.; genus uncertain (species accepted)
- STATUS: spec. nov.
- REFERENCE: Link
- SYNONYMY:   
  ≡ *Paracynoglossum bottae* (Deflers) R. R. Mill & A. G. Mill. 1984
- BASIS: Original material: Ad fauces montis Schibam (Haraz); alt. 2400–2800 m. (Exs. Nos 300 et 363) [from protologue]. Syntypes: [Yemen Arab Republic] Deflers 300, P; Deflers 363, P (P00622858\*)

---

*Cynoglossum brachystemon* (Fisch. & C. A. Mey.) Greuter & Stier in Biodivers. Data J. [hoc loco]. 2015

- ASSESSMENT: accepted
- STATUS: comb. nov.
- BASIS: Basionym: *Solenanthus brachystemon* Fisch. & C. A. Mey.

---

*Cynoglossum bracteolatum* Opiz in Berchtold & al., Oekon.-Techn. Fl. Böhm. 2(2): 158. 1839

- IPNI: absent (2014-11-05)
- ASSESSMENT: [*Cynoglossum officinale* L.]
- STATUS: spec. nov.
- REFERENCE: Link
- BASIS: Original material: An unbebauten und Schuttstellen. Časlau, 1807, Opiz [from protologue]

---

*Cynoglossum brassicifolium* Lag., Gen. Sp. Pl.: 10. 1816 *(‘brassicaefolium’)*

- IPNI: Cynoglossum brassicifolium Lag. -- Gen. Sp. Pl. [Lagasca] 10. 1816 (IK)
- ASSESSMENT: *Omphalodes brassicifolia*  (Lag.) Sweet
- STATUS: spec. nov.
- REFERENCE: Link
- SYNONYMY:   
  ≡ *Omphalodes brassicifolia*  (Lag.) Sweet 1826
- BASIS: Original material: in Regno Cordubense [from protologue]. Lectotype (López Gonzalez in Anales Jard. Bot. Madrid 37: 77—84. 1980): MA (MA94698\*) Lopez Gonzalez (Taxon 33: 336—337. 1984)
- COMMENTS: Correction of the epithet’s original spelling mandated by ICN Art. 60.8. A proposal to reject this name (López Gonzalez in Anales Jard. Bot. Madrid 37: 77—84. 1980) has not been approved.

---

*Cynoglossum bungei* (Boiss.) Greuter & Stier in Biodivers. Data J. [hoc loco]. 2015

- ASSESSMENT: accepted
- STATUS: comb. nov.
- BASIS: Basionym: *Mattia bungei* Boiss.

---

*Cynoglossum caeruleum* of Buchanan-Hamilton [D. Don, Prodr. Fl. Nepal.: 100. 1825]

- IPNI: Cynoglossum caeruleum Buch.-Ham. ex D.Don -- Prodr. Fl. Nepal. 100. 1825 [26 Jan-1 Feb 1825] ; nom. inval. (IK)
- STATUS: nom. inval. [pro syn. sub *Cynoglossum furcatum*]
- REFERENCE: Link

---

*Cynoglossum caesareum* Greuter & Burdet in Willdenowia 11: 34. 1981

- IPNI: Cynoglossum caesareum Greuter & Burdet -- Willdenowia 11(1): 34, nom. nov. 1981 (IK)
- ASSESSMENT: accepted
- STATUS: nom. nov.
- REFERENCE: Link
- BASIS: Replaced synonym: *Paracaryum cappadocicum* Boiss. & Balansa, non *Cynoglossum cappadocicum* Willd.

---

*Cynoglossum caespitosum* (A. DC.) Greuter & Burdet in Willdenowia 11: 34. 1981

- IPNI: Cynoglossum caespitosum (A.DC.) Greuter & Burdet -- Willdenowia 11(1): 34. 1981 (IK)
- ASSESSMENT: accepted
- STATUS: comb. nov.
- REFERENCE: Link
- BASIS: Basionym: *Mattia caespitosa* A. DC.

---

*Cynoglossum californicum* (A. Gray) A. Gray, Syn. Fl. N. Amer., ed. 2, 2: 476. 1886

- IPNI: Cynoglossum californicum A.Gray -- Syn. Fl. N. Amer. 2(1): 476. 1878 [May 1878] (IK)
- ASSESSMENT: *Hackelia californica*  (A. Gray) I. M. Johnst.
- STATUS: comb. nov.
- REFERENCE: Link
- BASIS: Basionym: *Echinospermum californicum*  A. Gray
- COMMENTS: Published in the index to Gray’s book as an apparent replacement for *Echinospermum californicum*, adopted in the preceding main text (p. 422). Although presumably due to an unintentional error [Brand 1921: 149], it is technically a validly published new combination.

---

*Cynoglossum calycinum* of Wallich., Numer. List: 26. 1829

- IPNI: Cynoglossum calycinum Wall. -- Numer. List [Wallich] n. 923. 1829 (IK)
- STATUS: nom. inval. [nom. nud.]
- REFERENCE: Link
- COMMENTS: The plants belong to *Cynoglossum wallichii* G. Don.

---

*Cynoglossum calycinum* C. A. Mey., Verz. Pfl. Casp. Meer.: 100. 1831

- IPNI: Cynoglossum calycinum C.A.Mey. -- Verz. Pfl. Casp. Meer. (C.A. von Meyer). 100. 1831 [Nov-Dec 1831] (IK)
- ASSESSMENT: *Suchtelenia calycina*  (C. A. Mey.) A. DC.
- STATUS: spec. nov.
- REFERENCE: Link
- SYNONYMY:   
  ≡ *Suchtelenia calycina*  (C. A. Mey.) A. DC. 1846
- BASIS: Original material: Ad crateres argillam cum aqua et aere eructuantes prope Karavansarai Koete-Koili in deserto inter Baku et Sallian [from protologue]. Type material: LE; isotype?: MPU (MPU019702\*)

---

*Cynoglossum campanulatum* (Riedl) Greuter & Stier in Biodivers. Data J. [hoc loco]. 2015

- ASSESSMENT: accepted
- STATUS: comb. nov.
- BASIS: Basionym: *Lindelofia campanulata* Riedl

---

*Cynoglossum canescens* Willd., Enum. Pl.: 180. 1809

- IPNI: Cynoglossum canescens Willd. -- Enum. Pl. [Willdenow] 1: 180. 1809 [Apr 1809] (IK)
- ASSESSMENT: Not *Cynoglossum* s. l.; genus uncertain [*“Cynoglossum” lanceolatum* Forssk.]
- STATUS: spec. nov.
- REFERENCE: Link
- SYNONYMY:   
  ≡ *Cynoglossum hispidum* Jacq. ex Desf.1815, nom. illeg.
- BASIS: Original material: in India orientali. C. [grown in hothouse] [from protologue]. Original specimens: B-W (B-W03335-01-0\*, B-W03335-01-0\*); Verdcourt (in Polhill, Fl. Trop. E. Africa, Boragin.: 112. 1991)
- COMMENTS: In synonymy, Willdenow cited *Cynoglossum micranthum* Desf. [inval.] and “*C. hispidum* Jacquin” which does not existed. The latter citation is an error for “*Cynoglossum hirsutum* sensu Jacquin” and may be taken to refer to Jacquin’s illustration (Hort. Schoenbrunn.: t. 489. 1804), which is also original material for Willdenow’s name. The type designated by Verdcourt (in Polhill, Fl. Trop. E. Africa, Boragin.: 112. 1991), however, is not original material and has no standing.

---

*Cynoglossum canescens* of Wallich, Numer. List: 26. 1829

- IPNI: Cynoglossum canescens Wall. -- Numer. List [Wallich] n. 918, partim. 1829 (IK)
- STATUS: [isonym]
- REFERENCE: Link
- COMMENTS: Wallich’s plants apparently belong to *Cynoglossum wallichii* G. Don.

---

*Cynoglossum cappadocicum* Willd., Sp. Pl. 1: 767. 1798

- IPNI: Cynoglossum cappadocicum Willd. -- Sp. Pl., ed. 4 [Willdenow] 1(2): 767. 1798 [Jul 1798] (IK)
- ASSESSMENT: *Omphalodes cappadocica*  (Willd.) DC.
- STATUS: spec. nov.
- REFERENCE: Link
- SYNONYMY:   
  ≡ *Omphalodes cappadocica*  (Willd.) DC. 1846
- BASIS: Original material: Omphalodes orientalis corni folio. Tournef. Cor. 7. Habitat in Cappadocica (v. s.) [from protologue]. Holotype: Roestel, B-W (B-W03345-01-0\*); probable isotype: P-TRF 665 (fide Davis, Fl. Turkey 6: 281. 1978)

---

*Cynoglossum capusii* (Franch.) Pazij in Bot. Mater. Gerb. Inst. Bot. Akad. Nauk Uzbeksk. S.S.R. 16: 43. 1961

- IPNI: Cynoglossum capusii (Franch.) Pazij -- in Not. Syst. Herb. Inst. Bot. Acad. Sci. Uzbekistan. xvi. 43 (1961). (IK)
- ASSESSMENT: accepted
- STATUS: comb. nov.
- REFERENCE: Link
- BASIS: Basionym: *Paracaryum capusii* Franch.
- COMMENTS: Has priority over *Cynoglossum tianschanicum* Popov.

---

*Cynoglossum castaneum* Riedl in Blumea 38: 462. 1994

- IPNI: Cynoglossum castaneum Riedl -- Blumea 38(2): 462 (1994). (IK)
- ASSESSMENT: accepted
- STATUS: spec. nov.
- REFERENCE: Link
- BASIS: Holotype: Central Java, Blumbang, Mt. Lawu, 26-xi-1982, *Afriastini 488* (K) [from protologue]. Isotype: A (A00075139\*)

---

*Cynoglossum castellanum* Pau, Not. Bot. Fl. Españ. 6: 80. 1895

- IPNI: absent (2014-11-05)
- ASSESSMENT: [*Cynoglossum officinale* L.]
- STATUS: spec. nov.
- REFERENCE: Link
- BASIS: Original material: *C. pictum*, F. F. Iparraguirre, hb. Habitat in dumetis. – Lecta Guadalajara. – 17 Maj. 1878. F. Fernández, in schedula [from protologue]

---

*Cynoglossum castrilense* Pau, Carta Bot. 3: 6. 1906

- IPNI: Cynoglossum castrilense Pau -- Cart. Bot. 3a; cf. Cuatrec. in Trab. Mus. Cienc. Nat. Barcelona, xii. 386 (1929), in syn. (IK)
- ASSESSMENT: [*Cynoglossum pustulatum* Boiss.]
- STATUS: spec. nov.
- REFERENCE: Link
- SYNONYMY:   
  ≡ *Cynoglossum valentinum* f. *castrilense* (Pau) Degen & Hervier 1907
    
  ≡ *Cynoglossum dioscoridis* var. *castrilense* (Pau) Brand 1921
- BASIS: Original material: not indicated. Type (inferred; sée Hervier in Bull. Acad. Int. Géogr. Bot. 17: 60. 1907): Sierra del Castril, bois, très rare, à 1700 mètres, juin, E. Reverchon

---

*Cynoglossum cavaleriei* H. Lév. in Repert. Spec. Nov. Regni Veg. 12: 534. 1913

- IPNI: Cynoglossum cavaleriei H.Lév. -- Repert. Spec. Nov. Regni Veg. 12: 534. 1913 (IK)
- ASSESSMENT: [*Antiotrema dunnianum* (Diels) Hand.-Mazz.]
- STATUS: spec. nov.
- REFERENCE: Link
- BASIS: Original material: Kouy-Tchéou: Gan-Pin rare, avril 1910 [recte: 1904] (Jul. Cavalerie, 2117) [from protologue]. Syntypes: E (E00284707\* E00284708\*), GH (GH00096675\*), W (W19240012369\*)

---

*Cynoglossum ceilanicum* of Thunberg, Mus. Nat. Acad. Upsal. 1-22: 166. 1792

- IPNI: absent (2014-11-05)
- STATUS: nom. inval. [nom. nud.]
- REFERENCE: Link
- COMMENTS: The epithet is a spelling variant of *zeylanicum*, subsequently validated in various combinations; see *Anchusa zeylanica* Vahl ex Hornem. (1813).

---

*Cynoglossum celebicum* Brand in Engler, Pflanzenr. 78 (IV.252): 147. 1921

- IPNI: Cynoglossum celebicum Brand -- Pflanzenr. (Engler) Borrag. Cynogloss. 147. 1921 (IK)
- ASSESSMENT: accepted
- STATUS: spec. nov.
- REFERENCE: Link
- BASIS: Holotype: Celebes: Loka, 13. Oktober 1895 (Gebr. Sarasin n. 1289; Herb. Berlin) [from protologue]; destroyed in B in World War II

---

*Cynoglossum cerinthoides* (Boiss.) Greuter & Burdet in Willdenowia 11: 34. 1981

- IPNI: Cynoglossum cerinthoides (Boiss.) Greuter & Burdet -- Willdenowia 11(1): 34. 1981 (IK)
- ASSESSMENT: accepted
- STATUS: comb. nov.
- REFERENCE: Link
- BASIS: Basionym: *Solenanthus cerinthoides*  Boiss.

---

*Cynoglossum cernuum* Baker in J. Linn. Soc., Bot. 20: 211. 1883

- IPNI: Cynoglossum cernuum Baker -- J. Linn. Soc., Bot. 20: 211. 1883 [1884 publ. 1883] (IK)
- ASSESSMENT: accepted
- STATUS: spec. nov.
- REFERENCE: Link
- BASIS: Original material: Central Madagascar, Baron 2033 [from protologue]. Holotype: K (K000418912); isotype: P (P00417683)

---

*Cynoglossum cheirifolium* L., Sp. Pl.: 134. 1753

- IPNI: Cynoglossum cheirifolium L. -- Sp. Pl. 1: 134. 1753 [1 May 1753] (IK)
- ASSESSMENT: accepted
- STATUS: spec. nov.
- REFERENCE: Link
- SYNONYMY:   
  ≡ *Pardoglossum cheirifolium* (L.) Barbier & Mathez 1973
- BASIS: Original material: in Creta. Hispania [from protologue]. Lectotype (Mill in Taxon 53: 801. 2004): Herb. Burser XIV(2): 32 (UPS)

---

*Cynoglossum cheirifolium* of Jacquin, Collectanea 3: 30. 1791

- IPNI: Cynoglossum cheirifolium Jacq. -- Coll. iii. 30. (IK)
- STATUS: [isonym]
- REFERENCE: Link

---

*Cynoglossum cheirifolium* of Sieber [Nyman, Consp. Fl. Eur. 3: 522. 1881]

- IPNI: Cynoglossum cheirifolium Sieber ex Nyman -- Consp. Fl. Eur. 3: 522. 1881 [prob. Jul 1881] (IK)
- STATUS: nom. inval. [pro syn. sub *Cynoglossum columnae*]
- REFERENCE: Link

---

*Cynoglossum cheirifolium* f. *ambiguum* Font Quer, Iter Marocc. 1927: sched. impr. No. 493. 1928

- IPNI: absent (2014-11-05)
- ASSESSMENT: [*Cynoglossum cheirifolium* subsp. *heterocarpum* (Kunze) Font Quer]
- STATUS: f. nov.
- REFERENCE: Link
- BASIS: Original material: in incultis littoris rhiphaei, pr. Marsa Saguira; Font Quer 493 [from protologue]. Isotype: MPU (MPU006272\*)
- COMMENTS: Validly published on printed herbarium label (ICN Art. 29.1 & 30.7).

---

*Cynoglossum cheirifolium* var. *antiatlanticum* Molero & J. M. Monts. in Treb. Inst. Bot. Barcelona 11: 25. 1987

- IPNI: Cynoglossum cheirifolium L. var. antiatlanticum Molero & J.M.Monts. -- Treb. Inst. Bot. Barcelona 11: 25 (1987). (IK)
- ASSESSMENT: [*Cynoglossum cheirifolium* subsp. *heterocarpum* (Kunze) Font Quer]
- STATUS: var. nov.
- REFERENCE: Link
- BASIS: Original material: Agadir: Col du Kerdouss, 29RN66, 1270 m, in dumosis raris et ad arva, solo calcareo, FC-9312, 24-V-1985 [from protologue] Syntypes: M (M0105868\*), M (MA340289)

---

*Cynoglossum cheirifolium* var. *arundanum* (Coss.) Maire in Bull. Soc. Hist. Nat. Afrique N. 27: 249. 1936

- IPNI: absent (2014-11-05)
- ASSESSMENT: [*Cynoglossum cheirifolium* subsp. *heterocarpum* (Kunze) Font Quer]
- STATUS: comb. & stat. nov. [species to variety]
- REFERENCE: Link
- BASIS: Basionym: *Cynoglossum arundanum* Coss.

---

*Cynoglossum cheirifolium* var. *calcaratum* DC. in Lamarck & Candolle, Fl. Franç. ed. 3, 6: 422. 1815

- IPNI: absent (2014-11-05)
- ASSESSMENT: [*Cynoglossum cheirifolium* L.]
- STATUS: var. nov.
- REFERENCE: Link
- BASIS: Original material: trouvée à Nismes [from protologue]. Holotype: G-DC (G00205645 p.p., dissected flowers in bag)

---

*Cynoglossum cheirifolium* var. *clavatum* (Viv.) DC., Prodr. 10: 154. 1846

- IPNI: absent (2014-11-05)
- ASSESSMENT: [*Cynoglossum cheirifolium* L.]
- STATUS: comb. & stat. nov. [species to variety]
- REFERENCE: Link
- BASIS: Basionym: *Cynoglossum clavatum* Viv.

---

*Cynoglossum cheirifolium* var. *controversum* (Sennen) Maire in Bull. Soc. Hist. Nat. Afrique N. 27: 249. 1936

- IPNI: absent (2014-11-05)
- ASSESSMENT: [*Cynoglossum cheirifolium* subsp. *heterocarpum* (Kunze) Font Quer]
- STATUS: comb. & stat. nov. [species to variety]
- REFERENCE: Link
- BASIS: Basionym: *Cynoglossum controversum* Sennen

---

*Cynoglossum cheirifolium* var. *gomaricum* Font Quer in Mem. Real Acad. Ci. Barcelona 22: 19, 351. 1931

- IPNI: absent (2014-11-05)
- ASSESSMENT: [*Cynoglossum cheirifolium* subsp. *heterocarpum* (Kunze) Font Quer]
- STATUS: var. nov.
- REFERENCE: Link
- BASIS: Original material: in saxosis calc. cacuminis montis Lexhab (Gomara-Imp. maroccano), ad 2.000–2.100 m alt.; d. 25 junii legi [from protologue]

---

*Cynoglossum cheirifolium* var. *heterocarpum* Kunze in Flora 29: 694. 1846

- IPNI: absent (2014-11-05)
- ASSESSMENT: [*Cynoglossum cheirifolium* subsp. *heterocarpum* (Kunze) Font Quer]
- STATUS: var. nov.
- REFERENCE: Link
- SYNONYMY:   
  ≡ *Cynoglossum heterocarpum* (Kunze) Willk. 1891
    
  ≡ *Cynoglossum cheirifolium* subsp. *heterocarpum* (Kunze) Font Quer 1931
    
  ≡ *Pardoglossum cheirifolium* subsp. *heterocarpum* (Kunze) Mathez 1976
- BASIS: Original material: Prope oppidum Chiclana in colle arenosa Cerro Santana dicta, Febr. c. fl. et Mart. c. fr. [from protologue]

---

*Cynoglossum cheirifolium* subsp. *heterocarpum* (Kunze) Font Quer in Mem. Real Acad. Ci. Barcelona 22: 351. 1931

- IPNI: absent (2014-11-05)
- ASSESSMENT: accepted
- STATUS: stat. nov. [variety to subspecies]
- REFERENCE: Link
- BASIS: Basionym: *Cynoglossum cheirifolium* var. *heterocarpum* Kunze

---

*Cynoglossum cheirifolium* var. *lanatum* (L.) Lehm., Pl. Asperif. Nucif.: 141. 1818 *(‘lanata’)*

- IPNI: absent (2014-11-05)
- ASSESSMENT: *Cynoglossum mathezii* Greuter & Burdet
- STATUS: comb. & stat. nov. [species to variety]
- REFERENCE: Link
- BASIS: Basionym: *Anchusa lanata* L.
- COMMENTS: Published in the format: *Cynoglossum cheirifolium* β *Anchusa* (*lanata*), correctable as above under ICN Art. 24.4.

---

*Cynoglossum cheirifolium* var. *lasianthum* Murb. in Acta Univ. Lund. 2, 19: 21. 1923

- IPNI: absent (2014-11-05)
- ASSESSMENT: [*Cynoglossum cheirifolium* subsp. *heterocarpum* (Kunze) Font Quer]
- STATUS: var. nov.
- REFERENCE: Link
- BASIS: Original material: Coteaux calcaires à Oumenast et à Amismiz, 700–1100 m [*Murbeck*] [from protologue]. Syntypes: LD (LD1216094\*, LD1225275\*, LD1226293\*), MPU (MPU008010\*)

---

*Cynoglossum cheirifolium* subvar. *mariolense* (Rouy) Brand in Engler, Pflanzenr. 78 (IV.252): 147. 1921

- IPNI: absent (2014-11-05)
- ASSESSMENT: [*Cynoglossum cheirifolium* subsp. *heterocarpum* (Kunze) Font Quer]
- STATUS: comb. & stat. nov. [variety to subvariety]
- REFERENCE: Link
- BASIS: Basionym: *Cynoglossum arundanum* var. *mariolense* Rouy

---

*Cynoglossum cheirifolium* var. *stylosum* Brand in Engler, Pflanzenr. 78 (IV.252): 123. 1921

- IPNI: absent (2014-11-05)
- ASSESSMENT: [*Cynoglossum cheirifolium* subsp. *heterocarpum* (Kunze) Font Quer]
- STATUS: var. nov.
- REFERENCE: Link
- BASIS: Original material: Algier: Oran, Hügel an der Küste (Debeaux 20. 4. 82; Herb. Berlin) [from protologue]. Holotype: destroyed in B in World War II
- COMMENTS: Independent of *Cynoglossum stylosum* Kar. & Kir..

---

*Cynoglossum cheirifolium* var.  *tubuliflorum* Maire in Bull. Soc. Hist. Nat. Afrique N. 27: 249. 1936

- IPNI: absent (2014-11-05)
- ASSESSMENT: [*Cynoglossum cheirifolium* subsp. *heterocarpum* (Kunze) Font Quer]
- STATUS: var. nov.
- REFERENCE: Link
- BASIS: Original material: Callitriaies et chênaies du Grand Atlas occidental chez les Ida-ou-Tanan, sur calcaire, 1200–1600 m, en fleurs en avril [from protologue]. Holotype: MPU (MPU003681\*)
- COMMENTS: Authorship of the name ascribed to “Maire & Wilczek” on type label.

---

*Cynoglossum cheranganiense* Verdc. in Polhill, Fl. Trop. E. Africa, Boragin.: 115. 1991

- IPNI: Cynoglossum cheranganiense Verdc. -- Fl. Trop. E. Africa, Boragin. 115. 1991 (IK)
- ASSESSMENT: accepted
- STATUS: spec. nov.
- REFERENCE: Link
- BASIS: Holotype: Kenya, Elgeyo district, Cherangani Hills, Kamalagon [Kamiligon], Mabberley & McCall 207 (K, holo!) [from protologue]. Isotype: EA (EA000001268\*)

---

*Cynoglossum chilense* of Cavanilles [Brandenburg, Guide Cavanilles Herbarium: 3. 1997]

- IPNI: absent (2014-11-05)
- STATUS: nom. inval. [nom. nud.]
- REFERENCE: Link

---

*Cynoglossum ciliatum* Douglas ex Lehm., Nov. Stirp. Pug. 2: 24. 1830

- IPNI: Cynoglossum ciliatum Douglas ex Lehm. -- Nov. Stirp. Pug. [Lehmann] 2: 24. 1830 [27 Aug 1830] (IK)
- ASSESSMENT: *Hackelia ciliata*  (Douglas ex Lehm.) I. M. Johnst.
- STATUS: spec. nov.
- REFERENCE: Link
- SYNONYMY:   
  ≡ *Hackelia ciliata*  (Douglas ex Lehm.) I. M. Johnst. 1923
- BASIS: Original material: in regionibus Americae septentrionalis ad occidentem vergentibus [Lehm., Nov. Stirp. Pug. 2: 5. 1830]

---

*Cynoglossum circinnatum* (Ledeb.) Greuter & Burdet in Willdenowia 11: 34. 1981

- IPNI: Cynoglossum circinnatum (Ledeb.) Greuter & Burdet -- Willdenowia 11(1): 34. 1981 (IK)
- ASSESSMENT: accepted
- STATUS: comb. nov.
- REFERENCE: Link
- BASIS: Basionym: *Solenanthus circinnatus* Ledeb.

---

*Cynoglossum clandestinum* Desf., Fl. Atlant. 1: 159. 1798

- IPNI: Cynoglossum clandestinum Desf. -- Fl. Atlant. 1: 159, t. 42. 1798 (IK)
- ASSESSMENT: accepted
- STATUS: spec. nov.
- REFERENCE: Link
- BASIS: Original material: ad limites agrorum Algeriae [from protologue]. Lectotype (Selvi & Sutory in Pl. Biosyst. 146: 463. 2012): P (P00307445\*)

---

*Cynoglossum clandestinum* var. *fallax* Samp. in Bol. Soc. Brot. 18: 66. 1902

- IPNI: absent (2014-11-05)
- ASSESSMENT: [*Cynoglossum clandestinum* Desf.]
- STATUS: var. nov.
- REFERENCE: Link
- BASIS: Original material: Torrão (nos campos) [from protologue]

---

*Cynoglossum clavatum* Viv., Fl. Libyc. Spec.: 11. 1824

- IPNI: Cynoglossum clavatum Viv. -- Fl. Libyc. Spec. 11. t. 5. f. 2. 1824 [pre-August 1824] (IK)
- ASSESSMENT: [*Cynoglossum cheirifolium* L.]
- STATUS: spec. nov.
- REFERENCE: Link
- SYNONYMY:   
  ≡ *Cynoglossum cheirifolium* var. *clavatum* (Viv.) DC. 1846
- BASIS: Original material: [Libya]. Original illustration: l.c. t. 5, fig. 2. Original specimen (GE) presumably destroyed in World War II

---

*Cynoglossum clusii* Loisel. in Cuvier, Dict. Sci. Nat. 12: 384. 1819

- IPNI: Cynoglossum clusii Loisel. -- Dict. Sci. Nat., ed. 2. [F. Cuvier] 12: 384. 1819 [1818 publ. 9 Jan 1819] (IK)
- ASSESSMENT: [*Lappula squarrosa*  (Retz.) Dumort.]
- STATUS: nom. illeg. [superfl.]
- REFERENCE: Link
- BASIS: Replaced synonym: *Myosotis lappula*  L.

---

*Cynoglossum coechinatum* (Popov) Greuter & Stier in Biodivers. Data J. [hoc loco]. 2015

- ASSESSMENT: accepted
- STATUS: comb. nov.
- BASIS: Basionym: *Rindera coechinata* Popov

---

*Cynoglossum coelestinum* Lindl. in Edwards’s Bot. Reg. 25: t. 36. 1839

- IPNI: Cynoglossum coelestinum Lindl. -- Edwards's Bot. Reg. 25: t. 36. 1839 (IK)
- ASSESSMENT: accepted
- STATUS: spec. nov.
- REFERENCE: Link
- SYNONYMY:   
  ≡ *Paracaryum coelestinum* (Lindl.) Benth. ex C. B. Clarke 1883
    
  ≡ *Adelocaryum coelestinum* (Lindl.) Brand 1915
    
  ≡ *Paracaryopsis coelestina* (Lindl.) R. R. Mill 1991
- BASIS: Original material: raised by the Horticultural Society from seeds presentd by John Nimmo, Esq. of Bombay, and flowered the first time in August 1838 [from protologue]
- COMMENTS: *Echinospermum caelestinum* Wight (1850) is heterotypic but taxonomically synonymous.

---

*Cynoglossum coeruleum* Hochst. ex A. DC. in Candolle, Prodr. 10: 148. 1846

- IPNI: Cynoglossum coeruleum Hochst. ex DC. -- Prodr. [A. P. de Candolle] 10: 148. 1846 [8 Apr 1846] (IK)
- ASSESSMENT: Not *Cynoglossum* s. l.; genus uncertain (species accepted)
- STATUS: spec. nov.
- REFERENCE: Link
- SYNONYMY:   
  ≡ *Paracynoglossum afrocaeruleum* R. R. Mill 1984, nom. illeg.
    
  ≡ *Cynoglossum afrocaeruleum* Riedl 1985, nom. illeg.
- BASIS: Original material: in agris Abyssiniae; pl. Schimper abyss. sect. 2. n. 542 [from protologue]. Lectotype (Mill Notes Roy. Bot. Gard. Edinb.: 481. 1984) Ethiopia, Endschedcap, Schimper 542, G-DC (G00202098\*); isolectotypes: BM (BM000930344\*), BR (BR0000008359049\*), K (K000418927\*), S (S11-34573\*), STU (STU000395\*)
- COMMENTS: Erroneously believed by Mill & Miller (in Notes Royal Bot Gard. Edin. 41: 474. 1984) to be a later (para)homonym of *Cynoglossum caeruleum* of Buchanan-Hamilton [pro syn.], which was not validly published.

---

*Cynoglossum coeruleum* subsp. *geometricum* of Edwards [in Hedberg & al., Fl. Ethiopia & Eritrea 5: 93. 2006]

- IPNI: Cynoglossum coeruleum Hochst. ex DC. subsp. geometricum (Baker & C.H.Wright) S.Edwards -- Fl. Ethiopia & Eritrea 5: 93. 2006 ; nom. inval.
- STATUS: comb. inval. [lacking full basionym reference; ICN Art. 41.5]
- REFERENCE: Link
- COMMENTS: Intended basionym: *Cynoglossum geometricum* Baker & C. H. Wright (1905).

---

*Cynoglossum coeruleum* var. *hedbergiorum* of Edwards [in Hedberg & al., Fl. Ethiopia & Eritrea 5: 92. 2006]

- IPNI: Cynoglossum coeruleum Hochst. ex DC. var. hedbergiorum (Riedl) S.Edwards -- Fl. Ethiopia & Eritrea 5: 92. 2006 ; nom. inval.
- STATUS: comb. inval. [lacking full basionym reference; ICN Art. 41.5]
- REFERENCE: Link
- COMMENTS: Intended basionym: *Cynoglossum hedbergiorum* Riedl (1985).

---

*Cynoglossum coeruleum* var. *johnstonii* (Baker) Baker & Wright in Oliver & al., Fl. Trop. Afr. 4(2, 1): 54. 1905

- IPNI: absent (2014-11-05)
- ASSESSMENT: Not *Cynoglossum* s. l.; genus uncertain (*“Cynoglossum” coeruleum* subsp. *johnstonii* (Baker) Verdc.)
- STATUS: comb. & stat. nov. [species to variety]
- REFERENCE: Link
- BASIS: Basionym: *Cynoglossum johnstonii* Baker

---

*Cynoglossum coeruleum* subsp. *johnstonii* (Baker) Verdc. in Polhill, Fl. Trop. E. Africa, Boragin.: 109. 1991

- IPNI: Cynoglossum coeruleum Hochst. ex DC. subsp. johnstonii (Baker) Verdc. -- Fl. Trop. E. Africa, Boragin. 109. 1991 (IK)
- ASSESSMENT: Not *Cynoglossum* s. l.; genus uncertain (subspecies accepted)
- STATUS: stat. nov. [species to subspecies]
- REFERENCE: Link
- BASIS: Basionym: *Cynoglossum johnstonii* Baker
- COMMENTS: Incorrect as published, including the type of *Cynoglossum lanceolatum* subsp. *geometricum* (Baker & C. H. Wright) Brand (1921), but nevertheless legitimate (ICN Art. 52.3).

---

*Cynoglossum coeruleum* subsp. *kenyense* Verdc. in Polhill, Fl. Trop. E. Africa, Boragin.: 112. 1991

- IPNI: Cynoglossum coeruleum Hochst. ex DC. subsp. kenyense Verdc. -- Fl. Trop. E. Africa, Boragin. 112. 1991 (IK)
- ASSESSMENT: Not *Cynoglossum* s. l.; genus uncertain (subspecies accepted)
- STATUS: subsp. nov.
- REFERENCE: Link
- BASIS: Holotype: Kenya, Uasin Gishu Distrist, Eldoret, L. A. Cooke 45 (K, holo.!) [from protologue] (K000418922\*)

---

*Cynoglossum coeruleum* subsp. *latifolium* Verdc. in Polhill, Fl. Trop. E. Africa, Boragin.: 112. 1991

- IPNI: Cynoglossum coeruleum Hochst. ex DC. subsp. latifolium Verdc. -- Fl. Trop. E. Africa, Boragin. 112. 1991 (IK)
- ASSESSMENT: Not *Cynoglossum* s. l.; genus uncertain (subspecies accepted)
- STATUS: subsp. nov.
- REFERENCE: Link
- BASIS: Holotype: Kenya, Northern Frontier Province, Mt. Kulal, Bally 5549 (K, holo.!, EA, iso.!) [from protologue] (K000418919\*); isotype: EA (EA000001267\*)

---

*Cynoglossum coeruleum* var. *mannii* (Baker & C. H. Wright) Verdc. in Polhill, Fl. Trop. E. Africa, Boragin.: 110. 1991

- IPNI: Cynoglossum coeruleum Hochst. ex DC. var. mannii (Baker & C.H.Wright) Verdc. -- Fl. Trop. E. Africa, Boragin. 110. 1991 (IK)
- ASSESSMENT: Not *Cynoglossum* s. l.; genus uncertain [*“Cynoglossum” lanceolatum* subsp. *geometricum* (Baker & C. H. Wright) Brand]
- STATUS: comb. nov.
- REFERENCE: Link
- BASIS: Basionym: *Cynoglossum mannii* Baker & C. H. Wright

---

*Cynoglossum coeruleum* var. *winkleri* Brand in Engler, Pflanzenr. 78 (IV.252): 147. 1921

- IPNI: absent (2014-11-05)
- ASSESSMENT: Not *Cynoglossum* s. l.; genus uncertain [*“Cynoglossum” coeruleum* subsp. *kenyense* Verdc.]
- STATUS: var. nov.
- REFERENCE: Link
- BASIS: Original material: Ostafrika: zwischen Mairoti und dem ostafrikanischen Graben, überall auf der Hochebene (Winkler n. 4183) [from protologue]. Holotype: B†; isotype: WRSL (fide Verdcourt in Polhill, Fl. Trop. E. Africa, Boragin.: 112. 1991)

---

*Cynoglossum columnae* Ten., Fl. Napol. 1: 14. 1811

- IPNI: absent (2014-11-05)
- ASSESSMENT: accepted
- STATUS: spec. nov.
- REFERENCE: Link
- SYNONYMY:   
  ≡ *Rindera columnae* (Ten.) Roem. & Schult. 1819
    
  ≡ *Mattia columnae* (Ten.) G. Don 1838
- BASIS: Original material: “B. Encyclop. p. 238. Column. ecphr. pars pr. pag. 178” [from protologue]; referring to (1) an unnamed *Cynoglossum cristatum* var. ß in Lamarck, Encycl. 2: 238. 1786 and (2) the illustration of *Cynoglossa altera media fructu κοτυλώδης siue λυχνιώδης, mont.* in Colonna, Ekphr. 1: 178. 1606. This illustration, also cited by Lamarck, is the obvious choice for a type. The lectotype designated by Selvi & Cecchi (in Taxon 58: 621, 2009): “Cynoglossum columnae Ten./in pascuis montanis” Tenore, FI, is hardly appropriate as it was probably collected long after 1811, having been sent to Florence in 1844, and moreover consists of two plants in different stages of development, which probably represent two distinct gatherings

---

*Cynoglossum columnae* of Bivona, Stirp. Rar. Sicilia 2: [7]. 1814

- IPNI: Cynoglossum columnae Biv. -- Stirp. Rar. Sicil. Manip. ii. [7]. (IK)
- STATUS: [isonym]
- REFERENCE: Link

---

*Cynoglossum columnae* var. *dolopicum* Hausskn. in Mitth. Thüring. Bot. Vereins, ser. 2, 8: 54. 1895

- IPNI: absent (2014-11-05)
- ASSESSMENT: [*Cynoglossum columnae* Ten.]
- STATUS: var. nov.
- REFERENCE: Link
- BASIS: Original material: in saxosis pr. Mon. Korona P[indi]. D[olopici]. [from protologue]. Original specimen: JE (JE00016096\*)

---

*Cynoglossum columnae* var. *nebrodense* Strobl in Flora 67: 622. 1884

- IPNI: absent (2014-11-05)
- ASSESSMENT: [*Cynoglossum nebrodense* Guss.]
- STATUS: var. nov.
- REFERENCE: Link
- BASIS: Original material: Madonie (Todaro f[lora]. s[icula]. e[xsiccata].), Salto della Botte (Herb. Palermo's), Monte Scalone (Herb. Mina), Ferro soprano (Herb. Mina c. spec.). April-Juni. Dr. Mina und Todaro f. sic. exsicc. Nr. 1224 … auch am M. S. Anelo bei Neapel [from protologue]
- COMMENTS: Intended to be based on *Cynoglossum nebrodense* of Jan (“1826” [1831]) which, however, was not validly published.

---

*Cynoglossum columnae* var. *obtusifolium* of Haussknecht [Brand in Engler, Pflanzenr. 78 (IV.252): 127. 1921]

- IPNI: absent (2014-11-05)
- STATUS: nom. inval. [pro syn. sub *Cynoglossum montanum* var. *asiaticum*]
- REFERENCE: Link

---

*Cynoglossum columnae* var. *virescens* of Haussknecht and Bornmüller [Brand in Engler, Pflanzenr. 78 (IV.252): 127. 1921]

- IPNI: absent (2014-11-05)
- STATUS: nom. inval. [pro syn. sub *Cynoglossum montanum* subvar. *natolicum*]
- REFERENCE: Link

---

*Cynoglossum congestum* Poepp. of Alphonse de Candolle [in Candolle, Prodr. 10: 132. 1846]

- IPNI: Cynoglossum congestum Poepp. ex DC. -- Prodr. [A. P. de Candolle] 10: 132. 1846 [8 Apr 1846] (IK)
- STATUS: nom. inval. [pro syn. sub *Eritrichium congestum*]
- REFERENCE: Link

---

*Cynoglossum controversum* of Sennen [in Sennen & Mauricio, Cat. Fl. Rif Orient.: 82. 1933]

- IPNI: Cynoglossum controversum Sennen & Mauricio -- Cat. Fl. Rif Orient. 82. 1933 (IK)
- STATUS: nom. inval. [nom. nud.]
- REFERENCE: Link
- COMMENTS: Also appearing on undated printed labels. Validated in 1936.

---

*Cynoglossum controversum* Sennen, Diagn. Nouv.: 159, 197. 1936

- IPNI: Cynoglossum controversum Sennen -- Diagn. Nouv. 159, 197. 1936 [Jul 1936] (IK)
- ASSESSMENT: [*Cynoglossum cheirifolium* subsp. *heterocarpum* (Kunze) Font Quer]
- STATUS: spec. nov.
- REFERENCE: Link
- SYNONYMY:   
  ≡ *Cynoglossum cheirifolium* var. *controversum* (Sennen) Maire 1936
- BASIS: Original material: Maroc: Melilla et Tafersit – Sennen & Mauricio … dans le Rif oriental, et an delà du Muluya (Beni-Snassen) … Du Muluya au Nékor … Hidum, Yazinen, Taurirt, Beni-Sicar, Sidi-Had el Hach, Cabo Tres Forças, Segangan, Muley-Rechid, Beni-Medien; Ain-Zora, Kadia, Dar-Kebdani, dj. Kerker, Berkane, … Taforalt [from protologue]. Syntypes: MPU (MPU008427\*, MPU009370\*)

---

*Cynoglossum controversum* f. *albiflorum* of Sennen [in Sennen & Mauricio, Cat. Fl. Rif Orient.: 82. 1933]

- IPNI: absent (2014-11-05)
- STATUS: nom. inval. [nom. nud.]
- REFERENCE: Link

---

*Cynoglossum cordifolium* of Hochstetter [Brand in Engler, Pflanzenr. 78 (IV.252): 141. 1921]

- IPNI: Cynoglossum cordifolium Hochst. ex Brand -- Pflanzenr. (Engler) Borrag. Cynogloss. 141, in syn. 1921 (IK)
- STATUS: nom. inval. [pro syn. sub *Cynoglossum amplifolium*]
- REFERENCE: Link

---

*Cynoglossum corymbiforme* (DC. & A. DC.) Greuter & Burdet in Willdenowia 11: 34. 1981

- IPNI: Cynoglossum corymbiforme (DC. & A.DC.) Greuter & Burdet -- Willdenowia 11(1): 34. 1981 (IK)
- ASSESSMENT: accepted
- STATUS: comb. nov.
- REFERENCE: Link
- BASIS: Basionym: *Mattia corymbiformis* DC. & A. DC.

---

*Cynoglossum creticum* Mill., Gard. Dict., ed. 8: Cynoglossum n. 3. 1768

- IPNI: Cynoglossum creticum Mill. -- Gard. Dict., ed. 8. n. 3. 1768 [16 Apr 1768] (IK)
- ASSESSMENT: accepted
- STATUS: spec. nov.
- REFERENCE: Link
- BASIS: Original material: Grows naturally in Andalusia, I received the seeds of this from Gibraltar [from protologue]. Neotype (Selvi & Jarvis in Taxon 60: 1477. 2011): Spain. Andalucia, Prov. de Cádiz, 10 km W. of Puerto de Santa Maria, 17 April 1951, A.H.G. Alston 10283, BM

---

*Cynoglossum creticum* var. *atlanticum* (Murb.) Maire in Jahandiez & Maire, Cat. Pl. Maroc: 593. 1934

- IPNI: absent (2014-11-05)
- ASSESSMENT: [*Cynoglossum creticum* Mill.]
- STATUS: comb. & stat. nov. [species to variety]
- REFERENCE: Link
- BASIS: Basionym: *Cynoglossum atlanticum* Murb.

---

*Cynoglossum creticum* var. *doumerguei* Sennen & Mauricio in Sennen, Diagn. Nouv.: 238. 1936

- IPNI: absent (2014-11-05)
- ASSESSMENT: [*Cynoglossum creticum* Mill.]
- STATUS: var. nov.
- REFERENCE: Link
- BASIS: Original material: Maroc: Beni-Sicar; Bco. de Hidum. Abonde à Taforalt, Zegzel (Beni-Snassen) [from protologue]. Syntype: Hidum, *Sennen & Mauricio 9487*, MPU (MPU009431\*)

---

*Cynoglossum creticum* f. *micranthum* Faure & Maire in Bull. Soc. Hist. Nat. Afrique N. 29: 437. 1938

- IPNI: absent (2014-11-05)
- ASSESSMENT: [*Cynoglossum creticum* Mill.]
- STATUS: f. nov.
- REFERENCE: Link
- BASIS: Original material: Maroc oriental: Martimprey du Kiss (A. Faure). Syntypes: Faure s.n., 18.4.1937 MPU (MPU004014\*, MPU004015\*)

---

*Cynoglossum creticum* f. *pallidum* Maire in Jahandiez & Maire, Cat. Pl. Maroc: 593. 1934 *(‘pallida’)*

- IPNI: absent (2014-11-05)
- ASSESSMENT: [*Cynoglossum creticum* Mill.]
- STATUS: f. nov.
- REFERENCE: Link
- BASIS: Original material: R. Sidi Mohammed Zekri (F.-Q. et Maire) [from protologue]. Holotype: 21.6.1929, MPU (MPU001215\*)

---

*Cynoglossum creticum* var. *pictum* (Aiton) Maire in Jahandiez & Maire, Cat. Pl. Maroc: 593. 1934

- IPNI: absent (2014-11-05)
- ASSESSMENT: [*Cynoglossum creticum* Mill.]
- STATUS: comb. & stat. nov. [species to variety]
- REFERENCE: Link
- BASIS: Basionym: *Cynoglossum pictum* Aiton

---

*Cynoglossum creticum* subvar. *pseudocheirifolium* Brand in Engler, Pflanzenr. 78 (IV.252): 130. 1921

- IPNI: absent (2014-11-05)
- ASSESSMENT: [*Cynoglossum creticum* Mill.]
- STATUS: subvar. nov.
- REFERENCE: Link
- BASIS: Original material: hauptsächlich auf Kreta, wo die typische Form anscheinend fehlt (Sieber, Weiß usw.). Außerdem in Griechenland hin und wieder: Euboea (Beck), Nauplia (Berger), “Napoli di Romaniaˮ (Herb. Zuccarini). Auch in Palästina: Saida (Gaillardot n. 5), Damar (Gaillardot n. 2082), Ghezir (Gaillardot n. 2996) [from protologue]

---

*Cynoglossum creticum* var. *siculum* (Guss.) Brand in Engler, Pflanzenr. 78 (IV.252): 131. 1921

- IPNI: absent (2014-11-05)
- ASSESSMENT: [*Cynoglossum creticum* Mill.]
- STATUS: comb. & stat. nov. [species to variety]
- REFERENCE: Link
- BASIS: Basionym: *Cynoglossum siculum* Guss.

---

*Cynoglossum crista-galli* (Rech. f. & Riedl) Greuter & Stier in Biodivers. Data J. [hoc loco]. 2015

- ASSESSMENT: accepted
- STATUS: comb. nov.
- BASIS: Basionym: *Mattiastrum crista-galli* Rech. f. & Riedl

---

*Cynoglossum cristatum* of Lamarck, Encycl. 2: 238. 1786

- IPNI: absent (2014-11-05)
- STATUS: [isonym]
- REFERENCE: Link
- COMMENTS: Even though Lamarck does not mention Schreber’s *Cynoglossum cristatum* explicitly, the species he so names comprises the two very same elements: Tournefort’s plant from the Orient and, as an unnamed var. β, Colonna’s Italian one.

---

*Cynoglossum cristatum* Schreb. in Nova Acta Phys.-Med. Acad. Caes. Leop.-Carol. Nat. Cur. 3: 476. 1767

- IPNI: Cynoglossum cristatum Schreb. -- Nova Acta Phys.-Med. Acad. Caes. Leop.-Carol. Nat. Cur. 3: 476. 1767; Lam. Tabl. Encyc. 1: 400. (IK)
- ASSESSMENT: accepted
- STATUS: spec. nov.
- REFERENCE: Link
- SYNONYMY:   
  ≡ *Omphalodes cristata*  (Schreb.) Schrank 1812
    
  ≡ *Rindera cristata* (Schreb.) Roem. & Schult. 1819
    
  ≡ *Mattia cristata* (Schreb.) G. Don 1838
    
  ≡ *Paracaryum cristatum* (Schreb.) Boiss. 1856
    
  ≡ *Mattiastrum cristatum* (Schreb.) Brand 1915
- BASIS: Original material: In montibus Vulvensium, prope oppidum Anversan, inque Oriente locis saxosis apricis … Column. Ecphras. I. p. 177, t. 178.b. … Moris. Hist. 3. p. 449. S. II. t. 30. f. 7. ex Columna … *Cynoglossum orientale, buglossi folio fructo umbilicato, cristato,* Tournef. cor. 7 Original specimen: M (M0174163\*)
- COMMENTS: Schreber’s species comprised two discordant elements: Tournefortʼs plant from the Orient of which Schreber had a specimen, presumably through Gundelsheimer, and the plant from Anversa degli Abruzzi illustrated by Colonna, which subsequently became the basis of *Cynoglossum columnae* Ten. (1811). In conformity with generally accepted practice, the former is to be regarded as the type.

---

*Cynoglossum cristatum* of Aucher [Candolle, Prodr. 10: 160. 1846]

- IPNI: Cynoglossum cristatum Aucher ex DC. -- Prodr. [A. P. de Candolle] 10: 160. 1846 [8 Apr 1846] (IK)
- STATUS: nom. inval. [pro syn. sub *Omphalodes rugulosa*]
- REFERENCE: Link

---

*Cynoglossum cristatum* subsp. *carduchorum* (R. R. Mill) Greuter & Burdet in Willdenowia 11: 34. 1981

- IPNI: Cynoglossum cristatum Aucher ex DC. subsp. carduchorum (R.R.Mill) Greuter & Burdet -- Willdenowia 11(1): 34. 1981 (IK)
- ASSESSMENT: accepted
- STATUS: comb. nov.
- REFERENCE: Link
- BASIS: Basionym: *Paracaryum cristatum* subsp. *carduchorum* R. R. Mill

---

*Cynoglossum cristulatum* (Lipsky) Greuter & Stier in Biodivers. Data J. [hoc loco]. 2015

- ASSESSMENT: accepted
- STATUS: comb. nov.
- BASIS: Basionym: *Rindera cristulata* Lipsky

---

*Cynoglossum cyaneum* of gardeners, ascribed to Hooker [in Gard. Chron. 1858: 240. 1858]

- IPNI: Cynoglossum cyaneum Hort. ex Hook.f. -- Gard. Chron. (1858) 240. (IK)
- STATUS: no name [ICN Art. 35.2]
- REFERENCE: Link
- COMMENTS: Provisional epithet mentioned in the protologue of *Cynoglossum nobile* Hook. f..

---

*Cynoglossum cyclhymenium* (Boiss.) Greuter & Stier in Biodivers. Data J. [hoc loco]. 2015

- ASSESSMENT: accepted
- STATUS: comb. nov.
- BASIS: Basionym: *Paracaryum rugulosum*  var. *cyclhymenium* Boiss.

---

*Cynoglossum cynoglossoides* (Rech. f. & Riedl) Greuter & Stier in Biodivers. Data J. [hoc loco]. 2015

- ASSESSMENT: accepted
- STATUS: comb. nov.
- BASIS: Basionym: *Mattiastrum cynoglossoides* Rech. f. & Riedl

---

*Cynoglossum decipiens* Lojac., Fl. Sicul. 2(2): 89. 1907

- IPNI: Cynoglossum decipiens Lojac. -- Fl. Sicul. (Lojacono) 2(2): 89. 1907 (IK)
- ASSESSMENT: [*Cynoglossum cheirifolium* L.]
- STATUS: spec. nov.
- REFERENCE: Link
- BASIS: Original material: none indicated in protologue

---

*Cynoglossum decumbens* of Gueldenstedt, Reis. Russland 1: 190 & 2: 343. 1787

- IPNI: absent (2014-11-05)
- STATUS: nom. inval. [nom. nud.]
- REFERENCE: Link

---

*Cynoglossum decumbens* of Gueldenstedt [Ledeb., Fl. Ross. 3: 167. 1847]

- IPNI: Cynoglossum decumbens Gueldenst. ex Ledeb. -- Fl. Ross. (Ledeb.) 3(1,8): 167. 1847 [Oct 1847] (IK)
- STATUS: nom. inval. [nom. nud.]
- REFERENCE: Link

---

*Cynoglossum decurrens* Ruiz & Pav., Fl. Peruv. 2: 6. 1799

- IPNI: Cynoglossum decurrens Ruiz & Pav. -- Fl. Peruv. [Ruiz & Pavon] 2: 6. 1799 (IK)
- ASSESSMENT: Not *Cynoglossum* s. l.; genus uncertain [*“Cynoglossum” limense* Willd.]
- STATUS: spec. nov.
- REFERENCE: Link
- SYNONYMY:   
  ≡ *Cynoglossum alatum* Molina 1810, nom. illeg.
- BASIS: Original material: in nemoribus Conceptionis Chile ad Gavilan paludem et Palomares tractus [from protologue]

---

*Cynoglossum decurrens* var. *limense* (Willd.) DC., Prodr. 10: 153. 1846

- IPNI: Cynoglossum decurrens var. limense (Willd.) DC. -- Prodr. [A. P. de Candolle] 10: 153. 1846 [8 Apr 1846] (GCI)
- ASSESSMENT: Not *Cynoglossum* s. l.; genus uncertain [*“Cynoglossum” limense* Willd.]
- STATUS: comb. & stat. nov. [species to variety]
- REFERENCE: Link
- BASIS: Basionym: *Cynoglossum limense* Willd.
- COMMENTS: Name legitimate but combination inappropriate under the rules of priority.

---

*Cynoglossum deflexum* (Wahlenb.) Roth, Enum. Pl. Phaen. Germ. 1(1): 589. 1827

- IPNI: Cynoglossum deflexum Roth -- Enum. Pl. Phan. Germ. i. I. 589. (IK)
- ASSESSMENT: *Hackelia deflexa*  (Wahlenb.) Opiz
- STATUS: comb. nov.
- REFERENCE: Link
- BASIS: Basionym: *Myosotis deflexa*  Wahlenb.

---

*Cynoglossum densefoliatum* Chiov. in Ann. Bot. (Roma) 9: 82. 1911

- IPNI: Cynoglossum densefoliatum Chiov. -- Ann. Bot. (Rome) 9: 82. 1911 (IK)
- ASSESSMENT: Not *Cynoglossum* s. l.; genus uncertain [*“Cynoglossum” coeruleum* Hochst. ex A. DC.]
- STATUS: spec. nov.
- REFERENCE: Link
- BASIS: Original material: Dembià: Gondar pascoli aprici lungo il fiume Cahà presso la chiesa di Fasìl Odòs 7, X 1909 (Chiovenda n. 2411) [from protologue]. Type in FI-W

---

*Cynoglossum densum* (Rech. f. & Riedl) Greuter & Stier in Biodivers. Data J. [hoc loco]. 2015

- ASSESSMENT: accepted
- STATUS: comb. nov.
- BASIS: Basionym: *Mattiastrum densum* Rech. f. & Riedl

---

*Cynoglossum denticulatum* A. DC. in Candolle, Prodr. 10: 150. 1846

- IPNI: Cynoglossum denticulatum A.DC. -- Prodr. [A. P. de Candolle] 10: 150. 1846 [8 Apr 1846] (IK)
- ASSESSMENT: [*Cynoglossum wallichii* G. Don]
- STATUS: spec. nov.
- REFERENCE: Link
- SYNONYMY:   
  ≡ *Paracynoglossum denticulatum* (A. DC.) Popov 1953
- BASIS: Original material: in Emodi apricis inter 4 et 10 000 ped. alt. (v. s. a cl. Edgew[orth] comm. sub n. 390 partim, 391, 392) [from protologue]. Syntypes: G-DC (G00205521\*, G00205522\*, G00205523\*). Lectotype (to be designated elsewhere by König et al.): [India], Simla, Sept. 1834, *Edgeworth 390* partim, G-DC (G00205522\*)

---

*Cynoglossum denticulatum* var. *ramosius* A. DC. in Candolle, Prodr. 10: 150. 1846

- IPNI: absent (2014-11-05)
- ASSESSMENT: [*Cynoglossum wallichii* G. Don]
- STATUS: var. nov.
- REFERENCE: Link
- BASIS: Original material: Ejusd. [Edgeworth] N. 390 partim [in Emodi apricis inter 4 et 10 000 ped. alt.] [from protologue]. Holotype: G-DC (G00205540\*)

---

*Cynoglossum denticulatum* var. *zeylanicum* (Lehm.) Clarke in Hooker, Fl. Brit. India 4: 157. 1883

- IPNI: absent (2014-11-05)
- ASSESSMENT: *Cynoglossum zeylanicum* (Lehm.) Brand
- STATUS: comb. & stat. nov. [species to variety]
- REFERENCE: Link
- BASIS: Basionym: *Myosotis zeylanica*  Lehm.; Replaced synonym: *Anchusa zeylanica* Vahl ex Hornem., non *Anchusa zeylanica* J. Jacq.
- COMMENTS: Name legitimate but combination inappropriate under the rules of priority.

---

*Cynoglossum dielsii* (Bornm.) Greuter & Stier in Biodivers. Data J. [hoc loco]. 2015

- ASSESSMENT: accepted
- STATUS: comb. nov.
- BASIS: Basionym: *Mattiastrum dielsii* Bornm.

---

*Cynoglossum dieterlei* (F. Sadat) Greuter & Stier in Biodivers. Data J. [hoc loco]. 2015

- ASSESSMENT: accepted
- STATUS: comb. nov.
- BASIS: Basionym: *Mattiastrum dieterlei* F. Sadat

---

*Cynoglossum diffusum* of Roxburgh, Hort. Bengal.: 13. 1814

- IPNI: Cynoglossum diffusum Roxb. -- Hort. Bengal. [13]; Fl. Ind. i. 4. (IK)
- STATUS: nom. inval. [nom. nud.]
- REFERENCE: Link

---

*Cynoglossum diffusum* Roxb. ex Lehm., Pl. Asperif. Nucif.: 140. 1818

- IPNI: Cynoglossum diffusum Roxb. -- Hort. Bengal. 13 (1814), nomen; Lehm. Asperifol. 140 (1818), descr. (IK)
- ASSESSMENT: *Bothriospermum zeylanicum* (J. Jacq.) Druce
- STATUS: spec. nov.
- REFERENCE: Link
- BASIS: Original material: Hornemann … specimina mecum communicavit. Habitat in Benghalia [from protologue]. Holotype presumably at MEL

---

*Cynoglossum diffusum* of Roxburgh, Fl. Ind. 2: 7. 1824

- IPNI: Cynoglossum diffusum Roxb. -- Hort. Bengal. [13]; Fl. Ind. i. 4. (IK)
- STATUS: [isonym]
- REFERENCE: Link

---

*Cynoglossum dioscoridis* Vill., Prosp. Hist. Pl. Dauphiné: 21. 1779

- IPNI: Cynoglossum dioscoridis Vill. -- Prosp. Hist. Pl. Dauphiné 21. 1779 [16 Apr 1779] ; Hist. Pl. Dauphiné 2: 457 (1787) (IK)
- ASSESSMENT: accepted
- STATUS: spec. nov.
- REFERENCE: Link
- BASIS: Original material not specified in protologue. Lectotype (Selvi & Sutorý in Pl. Biosyst. 146: 477. 2012): [France, rég. Rhone-Alps, Dép. Isère, distr. Chartreuse] à St Eynard, dans le Champsaur, GRE (GRE-1837.27682)

---

*Cynoglossum dioscoridis* of Ledebour, Fl. Ross. 3: 166. 1847

- IPNI: Cynoglossum dioscoridis Ledeb. -- Fl. Ross. (Ledeb.) 3(1,8): 166. 1847 [Oct 1847] (IK)
- STATUS: [isonym]
- REFERENCE: Link

---

*Cynoglossum dioscoridis* var. *castrilense* (Pau) Brand in Engler, Pflanzenr. 78 (IV.252): 119. 1921

- IPNI: absent (2014-11-05)
- ASSESSMENT: [*Cynoglossum pustulatum* Boiss.]
- STATUS: comb. & stat. nov. [species to variety]
- REFERENCE: Link
- BASIS: Basionym: *Cynoglossum castrilense* Pau

---

*Cynoglossum dioscoridis* var. *maroccanum* (Brand) Maire in Bull. Soc. Hist. Nat. Afrique N. 22: 56. 1931

- IPNI: absent (2014-11-05)
- ASSESSMENT: [*Cynoglossum dioscoridis* Vill.]
- STATUS: comb. nov.
- REFERENCE: Link
- BASIS: Basionym: *Cynoglossum montanum* var. *maroccanum* Brand

---

*Cynoglossum dioscoridis* var. *nebrodense* (Guss.) Ball in J. Linn. Soc. London16: 569. 1878

- IPNI: absent (2014-11-05)
- ASSESSMENT: *Cynoglossum nebrodense* Guss.
- STATUS: comb. & stat. nov. [species to variety]
- REFERENCE: Link
- BASIS: Basionym: *Cynoglossum nebrodense* Guss.
- COMMENTS: According to Brand (in Engler, Pflanzenr. 78 (IV.252): 128. 1921) a misapplied name, the cited specimens are the basis of *Cynoglossum montanum* var. *maroccanum* Brand.

---

*Cynoglossum discolor* Baker in J. Linn. Soc., Bot. 20: 212. 1883

- IPNI: Cynoglossum discolor Baker -- J. Linn. Soc., Bot. 20: 212. 1883 [1884 publ. 1883] (IK)
- ASSESSMENT: [*Cynoglossum cernuum* Baker]
- STATUS: spec. nov.
- REFERENCE: Link
- BASIS: Original material: Central Madagascar, Baron 1828 [from protologue]. Syntypes: K (K000418911\*), P (P00417684\*, P00417685\*)

---

*Cynoglossum divaricatum* Steph. ex Lehm., Pl. Asperif. Nucif.: 161. 1818

- IPNI: Cynoglossum divaricatum Steph. ex Lehm. -- Pl. Asperif. Nucif. 1: 161. 1818 [Jul-early Sep 1818] (IK)
- ASSESSMENT: accepted
- STATUS: spec. nov.
- REFERENCE: Link
- BASIS: Original material: in Sibiria (v. s.) [from protologue]. Holotype perhaps in MEL

---

*Cynoglossum drummondii* Benth., Fl. Austral. 4: 409. 1868

- IPNI: Cynoglossum drummondii Benth. -- Fl. Austral. 4: 409. 1868 [16 Dec 1868] (IK)
- ASSESSMENT: [*Cynoglossum australe* R. Br.]
- STATUS: spec. nov.
- REFERENCE: Link
- SYNONYMY:   
  ≡ *Cynoglossum australe* var. *drummondii* (Benth.) Brand 1921
- BASIS: Original material: S. Australia. Mount Remarkable, F. Mueller ; Mount Searle, Warburton (with large flowers). W. Australia, Drummond, n. 504 (with small flowers) [from protologue]. Syntype: Drummond 504, BM (BM001040565\*)

---

*Cynoglossum dubium* of Hochstetter [Brand in Engler, Pflanzenr. 78 (IV.252): 146. 1921]

- IPNI: Cynoglossum dubium Hochst. ex Brand -- Pflanzenr. (Engler) Borrag. Cynogloss. 146, in syn. 1921 (IK)
- STATUS: nom. inval. [pro syn. sub *Cynoglossum coeruleum*]
- REFERENCE: Link

---

*Cynoglossum dubium* (Fisch. & C. A. Mey.) Greuter & Stier in Biodivers. Data J. [hoc loco]. 2015

- ASSESSMENT: accepted
- STATUS: comb. nov.
- BASIS: Basionym: *Solenanthus dubius* Fisch. & C. A. Mey.

---

*Cynoglossum dumanii* (Aytaç & R. R. Mill) Greuter & Stier in Biodivers. Data J. [hoc loco]. 2015

- ASSESSMENT: accepted
- STATUS: comb. nov.
- BASIS: Basionym: *Rindera dumanii* Aytaç & R. R. Mill

---

*Cynoglossum dunnianum* Diels in Notes Roy. Bot. Gard. Edinburgh 5: 168. 1912

- IPNI: Cynoglossum dunnianum Diels -- Notes Roy. Bot. Gard. Edinburgh 5: 168. 1912 (IK)
- ASSESSMENT: *Antiotrema dunnianum* (Diels) Hand.-Mazz.
- STATUS: spec. nov.
- REFERENCE: Link
- SYNONYMY:   
  ≡ *Antiotrema dunnianum* (Diels) Hand.-Mazz. 1920
- BASIS: SYNTYPES: Original material: hills at south end of the Lang-Kong valley, near the village of Kai-hi-dsi. Lat. 26° 25' N. Alt. 7500 ft. April 1906.; G. Forrest. No. 2004. Along the base of the eastern flank of the Tali Range. Lat. 25° 40' N. Alt. 7-8000 ft. April June 1906.“ G. Forrest. No. 4476. Also Mengtse (Hancock No. 22) and very similar Henry 10600, 10600A [from protologue]. Syntype: Forrest 2004, P (P00622857\*)

---

*Cynoglossum echinatum* Thunb., Prodr. Pl. Cap.: 34. 1794

- IPNI: Cynoglossum echinatum Thunb. -- Prodr. Pl. Cap. 1: 34. 1794 (IK)
- ASSESSMENT: [*Lappula cynoglossoides*  (Lam.) Gürke (?)]
- STATUS: spec. nov.
- REFERENCE: Link
- BASIS: Original material not mentioned in protologue. Original specimens: LD (LD1259484\*), SBT (SBT12941 \*)

---

*Cynoglossum edgeworthii* A. DC. in Candolle, Prodr. 10: 150. 1846

- IPNI: Cynoglossum edgeworthii A.DC. -- Prodr. [A. P. de Candolle] 10: 150. 1846 [8 Apr 1846] (IK)
- ASSESSMENT: [*Cynoglossum furcatum* Wall.]
- STATUS: spec. nov.
- REFERENCE: Link
- BASIS: Original material: in apricis Emodi, inter 4 et 6000 ped. alt. (v. s. comm.. a cl. Edgew(orth) sub n. 400 [from protologue]. Holotype: G-DC (G00205524\*)

---

*Cynoglossum elongatum* Hornem., Hort. Bot. Hafn. 2: 956. 1815

- IPNI: Cynoglossum elongatum Hornem. -- Hort. Bot. Hafn. ii. 956. 1815 (IK)
- ASSESSMENT: [*Cynoglossum dioscoridis* Vill.]
- STATUS: spec. nov.
- REFERENCE: Link
- BASIS: Original material: D [grown out-of-doors] intr. 1813; [sub] Cynogl. lanceolatum Forssk. nomine a celeberr. Schradero missum fuit [from protologue]. Probable type: C (C10013450\*)

---

*Cynoglossum emarginatum* Lam., Tabl. Encycl. 1: 400. 1792

- IPNI: Cynoglossum emarginatum Lam. -- Tabl. Encycl. i. 400. (IK)
- ASSESSMENT: [*Cynoglossum racemosum* Schreb.]
- STATUS: spec. nov.
- REFERENCE: Link
- SYNONYMY:   
  ≡ *Mattia emarginata* (Lam.) Roem. & Schult. 1819
- BASIS: Original material: Ex Oriente. *Cynoglossum orientale minus, flore campanulato caeruleo* Tournef. Cor. 7 [from protologue]. Holotype: presumably P-TRF
- COMMENTS: Tournefort’s phrase name from the Corollarium (1707: 7) is cited in the protologue of no less than three *Cynoglossum* names: *Cynoglossum racemosum* Schreb. (1767), *Cynoglossum emarginatum* Lam. (1791) and *Cynoglossum angustifolium* Willd. (1798). The three specimens used by the authors of these names were likely duplicates of a single gathering made by Tournefort and his companion Gundelsheimer during their travel through the Orient. However, as none of these names were typified at that time, and the later authors did not cite an earlier name in synonymy, the three names are heterotypic, and legitimate.

---

*Cynoglossum emodi* of Schouw in Neue Allg. Deutsche Garten- Blumenzeitung 6: 352. 1850

- IPNI: Cynoglossum emodi Schouw -- Ind. Sem. Hafn. Coll. (1846) 4; ex Lehm. in Hamb. Gartenz. vi. (1850) 352. (IK)
- STATUS: nom. inval. [pro syn. sub *Lindelofia anchusoides* (Lindl.) Lehm.]
- REFERENCE: Link

---

*Cynoglossum emodum* of Schouw, Index Sem. Hort. Haun. 1846: 4. 1847

- IPNI: Cynoglossum emodi Schouw -- Ind. Sem. Hafn. Coll. (1846) 4; ex Lehm. in Hamb. Gartenz. vi. (1850) 352. (IK)
- STATUS: nom. inval. [nom. nud.]
- REFERENCE: Link

---

*Cynoglossum emodum* of Schouw in Linnaea 24: 160. 1851

- IPNI: Cynoglossum emodum Schouw -- Linnaea 24: 160. 1851 (IK)
- STATUS: nom. inval. [nom. nud.]
- REFERENCE: Link

---

*Cynoglossum enerve* ascribed to Turczaninov [in Bull. Soc. Imp. Naturalistes Moscou 20: 259. 1840]

- IPNI: Cynoglossum enerve Turcz. -- Bull. Soc. Imp. Naturalistes Moscou (1840) 259. (IK)
- STATUS: no name [ICN Art. 35.2]
- REFERENCE: Link
- COMMENTS: Turczaninov describes Drège specimens under the label name *Echinospermum enerve* E. Mey., a name that he does not accept as he refers the plants to *Cynoglossum*; nor does he make the combination under the latter generic name, as has often been assumed.

---

*Cynoglossum enerve* (E. Mey. ex Drège) Turcz. ex B. D. Jacks., Index Kew. 1: 687. 1893

- IPNI: absent (2014-11-05)
- ASSESSMENT: [*Cynoglossum hispidum* Thunb.]
- STATUS: comb. nov.
- REFERENCE: Link
- BASIS: Basionym: *Echinospermum enerve*  E. Mey. ex Drège
- COMMENTS: There may have been earlier validations of this combination, but none that we could find.

---

*Cynoglossum erectum* Schweigg. ex Schrank in Denkschr. Königl.-Baier. Bot. Ges. Regensburg 2: 29. 1822

- IPNI: Cynoglossum erectum Schweigg. ex Schrank -- in Denkschr. Bot. Ges. Regensb. ii. (1822) 29. (IK)
- ASSESSMENT: [*Hackelia revoluta*  (Ruiz & Pav.) I. M. Johnst. (?)]
- STATUS: spec. nov.
- REFERENCE: Link
- BASIS: Original material: im Cap-Hause … des K. botanischen Gartens zu München [from protologue]

---

*Cynoglossum erectum* L. C. Higgins in Phytologia 33: 411. 1976

- IPNI: Cynoglossum erectum L.C.Higgins -- Phytologia 33(6): 411. 1976 (IK)
- ASSESSMENT: Not *Cynoglossum* s. l.; genus uncertain [*“Cynoglossum” henricksonii* L. C. Higgins]
- STATUS: nom. illeg. [homonym]
- REFERENCE: Link
- SYNONYMY:   
  ≡ *Cynoglossum henricksonii* L. C. Higgins 1976
- BASIS: Holotype: Mexico: Zacatecas, 10 km west northwestof Tecolotes, on road to Coapa. Latitude 24°39’N longitude 102°02’W; altitude 1800 meters; 17 June 1972. F. Chiang, T. Wendt and M. C. Johnston 7890. Holotype deposited at (TEX) [from protologue]. Isotype: WTS

---

*Cynoglossum eriocalycinum* (Boiss. & Buhse) Greuter & Stier in Biodivers. Data J. [hoc loco]. 2015

- ASSESSMENT: accepted
- STATUS: comb. nov.
- BASIS: Basionym: *Cynoglossum eriocalycinum* (Boiss. & Buhse) Greuter & Stier

---

*Cynoglossum erysimifolium* (Boiss.) Greuter & Burdet in Willdenowia 11: 34. 1981

- IPNI: Cynoglossum erysimifolium (Boiss.) Greuter & Burdet -- Willdenowia 11(1): 34. 1981 (IK)
- ASSESSMENT: accepted
- STATUS: comb. nov.
- REFERENCE: Link
- BASIS: Basionym: *Paracaryum erysimifolium* Boiss.

---

*Cynoglossum erythraeum* (Brand) Riedl in Oesterr. Bot. Z. 119: 71. 1971

- IPNI: Cynoglossum erythraeum (Brand) Riedl -- Oesterr. Bot. Z. 119(1-3): 71. 1971 (IK)
- ASSESSMENT: Not *Cynoglossum* s. l.; *Microparacaryum* sp. [required combination not yet published]
- STATUS: comb. nov.
- REFERENCE: Link
- BASIS: Basionym: *Adelocaryum erythraeum* Brand

---

*Cynoglossum ferganicum* (Popov) Greuter & Stier in Biodivers. Data J. [hoc loco]. 2015

- ASSESSMENT: accepted
- STATUS: comb. nov.
- BASIS: Basionym: *Rindera ferganica* Popov

---

*Cynoglossum fiebrigii* K. Krause in Bot. Jahrb. Syst. 37: 634. 1906

- IPNI: Cynoglossum fiebrigii K.Krause -- Bot. Jahrb. Syst. 37(5): 634. 1906 [30 Oct 1906] (GCI)
- ASSESSMENT: [*Hackelia revoluta*  (Ruiz & Pav.) I. M. Johnst.]
- STATUS: spec. nov.
- REFERENCE: Link
- BASIS: Original material: Bolivia australis: Prope Calderillo, in saxosis, 3300 m s. m. (Fiebrig n. 3214) [from protologue]

---

*Cynoglossum flaviflorum* (Rech. f. & Riedl) Greuter & Stier in Biodivers. Data J. [hoc loco]. 2015

- ASSESSMENT: accepted
- STATUS: comb. nov.
- BASIS: Basionym: *Mattiastrum flaviflorum* Rech. f. & Riedl

---

*Cynoglossum flexuosum* of Lehmann [Brand in Engler, Pflanzenr. 78 (IV.252): 137. 1921]

- IPNI: Cynoglossum flexuosum Lehm. ex Brand -- Pflanzenr. (Engler) Borrag. Cynogloss. 137, in syn. 1921 (IK)
- STATUS: nom. inval. [pro syn. sub *Cynoglossum lanceolatum*]
- REFERENCE: Link

---

*Cynoglossum flexuosum* of Kazmi in J. Arnold Arbor. 52: 352. 1971 [Apr]

- IPNI: Cynoglossum flexuosum (Brand) Kazmi -- J. Arnold Arbor. 52 (2): 352 (Apr. 1971). (IK)
- STATUS: [isonym]
- REFERENCE: Link

---

*Cynoglossum flexuosum* (Brand) Riedl in Oesterr. Bot. Z. 119: 71. 1971 [Feb]

- IPNI: Cynoglossum flexuosum (Brand) Riedl -- Oesterr. Bot. Z. 119(1-3): 71. 1971 (IK)
- ASSESSMENT: accepted
- STATUS: comb. nov.
- REFERENCE: Link
- BASIS: Basionym: *Adelocaryum flexuosum* Brand

---

*Cynoglossum foetens* of Gilibert, Fl. Lit. Inch. 1: 22. 1782 *(‘faetens’)*

- IPNI: Cynoglossum foetens Gilib. -- Fl. Lit. Inch. i. 22. 1782 (IK)
- STATUS: nom. inval. [suppressed work, ICN Art. 34.1 and App. VI]
- REFERENCE: Link

---

*Cynoglossum foliosum* (Paine) Greuter & Burdet in Willdenowia 11: 34. 1981

- IPNI: Cynoglossum foliosum (Paine) Greuter & Burdet -- Willdenowia 11(1): 34. 1981 (IK)
- ASSESSMENT: accepted
- STATUS: comb. nov.
- REFERENCE: Link
- BASIS: Basionym: *Cerinthopsis foliosa* Paine

---

*Cynoglossum formosanum* Nakai in Bot. Mag. (Tokyo) 37: 4. 1923

- IPNI: Cynoglossum formosanum Nakai -- Bot. Mag. (Tokyo) 1923, xxxvii. 4. (IK)
- ASSESSMENT: Not *Cynoglossum* s. l.; genus uncertain (species accepted)
- STATUS: spec. nov.
- REFERENCE: Link
- BASIS: Original material: Liukiu: in insula Okinawa (Y. Tashiro), ibidem (J. Matsumura). Formosa: Kagi (T.Kawakami et Mori n. 1746). Funsin (Honda n. 5), Shintsiku (T. Makino) [from protologue]

---

*Cynoglossum formosanum* f. *albiflorum* Masam. in J. Soc. Trop. Agric. 2: 152. 1930

- IPNI: Cynoglossum formosanum Nakai f. albiflorum Masam. -- J. Soc. Trop. Agric. 2(2): 152. 1930 [30 Oct 1930]
- ASSESSMENT: Not *Cynoglossum* s. l.; genus uncertain [*“Cynoglossum” formosanum* Nakai]
- STATUS: f. nov.
- REFERENCE: Link
- SYNONYMY:   
  ≡ *Cynoglossum lanceolatum* f. *albiflorum* (Masam.) Yonek. 2005
- BASIS: Original material: Formosa: Sikayo, Tosei-gun, Taichu-shu – Masamune s.n. Jul. 9, 1930 [from protologue]

---

*Cynoglossum formosum* (R. R. Mill) Greuter & Burdet in Willdenowia 11: 35. 1981

- IPNI: Cynoglossum formosum (R.R.Mill) Greuter & Burdet -- Willdenowia 11(1): 35. 1981 (IK)
- ASSESSMENT: accepted
- STATUS: comb. nov.
- REFERENCE: Link
- BASIS: Basionym: *Solenanthus formosus* R. R. Mill

---

*Cynoglossum fornicatum* (Pazij) Greuter & Stier in Biodivers. Data J. [hoc loco]. 2015

- ASSESSMENT: accepted
- STATUS: comb. nov.
- BASIS: Basionym: *Rindera fornicata* Pazij

---

*Cynoglossum fulvum* Rudolphi in J. Bot. (Schrader) 1799: 279. 1800

- IPNI: Cynoglossum fulvum Rudolphi -- J. Bot. (Schrader) 1799(2): 280 (279). 1800 [1799 publ. Apr. 1800] (IK)
- ASSESSMENT: [*Cynoglossum clandestinum* Desf.]
- STATUS: spec. nov.
- REFERENCE: Link
- BASIS: Original material: von meinem verstorbenen Freund Teede … bei Lissabon gesammelt [from protologue]

---

*Cynoglossum furcatum* Wall. in Roxburgh, Fl. Ind. 2: 6. 1824

- IPNI: Cynoglossum furcatum Wall. -- Roxb. Fl. Ind., ed. Carey & Wall. ii. 6 (1824). (IK)
- ASSESSMENT: accepted
- STATUS: spec. nov.
- REFERENCE: Link
- BASIS: Original material: A native of Nepala, and from thence introduced in 1818 into the Bontanic Garden where it blossoms during the rainy season [from protologue]. Lectotype (Verdcourt in Kew Bull. 43: 347. 1988, as “holotype”): Wallich 919.2, herb. Wallich, K; isotypes: A (A00096679\*), BM (BM001124577\*), E (E00288392\*) G-DC (G00205533\*)
- COMMENTS: The initials “N.W.” at the end of the last paragraph of the description on page 7 demonstrate Wallich’s authorship (see also TL-2 #9724, Note).

---

*Cynoglossum furcatum* f. *albiflorum* (H. Hara) Yonek. in J. Jap. Bot. 80: 329. 2005

- IPNI: Cynoglossum furcatum Wall. f. albiflorum (H.Hara) Yonek. -- J. Jap. Bot. 80(6): 329. 2005 [Dec 2005]
- ASSESSMENT: [*Cynoglossum furcatum* Wall.]
- STATUS: comb. nov.
- REFERENCE: Link
- BASIS: Basionym: *Cynoglossum zeylanicum* f. *albiflorum* H. Hara

---

*Cynoglossum furcatum* var. *lanceolatum* C. B. Clarke in Hooker, Fl. Brit. India 4: 156. 1883

- IPNI: absent (2014-11-05)
- ASSESSMENT: [*Cynoglossum furcatum* Wall.]
- STATUS: nom. nov.
- REFERENCE: Link
- BASIS: Replaced synonym (see Verdcourt in Kew Bull. 43: 347. 1988): *Cynoglossum heynei* G. Don

---

*Cynoglossum furcatum* var. *tenerum* Royle ex Benth. in Royle, Ill. Bot. Himal. Mts.: 306. 1836

- IPNI: absent (2014-11-05)
- ASSESSMENT: [*Cynoglossum furcatum* Wall.]
- STATUS: var. nov.
- REFERENCE: Link
- BASIS: Original material: Mussooree [from protologue]

---

*Cynoglossum furcatum* var. *villosulum* (Nakai) Riedl in Novon 4: 46. 1994

- IPNI: Cynoglossum furcatum Wall. var. villosulum (Nakai) Riedl -- Novon 4(1): 46. 1994 (IK)
- ASSESSMENT: [*Cynoglossum furcatum* Wall.]
- STATUS: comb. & stat. nov. [species to variety]
- REFERENCE: Link
- BASIS: Basionym: *Cynoglossum villosulum* Nakai

---

*Cynoglossum furcatus* Wall. in Roxburgh, Fl. Ind. 2: 6. 1824

- IPNI: Cynoglossum furcatus Wall. -- in Roxb. Fl. Ind. ed. Carey ii. 6. (IK)
- STATUS: wrong epithet citation by IPNI [*furcatus* instead of *furcatum*]
- REFERENCE: Link

---

*Cynoglossum gansuense* Y. L. Liu in Acta Phytotax. Sin. 19: 519. 1981

- IPNI: Cynoglossum gansuense Y.L.Liu -- Acta Phytotax. Sin. 19(4): 519 (1981). (IK)
- ASSESSMENT: accepted
- STATUS: spec. nov.
- REFERENCE: Link
- BASIS: Holotype: Qinghai: Huangyuan, VII. 1957, P. C. Tsoong 8882 (typus in Herb. Inst. Bot. Acad. Sin. conserv.)

---

*Cynoglossum geometricum* Baker & C. H. Wright in Thiselton-Dyer, Fl. Trop. Afr. 4(2, 1): 52. 1905

- IPNI: Cynoglossum geometricum Baker & C.H.Wright -- Fl. Trop. Afr. [Oliver et al.] 4(2.1): 52. 1905 [Dec 1905] (IK)
- ASSESSMENT: Not *Cynoglossum* s. l.; genus uncertain (*“Cynoglossum” lanceolatum* subsp. *geometricum* (Baker & C. H. Wright) Brand)
- STATUS: spec. nov.
- REFERENCE: Link
- SYNONYMY:   
  ≡ *Cynoglossum lanceolatum* subsp. *geometricum* (Baker & C. H. Wright) Brand 1921
    
  ≡ *Paracynoglossum geometricum* (Baker & C. H. Wright) R. R. Mill 1984
- BASIS: Original material: Nile Land. Uganda: Ruwenzori,7000 ft., Doggett! British East Africa: between Eldama Ravine and Mau Plateau, 7000--8000 ft., Whyte!; Mozamb. Dist. British Central Africa: Nyasaland; Zomba, Purves, 1901! Mount Chiradzulu, Whyte! and without precise locality, Buchanan, 1145 [from protologue]. Lectotype (Hilliard & Burtt in Notes Roy. Bot. Gard. Edinburgh 43: 348. 1986): Malawi, Mt. Chiradzulu, Whyte, K (K000418916)

---

*Cynoglossum germanicum* Jacq., Observ. Bot. 2: 31. 1767

- IPNI: Cynoglossum germanicum Jacq. -- Observ. Bot. [Jacquin] ii. 31. 1767 [Apr 1767] (IK)
- ASSESSMENT: accepted
- STATUS: spec. nov.
- REFERENCE: Link
- BASIS: Original material: in eodem agro [Vindobonensi]. Idem Nordheimi carptum clarissimus mecum Willigius communicavit [from protologue]

---

*Cynoglossum germanicum* var. *hungaricum* (Simonk.) Brand in Engler, Pflanzenr. 78 (IV.252): 125. 1921

- IPNI: absent (2014-11-05)
- ASSESSMENT: [*Cynoglossum montanum* L.]
- STATUS: comb. & stat. nov. [species to variety]
- REFERENCE: Link
- BASIS: Basionym: *Cynoglossum hungaricum* Simonk.

---

*Cynoglossum germanicum* subsp. *pellucidum* (Lapeyr.) Sutorý in Čas. Morav. Mus. Brne, Vědy Přír. 73: 150. 1988

- IPNI: Cynoglossum germanicum Jacq. subsp. pellucidum (Lapeyr.) Sutorý -- Čas. Morav. Muz., Vědy Přír. 73(1–2): 150 (1988):. (IK)
- ASSESSMENT: accepted
- STATUS: comb. & stat. nov. [species to subspecies]
- REFERENCE: Link
- BASIS: Basionym: *Cynoglossum pellucidum* Lapeyr.

---

*Cynoglossum germanicum* subsp. *rotundum* Sutorý in Čas. Morav. Mus. Brne, Vědy Přír. 73: 150. 1988

- IPNI: Cynoglossum germanicum Jacq. subsp. rotundum Sutorý -- Čas. Morav. Muz., Vědy Přír. 73(1–2): 150 (1988). (IK)
- ASSESSMENT: accepted
- STATUS: subsp. nov.
- REFERENCE: Link
- SYNONYMY:   
  ≡ *Cynoglossum rotundum* (Sutorý) Landolt 2010
- BASIS: Original material: Neuchâtel (Creux du Van). Henri Thomas. Deposited in G [from protologue]. Holotype: G (G00177370\*)

---

*Cynoglossum gileadense* Willd. in Roem. & Schult., Syst. Veg. 4: 761. 1819

- IPNI: Cynoglossum gileadense Willd. ex Roem. & Schult. -- Syst. Veg., ed. 15 bis [Roemer & Schultes] 4: 761. 1819 (IK)
- ASSESSMENT: [*Cynoglossum apenninum* L.]
- STATUS: spec. nov.
- REFERENCE: Link
- BASIS: Original material: Herb. Willd. [from protologue]. Holotype: B-W
- COMMENTS: The whole paragraph comprising the protologue (i.e., both the name and the diagnosis) is ascribed to “Willd. MSS”; hence the author of the name is Willdenow alone. Roemer & Schultes (l.c.) place Willdenow’s species under *Cynoglossum mollissimum* Lehm., which was obviously, though not demonstrably, based on the very same specimen. Willdenow’s epithet suggests an origin from Jordanian Gilead, whereas *C. mollissimum* is said to originate fron “Djilan” (Gilan) in NW Iran: Both provenances are erroneous (see Brand in Engler, Pflanzenr. 78 (IV.252): 154. 1921), but the (wrong) label associated with the type had Gilan, not Gilead.

---

*Cynoglossum glabellum* Riedl in Blumea 38: 462. 1994

- IPNI: Cynoglossum glabellum Riedl -- Blumea 38(2): 462 (1994). (IK)
- ASSESSMENT: accepted
- STATUS: spec. nov.
- REFERENCE: Link
- BASIS: Holotype: Veldkamp & Stevens 5554 (L), New Guinea, Goropu Mts (Mt Suckling), 1750 m, 9° 34' S, 148° 56' E, 11-vi-1972 [from protologue]; isotype: US (US00604019\*)

---

*Cynoglossum glabratum* (Pazij) Greuter & Stier in Biodivers. Data J. [hoc loco]. 2015

- ASSESSMENT: accepted
- STATUS: comb. nov.
- BASIS: Basionym: *Rindera glabrata* Pazij

---

*Cynoglossum glandulosum* (Khat.) Greuter & Stier in Biodivers. Data J. [hoc loco]. 2015

- ASSESSMENT: accepted
- STATUS: comb. nov.
- BASIS: Basionym: *Paracaryum glandulosum* Khat.

---

*Cynoglossum glastifolium* Willd., Sp. Pl. 1: 764. 1798

- IPNI: Cynoglossum glastifolium Willd. -- Sp. Pl., ed. 4 [Willdenow] 1(2): 764. 1798 [Jul 1798] (IK)
- ASSESSMENT: accepted
- STATUS: spec. nov.
- REFERENCE: Link
- SYNONYMY:   
  ≡ *Rindera glastifolia* (Willd.) Roem. & Schult. 1819
    
  ≡ *Mattia glastifolia* (Willd.) G. Don 1834-1838
    
  ≡ *Paracaryum glastifolium* (Willd.) Boiss. 1849
    
  ≡ *Mattiastrum glastifolium*  (Willd.) Brand 1915
- BASIS: Original material: *C. orientale glastifolium, flore atro-coeruleo. Tournef. Cor.* 7. Habitat in Armenia [from protologue]. Holotype: “*Roestel”*, B-W (B-W03340-01-0\*); probable isotype: *Tournefort* BM (BM001014426)

---

*Cynoglossum glochidiatum* of Wallich, Numer. List: 26. 1829

- IPNI: Cynoglossum glochidiatum Wall. -- Numer. List [Wallich] n. 922. 1829 (IK)
- STATUS: nom. inval. [nom. nud.]
- REFERENCE: Link

---

*Cynoglossum glochidiatum* Wall. ex Benth. in Royle, Ill. Bot. Himal. Mts.: 306. 1836

- IPNI: Cynoglossum glochidiatum Benth. -- Ill. Bot. Himal. Mts. [Royle] 306. (IK)
- ASSESSMENT: [*Cynoglossum wallichii* G. Don]
- STATUS: spec. nov.
- REFERENCE: Link
- SYNONYMY:   
  ≡ *Cynoglossum wallichii* var. *glochidiatum* (Wall. ex Benth.) Kazmi 1971
    
  ≡ *Paracynoglossum glochidiatum* (Wall. ex Benth.) Valdés 2011
- BASIS: Original material: Wall. Cat. n. 922; *C. vesiculosum*. Wall. Cat. n. 920. Also the Nepal specimens of *C. canescens*. Wall. Cat. n. 918 [from protologue]. Syntypes: A (A00096677\*), G-DC (G00205527\*, G00205528\*), GZU (GZU000106063\*), K (K000942367, K000942368\*), M (M0174199\*). Lectotype (to be designated elsewhere by König et al.): [India, West Bengal], “Tundua” [Pundua], F. Del. [F. De Silva], *Wallich 922* (K000942368\*)

---

*Cynoglossum glochidiatum* var. *alpinum* (Clarke) Brand in Engler, Pflanzenr. 78 (IV.252): 133. 1921

- IPNI: absent (2014-11-05)
- ASSESSMENT: [*Cynoglossum wallichii* G. Don]
- STATUS: comb. nov.
- REFERENCE: Link
- BASIS: Basionym: *Cynoglossum wallichii* var. *alpinum* Clarke

---

*Cynoglossum glomeratum* Fraser f. ex Pursh, Fl. Amer. Sept. 2: 729. 1813

- IPNI: Cynoglossum glomeratum Fraser f. -- Cat. (1813) ex Pursh, Fl. Am. Sept. ii. 729. (IK)
- ASSESSMENT: *Oreocarya celosioides*  Eastw.
- STATUS: spec. nov.
- REFERENCE: Link
- BASIS: Original material: In upper Louisiana. Bradbury (v. s. in Herb. Bradbury) [from protologue]

---

*Cynoglossum graecum* (A. DC.) Greuter & Burdet in Willdenowia 11: 35. 1981

- IPNI: Cynoglossum graecum (A.DC.) Greuter & Burdet -- Willdenowia 11(1): 35. 1981 (IK)
- ASSESSMENT: accepted
- STATUS: comb. nov.
- REFERENCE: Link
- BASIS: Basionym: *Mattia graeca* A. DC.

---

*Cynoglossum grande* Douglas ex Lehm., Nov. Stirp. Pug. 2: 25. 1830

- IPNI: Cynoglossum grande Douglas ex Lehm. -- Nov. Stirp. Pug. [Lehmann] 2: 25. 1830 [27 Aug 1830] (IK)
- ASSESSMENT: Not *Cynoglossum* s. l.; genus uncertain (species accepted)
- STATUS: spec. nov.
- REFERENCE: Link
- BASIS: Original material not indicated in protologue. Holotype presumably at MEL. Possible original specimens: BM (BM001024947\*), NY (NY00335201\*)

---

*Cynoglossum grande* var. *laeve* (A. Gray) A. Gray, Syn. Fl. N. Amer., ed. 2, 2: 421. 1886

- IPNI: Cynoglossum grande var. laeve A.Gray -- Syn. Fl. N. Amer., ed. 2. 2(1): 421. 1886 (GCI)
- ASSESSMENT: Not *Cynoglossum* s. l.; genus uncertain [*“Cynoglossum” grande* Douglas ex Lehm.]
- STATUS: comb. & stat. nov. [species to variety]
- REFERENCE: Link
- BASIS: Basionym: *Cynoglossum laeve* A. Gray

---

*Cynoglossum grandiflorum* Benth. in Royle, Ill. Bot. Himal. Mts.: 305. 1836

- IPNI: Cynoglossum grandiflorum Benth. -- Ill. Bot. Himal. Mts. [Royle] 305. (IK)
- ASSESSMENT: [*Cynoglossum microglochin* Royle ex Benth.]
- STATUS: spec. nov.
- REFERENCE: Link
- BASIS: Original material: Kashmere, Harpoo, Choor, Kederkanta [from protologue]. Original specimen(?): PH (PH00009263\*). Lectotype (to be designated elsewhere by König et al.): N. W. India [Cashmere, Hurpoo, Choor, Kedarkanta], *Royle* (K000998430)

---

*Cynoglossum gussonei* Strobl in Flora 67: 622. 1884

- IPNI: absent (2014-11-05)
- ASSESSMENT: *Cynoglossum nebrodense* Guss.
- STATUS: nom. illeg. [superfl.]
- REFERENCE: Link
- BASIS: Replaced synonym: *Cynoglossum nebrodense* Guss.
- COMMENTS: Proposed as nom. nov. for *Cynoglossum nebrodense* Guss. (1827) on the erroneous assumptions that *Cynoglossum nebrodense* of Jan (1831) was published in 1826 already and is a validly published name.

---

*Cynoglossum gymnandrum* (Coss.) Greuter & Burdet in Willdenowia 11: 35. 1981

- IPNI: Cynoglossum gymnandrum (Coss.) Greuter & Burdet -- Willdenowia 11(1): 35. 1981 (IK)
- ASSESSMENT: accepted
- STATUS: comb. nov.
- REFERENCE: Link
- BASIS: Basionym: *Mattia gymnandra* Coss.

---

*Cynoglossum haenkei* Schult., Oestr. Fl., ed. 2, 1: 361. 1814

- IPNI: Cynoglossum haenkei Schult. -- Oestr. Fl. ed. II. i. 362. (IK)
- ASSESSMENT: [*Cynoglossum germanicum* Jacq.]
- STATUS: nom. illeg. [superfl.]
- REFERENCE: Link
- BASIS: Replaced synonym: *Cynoglossum sylvaticum* Haenke

---

*Cynoglossum hanangense* Verdc. in Polhill, Fl. Trop. E. Africa, Boragin.: 113. 1991

- IPNI: Cynoglossum hanangense Verdc. -- Fl. Trop. E. Africa, Boragin. 113. 1991 (IK)
- ASSESSMENT: accepted
- STATUS: spec. nov.
- REFERENCE: Link
- BASIS: Holotype: Tanzania, Mbulu District, Mt. Hanang [Guruwe], B.D. Burtt 2264 (K, holo.!, EA, iso.) [from protologue]

---

*Cynoglossum hedbergiorum* Riedl in Linzer Biol. Beitr. 17: 319. 1985

- IPNI: Cynoglossum hedbergiorum Riedl -- Linzer Biol. Beitr. 17(2): 319 (1985). (IK)
- ASSESSMENT: Not *Cynoglossum* s. l.; genus uncertain [*“Cynoglossum” coeruleum* Hochst. ex A. DC.]
- STATUS: spec. nov.
- REFERENCE: Link
- BASIS: Holotype: Ethiopia, Shoa province, Sq. 59B. Near Ankobar, 162 km north east of Addis Ababa on Ankobar road. Alt. 3310 m (Aethiopia, prov. Shoa. Prope Ankobar, 162 km ab Addis Abeba boreo-orientem versus secus viam ad Ankobar ducentem, alt. 3310 m). Holotypus, UPS.-Leg. J.W. Ash Junio 26, 1971, no 1016 [from protologue]

---

*Cynoglossum hedgei* (Aytaç & R. R. Mill) Greuter & Stier in Biodivers. Data J. [hoc loco]. 2015

- ASSESSMENT: accepted
- STATUS: comb. nov.
- BASIS: Basionym: *Paracaryum hedgei* Aytaç & R. R. Mill

---

*Cynoglossum hellwigii* Brand in Repert. Spec. Nov. Regni Veg. 13: 546. 1915

- IPNI: Cynoglossum hellwigii Brand -- Repert. Spec. Nov. Regni Veg. 13: 546. 1915 (IK)
- ASSESSMENT: accepted
- STATUS: spec. nov.
- REFERENCE: Link
- BASIS: Original material: Neu-Guinea: Kaiser-Wilhelmsland: Finisterrae-Gebirge, zwischen den Steinen des Flussbettes. Im Oktober abblühend (Hellwig no. 362) [from protologue]

---

*Cynoglossum henricksonii* L. C. Higgins in Phytologia 34: 234. 1976

- IPNI: Cynoglossum henricksonii L.C.Higgins -- Phytologia 34(3): 234, nom. 1976 (IK)
- ASSESSMENT: Not *Cynoglossum* s. l.; genus uncertain (species accepted)
- STATUS: nom. nov.
- REFERENCE: Link
- BASIS: Replaced synonym: *Cynoglossum erectum* L. C. Higgins, non *Cynoglossum erectum* Schweigg. ex Schrank

---

*Cynoglossum heratense* (Rech. f. & Riedl) Greuter & Stier in Biodivers. Data J. [hoc loco]. 2015

- ASSESSMENT: accepted
- STATUS: comb. nov.
- BASIS: Basionym: *Mattiastrum heratense* Rech. f. & Riedl

---

*Cynoglossum heterocarpum* (Kunze) Willk., Ill. Fl. Hispan. 2: 121. 1891

- IPNI: Cynoglossum heterocarpum Kuntze ex Willk. -- Illustr. Fl. Hisp. ii. (1892 ?) 121 t. 160; et Suppl. Prodr. Fl. Hisp. 166. (IK)
- ASSESSMENT: *Cynoglossum cheirifolium* subsp. *heterocarpum* (Kunze) Font Quer
- STATUS: comb. & stat. nov. [variety to species]
- REFERENCE: Link
- BASIS: Basionym: *Cynoglossum cheirifolium* var. *heterocarpum* Kunze

---

*Cynoglossum heterocarpum* var. *mariolense* (Rouy) Willk., Suppl. Prodr. Fl. Hisp.: 167. 1893

- IPNI: absent (2014-11-05)
- ASSESSMENT: [*Cynoglossum cheirifolium* subsp. *heterocarpum* (Kunze) Font Quer]
- STATUS: comb. nov.
- REFERENCE: Link
- BASIS: Basionym: *Cynoglossum arundanum* var. *mariolense* Rouy

---

*Cynoglossum heynei* G. Don, Gen. Hist. 4: 354. 1837–1838

- IPNI: Cynoglossum heynei G.Don -- Gen. Hist. iv. 354. (IK)
- ASSESSMENT: [*Cynoglossum furcatum* Wall.]
- STATUS: spec. nov.
- REFERENCE: Link
- SYNONYMY:   
  ≡ *Cynoglossum furcatum* var. *lanceolatum* C. B. Clarke 1883
    
  ≡ *Cynoglossum zeylanicum* var. *lanceolatum* (C. B. Clarke) Brand 1921
- BASIS: Original material: Native of the East Indies, at Dindigul. *C. lanceolatum*, Heyne, herb. ex Wall. cat. no. 921. but not of Forsk. [from protologue]. Lectotype (Verdcourt in Kew Bull. 43: 347. 1988): Wallich 921.1, herb. Wallich, K; possible isolctotypes: A (A00096681\*), BM (BM001124575\*)

---

*Cynoglossum himalayense* (Klotzsch) Greuter & Stier in Biodivers. Data J. [hoc loco]. 2015

- ASSESSMENT: accepted
- STATUS: comb. nov.
- BASIS: Basionym: *Mattia himalayensis* Klotzsch

---

*Cynoglossum hintoniorum* B. L. Turner in Phytologia 79: 306. 1996

- IPNI: Cynoglossum hintoniorum B.L.Turner -- Phytologia 79(4): 306. 1996 [Oct 1995 publ. 10 Jul 1996] (IK)
- ASSESSMENT: *Hackelia hintoniorum*  (B. L. Turner) Sutorý
- STATUS: spec. nov.
- REFERENCE: Link
- SYNONYMY:   
  ≡ *Hackelia hintoniorum*  (B. L. Turner) Sutorý 2010
- BASIS: Holotype: Mexico. Oaxaca. Distrito Miahuatlán, Cerro Quiexobra, 3145 m. 19 Oct 1995, Hinton et al. 26206 (HOLOTYPE: TEX) [from protologue]. Isotype: IEB (IEB000224662\*)

---

*Cynoglossum hirsutissimum* Lehm., Pl. Asperif. Nucif.: 145. 1818

- IPNI: Cynoglossum hirsutissimum Lehm. -- Pl. Asperif. Nucif. 1: 147 (err. typ. 145). 1818 [Jul-early Sep 1818] (IK)
- ASSESSMENT: accepted
- STATUS: spec. nov.
- REFERENCE: Link
- BASIS: Original material: in Africa (v. s.) [from protologue]. Original drawing: MEL (reproduced in Ann. Naturhist. Mus. Wien 97B: 511. 1995); according to Riedl (l.c.: 509—512) likely representing *C. austroafricanum* Hilliard & Burtt

---

*Cynoglossum hirsutum* Thunb., Prodr. Pl. Cap.: 34. 1794

- IPNI: Cynoglossum hirsutum Thunb. -- Prodr. Pl. Cap. 1: 34. 1794 (IK)
- ASSESSMENT: Not *Cynoglossum* s. l.; genus uncertain [*“Cynoglossum” lanceolatum* Forssk.]
- STATUS: spec. nov.
- REFERENCE: Link
- SYNONYMY:   
  ≡ *Cynoglossum lanceolatum* var. *hirsutum* (Thunb.) DC. 1846
- BASIS: Described from S Afrca; original material not indicated in protologue. Original specimen: Cape of Good Hope, Thunberg, UPS

---

*Cynoglossum hirsutum* of Jacquin, Pl. Rar. Hort. Schoenbr. 4: 45. 1804

- IPNI: Cynoglossum hirsutum Jacq. -- Pl. Rar. Hort. Schoenbr. 4: 45, t. 489. 1804 (IK)
- STATUS: [isonym]
- REFERENCE: Link

---

*Cynoglossum hirsutum* of gardeners [Steudel, Nomencl. Bot.: 251. 1821]

- IPNI: absent (2014-11-05)
- STATUS: nom. inval. [pro syn. sub *Cynoglossum canescens*]
- REFERENCE: Link

---

*Cynoglossum hirtum* of Steudel, Nomencl. Bot.: 251. 1821

- IPNI: absent (2014-11-05)
- STATUS: nom. inval. [pro syn. sub *Cynoglossum canescens*]
- REFERENCE: Link

---

*Cynoglossum hispidum* Thunb., Prodr. Pl. Cap.: 34. 1794

- IPNI: Cynoglossum hispidum Thunb. -- Prodr. Pl. Cap. 1: 34. 1794 (IK)
- ASSESSMENT: Not *Cynoglossum* s. l.; genus uncertain [*“Cynoglossum” lanceolatum* Forssk.]
- STATUS: spec. nov.
- REFERENCE: Link
- BASIS: Described from S Africa; original material not indicated in protologue

---

*Cynoglossum hispidum* ascribed to Pursh by Steudel, Nomencl. Bot.: 251. 1821

- IPNI: Cynoglossum hispidum Pursh -- Fl. Amer. Sept. (Pursh) 2: 729. 1813 [Dec 1813] (IK)
- STATUS: nom. inval. [pro syn. sub *Cynoglossum canescens*]
- REFERENCE: Link
- COMMENTS: In Pursh (Fl. Amer. Sept. 2: 729. 1813) the word “hispidum” is not an epithet but is part of description of the species, *Cynoglossum glomeratum*.

---

*Cynoglossum hispidum* Jacq. ex Desf., Tabl. École Bot., ed. 2: 87. 1815

- IPNI: Cynoglossum hispidum Jacq. ex Roem. & Schult. -- Syst. Veg., ed. 15 bis [Roemer & Schultes] 4: 79, 761. 1819 (IK)
- ASSESSMENT: Not *Cynoglossum* s. l.; genus uncertain [*“Cynoglossum” lanceolatum* Forssk.]
- STATUS: nom. illeg. [superfl. and homonym]
- REFERENCE: Link
- BASIS: Replaced synonym: *Cynoglossum canescens* Willd.
- COMMENTS: “*Cynoglossum hispidum* Jacq. h. sch.” as accepted by Desfontaines, is a (non-correctable) error for “*C. hirsutum* Thunb.” as described and illustrated by Jacquin (Hort. Schoenbrunn. 4: 45, t. 489. 1804). Desfontaines’ name is illegitimate due to inclusion of *C. canescens* Willd. in synonymy.

---

*Cynoglossum hissaricum* (Lipsky) Greuter & Stier in Biodivers. Data J. [hoc loco]. 2015

- ASSESSMENT: accepted
- STATUS: comb. nov.
- BASIS: Basionym: *Trachelanthus hissaricus* Lipsky

---

*Cynoglossum hochstetteri* Vatke ex Engl. in Abh. Preuss. Akad. Wiss. 2: 353. 1892

- IPNI: Cynoglossum hochstetteri Vatke ex Engl. -- Abh. Preuss. Akad. Wiss. 1891, ii. (1892) 353 nomen. (IK)
- ASSESSMENT: *Cynoglossopsis latifolia*  (Hochst. ex A. Rich.) Brand
- STATUS: nom. nov.
- REFERENCE: Link
- BASIS: Replaced synonym: *Echinospermum latifolium*  Hochst. ex A. Rich., non *Cynoglossum latifolium* R. Br.

---

*Cynoglossum hochstetteri* var. *calathiforme* Chiov. in Ann. Bot. (Roma) 10: 398. 1912

- IPNI: absent (2014-11-05)
- ASSESSMENT: [*Cynoglossopsis latifolia*  (Hochst. ex A. Rich.) Brand]
- STATUS: var. nov.
- REFERENCE: Link
- BASIS: Original material: Eritrea: Samhar a Salomonà m. 450 c. 10. IV. 1910 (Pappi n. 8672); Assaorta, Valle Avero 5. IV. 1892 (Terracciano e Pappi n. 17); tra Cualo ed Enrot m. 500 c. 18. III. 1893 (Pappi n. 3050); Habab ad Oazat 21. IV. 1909 (Pappi n. 8338) [from protologue]. Syntypes: FT?

---

*Cynoglossum holochiton* (Popov) Greuter & Stier in Biodivers. Data J. [hoc loco]. 2015

- ASSESSMENT: accepted
- STATUS: comb. nov.
- BASIS: Basionym: *Rindera holochiton*  Popov

---

*Cynoglossum holosericeum* Steven in Mem. Soc. Imp. Naturalistes Moscou 3: 255. 1812

- IPNI: Cynoglossum holosericeum Steven -- Mém. Soc. Imp. Naturalistes Moscou iii. (1812) 255. (IK)
- ASSESSMENT: accepted
- STATUS: spec. nov.
- REFERENCE: Link
- BASIS: Original material: [Caucasus] in summis alpibus inter Chinalug [Khingal] et Wandam [from protologue]. Holotype (?): H

---

*Cynoglossum howardii* A. Gray, Syn. Fl. N. Amer. 2: 188. 1878 *(‘howardi’)*

- IPNI: Cynoglossum howardii A.Gray -- Syn. Fl. N. Amer. 2(1): 188. 1878 [May 1878] (as howardi) (GCI)
- ASSESSMENT: *Eritrichium howardii*  (A. Gray) Rydb.
- STATUS: spec. nov.
- REFERENCE: Link
- SYNONYMY:   
  ≡ *Eritrichium howardii*  (A. Gray) Rydb. 1900
- BASIS: Original material: Rocky Mountains in Montana, Winslow J. Howard [from protologue]. Holotype: GH (GH00096672\*); isotype: NY (NY00335202\*, fragm.)
- COMMENTS: Correction of the epithet’s original spelling mandated by ICN Art. 60.12.

---

*Cynoglossum humifusum* of Poeppig [A. de Candolle in Candolle, Prodr. 10: 133. 1846]

- IPNI: Cynoglossum humifusum Poepp. ex DC. -- Prodr. [A. P. de Candolle] 10: 133, in nota. 1846 [8 Apr 1846] (IK)
- STATUS: nom. inval. [pro syn. sub *Eritrichium procumbens*]
- REFERENCE: Link

---

*Cynoglossum hungaricum* Simonk. in Természetrajzi Füz. 2: 151. 1878

- IPNI: Cynoglossum hungaricum Simonk. -- in Termesz. Fuzet. 2: 151 (1878). (IK)
- ASSESSMENT: [*Cynoglossum montanum* L.]
- STATUS: spec. nov.
- REFERENCE: Link
- SYNONYMY:   
  ≡ *Cynoglossum germanicum* var. *hungaricum* (Simonk.) Brand 1921
- BASIS: Original material: in the protologue, various localities are cited [in Hungarian] . Lectotype (Brand in Engler, Pflanzenr. 78 (IV.252): 125. 1921, a “Original”): auf dem Adlersberg bei Budapest (Simonkai n. 2636)
- COMMENTS: The protologue paper is entirely in Hungarian; the author is given as Simkovics who is the same as Simonkai. Kováts (Studia Bot. Hung. 10: 124. 1975) gives the lectotype as: “Sashegy” ad Budapest (Hungary), which may or may not be the same specimen as Brand’s.

---

*Cynoglossum hupehense* (R. R. Mill) Greuter & Stier in Biodivers. Data J. [hoc loco]. 2015

- ASSESSMENT: accepted
- STATUS: comb. nov.
- BASIS: Basionym: *Solenanthus hupehensis* R. R. Mill

---

*Cynoglossum hybridum* Thuill., Fl. Env. Paris, ed. 2: 94. 1799

- IPNI: Cynoglossum hybridum Thuill. -- Fl. Par. 94. (IK)
- ASSESSMENT: [*Cynoglossum officinale* L.]
- STATUS: spec. nov.
- REFERENCE: Link
- SYNONYMY:   
  ≡ *Cynoglossum officinale* f. *hybridum* (Thuill.) Brand 1921
- BASIS: Original material: dans les bois du Gravier; dans celui du Pileux, près Villegenis [from protologue]

---

*Cynoglossum hystrix* Greuter & Stier in Biodivers. Data J. [hoc loco]. 2015

- ASSESSMENT: accepted
- STATUS: nom. nov.
- BASIS: Replaced synonym: *Rindera echinata* Regel, non *Cynoglossum echinatum* Thunb.

---

*Cynoglossum imeretinum* Kusn. in Kuznecov & al., Fl. Caucas. Crit. 4(2): 138. 1913

- IPNI: Cynoglossum imeretinum Kusn. -- Fl. Caucas. Crit. iv. II. 138 (1913). (IK)
- ASSESSMENT: [*Cynoglossum furcatum* Wall.]
- STATUS: spec. nov.
- REFERENCE: Link
- SYNONYMY:   
  ≡ *Paracynoglossum imeretinum* (Kusn.) Popov 1953
- BASIS: Original material: v. s. in h. P. J. Th. Hab. in Transcaucasia occident. [with 6 syntype specimens collected in the Kutaisi area by Medved’ev (2) and Buš (3), and in the Artvin district, Turkey, by Voronov] [from protologue]

---

*Cynoglossum incanum* (Ledeb.) Greuter & Burdet in Willdenowia 11: 35. 1981

- IPNI: Cynoglossum incanum (Ledeb.) Greuter & Burdet -- Willdenowia 11(1): 35. 1981 (IK)
- ASSESSMENT: accepted
- STATUS: comb. nov.
- REFERENCE: Link
- BASIS: Basionym: *Mattia incana* Ledeb.

---

*Cynoglossum indecorum* Greuter & Stier in Biodivers. Data J. [hoc loco]. 2015

- ASSESSMENT: accepted
- STATUS: nom. nov.
- BASIS: Replaced synonym: *Solenanthus micranthus* Riedl, non *Cynoglossum micranthum* Poir.

---

*Cynoglossum integerrimum* (P. Myrzakulov) Greuter & Stier in Biodivers. Data J. [hoc loco]. 2015

- ASSESSMENT: accepted
- STATUS: comb. nov.
- BASIS: Basionym: *Paracaryum integerrimum* Myrz.

---

*Cynoglossum intermedium* Fresen. in Mus. Senckenberg. 1: 169. 1834

- IPNI: Cynoglossum intermedium Fresen. -- Mus. Senckenberg. i. (1834) 169. (IK)
- ASSESSMENT: *Microparacaryum intermedium* (Fresen.) Hilger & Podlech
- STATUS: spec. nov.
- REFERENCE: Link
- SYNONYMY:   
  ≡ *Paracaryum intermedium* (Fresen.) Lipsky 1910
    
  ≡ *Microparacaryum intermedium* (Fresen.) Hilger & Podlech 1985
- BASIS: Original material: Peträisches Arabien (Rüppell) [from protologue]. Holotype: FR (FR0030035\*)

---

*Cynoglossum inyangense* E. S. Martins in Garcia de Orta, Ser. Bot. 9: 76. 1988

- IPNI: Cynoglossum inyangense E.S.Martins -- Garcia de Orta, Ser. Bot. 9(1-2): 76. 1988 [1987 publ. 1988] (IK)
- ASSESSMENT: accepted
- STATUS: spec. nov.
- REFERENCE: Link
- BASIS: Original material: Zimbabwe, Inyanga Downs, H. Wild 4934 (K, holotypus) [from protologue] Holotype: K (K000418914\*)

---

*Cynoglossum japonicum* Thunb. in Murray, Syst. Veg.: 187. 1784

- IPNI: Cynoglossum japonicum Thunb. -- Syst. Veg., ed. 14 (J. A. Murray). 187. 1784 [May-Jun 1784] (IK)
- ASSESSMENT: *Nihon japonicum* (Thunb.) Otero & al.
- STATUS: spec. nov.
- REFERENCE: Link
- SYNONYMY:   
  ≡ *Nihon japonicum* (Thunb.) Otero & al. 2014
- BASIS: Original material: [Japan] *Thunb. Jap. mspt.* M.(?) [from protologue]
- COMMENTS: Thunberg’s authorship of entries antedating his Flora Japonica is explicitly acknowledged in Murray’s preface, pp. XII-XIII.

---

*Cynoglossum japonicum* of Thunberg, Fl. Jap.: 81. 1784

- IPNI: Cynoglossum japonicum Thunb. -- Fl. Jap. (Thunberg) 81. 1784 [Aug 1784] (IK)
- STATUS: [isonym]
- REFERENCE: Link

---

*Cynoglossum javanicum* of Thunberg [Lehm., Pl. Asperif. Nucif.: 118. 1818]

- IPNI: Cynoglossum javanicum Thunb. ex Lehm. -- Pl. Asperif. Nucif. 1: 118. 1818 [Jul-early Sep 1818] (IK)
- STATUS: nom. inval. [pro syn. sub *Echinospermum javanicum*]
- REFERENCE: Link

---

*Cynoglossum javanicum* (Lehm.) A. DC. in Candolle, Prodr. 10: 588. 1846

- IPNI: absent (2014-11-05)
- ASSESSMENT: accepted
- STATUS: comb. nov.
- REFERENCE: Link
- BASIS: Basionym: *Echinospermum javanicum*  Lehm.

---

*Cynoglossum johnstonii* Baker in Bull. Misc. Inform. Kew 1894: 29. 1894 *(‘johnstoni’)*

- IPNI: Cynoglossum johnstonii Baker -- Bull. Misc. Inform. Kew 1894(85): 29. [Jan 1894] (IK)
- ASSESSMENT: Not *Cynoglossum* s. l.; genus uncertain [*“Cynoglossum” coeruleum* subsp. *johnstonii* (Baker) Verdc.]
- STATUS: spec. nov.
- REFERENCE: Link
- SYNONYMY:   
  ≡ *Cynoglossum coeruleum* var. *johnstonii* (Baker) Baker & Wright 1905
    
  ≡ *Cynoglossum coeruleum* subsp. *johnstonii* (Baker) Verdc. 1991
- BASIS: Original material: Kilimanjaro, alt. 6,000 ped. Johnston [from protologue]. Lectotype (Verdcourt in Polhill, Fl. Trop. E. Africa, Boragin.: 109. 1991): Tanzania, Kilimanjaro, 1800 m, *Johnston*, K (K000418924\*); isotype: BM (BM000930343\*)
- COMMENTS: Correction of the epithet’s original spelling mandated by ICN Art. 60.12.

---

*Cynoglossum kandavanense* (Bornm. & Gauba) Akhani in Stapfia 53: 80. 1998 *(‘kandavanensis’)*

- IPNI: Cynoglossum kandavanensis (Bornm. & Gauba) Akhani -- Stapfia 53: 80. 1998 (IK)
- ASSESSMENT: accepted
- STATUS: comb. nov.
- REFERENCE: Link
- BASIS: Basionym: *Lindelofia kandavanensis* Bornm. & Gauba
- COMMENTS: Correction of the epithet’s original spelling mandated by ICN Art. 23.5 & 32.2.

---

*Cynoglossum karakoricum* (Podlech & F. Sadat) Greuter & Stier in Biodivers. Data J. [hoc loco]. 2015

- ASSESSMENT: accepted
- STATUS: comb. nov.
- BASIS: Basionym: *Mattiastrum karakoricum* Podlech & F. Sadat

---

*Cynoglossum karamojense* Verdc. in Polhill, Fl. Trop. E. Africa, Boragin.: 115. 1991

- IPNI: Cynoglossum karamojense Verdc. -- Fl. Trop. E. Africa, Boragin. 115. 1991 (IK)
- ASSESSMENT: accepted
- STATUS: spec. nov.
- REFERENCE: Link
- BASIS: Original material: Uganda, Karamoja District, Mt. Morongolo, Dawkins 807 (K, holo.!, ENT, EA, iso.) [+ 3 paratypes] [from protologue]. Holotype: K; isotypes: ENT, EA

---

*Cynoglossum karataviense* (Pavlov ex Popov) Greuter & Stier in Biodivers. Data J. [hoc loco]. 2015

- ASSESSMENT: accepted
- STATUS: comb. nov.
- BASIS: Basionym: *Paracaryum karataviense* Pavlov ex Popov

---

*Cynoglossum karateginum* (Lipsky) Greuter & Stier in Biodivers. Data J. [hoc loco]. 2015

- ASSESSMENT: accepted
- STATUS: comb. nov.
- BASIS: Basionym: *Solenanthus karateginus*  Lipsky

---

*Cynoglossum khorassanicum* (Khat.) Greuter & Stier in Biodivers. Data J. [hoc loco]. 2015

- ASSESSMENT: accepted
- STATUS: comb. nov.
- BASIS: Basionym: *Paracaryum khorassanicum* Khat.

---

*Cynoglossum kokanicum* (Regel) Greuter & Stier in Biodivers. Data J. [hoc loco]. 2015

- ASSESSMENT: accepted
- STATUS: comb. nov.
- BASIS: Basionym: *Solenanthus kokanicus* Regel

---

*Cynoglossum korolkowii* (Lipsky) Greuter & Stier in Biodivers. Data J. [hoc loco]. 2015

- ASSESSMENT: accepted
- STATUS: comb. nov.
- BASIS: Basionym: *Trachelanthus korolkowii* Lipsky

---

*Cynoglossum korshinskyi* (Lipsky) Greuter & Stier in Biodivers. Data J. [hoc loco]. 2015

- ASSESSMENT: accepted
- STATUS: comb. nov.
- BASIS: Basionym: *Cyphomattia korshinskyi* Lipsky

---

*Cynoglossum krasniqii* of Wraber in Candollea 41: 145. 1986

- IPNI: Cynoglossum krasniqii Wraber -- Candollea 41(1): 145 (1986). (IK)
- STATUS: nom. inval. [type inappropriate]
- REFERENCE: Link
- COMMENTS: The presumed holotype consists of two specimens belonging to two different gatherings (as defined in ICN Art. 8.2): one in flower collected in April and one in fruit collected in July, and fails to meet the requirements of ICN Art. 40.1-2 for valid publication. The name does not appear to have ever been validated. As it is definitely needed, we take the liberty of validating it here in the name of its author.

---

*Cynoglossum krasniqii* Wraber in Biodivers. Data J. [hoc loco]. 2015

- ASSESSMENT: accepted
- STATUS: spec. nov.
- BASIS: Jugoslavija, Srbija, Kosovo: In pratis declivitatis meridionalis montis Paštrik supra vicum Gorožup prope oppidum Prizren. Solo calcareo, 1520 m s.m., 30.4.1983 (flor.), Leg. T. Wraber. Holotype: BEO; isotypes: LJU (LJU 111943 p.p., flowering plant), G (00177124 p.p., flowering plant)

---

*Cynoglossum kuhitangicum* (Raenko) Greuter & Stier in Biodivers. Data J. [hoc loco]. 2015

- ASSESSMENT: accepted
- STATUS: comb. nov.
- BASIS: Basionym: *Rindera kuhitangica*  Raenko

---

*Cynoglossum kuramense* (Turak.) Greuter & Stier in Biodivers. Data J. [hoc loco]. 2015

- ASSESSMENT: accepted
- STATUS: comb. nov.
- BASIS: Basionym: *Rindera kuramensis* Turak.

---

*Cynoglossum kurdistanicum* (Brand) Greuter & Burdet in Willdenowia 11: 35. 1981

- IPNI: Cynoglossum kurdistanicum (Brand) Greuter & Burdet -- Willdenowia 11(1): 35. 1981 (IK)
- ASSESSMENT: accepted
- STATUS: comb. nov.
- REFERENCE: Link
- BASIS: Basionym: *Mattiastrum kurdistanicum* Brand

---

*Cynoglossum laeve* A. Gray, Syn. Fl. N. Amer. 2: 188. 1878

- IPNI: Cynoglossum laeve A.Gray -- Syn. Fl. N. Amer. 2(1): 188. 1878 [May 1878] (IK)
- ASSESSMENT: Not *Cynoglossum* s. l.; genus uncertain [*“Cynoglossum” grande* Douglas ex Lehm.]
- STATUS: spec. nov.
- REFERENCE: Link
- SYNONYMY:   
  ≡ *Cynoglossum grande* var. *laeve* (A. Gray) A. Gray 1886
- BASIS: Original material: Plumas Co., California, *Mrs. Pulsifer-Ames* [from protologue]

---

*Cynoglossum laevigatum* L., Syst. Veg., ed. 13.: 157. 1774 *(‘laeuigatum’)*

- IPNI: Cynoglossum laevigatum L. -- Syst. Veg., ed. 13. 157. 1774 (IK)
- ASSESSMENT: *Cynoglossum tetraspis* (Pall.) Greuter & Burdet
- STATUS: nom. illeg. [superfl.]
- REFERENCE: Link
- BASIS: Replaced synonym: *Rindera tetraspis* Pall.
- COMMENTS: Correction of the epithet’s original spelling made under ICN Art. 60.1 (typographical or orthographical errors).

---

*Cynoglossum laevigatum* of Lehmann, Pl. Asperif. Nucif.: 174. 1818

- IPNI: Cynoglossum laevigatum Lehm. -- Pl. Asperif. Nucif. 1: 174. 1818 [Jul-early Sep 1818] (IK)
- STATUS: [isonym]
- REFERENCE: Link

---

*Cynoglossum lambertianum* (C. B. Clarke) Greuter & Stier in Biodivers. Data J. [hoc loco]. 2015

- ASSESSMENT: accepted
- STATUS: comb. nov.
- BASIS: Basionym: *Paracaryum lambertianum* C. B. Clarke

---

*Cynoglossum lamprocarpum* (Boiss.) Greuter & Burdet in Willdenowia 11: 35. 1981

- IPNI: Cynoglossum lamprocarpum (Boiss.) Greuter & Burdet -- Willdenowia 11(1): 35. 1981 (IK)
- ASSESSMENT: accepted
- STATUS: comb. nov.
- REFERENCE: Link
- BASIS: Basionym: *Paracaryum lamprocarpum* Boiss.

---

*Cynoglossum lanatum* Lam., Encycl. 2: 238. 1786

- IPNI: Cynoglossum lanatum Lam. -- Encycl. [J. Lamarck & al.] 2(1): 238. 1786 [16 Oct 1786] (IK)
- ASSESSMENT: accepted
- STATUS: spec. nov.
- REFERENCE: Link
- SYNONYMY:   
  ≡ *Mattia lanata* (Lam.) Roem. & Schult. 1809
    
  ≡ *Rindera lanata* (Lam.) Bunge 1851
    
  ≡ *Cyphomattia lanata* (Lam.) Boiss. 1875
- BASIS: Original material: *Cynoglossum orientale, flore roseo profundè laciniato, calyce tomentoso*. Tournef. Cor. 7. Tournefort a découvert cette plant dans le Levant [from protologue]. Lectotype (Davis, Fl. Turkey 6. 301: 1978): P-LA; probable isotype : M (M0174155\*)

---

*Cynoglossum lanatum* of Aucher-Eloy [A. de Candolle in Candolle, Prodr. 10: 169. 1846]

- IPNI: Cynoglossum lanatum Aucher ex DC. -- Prodr. [A. P. de Candolle] 10: 169. 1846 [8 Apr 1846] (IK)
- STATUS: nom. inval. [pro syn. sub *Mattia aucheri*]
- REFERENCE: Link

---

*Cynoglossum lanceolatum* Forssk., Fl. Aegypt.-Arab.: 41. 1775

- IPNI: Cynoglossum lanceolatum Forssk. -- Fl. Aegypt.-Arab. 41. 1775 [1 Oct 1775] (IK)
- ASSESSMENT: Not *Cynoglossum* s. l.; genus uncertain (species accepted)
- STATUS: spec. nov.
- REFERENCE: Link
- SYNONYMY:   
  ≡ *Paracynoglossum lanceolatum* (Forssk.) R. R. Mill 1984
- BASIS: Original material: Hadîe [from protologue]. Probable holotype: Herb. Forskålii. N° 312, C (C10002127\*)

---

*Cynoglossum lanceolatum* of Heyne [Wallich, Numer. List: 26. 1829]

- IPNI: Cynoglossum lanceolatum B.Heyne ex Wall. -- Numer. List [Wallich] n. 921. 1829 (IK)
- STATUS: nom. inval. [nom. nud.]
- REFERENCE: Link
- COMMENTS: In Wallich’s autographic list, the name is ascribed to Heyne and Forsskål’s *Cynoglossum lanceolatum* is associated with it only doubtfully. The corresponding specimens are the basis of *Cynoglossum heynei* G. Don.

---

*Cynoglossum lanceolatum* of Hochstetter [Candolle, Prodr. 10: 149. 1846]

- IPNI: Cynoglossum lanceolatum Hochst. ex DC. -- Prodr. [A. P. de Candolle] 10: 149. 1846 [8 Apr 1846] (IK)
- STATUS: nom. inval. [pro syn. sub *Cynoglossum micranthum*]
- REFERENCE: Link

---

*Cynoglossum lanceolatum* f. *albiflorum* (Masam.) Yonek. in J. Jap. Bot. 80: 329. 2005

- IPNI: Cynoglossum lanceolatum Forssk. f. albiflorum (Masam.) Yonek. -- J. Jap. Bot. 80(6): 329. 2005 [Dec 2005]
- ASSESSMENT: Not *Cynoglossum* s. l.; genus uncertain [*“Cynoglossum” formosanum* Nakai]
- STATUS: comb. nov.
- REFERENCE: Link
- BASIS: Basionym: *Cynoglossum formosanum* f. *albiflorum* Masam.

---

*Cynoglossum lanceolatum* var. *comoranum* Brand in Engler, Pflanzenr. 78 (IV.252): 140. 1921

- IPNI: absent (2014-11-05)
- ASSESSMENT: Not *Cynoglossum* s. l.; genus uncertain [*“Cynoglossum” lanceolatum* Forssk.]
- STATUS: var. nov.
- REFERENCE: Link
- BASIS: Original material: Comoren: Ohne nähere Angabe (Humblot n. 97) [from protologue]

---

*Cynoglossum lanceolatum* var. *formosanum* of Hara, Fl. E. Himalaya: 266. 1966

- IPNI: absent (2014-11-05)
- STATUS: nom. inval. [basionym not cited, ICN Art. 41.5]
- REFERENCE: Link
- COMMENTS: By implication, the intended basionym was *Cynoglossum formosanum* Nakai.

---

*Cynoglossum lanceolatum* subsp. *geometricum* (Baker & C. H. Wright) Brand in Engler, Pflanzenr. 78 (IV.252): 140. 1921

- IPNI: absent (2014-11-05)
- ASSESSMENT: Not *Cynoglossum* s. l.; genus uncertain (subspecies accepted)
- STATUS: comb. & stat. nov. [species to subspecies]
- REFERENCE: Link
- BASIS: Basionym: *Cynoglossum geometricum* Baker & C. H. Wright

---

*Cynoglossum lanceolatum* var. *hirsutum* (Thunb.) DC., Prodr. 10: 155. 1846

- IPNI: absent (2014-11-05)
- ASSESSMENT: Not *Cynoglossum* s. l.; genus uncertain [*“Cynoglossum” hirsutum* Thunb.]
- STATUS: comb. & stat. nov. [species to variety]
- REFERENCE: Link
- BASIS: Basionym: *Cynoglossum hirsutum* Thunb.

---

*Cynoglossum lanceolatum* var. *mannii* (Baker & C. H. Wright) Brand in Engler, Pflanzenr. 78 (IV.252): 140. 1921

- IPNI: absent (2014-11-05)
- ASSESSMENT: Not *Cynoglossum* s. l.; genus uncertain [*“Cynoglossum” lanceolatum* subsp. *geometricum* (Baker & C. H. Wright) Brand]
- STATUS: comb. & stat. nov. [species to variety]
- REFERENCE: Link
- BASIS: Basionym: *Cynoglossum mannii* Baker & C. H. Wright

---

*Cynoglossum lancifolium* Hook. f. in J. Proc. Linn. Soc., Bot. 7: 207. 1864

- IPNI: Cynoglossum lancifolium Hook.f. -- J. Proc. Linn. Soc., Bot. 7: 207. 1864 (IK)
- ASSESSMENT: Not *Cynoglossum* s. l.; genus uncertain [*Cynoglossum amplifolium* Hochst. ex A. DC.]
- STATUS: spec. nov.
- REFERENCE: Link
- SYNONYMY:   
  ≡ *Cynoglossum amplifolium* f. *macrocarpum* Brand 1921
- BASIS: Original material: Cameroons Mountains, alt. 7000–8000 feet [from protologue]. Original specimens (fide Verdcourt in Polhill, Fl. Trop. E. Africa, Boragin.: 106. 1991) *Mann 1266* [“*1866*”] & *2004*, K (K000029829\*, K000029830\*, K000029831\*, K000029832\*)

---

*Cynoglossum lanuginosum* Lehm., Pl. Asperif. Nucif.: 148. 1818

- IPNI: Cynoglossum lanuginosum Lehm. -- Pl. Asperif. Nucif. 1: 148. 1818 [Jul-early Sep 1818] (IK)
- ASSESSMENT: *Cynoglossum magellense*  Ten.
- STATUS: spec. nov.
- REFERENCE: Link
- BASIS: Original material: (v. s.) [details lacking] [from protologue]. Holotype: MEL (see Riedl in Ann. Naturhist. Mus. Wien 97B: 512. 1995)

---

*Cynoglossum lappula* (L.) Scop., Fl. Carniol., ed. 2, 1: 125. 1771

- IPNI: Cynoglossum lappula Scop. -- Fl. Carniol., ed. 2. 1: 125. 1771 (IK)
- ASSESSMENT: [*Lappula squarrosa*  (Retz.) Dumort.]
- STATUS: comb. nov.
- REFERENCE: Link
- BASIS: Basionym: *Myosotis lappula*  L.

---

*Cynoglossum lateriflorum* Lam., Encycl. 2: 239. 1786

- IPNI: Cynoglossum lateriflorum Lam. -- Encycl. [J. Lamarck & al.] 2(1): 239. 1786 [16 Oct 1786] (IK)
- ASSESSMENT: *Pectocarya lateriflora*  (Lam.) A. DC.
- STATUS: spec. nov.
- REFERENCE: Link
- SYNONYMY:   
  ≡ *Rindera lateriflora* (Lam.) Roem. & Schult. 1819
    
  ≡ *Mattia lateriflora* (Lam.) G. Don 1837-1838
    
  ≡ *Pectocarya lateriflora*  (Lam.) A. DC. 1846
- BASIS: Original material: a été découverte aux environs de Lima, dans des lieux arides, par M. Dombey, qui nous l’a communiquée. Domb. Herb. Peruv. [from protologue]. Holotype: P-LA; likely isotypes: F (F0052553F\*), MA (MA814846\*), US (US00110904\*, fragm.)

---

*Cynoglossum lateriflorum* of Aubry in Exerc. Hist. Nat. 1802: 25. 1802

- IPNI: Cynoglossum lateriflorum Aubr. -- Prog. Morb. x. 25. (IK)
- STATUS: [isonym]
- REFERENCE: Link
- COMMENTS: Macbride (in Proc. Amer. Acad. Arts 51: 543. 1916) based what he thought to be a new combination on this misapplied name: *Omphalodes lateriflora* Macbride, an illegitimate name for *O. littoralis* Lehm. (1818) that it was intended to replace.

---

*Cynoglossum lateriflorum* of gardeners [Steudel, Nomencl. Bot., ed. 2, 1: 464. 1840]

- IPNI: Cynoglossum lateriflorum Hort. ex Steud. -- Nomencl. Bot. [Steudel], ed. 2. 1: 464. 1840 (IK)
- STATUS: nom. inval. [pro syn. sub *Ktenospermum linifolium*]
- REFERENCE: Link

---

*Cynoglossum lateriflorum* of Bertero [DC., Prodr. 10: 120. 1846]

- IPNI: Cynoglossum lateriflorum Bert. ex DC. -- Prodr. [A. P. de Candolle] 10: 120. 1846 [8 Apr 1846] (IK)
- STATUS: nom. inval. [pro syn. sub *Pectocarya chilensis*]
- REFERENCE: Link

---

*Cynoglossum latifolium* R. Br., Prodr. Fl. Nov. Holland.: 496. 1810

- IPNI: Cynoglossum latifolium R.Br. -- Prodr. Fl. Nov. Holland. 496. 1810 [27 Mar 1810] (IK)
- ASSESSMENT: *Austrocynoglossum latifolium* (R. Br.) R. R. Mill
- STATUS: spec. nov.
- REFERENCE: Link
- SYNONYMY:   
  ≡ *Austrocynoglossum latifolium* (R. Br.) R. R. Mill 1989
- BASIS: Original material: [Australia] (J.) [Port Jackson, Brown] (v. v.) [from protologue]. Syntypes: BM (BM001040569\*), E, K

---

*Cynoglossum laxiflorum* (Trautv.) Greuter & Burdet in Willdenowia 11: 35. 1981

- IPNI: Cynoglossum laxiflorum (Trautv.) Greuter & Burdet -- Willdenowia 11(1): 35. 1981 (IK)
- ASSESSMENT: accepted
- STATUS: comb. nov.
- REFERENCE: Link
- BASIS: Basionym: *Paracaryum laxiflorum* Trautv.

---

*Cynoglossum laxum* G. Don, Gen. Hist. 4: 356. 1837–1838

- IPNI: Cynoglossum laxum G.Don -- Gen. Hist. iv. 356. (IK)
- ASSESSMENT: [*Hackelia uncinata* (Royle ex Benth.) C. E. C. Fischer]
- STATUS: spec. nov.
- REFERENCE: Link
- BASIS: Original material: H. [in hothouse]. Native of Emodus and Gosainsthan, in Nipaul. Rindèra glochidàta, Wall. cat. no. 926. Picòtia glochidàta, Wall. mss. [from protologue]. Syntypes: Wallich 926, E (E00288382\*), GZU (GZU000106060\*), NY (NY00335204\*)
- COMMENTS: The protologue of both *Cynoglossum laxum* and *Cynoglossum uncinatum* Royle ex Benth. included reference to the gathering(s) Wallich 926, designated as *Rindera glochidiata* of Wallich; however, the latter also included Royle’s own material, and as the name itself was not cited by Don, nor was it typified as yet, *C. laxum* is a legitimate name.

---

*Cynoglossum legionense* Rothm. in Cavanillesia 7: 120. 1935, pro hybr.

- IPNI: Cynoglossum × legionense Rothm. -- Cavanillesia vii. 120(1935). (IK)
- ASSESSMENT: accepted as hybrid
- STATUS: hybr. nov. [*C. creticum* × *C. officinale*]
- REFERENCE: Link
- BASIS: Original material: Regno Legion., in dumetis prope oppidum Ponferrada inter parentes legi [from protologue]

---

*Cynoglossum leptophyllum* (A. DC.) Greuter & Burdet in Willdenowia 11: 35. 1981

- IPNI: Cynoglossum leptophyllum (A.DC.) Greuter & Burdet -- Willdenowia 11(1): 35. 1981 (IK)
- ASSESSMENT: accepted
- STATUS: comb. nov.
- REFERENCE: Link
- BASIS: Basionym: *Mattia leptophylla* A. DC.

---

*Cynoglossum leptostachyum* DC., Prodr. 10: 152. 1846

- IPNI: Cynoglossum leptostachyum DC. -- Prodr. [A. P. de Candolle] 10: 152. 1846 [8 Apr 1846] (IK)
- ASSESSMENT: accepted
- STATUS: spec. nov.
- REFERENCE: Link
- BASIS: Original material: ad Cap. Bonae-Spei (Drège! 4889) (v. s. a cl. inv.) [from protologue]. Holotype: G-DC (G00205669\*)

---

*Cynoglossum limense* Willd., Sp. Pl. 1: 762. 1798

- IPNI: Cynoglossum limense Willd. -- Sp. Pl., ed. 4 [Willdenow] 1(2): 762. 1798 [Jul 1798] (IK)
- ASSESSMENT: Not *Cynoglossum* s. l.; genus uncertain (species accepted)
- STATUS: spec. nov.
- REFERENCE: Link
- SYNONYMY:   
  ≡ *Cynoglossum decurrens* var. *limense* (Willd.) DC. 1846
- BASIS: Original material: *C. foliis nervosas acutissimis*. Feuill. Peruv.“1” [2] p. “71” [765] t. 49. Habitat in Lima [from protologue]. Holotype: illustration in Feuillée, J. Obs. 2: t. 49. 1714. An asterisk, in the protologue, indicates that Willdenow saw no specimen
- COMMENTS: According to Johnston (Contr. Gray Herb. 78: 110. 1927) Feuillée found the plant in the “vallée dʼYlo”, near the southern boundary of Peru, not near Lima as Willdenow’s inappropriate specific name would suggest. As the plant has not been reported from within 500 miles of Ylo but from Concepcion (Chile) it is possible that its reputed occurrence there is the result of some confusion.

---

*Cynoglossum lineare* Ruiz & Pav., Fl. Peruv. 2: 6. 1799

- IPNI: Cynoglossum lineare Ruiz & Pav. -- Fl. Peruv. [Ruiz & Pavon] 2: 6. 1799 (IK)
- ASSESSMENT: [*Pectocarya lateriflora*  (Lam.) A. DC.]
- STATUS: spec. nov.
- REFERENCE: Link
- SYNONYMY:   
  ≡ *Pectocarya linearis*  (Ruiz. & Pav.) DC. 1846
- BASIS: Original material: in Sancti Jacobi Chilensis campis aridis [from protologue]

---

*Cynoglossum lineatum* of Risso [Cesati & al., Comp. Fl. Ital. 2: 380. 1876]

- IPNI: Cynoglossum lineatum Risso ex Ces., Pass. & Gibelli -- Comp. Fl. Ital. [Cesati] 2(17): 380. 1876 (IK)
- STATUS: nom. inval. [pro syn. sub *Cynoglossum cheirifolium*]
- REFERENCE: Link

---

*Cynoglossum linifolium* L., Sp. Pl.: 134. 1753

- IPNI: Cynoglossum linifolium L. -- Sp. Pl. 1: 134. 1753 [1 May 1753] (IK)
- ASSESSMENT: *Omphalodes linifolia*  (L.) Moench
- STATUS: spec. nov.
- REFERENCE: Link
- SYNONYMY:   
  ≡ *Omphalodes linifolia*  (L.) Moench 1794
- BASIS: Original material: *Moris. Hist.* 3. *p*. 449. *s.* ii, *t.* 30. f. 11. Habitat in Lusitania [from protologue]. Lectotype (Selvi in Taxon 53: 801. 2004): Herb. Clifford: 47, *Cynoglossum* 3, sheet 2 (BM000557922\*); possible isolectotype: BM (BM000557923\*)

---

*Cynoglossum linifolium* of Bové [Candolle, Prodr. 10: 159. 1846]

- IPNI: Cynoglossum linifolium Bové ex DC. -- Prodr. [A. P. de Candolle] 10: 159. 1846 [8 Apr 1846] (IK)
- STATUS: nom. inval. [pro syn. sub *Omphalodes micrantha*]
- REFERENCE: Link

---

*Cynoglossum linifolium* var. *caerulescens* DC. in Lamarck & Candolle, Fl. Franç., ed. 3, 6: 422. 1815

- IPNI: absent (2014-11-05)
- ASSESSMENT: [*Omphalodes linifolia*  (L.) Moench]
- STATUS: var. nov.
- REFERENCE: Link
- SYNONYMY:   
  ≡ *Omphalodes linifolia*  var. *caerulescens* (DC.) DC. 1846
- BASIS: Original material: trouvée dans les garrigues entre Carpentras et Bedoin [from protologue]. Holotype: G-DC (G00205740\*)
- COMMENTS: Candolle, in the protologue, ascribes the varietal epithet to “Req. in Guer. Vaucl. ed. 2, p. 251”). But in that work Guérin (Descr. Fontaine Vaucluse, ed. 2. 1813) describes the species with white flowers, having seen it only in fruit at the said locality.

---

*Cynoglossum lithospermifolium* Lam., Encycl. 2: 240. 1786

- IPNI: Cynoglossum lithospermifolium Lam. -- Encycl. [J. Lamarck & al.] 2(1): 240. 1786 [16 Oct 1786] (IK)
- ASSESSMENT: accepted
- STATUS: spec. nov.
- REFERENCE: Link
- SYNONYMY:   
  ≡ *Picotia lithospermifolia*  (Lam.) Roem. & Schult. 1819
    
  ≡ *Paracaryum lithospermifolium* (Lam.) Grande 1914
    
  ≡ *Mattiastrum lithospermifolium* (Lam.) Brand 1915
    
  ≡ *Cynoglossum myosotoides* Labill. 1791, nom illeg.
    
  ≡ *Omphalodes myosotoides*  Schrank 1812, nom illeg.
    
  ≡ *Paracaryum myosotoides* Boiss. 1849, nom illeg.
- BASIS: Original material: Nous avons vu cette plante dans lʼHerbier de M. Jussieu; elle croît vraisemblement dans lʼEgypte [from protologue]. Holotype (presumably): P-JU

---

*Cynoglossum lithospermifolium* subsp. *cariense* (Boiss.) Greuter & Burdet in Willdenowia 11: 35. 1981

- IPNI: Cynoglossum lithospermifolium Lam. subsp. cariense (Boiss.) Greuter & Burdet -- Willdenowia 11(1): 35. 1981 (IK)
- ASSESSMENT: accepted
- STATUS: comb. nov.
- REFERENCE: Link
- BASIS: Basionym: *Omphalodes cariensis* Boiss.

---

*Cynoglossum littorale* (Lehm.) Spreng., Syst. Veg. 1: 567. 1824 *(‘litorale’)*

- IPNI: Cynoglossum litorale Spreng. -- Syst. Veg. (ed. 16) [Sprengel] 1: 567. 1824 [dated 1825; publ. in late 1824] (IK)
- ASSESSMENT: *Omphalodes littoralis*  Lehm.
- STATUS: comb. nov.
- REFERENCE: Link
- BASIS: Basionym: *Omphalodes littoralis*  Lehm.

---

*Cynoglossum longepetiolatum* De Wild., Pl. Bequaert. 4: 15. 1926

- IPNI: Cynoglossum longepetiolatum De Wild. -- Pl. Bequaert. iv. 15 (1926). (IK)
- ASSESSMENT: Not *Cynoglossum* s. l.; genus uncertain [*“Cynoglossum” amplifolium* Hochst. ex A. DC.]
- STATUS: spec. nov.
- REFERENCE: Link
- BASIS: Original material: Mukule, 26 septembre 1914 (J. Bequaert, n. 5904 ...) [from protologue]. Syntypes: BR (BR0000008865359 \*, BR0000008865366\*)

---

*Cynoglossum longiflorum* Lehm., Pl. Asperif. Nucif.: 162. 1818

- IPNI: Cynoglossum longiflorum Lehm. -- Pl. Asperif. Nucif. 1: 162. 1818 [Jul-early Sep 1818] (IK)
- ASSESSMENT: [*Caccinia macranthera* (Banks & Sol.) Brand]
- STATUS: spec. nov.
- REFERENCE: Link
- BASIS: Original material: circa Aleppum [from protologue]. Holotype (presumably): MEL

---

*Cynoglossum longiflorum* Royle ex Benth. in Royle, Ill. Bot. Himal. Mts.: 305. 1836

- IPNI: Cynoglossum longiflorum Benth. -- Ill. Bot. Himal. Mts. [Royle] 305. (IK)
- ASSESSMENT: [*Cynoglossum longifolium* (Leichtlin ex Beck & F. Abel) Greuter & Stier]
- STATUS: nom. illeg. [homonym]
- REFERENCE: Link
- SYNONYMY:   
  ≡ *Omphalodes longiflora*  A. DC. 1846
    
  ≡ *Paracaryum longiflorum* (A. DC.) Boiss. 1849
    
  ≡ *Lindelofia spectabilis* Lehm. 1850, nom. illeg.
    
  ≡ *Anchusopsis longiflora* (A. DC.) Bisch. 1852
    
  ≡ *Lindelofia longiflora* (A. DC.) Baill. 1890
- BASIS: Original material: Exemplaria incompleta: Cashmere [from protologue]

---

*Cynoglossum longifolium* (Leichtlin ex Beck & F. Abel) Greuter & Stier in Biodivers. Data J. [hoc loco]. 2015

- ASSESSMENT: accepted
- STATUS: comb. nov.
- BASIS: Basionym: *Lindelofia longifolia* Leichtlin ex Beck & F. Abel

---

*Cynoglossum longipedicellatum* (Riedl) Greuter & Stier in Biodivers. Data J. [hoc loco]. 2015

- ASSESSMENT: accepted
- STATUS: comb. nov.
- BASIS: Basionym: *Lindelofia longipedicellata* Riedl

---

*Cynoglossum longipes* (Boiss. & Balansa) Greuter & Burdet in Willdenowia 11: 35. 1981

- IPNI: Cynoglossum longipes (Boiss.) Greuter & Burdet -- Willdenowia 11(1): 35. 1981 (IK)
- ASSESSMENT: accepted
- STATUS: comb. nov.
- REFERENCE: Link
- BASIS: Basionym: *Paracaryum longipes* Boiss. & Balansa

---

*Cynoglossum loreyi* Jord. ex Lange in Vidensk. Meddel. Naturhist. Foren. Kjøbenhavn 1863: 28. 1863

- IPNI: Cynoglossum loreyi Jord. ex Lange -- Vidensk. Meddel. Naturhist. Foren. Kjøbenhavn (1863) 28. (IK)
- ASSESSMENT: [*Cynoglossum dioscoridis* Vill.]
- STATUS: spec. nov.
- REFERENCE: Link
- SYNONYMY:   
  ≡ *Cynoglossum pictum* var. *loreyi* (Jord. ex Lange) Nyman 1881
- BASIS: Original material: Aranjuez, 21.Mai c. fl. et fr. jun. Specimina mea [Lange] excte cum specimine a cel. Jordan misso conveniunt [from protologue]. Syntypes (presumably): C

---

*Cynoglossum lowryanum* J. S. Mill. in Adansonia, ser. 3, 27: 122. 2005

- IPNI: Cynoglossum lowryanum J.S.Mill. -- Adansonia ser. 3, 27(1): 122 (115; figs. 2-3). 2005 [30 Jun 2005]
- ASSESSMENT: accepted
- STATUS: spec. nov.
- REFERENCE: Link
- BASIS: Original material: Perrier de la Bâthie 2206, Madagascar, Prov. Toamasina, bassin du l’Onive-Mangoro, Forêt d’Andasibe, 1400 m, 18°56’S, 48°25’E, fl., fr., Nov. 1911 (holo-, P!) [from protologue]. Holotype: (P00319882 = PL03494374\*)

---

*Cynoglossum lucidum* Stokes, Bot. Mat. Med. 1: 277. 1812

- IPNI: Cynoglossum lucidum Stokes -- Bot. Mat. Med. i. 277. 1812 (IK)
- ASSESSMENT: Not *Cynoglossum* s. l.; genus uncertain [*“Cynoglossum” virginianum* L.]
- STATUS: nom. illeg. [superfl.]
- REFERENCE: Link
- BASIS: Replaced synonym: *Cynoglossum virginianum* L.

---

*Cynoglossum luristanicum* (Nábělek) Greuter & Stier in Biodivers. Data J. [hoc loco]. 2015

- ASSESSMENT: accepted
- STATUS: comb. nov.
- BASIS: Basionym: *Paracaryum luristanicum* Nábělek

---

*Cynoglossum lusitanicum* L., Sp. Pl., ed. 2: 193. 1762

- IPNI: absent (2014-11-05)
- ASSESSMENT: *Omphalodes* sp. (nomen dubium)
- STATUS: spec. nov.
- REFERENCE: Link
- SYNONYMY:   
  ≡ *Omphalodes lusitanica*  (L.) Schrank 1812
- BASIS: Original material: in Lusitania [from protologue]. Lectotype (Selvi in Taxon 53: 802. 2004): Herb. Linn. No. 183.8, LINN (LINN-HL183-8\*)
- COMMENTS: See under *Omphalodes lusitanica*  (L.) Schrank.

---

*Cynoglossum lusitanicum* of Miller, Gard. Dict., ed. 8: Cynoglossum n. 6. 1768

- IPNI: Cynoglossum lusitanicum Mill. -- Gard. Dict., ed. 8. n. 6. 1768 [16 Apr 1768] (IK)
- STATUS: [isonym]
- REFERENCE: Link

---

*Cynoglossum lusitanicum* Fortis, Osserv. Sopra Cherso ed Osero: 68. 1771

- IPNI: Cynoglossum lusitanicum Fortis -- Osserv. Sopra Cherso ed Osero 68. (IK)
- ASSESSMENT: [*Lappula squarrosa*  (Retz.) Dumort.(?)]
- STATUS: nom. illeg. [homonym]
- REFERENCE: Link
- BASIS: Original material: described from isola di Cherso [according to protologue]
- COMMENTS: Tentative identification, by F. Selvi, Firenze, based on the original description.

---

*Cynoglossum lusitanicum* of Lamarck, Encycl. 2: 239. 1786

- IPNI: Cynoglossum lusitanicum Lam. -- Encycl. [J. Lamarck & al.] 2(1): 239. 1786 [16 Oct 1786] (IK)
- STATUS: [isonym]
- REFERENCE: Link
- COMMENTS: Sometimes erroneously considered a (heterotypic) later homonym, but although the description differs, the type of *Cynoglossum lusitanicum* L. was explicitly included.

---

*Cynoglossum lycium* of Boissier [Brand in Engler, Pflanzenr. 78 (IV.252): 127. 1921]

- IPNI: Cynoglossum lycium Boiss. ex Brand -- Pflanzenr. (Engler) Borrag.-Cynogloss. 127 (1921), in syn. (IK)
- STATUS: nom. inval. [pro syn. sub *Cynoglossum montanum* var. *asiaticum*]
- REFERENCE: Link

---

*Cynoglossum macranthum* Regel & Smirn. in Trudy Imp. S.-Peterburgsk. Bot. Sada 5: 623. 1877

- IPNI: Cynoglossum macranthum Regel & Smirnow -- Trudy Imp. S.-Peterburgsk. Bot. Sada v. (1877) 623. (IK)
- ASSESSMENT: [*Cynoglossum anchusoides* Lindl.]
- STATUS: spec. nov.
- REFERENCE: Link
- BASIS: Original material: In Turkestania prope Taschkent floribus caeruleis (A. Regel), ad fluvium Syr Darjy floribus ut videtur albis (Golicke) [from protologue]. Syntypes presumably at LE

---

*Cynoglossum macrocalycinum* Riedl in Novon 4: 46. 1994

- IPNI: Cynoglossum macrocalycinum Riedl -- Novon 4(1): 46. 1994 (IK)
- ASSESSMENT: accepted
- STATUS: spec. nov.
- REFERENCE: Link
- BASIS: Original material: China, Sinkiang [Xinjiang], Shawan County, Ziniquan, Ning jiahe, hill slope, 1,750 m, T. Zhou s.n. (holotype, NAS) [from protologue]

---

*Cynoglossum macrolimbe* Riedl in Linzer Biol. Beitr. 22: 310. 1990

- IPNI: Cynoglossum macrolimbe Riedl -- Linzer Biol. Beitr. 22(1): 310 (1990). (IK)
- ASSESSMENT: accepted
- STATUS: spec. nov.
- REFERENCE: Link
- BASIS: Original material: Papua-Nova Guinea: in declivibus montium Dayman, in valle fluminis Guarim superioris. 2050 m. 30 Maio 1953. BRASS n. 22640 (holotypus LAE, isotypus A).[from protologue]. Isotype: A (A00075140\*)

---

*Cynoglossum macrophyllum* of Royle [Bentham in Royle, Ill. Bot. Himal. Mts.: 305. 1836]

- IPNI: absent (2014-11-05)
- STATUS: nom. inval. [pro syn. sub *Cynoglossum uncinatum* var. *laxiflorum*]
- REFERENCE: Link

---

*Cynoglossum macrophyllum* of Royle [Candolle, Prodr. 10: 136. 1846]

- IPNI: Cynoglossum macrophyllum Royle ex DC. -- Prodr. [A. P. de Candolle] 10: 136. 1846 [8 Apr 1846] (IK)
- STATUS: nom. inval. [pro syn. sub *Cynoglossum uncinatum* var. *laxiflorum*]
- REFERENCE: Link

---

*Cynoglossum macropterum* of I. G. Borshchow in Zap. Imp. Akad. Nauk 7(1): 50. 1865

- IPNI: Cynoglossum macropterum Borszcz. ex Trautv. -- Trudy Imp. S.-Peterburgsk. Bot. Sada ix. (1884) 49. (IK)
- STATUS: nom. inval. [nom. nud.]
- REFERENCE: Link
- COMMENTS: According to Trautvetter (in Trudy Imp. S.-Peterburgsk. Bot. Sada 9: 49. 1884) probably an error for *Cynoglossum macrostylum* Bunge.

---

*Cynoglossum macrostylum* ascribed to Bunge (1847) by Jackson, Index Kew. 1: 687. 1893

- IPNI: Cynoglossum macrostylum Bunge -- Lehm. Reliq. Bot. (1847) 236. (IK)
- STATUS: non-existent (bibliographic error)
- REFERENCE: Link
- COMMENTS: Bunge in 1847 (in Arbeiten Naturf. Vereins Riga 1: 117—253) published only the first part of Lehmann’s *Reliquiae*. The second part, together with a second edition of the first, was published in 1852 under a different main title (Beitr. Fl. Russl.).

---

*Cynoglossum macrostylum* Bunge, Beitr. Fl. Russl.: 236. 1852

- IPNI: Cynoglossum macrostylum Bunge -- Beitr. Fl. Russl. 236 (1852). [7 Nov 1852] (IK)
- ASSESSMENT: [*Cynoglossum anchusoides* Lindl.]
- STATUS: spec. nov.
- REFERENCE: Link
- SYNONYMY:   
  ≡ *Lindelofia macrostyla* (Bunge) Popov 1953
    
  ≡ *Lindelofia anchusoides* subsp. *macrostyla* (Bunge) Kamelin 1975
- BASIS: Original material: Zwischen Buchara und Samarkand an Gräben bei Katte-Kurgan, … auch … in den Thälern des Karatau-Gebirges am obern Sarafschan 31. Aug. 1841 [from protologue]. Syntypes: P (P03512700\*, with Bunge’s handwriting, lectotype designated here by Hilger & Stier; P03512699\*, paralectotype)

---

*Cynoglossum magellense*  Ten., Fl. Napol. 1: 66. 1811–1815

- IPNI: absent (2014-11-05)
- ASSESSMENT: accepted
- STATUS: spec. nov.
- REFERENCE: Link
- SYNONYMY:   
  ≡ *Rindera magellensis* (Ten.) Roem. & Schult. 1819
    
  ≡ *Mattia magellensis* (Ten.) G. Don 1838
- BASIS: Original material not indicated in protologue. Lectotype (Selvi & Cecchi in Taxon 58: 622. 2009): Velino, herb. Tenore (NAP)

---

*Cynoglossum magellense*  of Tenore, Fl. Napol. 3: 183. 1824-1829

- IPNI: Cynoglossum magellense Ten. -- Fl. Napol. 3: 183, t. 117. [1824-1829] (IK)
- STATUS: [isonym]
- REFERENCE: Link

---

*Cynoglossum malabaricum* (C. B. Clarke) Riedl in Oesterr. Bot. Z. 119: 71. 1971

- IPNI: Cynoglossum malabaricum (C.B.Clarke) Riedl -- Oesterr. Bot. Z. 119(1-3): 71. 1971 (IK)
- ASSESSMENT: accepted
- STATUS: comb. nov.
- REFERENCE: Link
- BASIS: Basionym: *Paracaryum malabaricum* C. B. Clarke

---

*Cynoglossum mannii* Baker & C. H. Wright in Thiselton-Dyer, Fl. Trop. Afr. 4(2, 1): 52. 1905

- IPNI: Cynoglossum mannii Baker & C.H.Wright -- Fl. Trop. Afr. [Oliver et al.] 4(2.1): 52. 1905 [Dec 1905] (IK)
- ASSESSMENT: Not *Cynoglossum* s. l.; genus uncertain [*“Cynoglossum” coeruleum* Hochst. ex A. DC.]
- STATUS: spec. nov.
- REFERENCE: Link
- SYNONYMY:   
  ≡ *Cynoglossum lanceolatum* var. *mannii* (Baker & C. H. Wright) Brand 1921
    
  ≡ *Cynoglossum coeruleum* var. *mannii* (Baker & C. H. Wright) Verdc. 1991
- BASIS: Original material: Upper Guinea. Cameroons: Cameroon Mountain. 7000-8000 ft., Mann, 2005? [from protologue]. Syntypes: *Mann 2005*, K (K000418935\*, K000418936\*, K000418937\*)

---

*Cynoglossum marifolium* of Roxburgh, Hort. Bengal.: 13. 1814

- IPNI: Cynoglossum marifolium Roxb. -- Hort. Bengal. 13 (1814), nomen; Fl. Ind., ed. Carey & Wall., ii. 8 (1824); Fl.Ind., ed. Carey, i. 457 (1832). (IK)
- STATUS: nom. inval. [nom. nud.]
- REFERENCE: Link

---

*Cynoglossum marifolium* Roxb., Fl. Ind. 2: 8. 1824

- IPNI: Cynoglossum marifolium Roxb. -- Hort. Bengal. 13 (1814), nomen; Fl. Ind., ed. Carey & Wall., ii. 8 (1824); Fl.Ind., ed. Carey, i. 457 (1832). (IK)
- ASSESSMENT: [*Bothriospermum zeylanicum* (J. Jacq.) Druce]
- STATUS: spec. nov.
- REFERENCE: Link
- BASIS: Original material: eastern parts of Bengal [from protologue]
- COMMENTS: Described without any reference to the earlier *Heliotropium marifolium*, with which it has been synonymised by general agreement following Clarke (in Hooker, Fl. India 4: 152. 1885). However, Roxburgh’s original description does not readily lend itself to such an interpretation (e.g., flowers borne singly between the leaves; corolla with throat closed by scales). The novel interpretation given above will be justified elsewhere (Greuter, in prep.).

---

*Cynoglossum maritimum* of Willdenow [Brand in Engler, Pflanzenr. 78 (IV.252): 107. 1921]

- IPNI: Cynoglossum maritimum Willd. ex Brand -- Pflanzenr. (Engler) Borrag.-Cynogloss. 107 (1921), in syn. (IK)
- STATUS: nom. inval. [pro syn. sub *Omphalodes litoralis*]
- REFERENCE: Link

---

*Cynoglossum maroccanum* of Sennen & Mauricio, Cat. Fl. Rif Orient.: 82. 1933

- IPNI: absent (2014-11-05)
- STATUS: nom. inval. [nom. nud.]
- REFERENCE: Link

---

*Cynoglossum maroccanum* of Sennen, Campagnes Bot. Maroc Or.: 110. 1936

- IPNI: Cynoglossum maroccanum Sennen -- Campagn. Bot. Maroc Or. 1930-5, 110 (1936), in obs., nomen. (IK)
- STATUS: nom. inval. [nom. nud.]
- REFERENCE: Link

---

*Cynoglossum mathezii* Greuter & Burdet in Willdenowia 11: 35. 1981

- IPNI: Cynoglossum mathezii Greuter & Burdet -- Willdenowia 11(1): 35, nom. nov. 1981 (IK)
- ASSESSMENT: accepted
- STATUS: nom. nov.
- REFERENCE: Link
- BASIS: Replaced synonym: *Anchusa lanata* L., non *Cynoglossum lanatum* Lam.

---

*Cynoglossum medium* (Turrill) Greuter & Stier in Biodivers. Data J. [hoc loco]. 2015

- ASSESSMENT: accepted
- STATUS: comb. nov.
- BASIS: Basionym: *Bilegnum medium* Turrill

---

*Cynoglossum meeboldii* Brand in Repert. Spec. Nov. Regni Veg. 14: 323. 1916

- IPNI: Cynoglossum meeboldii Brand -- Repert. Spec. Nov. Regni Veg. 14: 323. 1916 (IK)
- ASSESSMENT: accepted
- STATUS: spec. nov.
- REFERENCE: Link
- BASIS: Original material: Südliches Vorderindien: Kulhutty Bababuden, 2000 m. November 1908, blühend u. fruchtend (Meebold no. 6752, Herb. Breslau; forma glabrata) [from protologue] Presumed holotype: WRSL

---

*Cynoglossum melananthum* Pau, Carta Bot. 3: 6. 1906

- IPNI: absent (2014-11-05)
- ASSESSMENT: accepted
- STATUS: spec. nov. [but see comments]
- REFERENCE: Link
- BASIS: Original material: Reverchon exs. l. c. [Plantes d’Espagne] 1903, 1904 y 1905 [from protologue]
- COMMENTS: Pau describes this taxon as one of four “pequeñas especies” placed under *Cynoglossum valentinum* “Lge.”, one of the four being *C. valentinum* Lag. itself. Two (including the present one) he terms “psp.” (prospecies), rather than species, but as they are coordinate with two species, and all are referred to as “small species” in the preceding paragraph, we consider them to be published as binomials not as ternary (infraspecific) combinations.

---

*Cynoglossum membranaceum* A. DC. in Candolle, Prodr. 10: 150. 1846

- IPNI: Cynoglossum membranaceum A.DC. -- Prodr. [A. P. de Candolle] 10: 150. 1846 [8 Apr 1846] (IK)
- ASSESSMENT: [*Cynoglossum wallichii* G. Don]
- STATUS: spec. nov.
- REFERENCE: Link
- BASIS: Original material: In Emodo inter 10 et 11000 ped. alt. legit cl. Edgeworth; spec. a cl. Edgew. comm. sub n. 394 [from protologue]. Holotype: G-DC (G00205525\*)

---

*Cynoglossum mexicanum* Cham. & Schltdl. in Linnaea 5: 114. 1830

- IPNI: Cynoglossum mexicanum Cham. & Schltdl. -- Linnaea 5: 114. 1830 (IK)
- ASSESSMENT: *Hackelia mexicana*  (Cham. & Schltdl.) I. M. Johnst.
- STATUS: spec. nov.
- REFERENCE: Link
- SYNONYMY:   
  ≡ *Hackelia mexicana*  (Cham. & Schltdl.) I. M. Johnst. 1923
- BASIS: Original material: plantae mexicanae a Schiede et Deppe collectae [from title], in monte Macultepec [from protologue]. Syntypes: GH (Herbarium Specimens\*), HAL (HAL0095748\*, HAL0107370\*)

---

*Cynoglossum micranthum* of Desfontaines, Tabl. École Bot.: 220. 1804

- IPNI: Cynoglossum micranthum Desf. -- Tabl. École Bot. 220. 1804 (IK)
- STATUS: nom. inval. [nom. nud.]
- REFERENCE: Link

---

*Cynoglossum micranthum* Poir. in Lamarck, Encycl. Suppl. 2: 431. 1811

- IPNI: absent (2014-11-05)
- ASSESSMENT: Not *Cynoglossum* s. l.; genus uncertain [*“Cynoglossum” lanceolatum* Forssk.]
- STATUS: nom. illeg. [superfl.]
- REFERENCE: Link
- BASIS: Replaced synonym: *Cynoglossum canescens* Willd.

---

*Cynoglossum micranthum* of Dalzell & A. Gibson, Bombay Fl.: 172. 1861

- IPNI: Cynoglossum micranthum Dalzell & A.Gibson -- Bombay Fl. [Dalzell & Gibson] 172. 1861 (IK)
- STATUS: [isonym]
- REFERENCE: Link

---

*Cynoglossum micranthum* var. *decurrens* Trimen, Handb. Fl. Ceylon 3: 203. 1895

- IPNI: absent (2014-11-05)
- ASSESSMENT: Not *Cynoglossum* s. l.; genus uncertain [*“Cynoglossum” limense* Willd.]
- STATUS: var. nov.
- REFERENCE: Link
- BASIS: Original material: in Himalaya and Nilgiri Mountains (?) [from protologue]
- COMMENTS: The reference to Moon (Cat. Pl. Ceylon: 12. 1824) is to a misapplication of the name of a S. American plant (*Cynoglossum decurrens* Ruiz & Pav.) to a Ceylonese taxon.

---

*Cynoglossum microcarpum* Kern. in Ber. Naturwiss.-Med. Vereins Innsbruck 1: 109. 1870

- IPNI: Cynoglossum microcarpum Kern. -- Nov. Pl. Sp. Dec. ii. 13. (IK)
- ASSESSMENT: [*Cynoglossum wallichii* G. Don]
- STATUS: spec. nov.
- REFERENCE: Link
- BASIS: Original material: In regione montana sup. Himalaja. Lahul. det. Jäschke 1869 [from protologue]

---

*Cynoglossum microglochin* Royle ex Benth. in Royle, Ill. Bot. Himal. Mts.: 305. 1836

- IPNI: Cynoglossum microglochin Benth. -- Ill. Bot. Himal. Mts. [Royle] 305. (IK)
- ASSESSMENT: accepted
- STATUS: spec. nov.
- REFERENCE: Link
- BASIS: Original material: Cashmere [from protologue]

---

*Cynoglossum microglochin* var. *marganum* Brand in Engler, Pflanzenr. 78 (IV.252): 134. 1921

- IPNI: absent (2014-11-05)
- ASSESSMENT: [*Cynoglossum microglochin* Royle ex Benth.]
- STATUS: var. nov.
- REFERENCE: Link
- BASIS: Original material: Kaschmir: Marganpaß, 3000 m ü. M. (Meebold n. 4181, Herb. Breslau) [from protologue]. Presumed holotype: WRSL

---

*Cynoglossum microglochin* var. *nervosum* (Benth. ex C. B. Clarke) Y. J. Nasir in Ali, Fl. Pakistan 191: 163. 1989

- IPNI: Cynoglossum microglochin Benth. var. nervosum (Benth. ex C.B.Clarke) Y.J.Nasir -- Fl. Pakistan 191: 163 (1989):. (IK)
- ASSESSMENT: [*Cynoglossum microglochin* Royle ex Benth.]
- STATUS: comb. nov.
- REFERENCE: Link
- BASIS: Basionym: *Cynoglossum nervosum* Benth. ex C. B. Clarke
- COMMENTS: See under *Cynoglossum petiolatum* var. *nervosum* (Benth. ex C. B. Clarke) Bennet.

---

*Cynoglossum minimum* (Brand) Greuter & Stier in Biodivers. Data J. [hoc loco]. 2015

- ASSESSMENT: accepted
- STATUS: comb. nov.
- BASIS: Basionym: *Solenanthus minimus* Brand

---

*Cynoglossum minutiflorum* Greuter & Stier in Biodivers. Data J. [hoc loco]. 2015

- ASSESSMENT: accepted
- STATUS: nom. nov.
- BASIS: Replaced synonym: *Lindelofia micrantha* Rech. f. & Riedl, non *Cynoglossum micranthum* Poir.

---

*Cynoglossum modestum* (Boiss. & Hausskn.) Greuter & Stier in Biodivers. Data J. [hoc loco]. 2015

- ASSESSMENT: accepted
- STATUS: comb. nov.
- BASIS: Basionym: *Paracaryum modestum* Boiss. & Hausskn.

---

*Cynoglossum modorense* Rech. in Allg. Bot. Z. Syst. 20: 22. 1914, pro. hybr.

- IPNI: Cynoglossum modorense Rechinger -- Allg. Bot. Z. Syst. 1914, xx. 22, hybr. (IK)
- ASSESSMENT: accepted as hybrid
- STATUS: hybr. nov. [*C. germanicum* × *C. officinale*]
- REFERENCE: Link
- BASIS: Original material: Ungarn: Kleine Karpathen, bei Modern (1908) [from protologue]

---

*Cynoglossum moenchii* of hort. tic. [Pavia] [Zuccagni, Syn. Pl. 1801: 20. 1801]

- IPNI: absent (2014-11-05)
- STATUS: nom. inval. [nom. nud., fide Grande in Bull. Orto Bot. Regia Univ. Napoli 4: 171. 1913]
- REFERENCE: Link

---

*Cynoglossum moenchii* of “Nocca. Roem. H. Turic.” [Zürich] [Steudel, Nomencl. Bot., ed. 2, 1: 464. 1840]

- IPNI: Cynoglossum moenchii Nocca & Roem. ex Steud. -- Nomencl. Bot. [Steudel], ed. 2. 1: 464. 1840 (IK)
- STATUS: nom. inval. [pro syn. sub *Cynoglossum dioscoridis*]
- REFERENCE: Link

---

*Cynoglossum moeszii* of Jávorka, Magyar Fl.: 842. 1925, pro hybr. *Cynoglossum hungaricum* x *officinale*

- IPNI: absent (2014-11-05)
- STATUS: nom. inval. [nom. nud.]
- REFERENCE: Link

---

*Cynoglossum molle* Phil. in Linnaea 29: 18. 1858

- IPNI: Cynoglossum molle Phil. -- Linnaea 29: 18. 1858 (IK)
- ASSESSMENT: [*Cynoglossum creticum* Mill.]
- STATUS: spec. nov.
- REFERENCE: Link
- BASIS: Original material: [Chile] Prope Tomé specimina duo in initio antheseos legit orn. Germain [from protologue]. Holotype: SGO (SGO000004031\*)

---

*Cynoglossum mollissimum* Lehm., Pl. Asperif. Nucif.: 165. 1818

- IPNI: Cynoglossum mollissimum Lehm. -- Pl. Asperif. Nucif. 1: 165. 1818 [Jul-early Sep 1818] (IK)
- ASSESSMENT: [*Cynoglossum apenninum* L.]
- STATUS: spec. nov.
- REFERENCE: Link
- SYNONYMY:   
  ≡ *Solenanthus mollissimus*  (Lehm.) DC. & A. DC. 1846
- BASIS: Original material: in Persiae provincia Djilan (v. s.) [from protologue]. Holotype formerly in B-W (†)
- COMMENTS: See Brand (in Engler, Pflanzenr. 78: 154. 1921). See also comments under *Cynoglossum gileadense* Willd..

---

*Cynoglossum monophlebium* Baker in J. Linn. Soc., Bot. 20: 211. 1883

- IPNI: Cynoglossum monophlebium Baker -- J. Linn. Soc., Bot. 20: 211. 1883 [1884 publ. 1883] (IK)
- ASSESSMENT: accepted
- STATUS: spec. nov.
- REFERENCE: Link
- BASIS: Original material: Central Madagascar, Baron 1871! and a monster with fasciated stems three inches broad, Baron 2009! [from protologue]. Lectotype (Miller in Adansonia 27: 122. 2005): Baron 1871, K (K000418909\*); isolectotype: P (P00417686\*); paralectotype: K (K000418910\*)

---

*Cynoglossum montanum* L., Demonstr. Pl.: 5. 1753

- IPNI: Cynoglossum montanum L. -- Demonstr. Pl. 5. 1753 [3 Oct 1753] (IK)
- ASSESSMENT: accepted
- STATUS: spec. nov.
- REFERENCE: Link
- BASIS: Original material: *Cynoglossa media altera, virente folio, rubro flore, montana frigidarum regionum.* Col. Ecphr. 1. p. 176. t.. 175 [from protologue]. Lectotype (Lacaita in *Bull. Orto Bot. Regia Univ. Napoli* 3 : 291. 1913): illustration in Colonna, Ekphrasis: 175. 1616 ; epitype (Selvi in Taxon 57: 297. 2008): Abruzzo, radure e margini di faggeta attorno al Lago di Campotosto (Aquila), 1200 m, suolo arenaceo, 23 Jun 2003, *Bigazzi & Selvi 03.08* (FI)

---

*Cynoglossum montanum* Lam., Fl. Franç. 2: 277. 1779

- IPNI: absent (2014-11-05)
- ASSESSMENT: *Cynoglossum apenninum* L.
- STATUS: nom. illeg. [superfl. and homonym]
- REFERENCE: Link
- BASIS: Replaced synonym: *Cynoglossum apenninum* L.

---

*Cynoglossum montanum* var. *alpinum*  Brand in Engler, Pflanzenr. 78 (IV.252): 127. 1921

- IPNI: absent (2014-11-05)
- ASSESSMENT: *Cynoglossum alpinum* (Brand) Riedl
- STATUS: var. nov.
- REFERENCE: Link
- SYNONYMY:   
  ≡ *Cynoglossum alpinum* (Brand) Riedl 1985
- BASIS: Original material: Abyssinien: auf allen Alpen von 1300 m bis 3500 m ü. M. (Schimper n. 49). Auf Berg Gunna (Schimper n. 1194 vom 15. Sept. 1863). Ohne nähere Angabe (Schimper n. 227). Galla-Gebiet: Ego (Ellenbeck n. 374a); Hararmaja See (Ellenbeck n. 464); Gara Mulata (Ellenbeck n. 549); Schoa: Sequala (Ellenbeck n. 1656) [from protologue]. Lectotype (Riedl in Linzer Biol. Beitr. 17: 318. 1985, as “Holotypus”): *Schimper 49*, W; paralectotypes: *Schimper 227*, K (K000418934\*), *Schimper 1194*, K (K000418933\*)
- COMMENTS: The designation of *Schimper 1194* (K) as lectotype by Burtt, Notes Roy. Bot. Gard. Edinb. 43: 345. 1986, being later, has no standing.

---

*Cynoglossum montanum* var. *asiaticum* Brand in Engler, Pflanzenr. 78 (IV.252): 127. 1921

- IPNI: absent (2014-11-05)
- ASSESSMENT: [*Cynoglossum montanum* L.]
- STATUS: var. nov.
- REFERENCE: Link
- BASIS: Original material: In Vorderasien. In Syrien auf dem Libanon und Antilibanon … am Sannin (Ehrenberg; Kneucker n. 124); im Zedernwalde oberhalb Bscherre (Schweinfurth n. 418; Bornmüller n. 12186). … Pisidien (Heldreich n. 661). Lycien: auf dem Berge Elmalu (Bourgeau). Karien: Tmolus (Boissier). Auf dem Ida (Sintenis n. 689). Bithynien: Keschischdagh (Bornmüller n. 5326). Pontus: Trapezunt (C. Koch). Jildindagh (Bornmüller n. 24 44) usw. Armenien: Kharput: Schuschnas (Sintenis n. 299). Egin (Sintenis n. 2419, fälschlich bestimmt als C. Columnae). Gümüschchane (Sintenis n. 5677). Nördliches Persien: Westlicher Elburs im Tale Lur (Bornmüller n. 7733)

---

*Cynoglossum montanum* subsp. *extraeuropaeum* Brand in Engler, Pflanzenr. 78 (IV.252): 126. 1921

- IPNI: absent (2014-11-05)
- ASSESSMENT: [*Cynoglossum montanum* L.]
- STATUS: subsp. nov.
- REFERENCE: Link
- BASIS: Original material: The almost 40 specimens cited by Brand, under one of the 6 subordinate taxa accepted in the protologue, are all syntypes of the subspecific name. Most are cited under *Cynoglossum montanum* var. *alpinum*  Brand, *Cynoglossum montanum* var. *asiaticum* Brand, *Cynoglossum montanum* var. *latifolium Brand*, *Cynoglossum montanum* var. *maroccanum* Brand, *Cynoglossum nebrodense* var. *natolicum* Bornm. and *Cynoglossum montanum* var. *parvifolium* (K. Koch) Brand. The ditio classica thus includes Morocco, Algeria, Abyssinia and large parts of SW Asia

---

*Cynoglossum montanum* var. *latifolium Brand* in Engler, Pflanzenr. 78 (IV.252): 128. 1921

- IPNI: absent (2014-11-05)
- ASSESSMENT: [*Cynoglossum montanum* L.]
- STATUS: var. nov.
- REFERENCE: Link
- BASIS: Original material: Kurdistan: Mardin: Khurs (Sintenis n. 1053) [from protologue]

---

*Cynoglossum montanum* subsp. *linnaeanum* Brand in Engler, Pflanzenr. 78 (IV.252): 126. 1921

- IPNI: absent (2014-11-05)
- ASSESSMENT: [*Cynoglossum montanum* L.]
- STATUS: subsp. nov.
- REFERENCE: Link
- BASIS: More than 20 syntypes cited in the protologue, from peninsular Italy, Croatia, Bosnia, Greece, Hungary, and Caucasia
- COMMENTS: Even though, from the provenance of the cited specimens, the area given, and particularly the choice of epithet, it is abundantly clear that Brand considers this to be the “typical” subspecies of *Cynoglossum montanum*, he does not explicitly cite either that name itself nor its previously designated type under that subspecies. Therefore *C. montanum* subsp. *linnaeanum*, even though for all events and purposes a synonym of subsp. *montanum*, is a validly published name.

---

*Cynoglossum montanum* var. *maroccanum* Brand in Engler, Pflanzenr. 78 (IV.252): 128. 1921

- IPNI: absent (2014-11-05)
- ASSESSMENT: [*Cynoglossum dioscoridis* Vill.]
- STATUS: var. nov.
- REFERENCE: Link
- SYNONYMY:   
  ≡ *Cynoglossum dioscoridis* var. *maroccanum* (Brand) Maire 1931
- BASIS: Original material: Nordwestafrika: Auf dem Atlas und seinen Vorbergen. Marokko: zwischen Mogador und Marokko (Ball). Gebirge im Südwesten der Stadt Marokko (Ibrahim). Djebel Labgurt (Ibrahim). Im Tale Ait Mesun (Ball): Zweifellos … auch ... Algier: Djurdjura, Behni Sahla, Monzaïa usw. [from protologue]

---

*Cynoglossum montanum* subvar. *natolicum* (Bornm.) Brand in Engler, Pflanzenr. 78 (IV.252): 127. 1921

- IPNI: absent (2014-11-05)
- ASSESSMENT: [*Cynoglossum montanum* L.]
- STATUS: comb. & stat. nov. [variety to subvariety]
- REFERENCE: Link
- BASIS: Basionym: *Cynoglossum nebrodense* var. *natolicum* Bornm.

---

*Cynoglossum montanum* var. *parviflorum* Hayek in Repert Spec. Nov. Regni Veg. Beih. 30(2): 51. 1928

- IPNI: absent (2014-11-05)
- ASSESSMENT: *Cynoglossum pustulatum* subsp. *parvifolium* (Vis.) Sutorý
- STATUS: nom. nov.
- REFERENCE: Link
- BASIS: Replaced synonym: *Cynoglossum officinale* var. *parvifolium* Vis., non *Cynoglossum montanum* var. *parvifolium* (K. Koch) Brand
- COMMENTS: A curious case: by inadvertently using the epithet *parviflorum* instead of the intended, original *parvifolium* of Visiani, Hayek accidentally created a legitimate replacement name instead of the intended, illegitimate junior homonym.

---

*Cynoglossum montanum* var. *parvifolium* (K. Koch) Brand in Engler, Pflanzenr. 78 (IV.252): 128. 1921

- IPNI: absent (2014-11-05)
- ASSESSMENT: [*Cynoglossum montanum* L.]
- STATUS: comb. & stat. nov. [species to variety]
- REFERENCE: Link
- BASIS: Basionym: *Cynoglossum parvifolium* K. Koch

---

*Cynoglossum montbretii* (Riedl) Greuter & Burdet in Willdenowia 11: 35. 1981

- IPNI: Cynoglossum montbretii (Riedl) Greuter & Burdet -- Willdenowia 11(1): 35. 1981 (IK)
- ASSESSMENT: accepted
- STATUS: comb. nov.
- REFERENCE: Link
- BASIS: Basionym: *Mattiastrum montbretii* Riedl

---

*Cynoglossum morisonii* DC., Prodr. 10: 155. 1846 *(‘morisoni’)*

- IPNI: Cynoglossum morisonii DC. -- Prodr. [A. P. de Candolle] 10: 155. 1846 [8 Apr 1846] (IK)
- ASSESSMENT: [*Hackelia virginiana*  (L.) I. M. Johnst.]
- STATUS: spec. nov.
- REFERENCE: Link
- BASIS: Original material: Amer. bor. in Virginiâ (Mor.), Carolinâ (Ell.), Kentucky (Lehm.), Ohio (Frank!) et Canada (Hook. fl. bor. am. 2. p. 83) … Franck ! pl. exs. un. itin. … Moris. ox. s. 11. t. 30. f. 9 [from protologue]. Syntype: *Franck 1898*, G-DC (G00205765\*)
- COMMENTS: The same species as *Myosotis virginiana*  L. (1753; non *Cynoglossum virginianum* L. 1753), but not published as a replacement name for it. Candolle, in the protologue, considered *Myosotis ‘virginica’* L. as doubtfully synonymous. See also comments under *Myosotis virginiana*  L.. Correction of the epithet’s original spelling mandated by ICN Art. 60.12.

---

*Cynoglossum multicaule* (Rech. f. & Riedl) Greuter & Stier in Biodivers. Data J. [hoc loco]. 2015

- ASSESSMENT: accepted
- STATUS: comb. nov.
- BASIS: Basionym: *Mattiastrum multicaule* Rech. f. & Riedl

---

*Cynoglossum muricatum* Thunb., Prodr. Pl. Cap.: 34. 1794

- IPNI: Cynoglossum muricatum Thunb. -- Prodr. Pl. Cap. 1: 34. 1794 (IK)
- ASSESSMENT: *Lithospermum flexuosum* Lehm.
- STATUS: spec. nov.
- REFERENCE: Link
- SYNONYMY:   
  ≡ *Lithospermum flexuosum* Lehm. 1818, nom. nov.
- BASIS: Original material: [S. Africa] [not indicated in protologue]. Presumable holotype: LD (LD1259844\*)

---

*Cynoglossum myosotoides* Labill., Icon. Pl. Syr. 2: 6. 1791

- IPNI: Cynoglossum myosotoides Labill. -- Icon. Pl. Syr. 2: 6, t. 2. 1791 [Jul 1791] (IK)
- ASSESSMENT: *Cynoglossum lithospermifolium* Lam.
- STATUS: nom. illeg. [superfl.]
- REFERENCE: Link
- BASIS: Replaced synonym: *Cynoglossum lithospermifolium* Lam.

---

*Cynoglossum myosotoides* of Schimper [Candolle, Prodr. 10: 157. 1846]

- IPNI: Cynoglossum myosotoides Schimp. ex DC. -- Prodr. [A. P. de Candolle] 10: 157. 1846 [8 Apr 1846] (IK)
- STATUS: nom. inval. [pro syn. sub *Omphalodes rugulosa*]
- REFERENCE: Link

---

*Cynoglossum myosotoides* of Aucher-Eloy [Candolle, Prodr. 10: 155. 1846]

- IPNI: Cynoglossum myosotoides Aucher ex DC. -- Prodr. [A. P. de Candolle] 10: 159. 1846 [8 Apr 1846] (IK)
- STATUS: nom. inval. [pro syn. sub *Omphalodes hirsuta*]
- REFERENCE: Link

---

*Cynoglossum nebrodense* Guss., Fl. Sic. Prodr. 1: 216. 1827

- IPNI: Cynoglossum nebrodense Guss. -- Fl. Sic. Prodr. 1: 216. 1827 [Oct-Dec 1827] (IK)
- ASSESSMENT: accepted
- STATUS: spec. nov.
- REFERENCE: Link
- SYNONYMY:   
  ≡ *Cynoglossum officinale* var. *humile* Ten. 1831
- BASIS: Original material: In sylvis elatioribus montosis; Madonie alle fosse di S. Gandolfo, a Zotta funno, e nella valle della Sciacca [from protologue]. Lectotype (Selvi & Cecchi in Taxon 58: 622. 2009): Madonie alle fosse di S. Gandolfo, 30 giugno, Gussone, herb. siculum, NAP)

---

*Cynoglossum nebrodense* of Jan, Elench. Pl.: 3. 1831

- IPNI: absent (2014-11-05)
- STATUS: nom. inval. [nom. nud.]
- REFERENCE: Link
- COMMENTS: See comments under *Cynoglossum gussonei* Strobl.

---

*Cynoglossum nebrodense* var. *areolatum* Boiss., Fl. Orient. 4: 265. 1875

- IPNI: absent (2014-11-05)
- ASSESSMENT: [*Cynoglossum officinale* L.]
- STATUS: var. nov.
- REFERENCE: Link
- SYNONYMY:   
  ≡ *Cynoglossum officinale* var. *areolatum* (Boiss.) Kusn. 1913
- BASIS: Original material: in Daghestania prope Schoralo 6000’ (Rupr.!) [from protologue]. Presumed holotype: G-BOIS

---

*Cynoglossum nebrodense* subsp. *lucanum* Selvi & Sutorý in Pl. Biosystems [e-publ.] 146: 471. 2012

- IPNI: Cynoglossum nebrodense Guss. subsp. lucanum Selvi & Sutorý -- Pl. Biosystems 146(2): 471. 2012 [4 Apr 2012] [epublished]
- ASSESSMENT: accepted
- STATUS: subsp. nov.
- REFERENCE: Link
- BASIS: Holotype: Basilicata/Calabria: Calabria III orient. Loc. arenos. lapidos. erectis ad montem Pollino, sol. calcar. 17–1800 m, 13.6.1877. Huter, Porta et Rigo, ex Itinere Italico III, n. 377 (FI); isotype in K; Figure 9 [from protologue]

---

*Cynoglossum nebrodense* f. *luxurians* of Popov [Komarov, Fl. SSSR 19: 668. 1953]

- IPNI: absent (2014-11-05)
- STATUS: nom. inval. [sine descr. lat., ICN Art. 39.1]
- REFERENCE: Link

---

*Cynoglossum nebrodense* var. *natolicum* Bornm. in Mitth. Thüring. Bot. Vereins 20: 40. 1905

- IPNI: absent (2014-11-05)
- ASSESSMENT: [*Cynoglossum montanum* L.]
- STATUS: var. nov.
- REFERENCE: Link
- SYNONYMY:   
  ≡ *Cynoglossum montanum* subvar. *natolicum* (Bornm.) Brand 1921
- BASIS: Original material: Amasia (Bornm. Exsicc. No. 751), … Tossia (prope Dikmen 10.V.1892, leg. Sintenis; no 5260), … Ineboli in Paphlagonien (prope Ibras 30.iv.1892 leg. Sint. No. 3735) und Güllek-Tepe in Cilicien (leg. Siehe, no. 415) [from protologue]. Original specimens preumably: B, JE; probable isosyntype: *Bornmüller*, PH (PH00009266)

---

*Cynoglossum nebrodense* var. *pustulatum* (Boiss.) Boiss., Voy. Bot. Espagne 2: 434. 1841

- IPNI: absent (2014-11-05)
- ASSESSMENT: *Cynoglossum pustulatum* Boiss.
- STATUS: stat. nov. [species to variety]
- REFERENCE: Link
- BASIS: Basionym: *Cynoglossum pustulatum* Boiss.

---

*Cynoglossum nebrodense* subsp. *pustulatum* (Boiss.) O. Bolòs & Vigo, Fl. Països Catalans 3: 207. 1995

- IPNI: Cynoglossum nebrodense Guss. subsp. pustulatum (Boiss.) O.Bolòs & Vigo -- Fl. Països Catalans 3: 207 (1995):. (IK)
- ASSESSMENT: *Cynoglossum pustulatum* Boiss.
- STATUS: stat. nov. [species via variety to subspecies]
- REFERENCE: Link
- BASIS: Basionym: *Cynoglossum pustulatum* Boiss.

---

*Cynoglossum nebrodense* subsp. *tenorei* Guss. ex Nyman, Consp. Fl. Eur. 3: 521. 1881 *(‘tenorii’)*

- IPNI: absent (2014-11-05)
- ASSESSMENT: [*Cynoglossum montanum* L.]
- STATUS: subsp. nov.
- REFERENCE: Link
- BASIS: Original material: *C. officinale* Ten. – Ital. mer. Sard. [from protologue]
- COMMENTS: Validated by indirect reference to Tenore’s description of *Cynoglossum officinale* (Fl. Napol. 3: 180. 1824—1826).

---

*Cynoglossum nebulicola* (R. R. Mill) Greuter & Stier in Biodivers. Data J. [hoc loco]. 2015

- ASSESSMENT: accepted
- STATUS: comb. nov.
- BASIS: Basionym: *Adelocaryum nebulicola*  R. R. Mill

---

*Cynoglossum nervosum* Benth. ex C. B. Clarke in Hooker, Fl. Brit. India 4: 158. 1883

- IPNI: Cynoglossum nervosum Benth. ex Hook.f. -- Fl. Brit. India [J. D. Hooker] 4(10): 158. 1883 [Jun 1883] (IK)
- ASSESSMENT: [*Cynoglossum microglochin* Royle ex Benth.]
- STATUS: spec. nov.
- REFERENCE: Link
- SYNONYMY:   
  ≡ *Cynoglossum petiolatum* var. *nervosum* (Benth. ex C. B. Clarke) Bennet 1983
    
  ≡ *Cynoglossum microglochin* var. *nervosum* (Benth. ex C. B. Clarke) Y. J. Nasir 1989
- BASIS: Original material: Western Himalaya; Kulu, Jalauri Pass, Edgeworth; Pangee, alt. 12,000 ft., Dr. Watt [from protologue]. Lectotype (Kazmi in J. Arnold Arbor. 52: 348. 1971, as “type”): *Edgeworth*, K

---

*Cynoglossum nervosum* var. *petiolatum* (Hook.) Kazmi in J. Arnold Arbor. 52: 350. 1971

- IPNI: Cynoglossum nervosum Benth. ex Hook.f. var. petiolatum (Hook.) Kazmi -- J. Arnold Arbor. 52(2): 350. 1971 (IK)
- ASSESSMENT: [*Cynoglossum microglochin* Royle ex Benth.]
- STATUS: comb. & stat. nov. [species to variety]
- REFERENCE: Link
- BASIS: Basionym: *Anchusa petiolata* Hook.
- COMMENTS: Name legitimate but combination inappropriate under the rules of priority.

---

*Cynoglossum neubaueri* (Rech. f.) Greuter & Stier in Biodivers. Data J. [hoc loco]. 2015

- ASSESSMENT: accepted
- STATUS: comb. nov.
- BASIS: Basionym: *Moltkia neubaueri* Rech. f.

---

*Cynoglossum nigrum* (Riedl) Greuter & Stier in Biodivers. Data J. [hoc loco]. 2015

- ASSESSMENT: accepted
- STATUS: comb. nov.
- BASIS: Basionym: *Mattiastrum nigrum*  Riedl

---

*Cynoglossum nitidum* Willd., Enum. Pl.: 181. 1809

- IPNI: Cynoglossum nitidum Willd. -- Enum. Pl. [Willdenow] 1: 181. 1809 [Apr 1809] (IK)
- ASSESSMENT: *Omphalodes nitida*  (Willd.) Hoffmanns. & Link
- STATUS: spec. nov.
- REFERENCE: Link
- SYNONYMY:   
  ≡ *Omphalodes nitida*  (Willd.) Hoffmanns. & Link 1810
- BASIS: Original material: *Omphalodes nitida*. Hoffmannsegg. & Link. fl. portug. … t. 25. Habitat in Lusitania [from protologue]. Original specimen: B-W (BW03343020\*)
- COMMENTS: See under *Omphalodes nitida*  (Willd.) Hoffmanns. & Link and *Omphalodes lusitanica*  (L.) Schrank.

---

*Cynoglossum nobile* Hook. f. in Gard. Chron. 1858: 240. 1858

- IPNI: Cynoglossum nobile Hook.f. -- Gard. Chron. 1858(13): 240. [27 Mar 1858] (IK)
- ASSESSMENT: [*Myosotidium hortensia*  (Decne.) Baill.]
- STATUS: spec. nov.
- REFERENCE: Link
- SYNONYMY:   
  ≡ *Myosotidium nobile*  (Hook. f.) Hook. 1859
- BASIS: Original material: Chatham Islands, east of New Zealand (S. lat 44°) (exemplar mancum) ... exhibited by Mr. Watson, of St. Albann’s, at the last meeting of the Horticultural Society. [from protologue]. Neotype (Heenan & Schönberger in New Zealand J. Bot. 47: 123-124. 2009)): Illustration in Curtis's Bot. Mag. 85: t. 5137. 1859. Type specimen: Specimen provided by Mr. Watson. Neotype (provided by Heenan & Schönberger N. Z. J. Bot. 47: 124 (2009): Illustration provided by Hooker (1859)

---

*Cynoglossum novoguineense* Riedl in Linzer Biol. Beitr. 22: 307. 1990 *(‘nova-guineese’)*

- IPNI: Cynoglossum nova-guineense Riedl -- Linzer Biol. Beitr. 22(1): 307 (1990). (IK)
- ASSESSMENT: accepted
- STATUS: spec. nov.
- REFERENCE: Link
- BASIS: Original material: Papua-Neuguinea: in monte Maneao, 22.6.1956. CRUTTWELL no. 754 (Holotypus, K); in declivibus septentrionalibus montis Dayman in montibus Maneao. 18.5.1953, BRASS no. 22229 (LAE, A) [from protologue]. Holotype: *Cruttwell* 754, K

---

*Cynoglossum nuttallii* Spreng., Syst. Veg. 1: 566. 1824

- IPNI: Cynoglossum nuttallii Spreng. -- Syst. Veg. (ed. 16) [Sprengel] 1: 565. 1824 [dated 1825; publ. in late 1824] (IK)
- ASSESSMENT: [*Pectocarya penicillata*  (Hook. & Arn.) A. DC.]
- STATUS: spec. nov.
- REFERENCE: Link
- SYNONYMY:   
  ≡ *Pectocarya lateriflora* var. *nuttallii* (Spreng.) Brand 1921
- BASIS: Original material: Ad fl. Missuri. (C. pilosum? Nutt.) [from protologue]

---

*Cynoglossum oblongifolium* (Popov) Greuter & Stier in Biodivers. Data J. [hoc loco]. 2015

- ASSESSMENT: accepted
- STATUS: comb. nov.
- BASIS: Basionym: *Rindera oblongifolia* Popov

---

*Cynoglossum obtusicalyx* Retief & A. E. van Wyk in S. African J. Bot. 62: 169. 1996

- IPNI: Cynoglossum obtusicalyx Retief & A.E.van Wyk -- S. African J. Bot. 62(3): 169 (1996). (IK)
- ASSESSMENT: accepted
- STATUS: spec. nov.
- REFERENCE: Link
- BASIS: Original material: Western Cape Province: 20 km E of Ceres, Renosterveld of hills, locally frequent on S. aspect in steep kloof, 21 October 1958, Acocks 19893 (PRE, holotypus; NBG, isotypus) [from protologue]. Holotype: PRE (PRE0138668-0\*); isotype: NBG

---

*Cynoglossum occidentale* A. Gray in Proc. Amer. Acad. Arts 10: 58. 1874

- IPNI: Cynoglossum occidentale A.Gray -- Proc. Amer. Acad. Arts x. (1874) 58. (IK)
- ASSESSMENT: Not *Cynoglossum* s. l.; genus uncertain (species accepted)
- STATUS: spec. nov.
- REFERENCE: Link
- BASIS: Original material: Sierra Nevada, in the north-eastern part of California, Rev. Mr. Burgess, and Sierra County, J. G. Lemmon [from protologue]. Syntypes: GH (GH00096673\*), NY (NY00335203\*)

---

*Cynoglossum ochroleucum* (Kar. & Kir.) Greuter & Stier in Biodivers. Data J. [hoc loco]. 2015

- ASSESSMENT: accepted
- STATUS: comb. nov.
- BASIS: Basionym: *Rindera ochroleuca* Kar. & Kir.

---

*Cynoglossum officinale* L., Sp. Pl.: 134. 1753

- IPNI: Cynoglossum officinale L. -- Sp. Pl. 1: 134. 1753 [1 May 1753] (IK)
- ASSESSMENT: accepted
- STATUS: spec. nov.
- REFERENCE: Link
- SYNONYMY:   
  ≡ *Cynoglossum ruderale* Salisb. 1796, nom. illeg.
- BASIS: Original material: *Cynoglossum foliis ovato-lanceolatis*. Hort. Cliff. *47*. *Cynoglossum majus vulgare*. Bauh. Pin. *257*. in Europae ruderatis [from protologue]. Lectotype (Verdcourt in Regnum Veg. 127: 40. 1993): Herb. Clifford: 47, *Cynoglossum* 1 (BM-000557917\*)

---

*Cynoglossum officinale* of Desfontaines, Fl. Atlant. 1: 158. 1798

- IPNI: Cynoglossum officinale Desf. -- Fl. Atlant. 1: 158. 1798 (IK)
- STATUS: [isonym]
- REFERENCE: Link

---

*Cynoglossum officinale* of Brotero, Fl. Lusit. 1: 295. 1804

- IPNI: Cynoglossum officinale Brot. -- Fl. Lusit. 1: 295. 1804 (IK)
- STATUS: [isonym]
- REFERENCE: Link

---

*Cynoglossum officinale* of Hooker & Arnott, Bot. Beechey Voy.: 152. 1833

- IPNI: Cynoglossum officinale Hook. & Arn. -- Bot. Beechey Voy. 152. 1833 [Oct 1833] (IK)
- STATUS: [isonym]
- REFERENCE: Link

---

*Cynoglossum officinale* of Willkomm in Flora 35: 217. 1852

- IPNI: Cynoglossum officinale Willk. -- Flora 35: 217. 1852 (IK)
- STATUS: [isonym]
- REFERENCE: Link

---

*Cynoglossum officinale* var. *albiflorum* Opiz in Berchtold & al., Oekon.-Techn. Fl. Böhm. 2(2): 157. 1839

- IPNI: absent (2014-11-05)
- ASSESSMENT: [*Cynoglossum officinale* L.]
- STATUS: var. nov.
- REFERENCE: Link
- BASIS: Original material: described from Bohemia [book title], no locality given in protologue
- COMMENTS: For the uppermost infraspecific category used in this work, designated by lower-case Greek letters, the authors occasionally (e.g. in a Note on p. 158), and as it seems consistently, use the term “Abart”, which is the 19th Century German equivalent of varietas.

---

*Cynoglossum officinale* var. *angustifolium* Cariot, Étude Fl., ed. 5, 2: 443. 1872 *(‘angustifolia’)*

- IPNI: absent (2014-11-05)
- ASSESSMENT: [*Cynoglossum officinale* L.]
- STATUS: var. nov.
- REFERENCE: Link
- BASIS: Original material: Au-dessus de Couzon, au pied des carriers [from protologue]

---

*Cynoglossum officinale* var. *areolatum* (Boiss.) Kusn. in Kuznecov & al., Fl. Caucas. Crit. 4(2): 129. 1913

- IPNI: absent (2014-11-05)
- ASSESSMENT: [*Cynoglossum officinale* L.]
- STATUS: comb. nov.
- REFERENCE: Link
- BASIS: Basionym: *Cynoglossum nebrodense* var. *areolatum* Boiss.

---

*Cynoglossum officinale* var. *bicolor* (Willd.) Lehm., Pl. Asperif. Nucif.: 153. 1818

- IPNI: absent (2014-11-05)
- ASSESSMENT: [*Cynoglossum officinale* L.]
- STATUS: comb. & stat. nov. [species to variety]
- REFERENCE: Link
- BASIS: Basionym: *Cynoglossum bicolor* Willd.

---

*Cynoglossum officinale* var. *bicolor* Cariot, Étude Fl., ed. 5, 2: 443. 1872

- IPNI: absent (2014-11-05)
- ASSESSMENT: [*Cynoglossum officinale* L.]
- STATUS: nom. illeg. [homonym]
- REFERENCE: Link
- BASIS: Original material: none indicated in protologue

---

*Cynoglossum officinale* f. *bicolor* (Willd.) Asch. & Graebn., Fl. Nordostdeut. Flachl.: 573. 1899

- IPNI: absent (2014-11-05)
- ASSESSMENT: [*Cynoglossum officinale* L.]
- STATUS: stat. nov. [species via variety to forma]
- REFERENCE: Link
- BASIS: Basionym: *Cynoglossum bicolor* Willd.

---

*Cynoglossum officinale* subvar. *bicolor* (Willd.) Rouy, Fl. France 10: 339. 1908

- IPNI: absent (2014-11-05)
- ASSESSMENT: [*Cynoglossum officinale* L.]
- STATUS: stat. nov. [species via variety to subvariety]
- REFERENCE: Link
- BASIS: Basionym: *Cynoglossum bicolor* Willd.

---

*Cynoglossum officinale* var. *canescens* as ascribed to Tenore, Fl. Napol. 3: 180. 1824–1829

- IPNI: absent (2014-11-05)
- STATUS: no name [ICN Art. 35.2]
- REFERENCE: Link
- COMMENTS: From the typography (italic rather than roman type) and the fact that Tenore further down consistently refers to the taxon as “var. A” it is clear that the words “Canescens. Tomentosum” are here used to describe an unnamed variety. See also comments under *Cynoglossum officinale* var. *minus* of Tenore.

---

*Cynoglossum officinale* var. *canescens* Ten., Syll. Pl. Fl. Neapol.: 82. 1831

- IPNI: absent (2014-11-05)
- ASSESSMENT: [*Cynoglossum officinale* L.]
- STATUS: var. nov.
- REFERENCE: Link
- BASIS: Original material: not specified in protologue
- COMMENTS: Contrary to the treatment in Tenore’s *Flora napolitana*, varietal epithets in the *Sylloge* (if any) are in italics but are set off by a semicolon from the descriptive text. In this case, *canescens* is the epithet and *tomentosum* the validating description.

---

*Cynoglossum officinale* f. *canescens* Domin in Sitzungsber. Königl. Böhm. Ges. Wiss., Math.-Naturwiss. Cl. 1902(58): 34. 1903

- IPNI: absent (2014-11-05)
- ASSESSMENT: [*Cynoglossum officinale* L.]
- STATUS: nom. illeg. [homonym, ICN Art. 53.4]
- REFERENCE: Link
- BASIS: Original material: [Czech Republic] Radotín , Karlstein , Srbsko [Domin] [from protologue]

---

*Cynoglossum officinale* var. *collinum* Vis., Fl. Dalmat. 2: 240. 1847

- IPNI: absent (2014-11-05)
- ASSESSMENT: [*Cynoglossum officinale* L.]
- STATUS: var. nov.
- REFERENCE: Link
- BASIS: Original material: Icon. SPENN. in NEES gen. pl. xvii. n. 5. Sow.engl. bot. pl. 92. f. 1. MATTH. sopra Diosc. ed. cit. p. 1250 [from protologue] vidi spec. orig. in herb Visianii [Degen 1937 under *C. parvifolium* (Vis.) Degen, Fl. Veleb. 2: 566. 1937]

---

*Cynoglossum officinale* var. *corsicum* E. Rev. ex Brand in Engler, Pflanzenr. 78 (IV.252): 118. 1921

- IPNI: absent (2014-11-05)
- ASSESSMENT: [*Cynoglossum barbaricinum* Arrigoni & Selvi]
- STATUS: var. nov.
- REFERENCE: Link
- BASIS: Original material: Korsika: Evisa (Reverchon 7./5., 6./7. 85). Im Buchenwalde bei La Fove de Vizzavoni (Kuegler) [from protologue]. Syntypes: Reverchon in Baenitz, Herb. Eur., B, BM, BR, E, FI, FR (FR00360780!, FR00360781!), K, MPU, P (Selvi & Sutorý in Pl. Biosystems 146: 476. 2012). Lectotype (designated here): Fl. Corsica: Evisa, [6 July] 1885, *Reverchon* in Baenitz, Herbarium europaeum, FR (FR00360781!)
- COMMENTS: Selvi & Sutorý (l.c.) do not consider this name to be validly published. Indeed it is not accompanied by a proper description. Brand writes: Flores ut in *C. creticum*; fructus ut in *C. officinale*. We accept this to be an unambiguous reference to the simultaneously published descriptions of the flowers and fruits, respectively, of the two named species.

---

*Cynoglossum officinale* [unranked] *dentatum* Lapeyr., Hist. Pl. Pyrénées: 87. 1813

- IPNI: absent (2014-11-05)
- ASSESSMENT: [*Cynoglossum officinale* L.]
- STATUS: var. nov. [ICN Art. 37.4]
- REFERENCE: Link
- BASIS: Original material: au Pic de Gard

---

*Cynoglossum officinale* var. *eglochidiatum* Domin in Sitzungsber. Königl. Böhm. Ges. Wiss., Math.-Naturwiss. Cl. 1902(58): 34. 1903

- IPNI: absent (2014-11-05)
- ASSESSMENT: [*Cynoglossum officinale* L.]
- STATUS: var. nov.
- REFERENCE: Link
- BASIS: Original material: [Czech Republic] Strakonice (Velenovský, Veselý), Buschberg bei Liebshausen , Radotín , Karlstein , Srbsko [Domin] [from protologue]

---

*Cynoglossum officinale* var. *glochidiatum* Domin in Sitzungsber. Königl. Böhm. Ges. Wiss., Math.-Naturwiss. Cl. 1902(58): 34. 1903

- IPNI: absent (2014-11-05)
- ASSESSMENT: [*Cynoglossum officinale* L.]
- STATUS: var. nov.
- REFERENCE: Link
- BASIS: Original material: [Czech Republic] Laun, Kožov, Vorlík, Kamaik [Domin] [from protologue]

---

*Cynoglossum officinale* var. *humile* Ten., Succ. Relaz. Viaggio Abruzzo: 50. 1830

- IPNI: absent (2014-11-05)
- ASSESSMENT: [*Cynoglossum nebrodense* Guss.]
- STATUS: nom. nov.
- REFERENCE: Link
- BASIS: Replaced synonym: *Cynoglossum nebrodense* Guss.
- COMMENTS: For additional information see: *Cynoglossum officinale* var. *minus* of Tenore.

---

*Cynoglossum officinale* f. *hybridum* (Thuill.) Brand in Engler, Pflanzenr. 78 (IV.252): 118. 1921

- IPNI: absent (2014-11-05)
- ASSESSMENT: [*Cynoglossum officinale* L.]
- STATUS: comb. & stat. nov. [species to forma]
- REFERENCE: Link
- BASIS: Basionym: *Cynoglossum hybridum* Thuill.
- COMMENTS: Brand erroneously cites the basionym from the first edition (1790) of Thuillier’s Flora rather than from the second (1799).

---

*Cynoglossum officinale* [unranked] *macranthum* Opiz in Berchtold & al., Oekon.-Techn. Fl. Böhm. 2(2): 156. 1839

- IPNI: absent (2014-11-05)
- ASSESSMENT: [*Cynoglossum officinale* L.]
- STATUS: infravar. nov.
- REFERENCE: Link
- BASIS: Original material: *Cynoglossum officinale* Kostelecký im Prager gemein. Herbar *F. E. Dittrich*! in sched.
- COMMENTS: In this Flora an unconventional system of infravarietal classification is used, with sometimes several hierarchically arranged, unnamed ranks.

---

*Cynoglossum officinale* [unranked] *micranthum* Opiz in Berchtold & al., Oekon.-Techn. Fl. Böhm. 2(2): 156. 1839

- IPNI: absent (2014-11-05)
- ASSESSMENT: [*Cynoglossum officinale* L.]
- STATUS: infravar. nov. [homonym, non Opiz, l.c.: 157]
- REFERENCE: Link
- BASIS: Original material: *Cynoglossum officinale Ed. Erxleben!* im gemeinsch. Herbar Prags
- COMMENTS: In this Flora an unconventional system of infravarietal classification is used, with sometimes several hierarchically arranged, unnamed ranks. Repetitive use of the same epithet for different but identically characterised taxa, as here, is not uncommon. Only one of these names can be used, in abidance to the decision of a (future?) first reviser (ICN Art. 53.6).

---

*Cynoglossum officinale* [unranked] *micranthum* Opiz in Berchtold & al., Oekon.-Techn. Fl. Böhm. 2(2): 157. 1839

- IPNI: absent (2014-11-05)
- ASSESSMENT: [*Cynoglossum officinale* L.]
- STATUS: infravar. nov. [homonym, non Opiz, l.c.: 156]
- REFERENCE: Link
- BASIS: Original material: *Opiz!* herb. n. 6033 (1810) [from protologue]
- COMMENTS: In this Flora an unconventional system of infravarietal classification is used, with sometimes several hierarchically arranged, unnamed ranks. Repetitive use of the same epithet for different but identically characterised taxa, as here, is not uncommon. Only one of these names can be used, in abidance to the decision of a (future?) first reviser (ICN Art. 53.6).

---

*Cynoglossum officinale* [unranked] *microcarpum* Sennen, Diagn. Nouv.: 25. 1936

- IPNI: absent (2014-11-05)
- ASSESSMENT: [*Cynoglossum officinale* L.]
- STATUS: „var. vel subsp.“ nov.
- REFERENCE: Link
- BASIS: Original material: Cerdagne: Estavar, Angoustrine, Val de Llo, etc. 1220–1700 m. environ [from protologue]. Syntype: *Sennen 6631*; original specimen: Cerdagne, Estavar, 24 Jun 1924, *Sennen s.n.*, PH (PH00009260\*)

---

*Cynoglossum officinale* var. *minus* of Tenore, Fl. Napol. 3: 180. 1824–1829 *(‘minor’)*

- IPNI: absent (2014-11-05)
- STATUS: nom. inval. [nom. nud.]
- REFERENCE: Link
- COMMENTS: In Tenore’s *C. officinale* var. *‘minor’* the latter word appears as a true epithet, not as a descriptive term as in the preceding var. A (see comments under *Cynoglossum officinale* var. *canescens* as ascribed to Tenore). However, the intended name was not validly publihed because the associated description of Sicilian plants, borrowed from Gussone’s *Cynoglossum nebrodense* Guss. (Fl. Sic. Prodr. 1: 216. 1827) was considered to doubtfully apply to the new variety (from Aspromonte in Calabria). Soon after, Tenore used the same name and description of Gussone, without question mark, to validate the name *C. officinale* var. *humile*, intended to designate a different plant from Abruzzo.

---

*Cynoglossum officinale* var. *montanum* Moris, Fl. Sardoa 3: 112. 1858–1859

- IPNI: absent (2014-11-05)
- ASSESSMENT: [*Cynoglossum germanicum* Jacq.]
- STATUS: nom. illeg. [superfl.]
- REFERENCE: Link
- BASIS: Replaced synonym: *Cynoglossum sylvaticum* Haenke (≡ *Cynoglossum officinale* var. *sylvaticum* (Haenke) Willd.)

---

*Cynoglossum officinale* var. *parvifolium* Vis., Fl. Dalmat. 2: 240. 1847

- IPNI: absent (2014-11-05)
- ASSESSMENT: *Cynoglossum pustulatum* subsp. *parvifolium* (Vis.) Sutorý
- STATUS: var. nov.
- REFERENCE: Link
- SYNONYMY:   
  ≡ *Cynoglossum parvifolium* (Vis.) Degen 1937, non K. Koch 1849
    
  ≡ *Cynoglossum pustulatum* subsp. *parvifolium* (Vis.) Sutorý 1989
- BASIS: Original material: in montibus Vellebith [from protologue]. Lectotype (Selvi & Cecchi in Taxon 58: 622. 2009): in mont. Vellebith, *Visiani* (PAD)

---

*Cynoglossum officinale* f. *paucisetum* (Borbás) Jáv., Magyar Fl.: 842. 1925

- IPNI: absent (2014-11-05)
- ASSESSMENT: [*Cynoglossum officinale* L.]
- STATUS: comb. & stat. nov. [species to forma]
- REFERENCE: Link
- BASIS: Basionym: *Cynoglossum paucisetum* Borbás

---

*Cynoglossum officinale* var. *purpurascens* of Popov [Komarov, Fl. SSSR 19: 672. 1953]

- IPNI: absent (2014-11-05)
- STATUS: nom. inval. [sine descr. lat., ICN Art. 39.1]
- REFERENCE: Link

---

*Cynoglossum officinale* var. *purpureum* Opiz in Berchtold & al., Oekon.-Techn. Fl. Böhm. 2(2): 156. 1839

- IPNI: absent (2014-11-05)
- ASSESSMENT: [*Cynoglossum officinale* L.]
- STATUS: nom. illeg. [superfl.]
- REFERENCE: Link
- BASIS: Replaced synonym: *Cynoglossum bicolor* Willd. (≡ *Cynoglossum officinale* var. *bicolor* (Willd.) Lehm.)
- COMMENTS: As to rank, see comment under *Cynoglossum officinale* var. *albiflorum* Opiz.

---

*Cynoglossum officinale* var. *scabrifolium* Willk. in Willkomm & Lange Prodr. Fl. Hispan. 2: 509. 1870 *(‘scabrifolia’)*

- IPNI: absent (2014-11-05)
- ASSESSMENT: [*Cynoglossum officinale* L.]
- STATUS: var. nov.
- REFERENCE: Link
- BASIS: Original material: in Catal. (c. Berga, Lérida. CSTA.!) atque in agro Madrit. (ad casa de Campo, CUT., LGE.! forma albiflora) [from protologue]
- COMMENTS: The phrase “forma albiflora” is used colloquially (a white-flowered form), not as the epithet in the name of a forma.

---

*Cynoglossum officinale* var. *sempervirens* Roth, Tent. Fl. Germ. 2: 218. 1789

- IPNI: absent (2014-11-05)
- ASSESSMENT: [*Cynoglossum officinale* L.]
- STATUS: var. nov.
- REFERENCE: Link
- BASIS: Original material: none indicated in protologue

---

*Cynoglossum officinale* var. *subglabrum* Mérat, Nouv. Fl. Env. Paris: 73. 1812 *(‘subglaber’)*

- IPNI: absent (2014-11-05)
- ASSESSMENT: [*Cynoglossum officinale* L.]
- STATUS: var. nov.
- REFERENCE: Link
- BASIS: Original material: à Saint-Germain-en-Laye, au Val [from protologue]

---

*Cynoglossum officinale* var. *sylvaticum* (Haenke) Willd., Sp. Pl. 1: 760. 1798

- IPNI: absent (2014-11-05)
- ASSESSMENT: [*Cynoglossum germanicum* Jacq.]
- STATUS: comb. & stat. nov. [species to variety]
- REFERENCE: Link
- BASIS: Basionym: *Cynoglossum sylvaticum* Haenke

---

*Cynoglossum officinale* var. *transiens* of Domin in Sitzungsber. Königl. Böhm. Ges. Wiss., Math.-Naturwiss. Cl. 1902v: 34. 1903

- IPNI: absent (2014-11-05)
- STATUS: nom. inval. [ICN Art. 26.2]
- REFERENCE: Link
- COMMENTS: In the protologue, Domin writes: “wobei man die häufigste var. *transiens* als typische Form anzusehen hat“. Even though “typical” in this context, is more likely to mean “characteristic” than “including the type of the species name”, we opt for a formalistic approach and consider the name as not validly published.

---

*Cynoglossum officinale* var. *umbrosum* Car. in Parlatore, Fl. Ital. 6: 849. 1886

- IPNI: absent (2014-11-05)
- ASSESSMENT: [*Cynoglossum germanicum* Jacq.]
- STATUS: nom. illeg. [superfl.]
- REFERENCE: Link
- BASIS: Replaced synonym: *Cynoglossum sylvaticum* Haenke (≡ *Cynoglossum officinale* var. *sylvaticum* (Haenke) Willd.)

---

*Cynoglossum officinale* var. *villosum* Ten., Syll. Pl. Fl. Neapol.: 82. 1831

- IPNI: absent (2014-11-05)
- ASSESSMENT: [*Cynoglossum montanum* L.]
- STATUS: var. nov.
- REFERENCE: Link
- BASIS: Original material: Col. Ecphr. 176. t. 175. in … Aprutii Monte della Stella. Original element: illustration in  Colonna, Ekphrasis: 175. 1616)
- COMMENTS: See note under *Cynoglossum officinale* var. *canescens* Ten. (1831). In this case, *villosum* is the epithet and *pallide virens* the validating description. The illustration in Colonna has become the lectotype of *Cynoglossum montanum* L..

---

*Cynoglossum officinale* var. *virens* Rouy, Fl. France 10: 339. 1908

- IPNI: absent (2014-11-05)
- ASSESSMENT: [*Cynoglossum germanicum* Jacq.]
- STATUS: nom. illeg. [superfl.]
- REFERENCE: Link
- BASIS: Replaced synonym: *Cynoglossum sylvaticum* Haenke (≡ *Cynoglossum officinale* var. *sylvaticum* (Haenke) Willd.)

---

*Cynoglossum officinale* f. *viride* Domin in Sitzungsber. Königl. Böhm. Ges. Wiss., Math.-Naturwiss. Cl. 1902(58): 34. 1903

- IPNI: absent (2014-11-05)
- ASSESSMENT: [*Cynoglossum officinale* L.]
- STATUS: f. nov.
- REFERENCE: Link
- BASIS: Original material: [Czech Republic] Strakonice (Velenovský, Veselý), Buschberg bei Liebshausen [Domin] [from protologue]

---

*Cynoglossum officinale* var. *vulgare* of Moris, Fl. Sardoa 3: 111. 1858–1859

- IPNI: absent (2014-11-05)
- STATUS: nom. inval. [ICN Art. 26.2]
- REFERENCE: Link

---

*Cynoglossum olgae* (Regel & Smirn.) Greuter & Stier in Biodivers. Data J. [hoc loco]. 2015

- ASSESSMENT: accepted
- STATUS: comb. nov.
- BASIS: Basionym: *Solenanthus olgae* Regel & Smirn.

---

*Cynoglossum omphalodes* of Linnaeus, Sp. Pl., ed. 2: 193. 1762

- IPNI: Cynoglossum omphalodes L. -- Syst. Nat., ed. 10. 2: 914. 1759 [7 Jun 1759] (IK)
- STATUS: [isonym]
- REFERENCE: Link
- COMMENTS: The current IPNI citation is in error: Linnaeus in 1759 abbreviated the epithet to *“Omphal.”*. (see Greuter & al. in Taxon 63: 435. 2014)

---

*Cynoglossum omphaloides* L., Sp. Pl.: 135. 1753

- IPNI: Cynoglossum omphaloides L. -- Sp. Pl. 1: 135. 1753 [1 May 1753] (IK)
- ASSESSMENT: *Omphalodes omphaloides* (L.) Voss nom. rej. prop.; *Omphalodes verna*  Moench, nom. cons. prop.
- STATUS: spec. nov.
- REFERENCE: Link
- SYNONYMY:   
  ≡ *Omphalodes verna*  Moench 1794, nom. cons. prop.
    
  ≡ *Cynoglossum cheirifolium* var. *stylosum* Brand 1796
    
  ≡ *Omphalodes omphaloides* (L.) Voss 1895, nom. rej. prop.
- BASIS: Original material: Hort. Cliff. 47. Moris. hist. … s. 11. t. 26. f. 3; in Lusitaniae nemorosis [from protologue]. Lectotype (Selvi in Taxon 53: 802. 2004): Herb. Clifford: 47, *Cynoglossum* 4, BM (BM-000557924\*)
- COMMENTS: For details, see Greuter & al. (in Taxon 63: 435–436. 2014).

---

*Cynoglossum oschense* (Popov) Greuter & Stier in Biodivers. Data J. [hoc loco]. 2015

- ASSESSMENT: accepted
- STATUS: comb. nov.
- BASIS: Basionym: *Rindera oschensis* Popov

---

*Cynoglossum ovatifolium* Griseb. in Abh. Königl. Ges. Wiss. Göttingen 24: 271. 1879

- IPNI: Cynoglossum ovatifolium Griseb. -- Abh. Königl. Ges. Wiss. Göttingen 24: 271. 1879 (IK)
- ASSESSMENT: [*Hackelia revoluta*  (Ruiz & Pav.) I. M. Johnst.]
- STATUS: spec. nov.
- REFERENCE: Link
- BASIS: Original material: T. [Argentinia, Prov. Tucumán], Quebrada de Siambon [from protologue]. Holotype: *Lorentz & Hieronymus 1043*, GOET (GOET004435\*); isotype: CORD (CORD00006105\*)

---

*Cynoglossum ovatum* of Moon, Cat. Pl. Ceylon: 12. 1824

- IPNI: Cynoglossum ovatum Moon -- Cat. Pl. Ceylon. 12. 1824 (IK)
- STATUS: nom. inval. [nom. nud.]
- REFERENCE: Link

---

*Cynoglossum pallidiflorum* Grecescu, Consp. Fl. Roman.: 413. 1898

- IPNI: Cynoglossum pallidiflorum Grecescu -- Consp. Fl. Romaniei 413 (1898). (IK)
- ASSESSMENT: [*Cynoglossum creticum* Mill.]
- STATUS: spec. nov.
- REFERENCE: Link
- BASIS: Original material: [Romania], Mogoşóea; Comana [from protologue]

---

*Cynoglossum paniculatum* Hook. & Arn., Bot. Beechey Voy.: 37. 1830

- IPNI: Cynoglossum paniculatum Hook. & Arn. -- Bot. Beechey Voy. 37. 1830 [Dec 1830] (IK)
- ASSESSMENT: Not *Cynoglossum* s. l.; genus uncertain (species accepted)
- STATUS: spec. nov.
- REFERENCE: Link
- BASIS: Original material: [Chile], Conception . Mr. Macrae has sent specimens to the Horticultural Society, which he gathered at Murillo Bay in Peru [from protologue]. Syntypes: Conception, *Beechey*, K (K000573727\*); Murillo Bay, Peru, *Macrae*, K (K000573726\*)

---

*Cynoglossum paniculatum* var. *azocartii* (Phil.) Reiche in Anales Univ. Chile 121: 249. 1907 *(‘azocarti’)*

- IPNI: Cynoglossum paniculatum var. azocarti Reiche -- Anales Univ. Chile 121: 249. 1907 (GCI)
- ASSESSMENT: Not *Cynoglossum* s. l.; genus uncertain [*“Cynoglossum” paniculatum* Hook. & Arn.]
- STATUS: comb. & stat. nov. [species to variety]
- REFERENCE: Link
- BASIS: Basionym: *Cynoglossum azocartii* Phil.

---

*Cynoglossum paniculatum* f. *azocartii* (Phil.) Brand in Engler, Pflanzenr. 78 (IV.252): 137. 1921 *(‘azocarti’)*

- IPNI: Cynoglossum paniculatum f. azocarti Brand -- Pflanzenr. (Engler) 4, Fam. 252: 137. 1921 (GCI)
- ASSESSMENT: Not *Cynoglossum* s. l.; genus uncertain [*“Cynoglossum” paniculatum* Hook. & Arn.]
- STATUS: stat. nov. [species via variety to forma]
- REFERENCE: Link
- BASIS: Basionym: *Cynoglossum azocartii* Phil.

---

*Cynoglossum paniculatum* f. *philippianum* Brand in Engler, Pflanzenr. 78 (IV.252): 137. 1921

- IPNI: Cynoglossum paniculatum f. philippianum Brand -- Pflanzenr. (Engler) 4, Fam. 252: 137. 1921 (GCI)
- ASSESSMENT: Not *Cynoglossum* s. l.; genus uncertain [*“Cynoglossum” paniculatum* Hook. & Arn.]
- STATUS: f. nov.
- REFERENCE: Link
- BASIS: Original material: Chile: Cordillere von Chillan (Philippi n. 698). Concepcion (Neger). In Gebüschen beim Landgut San Juan (Philippi n. 318) [from protologue]

---

*Cynoglossum paphlagonicum* (Bornm.) Greuter & Burdet in Willdenowia 11: 35. 1981

- IPNI: Cynoglossum paphlagonicum (Bornm.) Greuter & Burdet -- Willdenowia 11(1): 35. 1981 (IK)
- ASSESSMENT: accepted
- STATUS: comb. nov.
- REFERENCE: Link
- BASIS: Basionym: *Mattiastrum paphlagonicum* Bornm.

---

*Cynoglossum papillosum* (Thunb.) Thunb., Fl. Cap. 2: 352. 1820; Fl. Cap., ed. [2]: 162. 1823

- IPNI: Cynoglossum papillosum Thunb. -- Fl. Cap. ii. 352. (IK)
- ASSESSMENT: [*Lobostemon glaucophyllus* (Jacq.) Buek]
- STATUS: comb. nov. [or nom. nov.?]
- REFERENCE: Link
- BASIS: [S. Africa], no original material indicated in protologue.  
  Basionym [or replaced synonym?]: *Echium papillosum* Thunb. (1818) [tentatively accepted here as being earlier than *Echium papillosum* Lehm. (1818 (Sep–Oct))]Basionym: *Echium papillosum* Thunb.
- COMMENTS: This new combination (or, should Lehmann’s *Echium papillosum* prove to have priority: replacement name) first appears among a set of Corrigenda in a rather mysterious instruction: “*Echium papillosum* deleatur & pro *Cynoglossum uvineatum* legatur: *Cynoglossum papillosum*.” *C. uvineatum* is a non-existent name, and pro commands the ablative not the accusative case. The only plausible – if conjectural – explanation, which also justifies the treatment in Schultes’ 1823 re-edition of the book (Thunb., Fl. Cap., ed. [2]: 162. 1823), is that the printer misread Thunberg’s last-minute scrawl, which had *muricatum* instead of *uvineatum* (see also Juel, Pl. Thunb.: 322. 1818), and *post* instead of *pro*. The instruction, thus corrected, translates: “delete *Echium papillosum* and [instead], after *Cynoglossum muricatum*, read: *Cynoglossum papillosum*”.

---

*Cynoglossum papuanum* Schltr. ex O. Brand in Bot. Jahrb. Syst. 62: 489. 1929

- IPNI: Cynoglossum papuanum Schltr. ex Brand -- Bot. Jahrb. Syst. 62(5): 489. 1929 [15 May 1929] (IK)
- ASSESSMENT: accepted
- STATUS: spec. nov.
- REFERENCE: Link
- BASIS: Original material: Nordöstl. Neu-Guinea: Saruwaged-Gebirge (KEYSSER no. 22 – Original der Art!) [from protologue]. Holotype perhaps B†

---

*Cynoglossum paracaryum* Greuter & Burdet in Willdenowia 11: 35. 1981

- IPNI: Cynoglossum paracaryum Greuter & Burdet -- Willdenowia 11(1): 35, nom. nov. 1981 (IK)
- ASSESSMENT: accepted
- STATUS: nom. nov.
- REFERENCE: Link
- BASIS: Replaced synonym: *Omphalodes hirsuta*  DC., non *Cynoglossum hirsutum* Thunb.
- COMMENTS: For additional information see: *Paracaryum hirsutum* (DC.) Boiss..

---

*Cynoglossum parviflorum* K. Krause in Bot. Jahrb. Syst. 37: 634. 1906

- IPNI: Cynoglossum parviflorum K.Krause -- Bot. Jahrb. Syst. 37(5): 634. 1906 [30 Oct 1906] (IK)
- ASSESSMENT: *Hackelia parviflora* (K. Krause) Brand
- STATUS: spec. nov.
- REFERENCE: Link
- SYNONYMY:   
  ≡ *Hackelia parviflora* (K. Krause) Brand 1931
- BASIS: Original material: Peru: Inter Tarma et La Oroya (Dep. Junin), in saxosis calcareis, 3700–4000 m s.m. (WEBERBAUER n. 2520) [from protologue]

---

*Cynoglossum parvifolium* K. Koch in Linnaea 22: 645. 1849

- IPNI: Cynoglossum parvifolium K.Koch -- Linnaea 22: 645. 1849 (IK)
- ASSESSMENT: [*Cynoglossum montanum* L.]
- STATUS: spec. nov.
- REFERENCE: Link
- SYNONYMY:   
  ≡ *Cynoglossum montanum* var. *parvifolium* (K. Koch) Brand 1921
- BASIS: Original material: Im pontischen Hochgebirge, im Gaue Pertakrek auf Urgestein, c. 5500' hoch [*Koch*] [from protologue]. Holotype probably B†

---

*Cynoglossum parvifolium* (Vis.) Degen, Fl. Veleb. 2: 566. 1937

- IPNI: Cynoglossum parvifolium (Vis.) Degen -- Fl. Veleb. ii. 566 (1937), cum descr. ampl. (IK)
- ASSESSMENT: *Cynoglossum pustulatum* subsp. *parvifolium* (Vis.) Sutorý
- STATUS: nom. illeg. [homonym]
- REFERENCE: Link
- BASIS: Basionym: *Cynoglossum officinale* var. *parvifolium* Vis.

---

*Cynoglossum patulum* (Lehm.) E. H. L. Krause in Sturm, Deutschl. Fl., ed. 2, 11: 33. 1903

- IPNI: Cynoglossum patulum E.H.L.Krause -- Deutschl. Fl. (Sturm), ed. 2. 11: 33. 1903 (IK)
- ASSESSMENT: *Lappula patula* (Lehm.) Asch. ex Gürke
- STATUS: comb. nov.
- REFERENCE: Link
- BASIS: Basionym: *Echinospermum patulum*  Lehm.

---

*Cynoglossum pauciflorum* Ruiz & Pav., Fl. Peruv. 2: 6. 1799

- IPNI: Cynoglossum pauciflorum Ruiz & Pav. -- Fl. Peruv. [Ruiz & Pavon] 2: 6. 1799 (IK)
- ASSESSMENT: [*Pectocarya pusilla* (A. DC.) A. Gray]
- STATUS: spec. nov.
- REFERENCE: Link
- BASIS: Original material: in Conceptionis Chile campis et arvis [from protologue]. Original specimen: MA (MA814806\*)

---

*Cynoglossum pauciflorum* of Bertero in Mercurio Chileno 13: 610. 1829

- IPNI: Cynoglossum pauciflorum Bertero -- Merc. Chil. (Apr. 1829) 510. (IK)
- STATUS: [isonym]
- REFERENCE: Link

---

*Cynoglossum paucisetum* Borbás in Oesterr. Bot. Z. 38: 44. 1888

- IPNI: Cynoglossum paucisetum Borbás -- Oesterr. Bot. Z. 38(2): 44. 1888 [Feb 1888]
- ASSESSMENT: [*Cynoglossum officinale* L.]
- STATUS: spec. nov.
- REFERENCE: Link
- SYNONYMY:   
  ≡ *Cynoglossum punctatum* Borbás ex T. Durand & B. D. Jacks. 1902, nom. illeg.
    
  ≡ *Cynoglossum officinale* f. *paucisetum* (Borbás) Jáv. 1925
- BASIS: Original material: In silvaticis montis Büdös Transsilvaniae (Jul. 1878) [from protologue] Lectotype (Sutorý Acta Bot. Hung. 54: 2012): in apertis sylvaticis montis “Büdös” [Büdes Hegy, ca 6 km E of Bixad], ad pagum Bükkszád [Bixad], 21 Jul 1878, BP (BP 564837); isolectotypes: BP (BP564836, BP564835)

---

*Cynoglossum pellucidum* Lapeyr., Hist. Pl. Pyrenées, Suppl.: 28. 1818

- IPNI: Cynoglossum pellucidum Lapeyr. -- Hist. Pl. Pyrenées Suppl. 28. (IK)
- ASSESSMENT: *Cynoglossum germanicum* subsp. *pellucidum* (Lapeyr.) Sutorý
- STATUS: spec. nov.
- REFERENCE: Link
- SYNONYMY:   
  ≡ *Cynoglossum germanicum* subsp. *pellucidum* (Lapeyr.) Sutorý 1988
- BASIS: Original material: M. Marchand l’a trouvée en 1814, sur la Hourquete d’Arreu [from protologue]. Presumed holotype: TLJ (F. Selvi, pers. comm.)

---

*Cynoglossum penicillatum* Hook. & Arn., Bot. Beechey Voy.: 371. 1839

- IPNI: Cynoglossum penicillatum Hook. & Arn. -- Bot. Beechey Voy. 371. 1839 [Jan-May 1839] (GCI)
- ASSESSMENT: *Pectocarya penicillata*  (Hook. & Arn.) A. DC.
- STATUS: spec. nov.
- REFERENCE: Link
- SYNONYMY:   
  ≡ *Pectocarya penicillata*  (Hook. & Arn.) A. DC. 1846
- BASIS: Original material: [California, *Douglas*] no further details in protologue. Original specimens: *Douglas*, E (E00369165), G-DC (G00204665), M (M0174209\*), NY (NY 547995\*)

---

*Cynoglossum persicum* (Boiss.) Greuter & Stier in Biodivers. Data J. [hoc loco]. 2015

- ASSESSMENT: accepted
- STATUS: comb. nov.
- BASIS: Basionym: *Omphalodes persica* Boiss.

---

*Cynoglossum petiolatum* (Hook.) A. DC. in Candolle, Prodr. 10: 149. 1846

- IPNI: Cynoglossum petiolatum A.DC. -- Prodr. [A. P. de Candolle] 10: 149. 1846 [8 Apr 1846] (IK)
- ASSESSMENT: [*Cynoglossum microglochin* Royle ex Benth.]
- STATUS: comb. nov.
- REFERENCE: Link
- BASIS: Basionym: *Anchusa petiolata* Hook.

---

*Cynoglossum petiolatum* var. *nervosum* (Benth. ex C. B. Clarke) Bennet in J. Econ. Taxon. Bot. 4: 592. 1983

- IPNI: Cynoglossum petiolatum A.DC. var. nervosum (Benth. ex Cl.) Bennet -- J. Econ. Taxon. Bot. 4(2): 592 (1983):. (IK)
- ASSESSMENT: [*Cynoglossum microglochin* Royle ex Benth.]
- STATUS: comb. nov.
- REFERENCE: Link
- BASIS: Basionym: *Cynoglossum nervosum* Benth. ex C. B. Clarke
- COMMENTS: At varietal rank, priority of the epithet *nervosum* dates already from 1971, when publication of the (inappropriate but legitimate) combination *Cynoglossum nervosum* var. *petiolatum* (Hook.) Kazmi established the corresponding autonym. Under ICN Art. 11.6, priority of the two epithets in question is reversed at varietal rank, so that *Cynoglossum microglochin* var. *nervosum* (Benth. ex C. B. Clarke) Y. J. Nasir (1989) is correct even for a variety that includes the types of both names.

---

*Cynoglossum phardycalyx* Greuter & Stier in Biodivers. Data J. [hoc loco]. 2015

- ASSESSMENT: accepted
- STATUS: nom. nov.
- BASIS: Replaced synonym: *Paracaryum platycalyx*  Riedl, non *Cynoglossum platycalyx* (Riedl) Greuter & Stier

---

*Cynoglossum pictum* Aiton, Hortus Kew. 1: 179. 1789

- IPNI: Cynoglossum pictum [Soland.] -- Hort. Kew. [W. Aiton] 1: 179. 1789 (IK)
- ASSESSMENT: [*Cynoglossum creticum* Mill.]
- STATUS: spec. nov.
- REFERENCE: Link
- SYNONYMY:   
  ≡ *Cynoglossum creticum* var. *pictum* (Aiton) Maire 1934
- BASIS: Original material: Nat. of Madeira. Mr. Francis Masson. Introd. 1777 [from protologue]

---

*Cynoglossum pictum* var. *corsicum* of E. Reverchon [Baenitz, sched. impr. Herb. Eur. a 1885]

- IPNI: absent (2014-11-05)
- STATUS: nom. inval. [nom. nud.]
- REFERENCE: Link
- COMMENTS: Subsequently validly published in the combination *Cynoglossum officinale* var. *corsicum* E. Rev. ex Brand (1921).

---

*Cynoglossum pictum* var. *loreyi* (Jord. ex Lange) Nyman, Consp. Fl. Eur. 3: 521. 1881

- IPNI: absent (2014-11-05)
- ASSESSMENT: [*Cynoglossum dioscoridis* Vill.]
- STATUS: comb. & stat. nov. [species to variety]
- REFERENCE: Link
- BASIS: Basionym: *Cynoglossum loreyi* Jord. ex Lange

---

*Cynoglossum pictum* var. *siculum* (Guss.) Nyman, Consp. Fl. Eur. 3: 521. 1881

- IPNI: absent (2014-11-05)
- ASSESSMENT: [*Cynoglossum creticum* Mill.]
- STATUS: comb. & stat. nov. [species to variety]
- REFERENCE: Link
- BASIS: Basionym: *Cynoglossum siculum* Guss.

---

*Cynoglossum pictum* var. *umbrosum* Rouy, Excurs. Bot. Espagne 1883: 17, 45. 1884

- IPNI: absent (2014-11-05)
- ASSESSMENT: [*Cynoglossum creticum* Mill.]
- STATUS: var. nov.
- REFERENCE: Link
- BASIS: Original material: [Spain, Madrid], dans le parc royal de la Casa de Campo
- COMMENTS: The cited publication is, at least in part, a separately paged preprint, not a reprint as TL-2 suggests. Page 17, with the locality data, also appears in Bull. Soc. Bot. France 31: 73. 1884; but page 45, with the validating description, only in 1888 (l.c. 35: 122).

---

*Cynoglossum pilosum* Ruiz & Pav., Fl. Peruv. 2: 6. 1799

- IPNI: Cynoglossum pilosum Ruiz & Pav. -- Fl. Peruv. [Ruiz & Pavon] 2: 6. 1799 (IK)
- ASSESSMENT: [*Pectocarya lateriflora*  (Lam.) A. DC.]
- STATUS: spec. nov.
- REFERENCE: Link
- SYNONYMY:   
  ≡ *Rindera pilosa* (Ruiz & Pav.) Roem. &j Schult. 1819
    
  ≡ *Mattia pilosa* (Ruiz & Pav.) G. Don 1837-1838
- BASIS: Original material: ICON. CXI. Fig. b; [Peru], in Cercado et Chancay Provinciis locis aridis versùs Limae et Arnedo tractus [from protologue]. Original illustration: MA archives)

---

*Cynoglossum pilosum* of Nuttall, Gen. N. Amer. Pl. 1: 114. 1818

- IPNI: Cynoglossum pilosum Nutt. -- Gen. N. Amer. Pl. [Nuttall]. 1: 114. 1818 [14 Jul 1818] : misapplied (GCI)
- STATUS: [isonym]
- REFERENCE: Link
- COMMENTS: Nuttall’s description under *Cynoglossum pilosum?* refers to “a mere variety of the Peruvian plant” [i.e., *C. pilosum* Ruiz & Pav.].

---

*Cynoglossum pindicum* (Aldén) Greuter & Burdet in Willdenowia 11: 35. 1981

- IPNI: Cynoglossum pindicum (Aldén) Greuter & Burdet -- Willdenowia 11(1): 35. 1981 (IK)
- ASSESSMENT: accepted
- STATUS: comb. nov.
- REFERENCE: Link
- BASIS: Basionym: *Solenanthus pindicus* Aldén

---

*Cynoglossum pitardianum* Greuter & Burdet in Willdenowia 11: 36. 1981

- IPNI: Cynoglossum pitardianum Greuter & Burdet -- Willdenowia 11(1): 36, nom. nov. 1981 (IK)
- ASSESSMENT: accepted
- STATUS: nom. nov.
- REFERENCE: Link
- BASIS: Replaced synonym: *Solenanthus atlanticus* Pit., non *Cynoglossum atlanticum* Murb.

---

*Cynoglossum plantaginifolium* (Lipsky) Greuter & Stier in Biodivers. Data J. [hoc loco]. 2015

- ASSESSMENT: accepted
- STATUS: comb. nov.
- BASIS: Basionym: *Solenanthus plantaginifolius* Lipsky

---

*Cynoglossum platycalyx* (Riedl) Greuter & Stier in Biodivers. Data J. [hoc loco]. 2015

- ASSESSMENT: accepted
- STATUS: comb. nov.
- BASIS: Basionym: *Lindelofia platycalyx* Riedl

---

*Cynoglossum platyphyllum* Klotzsch in Peters, Naturw. Reise Mossambique 6: 254. 1861

- IPNI: Cynoglossum platyphyllum Klotzsch -- Naturw. Reise Mossambique [Peters] 6(Bot., 1): 254. 1861 (IK)
- ASSESSMENT: Not *Cynoglossum* s. l.; genus uncertain [*“Cynoglossum” lanceolatum* Forssk.]
- STATUS: spec. nov.
- REFERENCE: Link
- BASIS: Original material: [Mozambique], auf der Insel Aujoana [from protologue]

---

*Cynoglossum polyanthum* (Rech. f. & Riedl) Greuter & Stier in Biodivers. Data J. [hoc loco]. 2015

- ASSESSMENT: accepted
- STATUS: comb. nov.
- BASIS: Basionym: *Mattiastrum polyanthum* Rech. f. & Riedl

---

*Cynoglossum polycarpum* (Rech. f.) Greuter & Burdet in Willdenowia 11: 36. 1981

- IPNI: Cynoglossum polycarpum (Rech.f.) Greuter & Burdet -- Willdenowia 11(1): 36. 1981 (IK)
- ASSESSMENT: accepted
- STATUS: comb. nov.
- REFERENCE: Link
- BASIS: Basionym: *Mattiastrum polycarpum* Rech. f.

---

*Cynoglossum pringlei* Greenm. in Proc. Amer. Acad. Arts 40: 30. 1904

- IPNI: Cynoglossum pringlei Greenm. -- Proc. Amer. Acad. Arts 40: 30. 1904 (GCI)
- ASSESSMENT: *Oncaglossum pringlei*  (Greenm.) Sutorý
- STATUS: spec. nov.
- REFERENCE: Link
- SYNONYMY:   
  ≡ *Oncaglossum pringlei*  (Greenm.) Sutorý 2010
- BASIS: Original material: MEXICO. State of Mexico: Fultenango Cañon, altitude 2500 m., 9 October, 1902, *C. G. Pringle*, no. 11,350 (hb. Gr.): “Morelia (Michuacán) et de Moro Leon (Guanajuato),” *A. Dugès* (hb. Gr., collection of 1893) [from protologue]. Syntype: *Pringle 11350*, GH (GH00311020\*)

---

*Cynoglossum prostratum* Buch.-Ham. ex D. Don, Prodr. Fl. Nepal.: 100. 1825

- IPNI: Cynoglossum prostratum Buch.-Ham. ex D.Don -- Prodr. Fl. Nepal. 100. 1825 [26 Jan-1 Feb 1825] (IK)
- ASSESSMENT: [*Bothriospermum zeylanicum* (J. Jacq.) Druce]
- STATUS: spec. nov.
- REFERENCE: Link
- BASIS: Original material: [Nepal], ad Baguanpur. *Hamilton* [from protologue]

---

*Cynoglossum punctatum* Borbás ex T. Durand & B. D. Jacks., Index Kew., Suppl. 1: 121. 1902

- IPNI: Cynoglossum punctatum Borbás ex T.Durand & B.D.Jacks. -- Index Kew. Suppl. 1: 121. 1902 [Jan-Feb 1902] (IK)
- ASSESSMENT: [*Cynoglossum officinale* L.]
- STATUS: nom. illeg. [superfl.]
- REFERENCE: Link
- BASIS: Replaced synonym: *Cynoglossum paucisetum* Borbás
- COMMENTS: A (non-correctable) error, later rectified in the “Addenda et Emendanda” (l.c.: 498. 1906).

---

*Cynoglossum pustulatum* Boiss., Elench. Pl. Nov.: 66. 1838

- IPNI: Cynoglossum pustulatum Boiss. -- Elench. Pl. Nov. 66. 1838 [Jun 1838] (IK)
- ASSESSMENT: accepted
- STATUS: spec. nov.
- REFERENCE: Link
- SYNONYMY:   
  ≡ *Cynoglossum nebrodense* var. *pustulatum* (Boiss.) Boiss. 1841
    
  ≡ *Cynoglossum nebrodense* subsp. *pustulatum* (Boiss.) O. Bolòs & Vigo 1995
- BASIS: Original material: in umbrosis et pinguibus montium Granatensium, Sierra Tejeda, Sierra Nevada alt. 4000'–6000' [from protologue]. Lectotype (Burdet & al. in Candollea 38: 406. 1983): G (G00177120\*); isolectotypes: G (G00177121\*, G00177122,\* G00177123\*)

---

*Cynoglossum pustulatum* subsp. *parvifolium* (Vis.) Sutorý in Čas. Morav. Mus. Brne, Vědy Přír. 74: 170. 1989

- IPNI: Cynoglossum pustulatum Boiss. subsp. parvifolium (Vis.) Sutorý -- Čas. Morav. Muz., Vědy Přir. 74(1–2): 170 (1989):. (IK)
- ASSESSMENT: accepted
- STATUS: comb. & stat. nov. [variety to subspecies]
- REFERENCE: Link
- BASIS: Basionym: *Cynoglossum officinale* var. *parvifolium* Vis.

---

*Cynoglossum pustulatum* subsp. *soilae* P. Monts. & Alejandre in Bull. Soc. Hist. Nat. Toulouse 141(2): 32. 2006

- IPNI: Cynoglossum pustulatum Boiss. subsp. soilae P.Monts. & Alejandre -- Bull. Soc. Hist. Nat. Toulouse 141(2): 32. 2006 [2005 publ. 15 Nov 2006]
- ASSESSMENT: accepted
- STATUS: subsp. nov.
- REFERENCE: Link
- BASIS: Original material: Campezo (Alava), Antoñana. in monte Soila. latere orientem versus, in glareosis ad pedem rupes culminis. cum Buxus. Quercus. Fagus rarioque, ad 885m altitudine, UTM WN478269. Leg. J.A. Alejandre & M.J. Escalante, die 10-VI-2004: holotypus JACA 277563R, Isotypo in herb. Alejandre 506/04 [from protologue]

---

*Cynoglossum pygmaeum* (Rech. f.) Greuter & Stier in Biodivers. Data J. [hoc loco]. 2015

- ASSESSMENT: accepted
- STATUS: comb. nov.
- BASIS: Basionym: *Mattiastrum pygmaeum* Rech. f.

---

*Cynoglossum racemosum* Schreb. in Nova Acta Phys.-Med. Acad. Caes. Leop.-Carol. Nat. Cur. 3: 475. 1767

- IPNI: Cynoglossum racemosum Schreb. -- Nova Acta Phys.-Med. Acad. Caes. Leop.-Carol. Nat. Cur. 3: 475. 1767 (IK)
- ASSESSMENT: accepted
- STATUS: spec. nov.
- REFERENCE: Link
- SYNONYMY:   
  ≡ *Paracaryum racemosum* (Schreb.) Britten 1906
    
  ≡ *Mattiastrum racemosum* (Schreb.) Brand 1915
- BASIS: Original material: *Cynoglossum orientale minus, flore campanulato caeruleo*. Tourn. cor. 7. in Oriente [from protologue]. Presumed holotype: *Tournefort* or *Gundelsheimer*, M; likely isotypes (Mill in Davis, Fl. Turkey 6: 286. 1978, as “type”): herb. Tournefort 657, P-TRF; *Cynoglossum angustifolium*, “Roestel”, B-W (BW03337010\*)
- COMMENTS: Tournefort’s phrase name from the Corollarium (p. 7, 1707) is cited in the protologue of no less than three *Cynoglossum* names: *Cynoglossum racemosum* Schreb. (1767), *Cynoglossum emarginatum* Lam. (1791) and *Cynoglossum angustifolium* Willd. (1789). The three specimens used by the authors of these names were likely duplicates of a single gathering made by Tournefort and his companion Gundelsheimer during their travel through the Orient. However, as none of these names were typified at that time, and the later authors did not cite any earlier name in synonymy, the three names are heterotypic and legitimate.

---

*Cynoglossum racemosum* of Roxburgh, Hort. Bengal.: 13. 1814

- IPNI: Cynoglossum racemosum Roxb. -- Hort. Bengal. 13 (1814), nomen; Fl. Ind., ed. Carey & Wall., ii. 6 (1824); Fl.Ind., ed. Carey, i. 456 (1832). (IK)
- STATUS: nom. inval. [nom. nud.]
- REFERENCE: Link

---

*Cynoglossum racemosum* Roxb., Fl. Ind. 2: 6. 1824

- IPNI: Cynoglossum racemosum Roxb. -- Hort. Bengal. 13 (1814), nomen; Fl. Ind., ed. Carey & Wall., ii. 6 (1824); Fl.Ind., ed. Carey, i. 456 (1832). (IK)
- ASSESSMENT: Not *Cynoglossum* s. l.; genus uncertain [*“Cynoglossum” lanceolatum* Forssk.]
- STATUS: nom. illeg. [homonym]
- REFERENCE: Link
- BASIS: Original material: on rubbish in the vicinity of Calcutta [from protologue]

---

*Cynoglossum ramosissimum* DC., Prodr. 10: 152. 1846

- IPNI: absent (2014-11-05)
- ASSESSMENT: [*Cynoglossum hirsutissimum* Lehm.]
- STATUS: spec. nov.
- REFERENCE: Link
- BASIS: Original material: No indication in protologue. If, as conjectured below, this is the result of a slip of the pen (or of memory) of one of the Candolles, the type would be the same as for *Cynoglossum hirsutissimum* Lehm.
- COMMENTS: A mysterious name, not even listed in Buek’s *Index*. It appears in a note under *Cynoglossum leptostachyum* DC., a new species, as follows: “An [*C. leptostachyum*] var. *C. ramosissimi* sed pubescens nec villoso-hirsutum …” [perhaps a variety of *C. r.* but pubescent rather than villous-hirsute …]; which, as the words “villoso-hirsutum” diagnose *C. ramosissimum* against *C. leptostachyum*, suffices to validate the former name. From the context, it is likely that *C. ramosissimum* is an error for *Cynoglossum hirsutissimum* Lehm., the next following entry, but as this cannot be proven, *C. ramosissimum* must be considered a legitimate name.

---

*Cynoglossum rechingeri* Greuter & Stier in Biodivers. Data J. [hoc loco]. 2015

- ASSESSMENT: accepted
- STATUS: nom. nov.
- BASIS: Replaced synonym: *Mattiastrum formosum* Rech. f. & Riedl, non *Cynoglossum formosum* (R. R. Mill) Greuter & Burdet

---

*Cynoglossum regelii* Greuter & Stier in Biodivers. Data J. [hoc loco]. 2015

- ASSESSMENT: accepted
- STATUS: nom. nov.
- BASIS: Replaced synonym: *Solenanthus hirsutus* Regel, non *Cynoglossum hirsutum* Thunb.

---

*Cynoglossum regium* (S. G. Gmel.) Greuter & Stier in Biodivers. Data J. [hoc loco]. 2015

- ASSESSMENT: accepted
- STATUS: comb. nov.
- BASIS: Basionym: *Symphytum regium*  S. G. Gmel.

---

*Cynoglossum remotum* Moench, Methodus: 419. 1794

- IPNI: Cynoglossum remotum Moench -- Methodus (Moench) 419. 1794 [4 May 1794] (IK)
- ASSESSMENT: *Cynoglossum apenninum* L.
- STATUS: nom. illeg. [superfl.]
- REFERENCE: Link
- BASIS: Replaced synonym: *Cynoglossum apenninum* L.

---

*Cynoglossum reuteri* (Boiss. & Hausskn.) Greuter & Burdet in Willdenowia 11: 36. 1981

- IPNI: Cynoglossum reuteri (Boiss. & Hausskn.) Greuter & Burdet -- Willdenowia 11(1): 36. 1981 (IK)
- ASSESSMENT: accepted
- STATUS: comb. nov.
- REFERENCE: Link
- BASIS: Basionym: *Paracaryum reuteri* Boiss. & Hausskn.

---

*Cynoglossum reverchonii* of Debeaux [Degen in Magyar Bot. Lapok 2: 311. 1903] *(‘reverchoni’)*

- IPNI: Cynoglossum reverchonii Debeaux ex Degen -- Magyar Bot. Lapok 1903, ii. 311, in syn. (IK)
- STATUS: nom. inval. [pro syn. sub *Solenanthus reverchonii*]
- REFERENCE: Link
- COMMENTS: The name also appears, without description, on some printed labels of E. Reverchon’s Pl. Espagne No. 1190, collected in Sierra de Castril in 1903 (GH). It was validated as *Solenanthus reverchonii*  Degen.

---

*Cynoglossum reverchonii* (Degen) Greuter & Burdet in Willdenowia 11: 36. 1981

- IPNI: Cynoglossum reverchonii (Debeaux ex Degen) Greuter & Burdet -- Willdenowia 11(1): 36. 1981 (IK)
- ASSESSMENT: accepted
- STATUS: comb. nov.
- REFERENCE: Link
- BASIS: Basionym: *Solenanthus reverchonii*  Degen

---

*Cynoglossum revolutum* Ruiz & Pav., Fl. Peruv. 2: 6. 1799

- IPNI: Cynoglossum revolutum Ruiz & Pav. -- Fl. Peruv. [Ruiz & Pavon] 2: 6. 1799 (IK)
- ASSESSMENT: *Hackelia revoluta*  (Ruiz & Pav.) I. M. Johnst.
- STATUS: spec. nov.
- REFERENCE: Link
- SYNONYMY:   
  ≡ *Hackelia revoluta*  (Ruiz & Pav.) I. M. Johnst. 1923
- BASIS: Original material: passim in Huassahuassi umbrosis [from protologue]. Original specimen: MA (MA814809\*)

---

*Cynoglossum rindera* of Linnaeus f., Suppl. Pl.: 130. 1782

- IPNI: Cynoglossum rindera L.f. -- Suppl. Pl. 130. 1782 [1781 publ. Apr 1782] (IK)
- STATUS: nom. inval. [ICN Art. 36.1(a)]
- REFERENCE: Link
- COMMENTS: Corrected to *Cynoglossum laevigatum* L. in the errata on page 468.

---

*Cynoglossum rindera* Pallas, Fl. Ross. 1(2): 96.1789

- IPNI: absent (2014-11-05)
- ASSESSMENT: *Cynoglossum tetraspis* (Pall.) Greuter & Burdet
- STATUS: nom. illeg. [superfl.]
- REFERENCE: Link
- BASIS: Replaced synonym: *Rindera tetraspis* Pall.

---

*Cynoglossum ritchiei* C. B. Clarke in Hooker, Fl. Brit. India 4: 157. 1883

- IPNI: Cynoglossum ritchiei C.B.Clarke -- Fl. Brit. India [J. D. Hooker] 4(10): 157. 1883 [Jun 1883] (IK)
- ASSESSMENT: accepted
- STATUS: spec. nov.
- REFERENCE: Link
- BASIS: Original material: Bombay; Belgaum, *Ritchie* [from protologue]

---

*Cynoglossum robustum* Hassk. in Acta Soc. Regiae Sci. Indo-Neerl. 1(8): 44. 1856

- IPNI: Cynoglossum robustum Hassk. -- in Ver. Nat. Ver. Nederl. Ind. i. (1856) 44. (IK)
- ASSESSMENT: *Cynoglossum* sp. (species dubia)
- STATUS: spec. nov.
- REFERENCE: Link
- BASIS: Original material: in horto Tjipannas [sic!] culta, origine mihi ignota [from protologue]. In a subsequent publication [extract from a letter of Hasskarl dated 6. April 1856, in Verslagen Meded. Afd. Natuurk. Kon. Akad. Wetensch. 5: 94. 1857] this reads, more sensibly: “Teysmann, hortulanus, reperit in Javae orientalis montibus altioribus”
- COMMENTS: From the description, this might well be *Cynoglossum amabile* Stapf & J. R. Drumm., which for priority reasons it would then threaten to displace.

---

*Cynoglossum rochelia* A. DC. in Candolle, Prodr. 10: 152. 1846

- IPNI: Cynoglossum rochelia A.DC. -- Prodr. [A. P. de Candolle] 10: 152. 1846 [8 Apr 1846] (IK)
- ASSESSMENT: accepted
- STATUS: spec. nov.
- REFERENCE: Link
- BASIS: Original material: in montibus et campis editioribus Mauritii (Bojer! Bouton!) et Borbonis (Bouton!) nec non ad fretum Bass Novae-Hollandiae [from protologue]. Syntypes: Mauritius, *Bojer*, G-DC (G00205654\*); id., *Bouton*, G-DC (G00205651\*); Réunion, *Bouton*, G-DC (G00205653\*); Détroit de Bass, *Urville*, G-DC (G00205671\*)

---

*Cynoglossum rotatum* Velen. in Sitzungsber. Königl. Böhm. Ges. Wiss., Math.-Naturwiss. Cl. 1893(37): 48. 1893

- IPNI: Cynoglossum rotatum Velen. -- Sitzungsber. Königl. Böhm. Ges. Wiss. (1892) 3. (IK)
- ASSESSMENT: [Cynoglossum montanum L.]
- STATUS: spec. nov.
- REFERENCE: Link
- BASIS: Original material: [Bulgaria], in calcareis supra Belledihan legi [*Velenovský*] a. 1893 [from protologue]
- COMMENTS: See Sutory in Phytol. Balcan. 14: 255—256. 2008.

---

*Cynoglossum rotundum* (Sutorý) Landolt, Fl. Indicativa: 268. 2010

- IPNI: Cynoglossum rotundum (Sutorý) Landolt -- Fl. Indicativa 268. 2010
- ASSESSMENT: *Cynoglossum germanicum* subsp. *rotundum* Sutorý
- STATUS: comb. & stat. nov. [subspecies to species]
- REFERENCE: Link
- BASIS: Basionym: *Cynoglossum germanicum* subsp. *rotundum* Sutorý

---

*Cynoglossum roylei* of Wallich, Numer. List: 26. 1829

- IPNI: Cynoglossum roylei Wall. -- Numer. List [Wallich] n. 917. 1829 (IK)
- STATUS: nom. inval. [nom. nud.]
- REFERENCE: Link

---

*Cynoglossum roylei* Wall. ex G. Don, Gen. Hist. 4: 356. 1837–1838

- IPNI: Cynoglossum roylei Wall. & G.Don -- Gen. Hist. iv. 356 (1837), descr. (IK)
- ASSESSMENT: [*Hackelia uncinata* (Royle ex Benth.) C. E. C. Fischer]
- STATUS: spec. nov.
- REFERENCE: Link
- BASIS: Original material: Wall. cat. no. 917; H. [in hothouse]; on Choor Mount [from protologue]

---

*Cynoglossum ruderale* Salisb., Prodr. Stirp. Chap. Allerton: 116. 1796

- IPNI: Cynoglossum ruderale Salisb. -- Prodr. Stirp. Chap. Allerton 116. 1796 [Nov-Dec 1796] (IK)
- ASSESSMENT: *Cynoglossum officinale* L.
- STATUS: nom. illeg. [superfl.]
- REFERENCE: Link
- BASIS: Replaced synonym: *Cynoglossum officinale* L.

---

*Cynoglossum rugosum* Sestini ex Roem. & Schult., Syst. Veg. 4: 764. 1819

- IPNI: Cynoglossum rugosum Sestini ex Roem. & Schult. -- Syst. Veg., ed. 15 bis [Roemer & Schultes] 4: 764. 1819 (IK)
- ASSESSMENT: [*Moltkia coerulea*  (Willd.) Lehm.]
- STATUS: spec. nov.
- REFERENCE: Link
- BASIS: Original material: Herb. Willd. MSS. In Galatia. Sestini [from protologue]; Holotype: B-W (B-W03336010\*); Isotype: HAL (HAL0108237\*)

---

*Cynoglossum rugulosum* (DC.) Greuter & Burdet in Willdenowia 11: 36. 1981

- IPNI: Cynoglossum rugulosum (DC.) Greuter & Burdet -- Willdenowia 11(1): 36. 1981 (IK)
- ASSESSMENT: accepted
- STATUS: comb. nov.
- REFERENCE: Link
- BASIS: Basionym: *Omphalodes rugulosa*  DC.

---

*Cynoglossum rupestre* of Hans [Brand in Engler, Pflanzenr. 78 (IV.252): 134. 1921]

- IPNI: Cynoglossum rupestre Hans ex Brand -- Pflanzenr. (Engler) Borrag.-Cynogloss. 134 (1921), in obs. (IK)
- STATUS: nom. inval. [not accepted, ICN Art. 36.1(a)]
- REFERENCE: Link

---

*Cynoglossum sabirense* (R. R. Mill & A. G. Mill.) J. R. I. Wood, Handb. Yemen Fl.: 241. 1997

- IPNI: Cynoglossum sabirense (R.R.Mill & A.G.Mill.) J.R.I.Wood -- Handbook Yemen Fl. 241 (1997):. (IK)
- ASSESSMENT: Not *Cynoglossum* s. l.; genus uncertain (species accepted)
- STATUS: comb. nov.
- REFERENCE: Link
- BASIS: Basionym: *Paracynoglossum sabirense* R. R. Mill & A. G. Mill.

---

*Cynoglossum salesianorum* of Sennen in Butl. Inst. Catalana Hist. Nat. 18: 176. 1918, pro hybr.

- IPNI: Cynoglossum salesianorum Sennen -- Butl. Inst. Catalana Hist. Nat. 1918, xviii. 176, nomen, hybr. (IK)
- STATUS: nom. inval. [nom. nud.]
- REFERENCE: Link

---

*Cynoglossum salesianorum* Sennen in Bol. Soc. Ibér. Ci. Nat. 29: 75. 1930, pro hybr.

- IPNI: Cynoglossum salesianorum Sennen & Sennen -- Bol. Soc. Iber. 1930, xxix. 75, descr. (IK)
- ASSESSMENT: accepted as hybrid
- STATUS: hybr. nov. [*C. cheirifolium* × *C. creticum*)
- REFERENCE: Link
- BASIS: Original material: [Sennen, Pl. Espagne] No. 3116. Barcelone, coteaux de Vallcarca, sur le calcaire, inter parentes [from protologue]. Syntype: MPU (MPU019684\*)
- COMMENTS: According to Aizpuru (in Castroviejo, Fl. Iber. 11: 463. 2012) the name already appears, as nom. nud., in Butl. Inst. Catalana Hist. Nat. 18: 176. 1918.

---

*Cynoglossum scabrum* Opiz in Berchtold & al., Oekon.-Techn. Fl. Böhm. 2(2): 160. 1839

- IPNI: absent (2014-11-05)
- ASSESSMENT: [*Cynoglossum germanicum* Jacq.]
- STATUS: spec. nov.
- REFERENCE: Link
- BASIS: Original material: in Böheim [Bohemia] (nach Römer und Schultes S. Mert. und Koch Deut. Flora 2. p. 68.) [from protologue]
- COMMENTS: Appears as an unnumbered entry preceded by the sign +, after the numbered entry *Cynoglos[s]um montanum*; but, as shown by other, parallel examples in the same work, it is nevertheless considered a species, not an infraspecific taxon subordinate to the latter. The inclusion, in synonymy, of the names *C. sylvaticum* (ascribed to Besser) and *C. haenkei* Schult. does not make *C. scabrum* illegitimate, because the type of *C. sylvaticum* Haenke is at the same time excluded by being cited under the preceding species, *C. montanum*.

---

*Cynoglossum scardicum* (Bornm.) Greuter & Burdet in Willdenowia 11: 36. 1981

- IPNI: Cynoglossum scardicum (Bornm.) Greuter & Burdet -- Willdenowia 11(1): 36. 1981 (IK)
- ASSESSMENT: accepted
- STATUS: comb. nov.
- REFERENCE: Link
- BASIS: Basionym: *Solenanthus scardicus* Bornm.

---

*Cynoglossum schlagintweitii* of Kazmi in J. Arnold Arbor. 52: 352. 1971 [Apr]

- IPNI: Cynoglossum schlagintweitii (Brand) Kazmi -- J. Arnold Arbor. 52(2): 352 (Apr. 1971). (IK)
- STATUS: [isonym]
- REFERENCE: Link

---

*Cynoglossum schlagintweitii* (Brand) Riedl in Oesterr. Bot. Z. 119: 70. 1971 [Febr]

- IPNI: Cynoglossum schlagintweitii (Brand) Riedl -- Oesterr. Bot. Z. 119(1-3): 70. 1971 (IK)
- ASSESSMENT: accepted
- STATUS: comb. nov.
- REFERENCE: Link
- BASIS: Basionym: *Adelocaryum schlagintweitii* Brand

---

*Cynoglossum schlumbergeri* (Boiss.) Greuter & Burdet in Willdenowia 11: 36. 1981

- IPNI: Cynoglossum schlumbergeri (Boiss.) Greuter & Burdet -- Willdenowia 11(1): 36. 1981 (IK)
- ASSESSMENT: accepted
- STATUS: comb. nov.
- REFERENCE: Link
- BASIS: Basionym: *Mattia schlumbergeri* Boiss.

---

*Cynoglossum scorpioides* Haenke in Jacquin, Collectanea 2: 3. 1787

- IPNI: Cynoglossum scorpioides Haenke -- in Jacq. Coll. ii. 3. (IK)
- ASSESSMENT: *Memoremea scorpioides* (Haenke) Otero & al.
- STATUS: spec. nov.
- REFERENCE: Link
- SYNONYMY:   
  ≡ *Memoremea scorpioides* (Haenke) Otero & al. 2014
- BASIS: Original material: in horto *Baumgarten* dicto Praga Bohemorum metropoli non procul, si colles ad dextram salutaveris: frequentissima vero in nemoribus ad ripas Albis prope trajectum *Stephan Ueberfuhr*, atque ad Moldavae cum Albi unionem supra Melnick regiam urbem: nec uspiam alibi visam [from protologue]. Lectotype (Kirschner & al. in Preslia 79: 344. 2007): Cynoglossum scorpioides, Bohemia, in vicinis Pragensi, ad Carlsstein, et St. Ivan, *Haenke* in herb. Jacquin W (W0011093\*)

---

*Cynoglossum semnanicum* Khat. in Iranian J. Bot. 8: 2. 1999

- IPNI: Cynoglossum semnanicum Khat. -- Iranian J. Bot. 8(1): 2 (1999). (IK)
- ASSESSMENT: accepted
- STATUS: spec. nov.
- REFERENCE: Link
- BASIS: Original material: Semnan, ca. 50 km N. of Semnan, between Sheli and Hikuh villages, 2400 m, *Assadi and Mozaffarian 40638* (holotypus TARI) [from protologue]

---

*Cynoglossum seravschanicum* (B. Fedtsch.) Popov in Korovin & al., Descr. Pl. Nov. Turkestan.: 64. 1916

- IPNI: Cynoglossum seravschanicum Popov -- Descr. Pl. Nov. Turkestan. [Korovin, Kultiasow & Popov] 64. 1916 (IK)
- ASSESSMENT: accepted
- STATUS: comb. nov.
- REFERENCE: Link
- BASIS: Basionym: *Trachelanthus seravschanicus* B. Fedtsch.

---

*Cynoglossum sessiliflorum* of Poeppig [Candolle, Prodr. 10: 133. 1846]

- IPNI: Cynoglossum sessiliflorum Poepp. ex DC. -- Prodr. [A. P. de Candolle] 10: 133. 1846 [8 Apr 1846] (IK)
- STATUS: nom. inval. [pro syn. sub *Eritrichium sessiliflorum*]
- REFERENCE: Link

---

*Cynoglossum sessiliflorum* (Rech. f. & Riedl) Greuter & Stier in Biodivers. Data J. [hoc loco]. 2015

- ASSESSMENT: accepted
- STATUS: comb. nov.
- BASIS: Basionym: *Mattiastrum sessiliflorum*  Rech. f. & Riedl

---

*Cynoglossum sessilifolium* of Poeppig [Reiche, Fl. Chile 5: 212. 1910]

- IPNI: Cynoglossum sessilifolium Poepp. ex Reiche -- Fl. Chile [Reiche] v. 212 (1910). (IK)
- STATUS: nom. inval. [pro syn. sub *Allocarya procumbens*]
- REFERENCE: Link

---

*Cynoglossum shepardii* (Post & Beauverd) Greuter & Burdet in Willdenowia 11: 36. 1981

- IPNI: Cynoglossum shepardii (Post & Beauverd) Greuter & Burdet -- Willdenowia 11(1): 36. 1981 (IK)
- ASSESSMENT: accepted
- STATUS: comb. nov.
- REFERENCE: Link
- BASIS: Basionym: *Paracaryum shepardii* Post & Beauverd

---

*Cynoglossum siculum* Guss., Fl. Sic. Prodr., Suppl.: 52. 1832

- IPNI: Cynoglossum siculum Guss. -- Fl. Sic. Prodr. Suppl.(1): 52. 1832 (IK)
- ASSESSMENT: [*Cynoglossum creticum* Mill.]
- STATUS: spec. nov.
- REFERENCE: Link
- SYNONYMY:   
  ≡ *Cynoglossum pictum* var. *siculum* (Guss.) Nyman 1881
    
  ≡ *Cynoglossum creticum* var. *siculum* (Guss.) Brand 1921
- BASIS: Original material: In apricis cultis montosis; Palermo al Caputo (Gasparrini). Aprili, Martio [from protologue]. Lectotype (Selvi & Cecchi in Taxon 58: 622. 2009): Palermo al Caputo, *Gussone*, herb. siculum, NAP

---

*Cynoglossum sintenisii* (Hausskn. ex Bornm.) Greuter & Burdet in Willdenowia 11: 36. 1981

- IPNI: Cynoglossum sintenisii (Bornm.) Greuter & Burdet -- Willdenowia 11(1): 36. 1981 (IK)
- ASSESSMENT: accepted
- STATUS: comb. nov.
- REFERENCE: Link
- BASIS: Basionym: *Paracaryum sintenisii* Hausskn. ex Bornm.

---

*Cynoglossum spelaeum* Hilliard & B. L. Burtt in Notes Roy. Bot. Gard. Edinburgh 37: 287. 1979

- IPNI: Cynoglossum spelaeum Hilliard & B.L.Burtt -- Notes Roy. Bot. Gard. Edinburgh 37(2): 287. 1979 (IK)
- ASSESSMENT: Not *Cynoglossum* s. l.; genus uncertain (species accepted)
- STATUS: spec. nov.
- REFERENCE: Link
- BASIS: Original material: Natal. Underberg distr., cobham Forest Reserve, Polele valley, in loose sandy soil at edge of overhang, flowers white, 20 iii 1977, Hilliard & Burtt 9728 (holo, E; iso. NU) [+ numerous paratypes]. Isotypes: K (K000418899\*), NU (NU0016515-0\*), PRE (PRE0659059-0\*, PRE0724426-0\*)

---

*Cynoglossum sphacioticum* Boiss. & Heldr. in Boissier, Diagn. Pl. Orient. 11: 125. 1849

- IPNI: Cynoglossum sphacioticum Boiss. & Heldr. -- Diagn. Pl. Orient. ser. 1, 11: 125. 1849 [Mar-Apr 1849] (IK)
- ASSESSMENT: accepted
- STATUS: spec. nov.
- REFERENCE: Link
- BASIS: Original material: rarò in summo vertice *Stravropodia* montium Sphacioticorum alt. 7000’, [*Heldreich*] [from protologue]. Lectotype (Strid, Mountain F. Greece 2: 59. 1991): G-BOIS (G00150523); isolectotypes: BM, G (G00177119\*), GOET (GOET005802\*)

---

*Cynoglossum stamineum* Desf. in Ann. Mus. Natl. Hist. Nat. 10: 431. 1807

- IPNI: Cynoglossum stamineum Desf. -- Ann. Mus. Natl. Hist. Nat. x. (1807) 431. (IK)
- ASSESSMENT: accepted
- STATUS: spec. nov.
- REFERENCE: Link
- SYNONYMY:   
  ≡ *Mattia staminea* (Desf.) Roem. & Schult. 1819
    
  ≡ *Solenanthus tournefortii*  DC. 1846, nom. illeg.
    
  ≡ *Solenanthus stamineus* (Desf.) Wettst. 1885
- BASIS: Original material: Elle croît naturellement en Cappadoce, et M. de Labillardière l’a aussi observée en Syrie [+ plate 36, reproducing Aubriet’s vellum painting of *Cynoglossum orientale, vulgari simile, flore minimo, longis staminibus donatum* of Tournefort (Coroll.: 7. 1703)] [from protologue]. Lectotype (Mill in Davis, Fl. Turkey 6: 304. 1978, as “holotype”: sheet 654, P-TRF
- COMMENTS: See also *Solenanthus dubius* Fisch. & C. A. Mey..

---

*Cynoglossum stamineum* of Marschall von Bieberstein, Fl. Taur.-Caucas. 3: 127. 1819–1820

- IPNI: Cynoglossum stamineum M.Bieb. -- Fl. Taur.-Caucas. 3: 127. [Dec 1819 or early 1820] (IK)
- STATUS: [isonym]
- REFERENCE: Link
- COMMENTS: See also *Solenanthus dubius* Fisch. & C. A. Mey..

---

*Cynoglossum stellulatum* of Wallich, Numer. List: 26. 1829

- IPNI: Cynoglossum stellulatum Wall. -- Numer. List [Wallich] n. 924. 1829 (IK)
- STATUS: nom. inval. [nom. nud.]
- REFERENCE: Link

---

*Cynoglossum stenolophum* (Boiss. & Balansa) Greuter & Burdet in Willdenowia 11: 36. 1981

- IPNI: Cynoglossum stenolophum (Boiss. & Balansa) Greuter & Burdet -- Willdenowia 11(1): 36. 1981 (IK)
- ASSESSMENT: accepted
- STATUS: comb. nov.
- REFERENCE: Link
- BASIS: Basionym: *Paracaryum stenolophum* Boiss. & Balansa

---

*Cynoglossum stewartii* Kazmi in J. Arnold Arbor. 52: 350. 1971

- IPNI: Cynoglossum stewartii Kazmi -- J. Arnold Arbor. 52(2): 350. 1971 (IK)
- ASSESSMENT: accepted
- STATUS: spec. nov.
- REFERENCE: Link
- BASIS: Original material: Kashmir: Pahlgam to Lake Sorus, 10,000–11,000 ft … 8 Aug. 1945, R. R. Stewart 21584 (GH-holotype) + numerous paratypes [from protologue]. Holotype: GH00092799\*

---

*Cynoglossum strictissimum* (Brand) Greuter & Stier in Biodivers. Data J. [hoc loco]. 2015

- ASSESSMENT: accepted
- STATUS: comb. nov.
- BASIS: Basionym: *Solenanthus strictissimus*  Brand

---

*Cynoglossum strictum* (K. Koch) Greuter & Burdet in Willdenowia 11: 36. 1981

- IPNI: Cynoglossum strictum (K.Koch) Greuter & Burdet -- Willdenowia 11(1): 36. 1981 (IK)
- ASSESSMENT: accepted
- STATUS: comb. nov.
- REFERENCE: Link
- BASIS: Basionym: *Omphalodes stricta*  K. Koch

---

*Cynoglossum stylosum* Kar. & Kir. in Bull. Soc. Imp. Naturalistes Moscou 15: 409. 1842

- IPNI: Cynoglossum stylosum Kar. & Kir. -- Bull. Soc. Imp. Naturalistes Moscou xv. (1842) 409. (IK)
- ASSESSMENT: accepted
- STATUS: spec. nov.
- REFERENCE: Link
- SYNONYMY:   
  ≡ *Solenanthus stylosus* (Kar. & Kir.) Lipsky 1904
    
  ≡ *Lindelofia stylosa* (Kar. & Kir.) Brand 1921
- BASIS: Original material: In pratensibus subalpinis Alatau ad fl. Sarchan [from protologue]. Lectotype (Kazmi in J. Arnold Arbor. 52: 337. 1971, as “holotype”): *Karelin & Kirilov*, LE; isolectotypes: *Karelin & Kirilov 1745*, BR (BR0000006966898\*), M (M0174179\*), US (US00110905\*)

---

*Cynoglossum stylosum* subsp. *pterocarpum* (Rupr.) Greuter & Stier in Biodivers. Data J. [hoc loco]. 2015

- ASSESSMENT: accepted
- STATUS: comb. nov.
- BASIS: Basionym: *Solenanthus nigricans* var. *pterocarpus* Rupr.

---

*Cynoglossum suaveolens* R. Br., Prodr. Fl. Nov. Holland.: 495. 1810

- IPNI: Cynoglossum suaveolens R.Br. -- Prodromus Florae Novae Hollandiae 1810 (APNI)
- ASSESSMENT: accepted
- STATUS: spec. nov.
- REFERENCE: Link
- BASIS: Original material: (M. [S coast], J. [Port Jackson area]), [*Brown*] [from protologue]. Original specimen: *Brown 2937*, BM (BM001040568\*)

---

*Cynoglossum suavifolium* Pau, Carta Bot. 3: 6. 1906

- IPNI: absent (2014-11-05)
- ASSESSMENT: [*Cynoglossum officinale* L.]
- STATUS: spec. nov.
- REFERENCE: Link
- BASIS: Original material: Ortigosa y Nieva de Cameros. – Julio 1905 [from protologue]
- COMMENTS: Pau describes this taxon as one of four “pequeñas especies” placed under *Cynoglossum valentinum* “Lge.”, one of the four being *C. valentinum* Lag. itself. Two (including the present one) he terms “psp.” (prospecies), rather than species, but as they are coordinate with two species, and all are referred to as “small species” in the preceding paragraph, we consider them to be published as binomials not as ternary (infraspecific) combinations.

---

*Cynoglossum subalpinum* T. C. E. Fr. in Notizbl. Bot. Gart. Berlin-Dahlem 8: 415. 1923

- IPNI: Cynoglossum subalpinum T.C.E.Fr. -- Notizbl. Bot. Gart. Berlin-Dahlem 8: 415. 1923 (IK)
- ASSESSMENT: Not *Cynoglossum* s. l.; genus uncertain [*“Cynoglossum” amplifolium* Hochst. ex A. DC.]
- STATUS: spec. nov.
- REFERENCE: Link
- SYNONYMY:   
  ≡ *Cynoglossum amplifolium* var. *subalpinum* (T. C. E. Fr.) Verdc. 1991
- BASIS: Original material: W.-Kenia: in der Hagenia-Hypericum-Region ca. 2900–3000 m ü.d.M. sehr häufig: Blühend und mit Frucht am 31. Januar 1922, ROB. E. et TH. C. E. FRIES n. 1331. –- Mt. Aberdare: ca. 2900 m ü. d. M. in feuchtem Gebüsch. Mit Frucht am 15. März 1922, ROB. E. et TH. C. E. FRIES n. 2423. --- Im Hagenia-Hypericum-Wald am Fuße des Sattima sehr häufig. Blühend und mit Frucht am 1. und 2. April 1922, ROB. E. et TH. C. E. FRIES n. 2684 und 2776. --- Kilmandjaro VOLKENS n. 2022 [from protologue]. Lectotype (Verdcourt in Polhill, Fl. Trop. E. Africa. Boragin.: 106. 1991, as “holotype”): *Fries & Fries 1331*, S; isolectotypes: BR (BR0000008865793), K (K000418926\*), S; paralectotype: *Fries & Fries 2776*, K (K000418925\*)

---

*Cynoglossum subglabrum* of (Mérat) [Domin in Sitzungsber. Königl. Böhm. Ges. Wiss., Math.-Naturwiss. Cl. 1902(58): 34. 1903]

- IPNI: absent (2014-11-05)
- STATUS: nom. inval. [pro syn. sub *Cynoglossum officinale*]
- REFERENCE: Link

---

*Cynoglossum subscaposum* (Rech. f. & Riedl) Greuter & Stier in Biodivers. Data J. [hoc loco]. 2015

- ASSESSMENT: accepted
- STATUS: comb. nov.
- BASIS: Basionym: *Mattiastrum subscaposum* Rech. f. & Riedl

---

*Cynoglossum sylvaticum* Haenke in Jacquin, Collectanea 2: 77. 1787

- IPNI: Cynoglossum sylvaticum Haenke -- in Jacq. Coll. ii. 77. (IK)
- ASSESSMENT: [*Cynoglossum germanicum* Jacq.]
- STATUS: spec. nov.
- REFERENCE: Link
- SYNONYMY:   
  ≡ *Cynoglossum officinale* var. *sylvaticum* (Haenke) Willd. 1798
    
  ≡ *Cynoglossum haenkei* Schult. 1814, nom. illeg.
- BASIS: Original material: Cynoglossa montana media rubro flore. *Column. ecph. pag.* 175. *tab.* 174. Nascitur in Austriae inferioris, sylvis … [from protologue]. Possible original specimen: M (M0174201\*); original illustration: Colonna, Ekphrasis: 175. 1616
- COMMENTS: The illustration in Colonna has become the lectotype of *Cynoglossum montanum* L..

---

*Cynoglossum sylvaticum* of Smith, Fl. Brit.: 216. 1800

- IPNI: absent (2014-11-05)
- STATUS: [isonym]
- REFERENCE: Link

---

*Cynoglossum teheranicum* Riedl in Oesterr. Bot. Z. 110: 512. 1963

- IPNI: Cynoglossum teheranicum Riedl -- Oesterr. Bot. Z. 110: 512. 1963 (IK)
- ASSESSMENT: [*Cynoglossum montanum* L.]
- STATUS: spec. nov.
- REFERENCE: Link
- BASIS: Original material: Persien; Zentral-Elburs: am Südabhang des Totschal im Tal Häfthous NW Teheran; Sand- u. Kiesboden des Bachbettes, 1300--1500 m, 4. VII. 1948. P. AELLEN 1426 (Holotypus, W) + one paratype 1440 (W) [from protologue]. Holotype: W 1961-0001745A; paratype: W 1961-0001745B

---

*Cynoglossum tenerum* (Bornm.) Greuter & Stier in Biodivers. Data J. [hoc loco]. 2015

- ASSESSMENT: accepted
- STATUS: comb. nov.
- BASIS: Basionym: *Paracaryum tenerum* Bornm.

---

*Cynoglossum tenorei* of Gussone [Nyman, Consp. Fl. Eur. 3: 521. 1881] *(‘tenorii’)*

- IPNI: Cynoglossum tenorei Guss. ex Nyman -- Consp. Fl. Eur. 3: 521. 1881 [prob. Jul 1881] (IK)
- STATUS: nom. inval. [pro syn. sub *Cynoglossum nebrodense* subsp. *tenorei*]
- REFERENCE: Link

---

*Cynoglossum tetraspis* (Pall.) Greuter & Burdet in Willdenowia 11: 36. 1981

- IPNI: Cynoglossum tetraspis (Pallas) Greuter & Burdet -- Willdenowia 11(1): 36. 1981 (IK)
- ASSESSMENT: accepted
- STATUS: comb. nov.
- REFERENCE: Link
- BASIS: Basionym: *Rindera tetraspis* Pall.

---

*Cynoglossum thomsonii* (C. B. Clarke) I. M. Johnst. in Contr. Gray Herb. 73: 72. 1924

- IPNI: Cynoglossum thomsonii I.M.Johnst. -- Contr. Gray Herb. 73: 72. 1924 (IK)
- ASSESSMENT: accepted
- STATUS: comb. nov.
- REFERENCE: Link
- BASIS: Basionym: *Paracaryum thomsonii* C. B. Clarke

---

*Cynoglossum tianschanicum* Popov in Bot. Mater. Gerb. Bot. Inst. Komarova Akad. Nauk S.S.S.R. 14: 305. 1951

- IPNI: Cynoglossum tianschanicum Popov -- Bot. Mater. Gerb. Bot. Inst. Komarova Akad. Nauk S.S.S.R. 14: 305. 1951 (IK)
- ASSESSMENT: [*Cynoglossum capusii* (Franch.) Pazij]
- STATUS: spec. nov.
- REFERENCE: Link
- BASIS: Original material: Typus: Tian-schan occidentalis: ad fl. Dshebogly-su in jugo montium Talas Alatau dictorum, prope pagum Novo-Nikolajevka, VIII 1921, Popov et Abolin; in Herb. Universitätis Asiae Mediae (Taschkent) conservatur [from protologue]. Holotype: TASH

---

*Cynoglossum timorense* Riedl in Blumea 38: 463. 1994

- IPNI: Cynoglossum timorense Riedl -- Blumea 38(2): 463 (1994). (IK)
- ASSESSMENT: accepted
- STATUS: spec. nov.
- REFERENCE: Link
- BASIS: Original material: Typus: *van Steenis 18303* (L), Central Port. Timor, Mt. Perdido, 1750–2100 m, 23-xii-1953 [from protologue]

---

*Cynoglossum tomentosum* Lehm., Pl. Asperif. Nucif.: 144. 1818

- IPNI: Cynoglossum tomentosum Lehm. -- Pl. Asperif. Nucif. 1: 144. 1818 [Jul-early Sep 1818] (IK)
- ASSESSMENT: [*Cynoglossum clandestinum* Desf. (?)]
- STATUS: spec. nov.
- REFERENCE: Link
- BASIS: Original material: in Italia [from protologue]. Holotype presumably at MEL

---

*Cynoglossum tosaense* Nakai in Bot. Mag. (Tokyo) 37: 5. 1923

- IPNI: Cynoglossum tosaense Nakai -- Bot. Mag. (Tokyo) 1923, xxxvii. 5. (IK)
- ASSESSMENT: [*Cynoglossum asperrimum* Nakai]
- STATUS: spec. nov.
- REFERENCE: Link
- SYNONYMY:   
  ≡ *Cynoglossum asperrimum* var. *tosaense* (Nakai) H. Hara 1948
- BASIS: Original material: Shikoku: Dogamori prov. Tosa (T. Makino) in monte Hönokawa (S. Yano), Quelpaert: sine loco speciali (Faurie n. 2044) [from protologue]

---

*Cynoglossum trianaeum* Wedd., Chlor. And. 2: 90. 1859

- IPNI: Cynoglossum trianaeum Wedd. -- Chlor. And. 2: 90. 1859 [1857 publ. 10 Oct 1859] (IK)
- ASSESSMENT: Not *Cynoglossum* s. l.; genus uncertain (species accepted)
- STATUS: spec. nov.
- REFERENCE: Link
- BASIS: Original material: Nouvelle-Grenade: Cordillères de la province d’Antioquia, h. 3000 m (Triana) [from protologue]. Original specimen: Triana 3784, COL (COL000004064\*)

---

*Cynoglossum trinervium* (Duthie) Greuter & Stier in Biodivers. Data J. [hoc loco]. 2015

- ASSESSMENT: accepted
- STATUS: comb. nov.
- BASIS: Basionym: *Paracaryum trinervium*  Duthie

---

*Cynoglossum triste* Diels in Notes Roy. Bot. Gard. Edinburgh 5: 169. 1912

- IPNI: Cynoglossum triste Diels -- Notes Roy. Bot. Gard. Edinburgh 5: 169. 1912 (IK)
- ASSESSMENT: accepted
- STATUS: spec. nov.
- REFERENCE: Link
- BASIS: Original material: eastern flank of the Lichiang Range. Lat. 27°12' N., Alt. 10–11,000 ft. June 1906, G. Forrest No. 2235 [from protologue]. Presumed holotype: E (E00284595\*)

---

*Cynoglossum triumfetti* Perret ex Colla, Herb. Pedem. 4: 256. 1835 *(‘triunfetti’)*

- IPNI: Cynoglossum triunfetti Perret ex Colla -- Herb. Pedem. iv. 256 (1835). (IK)
- ASSESSMENT: Not *Cynoglossum* s.l. (genus and species doubtful)
- STATUS: spec. nov.
- REFERENCE: Link
- BASIS: Original material: None indicated in protologue. Type presumably at TO

---

*Cynoglossum trollii* Melch. in Notizbl. Bot. Gart. Berlin-Dahlem 14: 354. 1939

- IPNI: Cynoglossum trollii Melch. -- Notizbl. Bot. Gart. Berlin-Dahlem 14: 354. 1939 (IK)
- ASSESSMENT: *Pseudomertensia trollii* (Melch.) R. R. Stewart & Kazmi
- STATUS: spec. nov.
- REFERENCE: Link
- SYNONYMY:   
  ≡ *Pseudomertensia trollii* (Melch.) R. R. Stewart & Kazmi 1970
- BASIS: Original material: Kashmir: Koragbal, im Tannenwald und an Tannenwaldlichtungen, 2600 m. (blühend und fruchtend, 28. Juli 1937 – C. Troll n. 8090) [from protologue]. Holotype probably B†?

---

*Cynoglossum troodi* H. Lindb. in Acta Soc. Sci. Fenn., Ser. B, Opera Biol. 2: 27. 1946

- IPNI: Cynoglossum troodi H.Lindb. -- Acta Soc. Sci. Fenn., Ser. B, Opera Biol. 2(7): 27 (1946); K. H. Rechinger in Svensk Bot. Tidskr. xliii. 38 (1949), descr. ampl. (IK)
- ASSESSMENT: accepted
- STATUS: spec. nov.
- REFERENCE: Link
- BASIS: Original material: specimina in Herb. Lund a P. SINTENIS lecta (“n. 828 auf dem Gipfel des Troodos, 18.6.1880”, sub. nom. “Paracaryum myosotoides Boiss.” et a E. W. KENNEDY (“n. 695 Chionistra, 28.5.37” sub nom. “Cynoglossum montanum L.”) et in Herb. Helsingfors a E. W. KENNEDY lecta (“n. 694 Kryos Potamos, 24.5.37”). M. Troodos, in glareosis in declivi montis Chionistra. In pinet (P. pallasiana) et in ruderatis juxta “Olympus Camp Hotel” et in pineto juxta “Military Camp” [LINDBERG] [from protologue]. Lectotype (Meikle, Fl. Cyprus: 1126. 1985): *Sintenis & R igo 828*, LD (LD1420386\*); isolectotypes: *Sintenis* 828, K, LD (LD1410366\*), MPU (MPU019685\*); paralectotypes: H\*, LD\*, S\* (see also Väre in Phytotaxa 47: 37. 2012)

---

*Cynoglossum tsaratananense* J. S. Mill. in Adansonia, ser. 3, 27: 126. 2005

- IPNI: Cynoglossum tsaratananense J.S.Mill. -- Adansonia ser. 3, 27(1): 126 (115; figs. 2, 5). 2005 [30 Jun 2005]
- ASSESSMENT: accepted
- STATUS: spec. nov.
- REFERENCE: Link
- BASIS: Original material: Perrier de la Bâthie 16465, Madagascar, Prov. Antsiranana, massif de Tsaratanana, près des eaux, 2600 m, 13°57’S, 48°52’E, fl., fr., Apr. 1929 (holo-, P!; iso-, MO!, P!) [from protologue]. Holotype: P (P00465860\*); isotypes: MO, P (P00465859\*)

---

*Cynoglossum tschotkalense* (Popov) Greuter & Stier in Biodivers. Data J. [hoc loco]. 2015

- ASSESSMENT: accepted
- STATUS: comb. nov.
- BASIS: Basionym: *Rindera tschotkalensis* Popov

---

*Cynoglossum tubiflorum* (Murb.) Greuter & Burdet in Willdenowia 11: 36. 1981

- IPNI: Cynoglossum tubiflorum (Murb.) Greuter & Burdet -- Willdenowia 11(1): 36. 1981 (IK)
- ASSESSMENT: accepted
- STATUS: comb. nov.
- REFERENCE: Link
- BASIS: Basionym: *Solenanthus tubiflorus* Murb.

---

*Cynoglossum turcomanicum* (Bornm. & Sint.) Greuter & Stier in Biodivers. Data J. [hoc loco]. 2015

- ASSESSMENT: accepted
- STATUS: comb. nov.
- BASIS: Basionym: *Paracaryum turcomanicum* Bornm. & Sint.

---

*Cynoglossum turkestanicum* (Regel) Greuter & Stier in Biodivers. Data J. [hoc loco]. 2015

- ASSESSMENT: accepted
- STATUS: comb. nov.
- BASIS: Basionym: *Kuschakewiczia turkestanica* Regel

---

*Cynoglossum ukaguruense* Verdc. in Polhill, Fl. Trop. E. Africa, Boragin.: 113. 1991

- IPNI: Cynoglossum ukaguruense Verdc. -- Fl. Trop. E. Africa, Boragin. 113. 1991 (IK)
- ASSESSMENT: accepted
- STATUS: spec. nov.
- REFERENCE: Link
- BASIS: Original material: Tanzania, Kilosa District, Ukaguru Mts., Mabberley 1341 (K, holo.!, DSM, iso.!) + 1 Paratype + Fig. 32.3 [from protologue]. Holotype: K (K000418918\*)

---

*Cynoglossum umbellatum* Waldst. & Kit., Descr. Icon. Pl. Hung. 2: 158. 1803–1805

- IPNI: Cynoglossum umbellatum Waldst. & Kit. -- Descr. Icon. Pl. Hung. ii. 158. (IK)
- ASSESSMENT: accepted
- STATUS: spec. nov.
- REFERENCE: Link
- SYNONYMY:   
  ≡ *Mattia umbellata* (Waldst. & Kit.) Schult. 1809
- BASIS: Original material: in clivis arenosis Banatus inter Deliblát & Dubovátz + Tab. 148 [from protologue]. Lectotype (Kováts in Stud. Bot. Hung. 10: 123-133. 1975): BP; presumed isolectotype: B-W (B-W03339010\*)  
  LECTO: W. & K. 1805 VIII. No. 15 [Kováts 1975, 1992]. TYPE in Budapest

---

*Cynoglossum uncinatum* Royle ex Benth. in Royle, Ill. Bot. Himal. Mts.: 305. 1836

- IPNI: Cynoglossum uncinatum Royle ex Benth. -- Ill. Bot. Himal. Mts. [Royle] 1(9): 305. 1836 [May 1836] (IK)
- ASSESSMENT: *Hackelia uncinata* (Royle ex Benth.) C. E. C. Fischer
- STATUS: spec. nov.
- REFERENCE: Link
- SYNONYMY:   
  ≡ *Hackelia uncinata* (Royle ex Benth.) C. E. C. Fischer 1932
    
  ≡ *Paracaryum uncinatum* (Royle ex Benth.) N. P. Balakr. 1970
- BASIS: Original material: *Rindera glochidiata*. Wall. Cat. n. 926. Choor, Acharanda, Dokree in Kunawur [from protologue]. Syntypes: Wallich 926, E (E00288382\*), GZU (GZU000106060\*), NY (NY00335204\*)
- COMMENTS: For additional information see: *Cynoglossum laxum* G. Don and *Rindera glochidiata* of Wallich.

---

*Cynoglossum uncinatum* var. *laxiflorum* Benth. in Royle, Ill. Bot. Himal. Mts.: 305. 1836

- IPNI: absent (2014-11-05)
- ASSESSMENT: [*Hackelia uncinata* (Royle ex Benth.) C. E. C. Fischer]
- STATUS: var. nov.
- REFERENCE: Link
- BASIS: Original material: *C. macrophyllum* Royle MSS, Nagkanda, Tuen, Urrukta [from protologue]

---

*Cynoglossum valentinum* Lag., Gen. Sp. Pl.: 10. 1816

- IPNI: Cynoglossum valentinum Lag. -- Gen. Sp. Pl. [Lagasca] 10. 1816 (IK)
- ASSESSMENT: [*Cynoglossum dioscoridis* Vill.]
- STATUS: spec. nov.
- REFERENCE: Link
- BASIS: Original material: *B.* [beatus = †] *Cavanilles* legit in Regno Valentino [from protologue]. Presumed holotype: MA (MA95034\*)

---

*Cynoglossum valentinum* f. *castrilense* (Pau) Degen & Hervier in Bull. Acad. Int. Géogr. Bot. 17: 61. 1907

- IPNI: absent (2014-11-05)
- ASSESSMENT: [*Cynoglossum pustulatum* Boiss.]
- STATUS: comb. & stat. nov. [species to forma]
- REFERENCE: Link
- BASIS: Basionym: *Cynoglossum castrilense* Pau

---

*Cynoglossum vanense* Sutorý in Edinburgh J. Bot. 61: 119 (-126; figs. 1-4). 2005

- IPNI: Cynoglossum vanense Sutorý -- Edinburgh J. Bot. 61(2-3): 119 (-126; figs. 1-4). 2005 [2004 publ. 12 Aug 2005]
- ASSESSMENT: accepted
- STATUS: spec. nov.
- REFERENCE: Link
- BASIS: Original material: Turkey, C10 Hakkàri: Tureckij Kurdistan [Turkish Kurdistan]; Schamsdinan [Ṣemdinli]; Cheljane-çaj, 11 vi 1916, *A. B. Schelnivkov & V. Schiptschinskij 100-16*, Expeditio Urmiensis Musei Caucasici 1916 (holo, LE) + 6 paratypes + Fig. 1-3 [from protologue]. Holotype: LE (l.c.: 120, Fig. 1A\*)

---

*Cynoglossum velebiticum* of Borbás in Balaton Tud. Tanulmányozásának Eredm. 2 (2, 2)[= Balaton Fl. 2]: 220. 1900.

- IPNI: absent (2014-11-05)
- STATUS: nom. inval. [nom. nud., fide Sutorý in Acta Bot. Hung. 54: 212. 12012]
- REFERENCE: Link

---

*Cynoglossum velebiticum* Borbás ex K. Maly in Verh. K.K. Zool.-Bot. Ges. Wien 54: 239. 1904

- IPNI: Cynoglossum velebiticum Borbás ex K.Malý -- Verh. K.K. Zool.-Bot. Ges. Wien 54(3-4): 239. 1904 [25 May 1904]
- ASSESSMENT: [*Cynoglossum pustulatum* subsp. *parvifolium* (Vis.) Sutorý]
- STATUS: spec. nov.
- REFERENCE: Link
- BASIS: Original material: Borbás, Herb. – . Kroat.[ia]: In silvis ad Brussani [Brušane in Velebit Mts] (Borbás, 1881) [from protologue]. Lectotype: (Sutorý in Acta Bot. Hung. 54: 212. 2012) in sylvis ad Brussani, 18 July 1881 SARA (SARA40111); isolectotypes: BP (BP136461, BP564830, BP564845)

---

*Cynoglossum vernale* Salisb., Prodr. Stirp. Chap. Allerton: 116. 1796

- IPNI: Cynoglossum vernale Salisb. -- Prodr. Stirp. Chap. Allerton 116. 1796 [Nov-Dec 1796] (IK)
- ASSESSMENT: *Omphalodes omphaloides* (L.) Voss, nom. rej. prop.; *Omphalodes verna*  Moench, nom. cons. prop.
- STATUS: nom. illeg. [superfl.]
- REFERENCE: Link
- BASIS: Replaced synonym: *Cynoglossum omphaloides* L.

---

*Cynoglossum vesiculosum* of Wallich, Numer. List: 26. 1829

- IPNI: Cynoglossum vesiculosum Wall. -- Numer. List [Wallich] n. 920. 1829 (IK)
- STATUS: nom. inval. [nom. nud.]
- REFERENCE: Link

---

*Cynoglossum vesiculosum* Wall. ex G. Don, Gen. Hist. 4: 354. 1837–1838

- IPNI: Cynoglossum vesiculosum Wall. & G.Don -- Gen. Hist. iv. 354 (1837), descr. (IK)
- ASSESSMENT: Not *Cynoglossum* s. l.; genus uncertain [*“Cynoglossum” lanceolatum* Forssk.]
- STATUS: spec. nov.
- REFERENCE: Link
- BASIS: Original material: (Wall. cat. no. 920.) H. [in hothouse] Native of Nipaul [from protologue]

---

*Cynoglossum villosulum* Nakai in Bot. Mag. (Tokyo) 37: 6. 1923

- IPNI: Cynoglossum villosulum Nakai -- Bot. Mag. (Tokyo) 1923, xxxvii. 6. (IK)
- ASSESSMENT: [*Cynoglossum furcatum* Wall.]
- STATUS: spec. nov.
- REFERENCE: Link
- SYNONYMY:   
  ≡ *Cynoglossum furcatum* var. *villosulum* (Nakai) Riedl 1994
- BASIS: Original material: Tsushima: Nii (Y. Yabr). Hondo: Komono prov. Ise (R. Yatabe). Nikko prov. Shimotsuke (K. Sawada). Makinoyama prov. Idzumi (S. Matsuda). Kongo-san prov. Kawachi (T. Tada). Shikoku: Sakurayamamura prov. Iyo (T. Makino) [from protologue]

---

*Cynoglossum virens* of Linnaeus, Fl. Monsp.: 29. 1756

- IPNI: absent (2014-11-05)
- STATUS: nom. inval. [nom. nud.]
- REFERENCE: Link
- COMMENTS: See  ICN Art. 33 Ex. 1 for a rationale for the invalidity of this name. See also *Cynoglossum virens* L. (1759).

---

*Cynoglossum virens* L., Amoen. Acad. 4: 494. 1759

- IPNI: absent (2014-11-05)
- ASSESSMENT: *Cynoglossum* sp. (doubtful species)
- STATUS: spec. nov.
- REFERENCE: Link
- BASIS: Original material: [Alsatia], in monte prope Masmünster reperi & in hortum Illust. Principis transtuli: postmodum inter Rappelschvvilam & S. Mariam … unde Lutetiam & Stutgardiam in Illustr. Ducis hortum transmisi [I. Bauhin in C. Bauhin, Phytopinax: 496. 1596). Original specimen perhaps in herb. C. Bauhin, BAS
- COMMENTS: As explained by Linnaeus (l.c.) in a statement on p. 475, the names listed in the *Flora monspeliensis* thesis refer to species entries in Magnol’s *Botanicon monspeliense* – if one numbers them sequentially by hand in that book. *Cynoglossum virens*, which in the thesis is listed at the end among the “[species] obscuriores” (rather obscure species), is linked via its number (1284) to an entry in the Appendix of Magnol  (Bot. Monsp.: 294. 1676): “*Cynoglossa folio virenti* I. B.” [I. Bauhin & al., Hist. Pl. 3: 600. 1651]. In spite of all this uncertainty, the diagnostic statement “folio virenti” just suffices to validate the Linnean binomial (which has not been dealt with by the  Linnean Plant Name Typification Project). Original material must be sought among elements used by either Magnol [none extant] or I. Bauhin to coin that “description”. However, the illustration in Bauhin & al. (l.c.), a good likeness of *Asperugo procumbens* L., is not original material. Magnol (l.c.) states “figura transposita est”, and Bauhin & al. (l.c.) explain: “sculptor autem ineptus corrupit bonam fig. C. Bauh. phyt. Cynogl semper virens: Cynoglossum folio viridi Ioh. Bauhino”. The reference is to C. Bauhin (Phytopinax: 496. 1596), where “*Cynoglossum semperuiuens* is described, but there is no illustration of it, nor could we find one anywhere in the published writings of either of the Bauhin brothers.

---

*Cynoglossum virens* Schreb., Spic. Fl. Lips.: 152. 1771

- IPNI: Cynoglossum virens Schreb. -- Spicil. Fl. Lips. [152]. (IK)
- ASSESSMENT: [*Cynoglossum montanum* L.]
- STATUS: nom. illeg. [homonym]
- REFERENCE: Link
- BASIS: Original material: *Cynoglossa media altra flore rubro* Col. Ecphr. I. 175; An dem Damme bey dem Gunnewitzer Teiche. Colonna’s illustration is the subequently designated lectotype of *Cynoglossum montanum* L. (1753) [from  Böhmer, Fl. Lips. Indig.: 12. 1750 ]
- COMMENTS: Validated in a list of names, in which the number 26 refers to Böhmer (l.c.). Böhmer has a diagnosis of his own, validating Schreber’s name.

---

*Cynoglossum virginianum* L., Sp. Pl.: 134. 1753

- IPNI: Cynoglossum virginianum L. -- Sp. Pl. 1: 134. 1753 [1 May 1753] (IK)
- ASSESSMENT: Not *Cynoglossum* s. l.; genus uncertain (species accepted)
- STATUS: spec. nov.
- REFERENCE: Link
- BASIS: Original material: *Cynoglossum foliis amplexicaulibus*. Gron. virg. 19. … in Virginia [from protologue]. Lectotype (Wells in Taxon 53: 802. 2004): Clayton 257 (BM000040317\*)
- COMMENTS: See also comments under *Myosotis virginiana*  L. (1753).

---

*Cynoglossum virginianum* var. *boreale* (Fernald) Cooperr. in Michigan Bot. 23: 166. 1984

- IPNI: Cynoglossum virginianum L. var. boreale (Fernald) Cooperr. -- Michigan Bot. 23(4): 166 (1984):. (IK)
- ASSESSMENT: Not *Cynoglossum* s. l.; genus uncertain [*“Cynoglossum” virginianum* subsp. *boreale* (Fernald) A. Haines]
- STATUS: comb. & stat. nov. [species to variety]
- REFERENCE: Link
- BASIS: Basionym: *Cynoglossum boreale* Fernald

---

*Cynoglossum virginianum* subsp. *boreale* (Fernald) A. Haines in Stantec Bot. Notes 13: 3. 2010

- IPNI: Cynoglossum virginianum L. subsp. boreale (Fernald) A.Haines -- Stantec Bot. Notes 13: 3. 2010 [8 Sep 2010]
- ASSESSMENT: Not *Cynoglossum* s. l.; genus uncertain (subspecies accepted)
- STATUS: stat. nov. [species via variety to subspecies]
- REFERENCE: Link
- BASIS: Basionym: *Cynoglossum boreale* Fernald

---

*Cynoglossum virginicum* L., Syst. Nat., ed. 12, 2: 146. 1767

- IPNI: Cynoglossum virginicum L. -- Syst. Nat., ed. 12. 2: 146. 1767 [15-31 Oct 1767] (IK)
- ASSESSMENT: Not *Cynoglossum* s. l.; genus uncertain [*“Cynoglossum” virginianum* L.]
- STATUS: nom. illeg. [superfl.]
- REFERENCE: Link
- BASIS: Replaced synonym: *Cynoglossum virginianum* L.

---

*Cynoglossum virginicum* of J. F. Gmelin, Syst. Nat. 2: 319. 1791

- IPNI: Cynoglossum virginicum J.F.Gmel. -- Syst. Nat., ed. 13[bis]. 2(1): 319. 1791 [late Sep-Nov 1791] (IK)
- STATUS: [isonym]
- REFERENCE: Link

---

*Cynoglossum virginicum* of Willdenow, Enum. Pl.: 180. 1809

- IPNI: Cynoglossum virginicum Willd. -- Enum. Pl. [Willdenow] 1: 180. 1809 [Apr 1809] (IK)
- STATUS: [isonym]
- REFERENCE: Link

---

*Cynoglossum viride* Eastw. in Proc. Calif. Acad. Sci., ser. 2, 6: 428. 1896

- IPNI: Cynoglossum viride Eastw. -- Proc. Calif. Acad. Sci. ser. 2, 6: 428, pl. 59. 1896 (GCI)
- ASSESSMENT: Not *Cynoglossum* s. l.; genus uncertain [*“Cynoglossum” occidentale* A. Gray]
- STATUS: spec. nov.
- REFERENCE: Link
- BASIS: Original material: Collected by Mrs. R. M. Austin, in the Cascade Mountains, Oregon … by Mr. Brandegee, at the Calaveras Grove of Big Trees … by the writer [Eastwood] at Sequoia Mills, Fresno County, May 20, 1894 … by Mr. Brandegee, at Frazer’s Mills. Type specimens all in the Herbarium of the California Academy of Sciences. Duplicates have been sent to the Gray Herbarium and to the National Herbarium [from protologue]. Syntype: 20 May 1895, Eastwood, CAS (CAS 0007280\*)

---

*Cynoglossum viridiflorum* Lehm., Pl. Asperif. Nucif.: 160. 1818

- IPNI: absent (2014-11-05)
- ASSESSMENT: accepted
- STATUS: spec. nov.
- REFERENCE: Link
- BASIS: Original material: H. W. [herb. Willdenow]; [Siberia] in locis arenosis prope Schemanaika et prope Schulba [*Pallas*] (v. s.) [from protologue]. Holotype: HAL (HAL0115101)
- COMMENTS: The specimen in HAL comes from the Herbarium Willdenow, where the species is lacking. The sheet is annotated in Willdenow’s handwriting: “Cynoglossum viridiflorum in humosis Sibiria Pallas Willd.” The source of Lehmann’s locality data should be looked for in Pallas (Reise Russ. Reich. 1771—1776).

---

*Cynoglossum viridiflorum* of Willdenow [Roem. & Schult., Syst. Veg., ed. 15 bis 4: 757. 1819]

- IPNI: Cynoglossum viridiflorum Willd. ex Roem. & Schult. -- Syst. Veg., ed. 15 bis [Roemer & Schultes] 4: 757. 1819 (IK)
- STATUS: [isonym]
- REFERENCE: Link

---

*Cynoglossum vulgare* L., Fl. Belg.: 14. 1760

- IPNI: absent (2014-11-05)
- ASSESSMENT: [*Cynoglossum officinale* L.]
- STATUS: spec. nov.
- REFERENCE: Link
- BASIS: Original material: *Cynoglossum foliis ovato-lanceolatis*. Linn. h. Cliff. 47. *corollis calycem aequantibus* Roy. lugdb. 406. [from  Gorter, Fl. Gelro-Zutph.: 32. 1745]. Original specimens: herb. Clifford, BM; herb. Royen, L.
- COMMENTS: There is no descriptive matter in the thesis, but the name (overlooked by the Linnean Plant Name Typification Project) is validated by numerical references to the treatments in two earlier works, by Commelijn (Cat. Pl. Indig. Hollandiae. 1709; not seen) and Gorter (1745; cited above). For all events and purposes *Cynoglossum vulgare* is a synonym of, and likely error for, *C. officinale* L. (1753); but it is technically legitimate, as the latter name is not mentioned, nor had its type been designated. The current lectotype of *C. officinale* qualifies as possible lectotype of *C. vulgare*, which would make the two names homotypic.

---

*Cynoglossum vulgare* of Gueldenstaedt, Reis. Russland 1: 420. 1787

- IPNI: absent (2014-11-05)
- STATUS: nom. inval. [nom. nud.]
- REFERENCE: Link

---

*Cynoglossum vulgare* of Gueldenstaedt [Ledeb., Fl. Ross. 3: 165. 1847]

- IPNI: Cynoglossum vulgare Gueldenst. ex Ledeb. -- Fl. Ross. (Ledeb.) 3(1,8): 165. 1847 [Oct 1847] (IK)
- STATUS: nom. inval. [pro syn. sub *Cynoglossum officinale*]
- REFERENCE: Link

---

*Cynoglossum vulgare* of Linnaeus [B. D. Jacks, Index Linn. Herb.: 66. 1912]

- IPNI: Cynoglossum vulgare L. ex B.D.Jacks. -- Index Linn. Herb. 66 (1912), non en. (IK)
- STATUS: [isonym]
- REFERENCE: Link

---

*Cynoglossum wallichii* G. Don, Gen. Hist. 4: 354. 1837–1838

- IPNI: Cynoglossum wallichii G.Don -- Gen. Hist. iv. 354. (IK)
- ASSESSMENT: accepted
- STATUS: spec. nov.
- REFERENCE: Link
- BASIS: Original material: Native of Nipaul, in Gosainsthan. *C. calycinum*, Wall. cat. no. 923 [from protologue]. Lectotype (Kazmi in J. Arnold Arbor. 52: 345. 1971, as “type”): *Wallich 923*, K ; possible isotypes: A (A00092800\*), BM (BM000603178\*)

---

*Cynoglossum wallichii* var. *alpinum* Clarke in Hooker, Fl. Brit. India 4: 157. 1883 *(‘alpina’)*

- IPNI: absent (2014-11-05)
- ASSESSMENT: [*Cynoglossum wallichii* G. Don]
- STATUS: var. nov.
- REFERENCE: Link
- SYNONYMY:   
  ≡ *Cynoglossum glochidiatum* var. *alpinum* (Clarke) Brand 1921
- BASIS: Original material: *Cynoglossum* sp. N. 7, Herb. Ind. Or. H. f. & T. – W. Subalpine Himalaya, Edgeworth, etc. [from protologue]

---

*Cynoglossum wallichii* var. *glochidiatum* (Wall. ex Benth.) Kazmi in J. Arnold Arbor. 52: 347. 1971

- IPNI: Cynoglossum wallichii G.Don var. glochidiatum (Wall. ex Benth.) Kazmi -- J. Arnold Arbor. 52(2): 347. 1971 (IK)
- ASSESSMENT: [*Cynoglossum wallichii* G. Don]
- STATUS: comb. & stat. nov. [species to variety]
- REFERENCE: Link
- BASIS: Basionym: *Cynoglossum glochidiatum* Wall. ex Benth.

---

*Cynoglossum watieri* (Batt. & Maire) Braun-Blanq. & Maire in Bull. Soc. Hist. Nat. Afrique N. 13: 19. 1922

- IPNI: Cynoglossum watieri Braun-Blanq. & Maire -- Bull. Soc. Hist. Nat. Afrique N. 1922, xiii. 19. (IK)
- ASSESSMENT: accepted
- STATUS: comb. nov.
- REFERENCE: Link
- BASIS: Basionym: *Solenanthus watieri* Batt. & Maire

---

*Cynoglossum wildii* E. S. Martins in Garcia de Orta, Ser. Bot. 9: 76. 1988

- IPNI: Cynoglossum wildii E.S.Martins -- Garcia de Orta, Ser. Bot. 9(1-2): 76. 1988 [1987 publ. 1988] (IK)
- ASSESSMENT: accepted
- STATUS: spec. nov.
- REFERENCE: Link
- BASIS: Original material: Zimbabwe, Mutare (Umtali), Himalayas, Engwa, *H. Wild 4437* (K, holotypus; LISC, SRGH) [from protologue]. Holotype: K (k000418913\*); isotypes: BR (BR0000008865779\*), LISC (LISC011308\*), PRE (PRE0659363-0)

---

*Cynoglossum xatarti* of J. Gay [Nyman, Consp. Fl. Eur. 3: 521. 1881]

- IPNI: Cynoglossum xatarti J.Gay ex Nyman -- Consp. Fl. Eur. 3: 521. 1881 [prob. Jul 1881] (IK)
- STATUS: nom. inval. [nom. nud., pro subvar. sub *Cynoglossum dioscoridis*]
- REFERENCE: Link

---

*Cynoglossum yemenense* (R. R. Mill & A. G. Mill.) Verdc. in Polhill, Fl. Trop. E. Africa, Boragin.: 110. 1991

- IPNI: Cynoglossum yemenense (R.R.Mill & A.G.Mill.) Verdc. -- Fl. Trop. E. Africa, Boragin. 110. 1991
- ASSESSMENT: Not *Cynoglossum* s. l.; genus uncertain (species accepted)
- STATUS: comb. nov.
- REFERENCE: Link
- BASIS: Basionym: *Paracynoglossum yemenense* R. R. Mill & A. G. Mill.

---

*Cynoglossum yemenense* of J. R. I. Wood, Handb. Yemen Fl.: 241. 1997

- IPNI: Cynoglossum yemenense (R.R.Mill & A.G.Mill.) J.R.I.Wood -- Handb. Yemen Fl. 241. 1997 ; nom. inval. (IK)
- STATUS: [isonym]
- REFERENCE: Link

---

*Cynoglossum zeylanicum* of Thunberg [Lehmann in Neue Schriften Naturf. Ges. Halle 3: 21. 1817]

- IPNI: absent (2014-11-05)
- STATUS: nom. inval. [pro syn. sub *Myosotis zeylanica*]
- REFERENCE: Link
- COMMENTS: See also *Cynoglossum ceilanicum* of Thunberg, nom nud.

---

*Cynoglossum zeylanicum* of Thunberg [Lehm., Pl. Asperif. Nucif.: 116. 1818]

- IPNI: Cynoglossum zeylanicum Thunb. ex Lehm. -- Pl. Asperif. Nucif. 1: 116. 1818 [Jul-early Sep 1818] ; nom. inval. (IK)
- STATUS: nom. inval. [pro syn. sub *Echinospermum zeylanicum*]
- REFERENCE: Link
- COMMENTS: See also *Cynoglossum ceilanicum* of Thunberg, nom nud.

---

*Cynoglossum zeylanicum* of Wight [Wall., Numer. List: 240. 1832]

- IPNI: Cynoglossum zeylanicum Wight ex Wall. -- Numer. List [Wallich] n. 7018. 1832 (IK)
- STATUS: nom. inval. [nom. nud.]
- REFERENCE: Link

---

*Cynoglossum zeylanicum* (Lehm.) Brand in Repert. Spec. Nov. Regni Veg. 13: 546. 1915

- IPNI: absent (2014-11-05)
- ASSESSMENT: accepted
- STATUS: comb. nov.
- REFERENCE: Link
- BASIS: Basionym: *Myosotis zeylanica*  Lehm.; Replaced synonym: *Anchusa zeylanica* Vahl ex Hornem., non *Anchusa zeylanica* J. Jacq.
- COMMENTS: Lacking direct reference to the basionym, which is referred to indirectly (via “*Cynoglossum zeylanicum* Thunb.”, mentioned as a synonym in the protologue of Myosotis zeylanica). ICN Art. 41.4 also ensures valid publication of the intended new combination.

---

*Cynoglossum zeylanicum* f. *albiflorum* H. Hara, Enum. Spermatophytarum Japon. 1: 172. 1948

- IPNI: Cynoglossum zeylanicum Thunb. ex Lehm. f. albiflorum H.Hara -- Enum. Spermatophytarum Japon. 1: 173. 1948
- ASSESSMENT: [*Cynoglossum zeylanicum* (Lehm.) Brand]
- STATUS: f. nov.
- REFERENCE: Link
- SYNONYMY:   
  ≡ *Cynoglossum furcatum* f. *albiflorum* (H. Hara) Yonek.
- BASIS: Original material: cannot be identified in protologue

---

*Cynoglossum zeylanicum* var. *lanceolatum* (C. B. Clarke) Brand in Engler, Pflanzenr. 78 (IV.252): 135. 1921

- IPNI: absent (2014-11-05)
- ASSESSMENT: [*Cynoglossum furcatum* Wall.]
- STATUS: comb. nov.
- REFERENCE: Link
- BASIS: Basionym: *Cynoglossum furcatum* var. *lanceolatum* C. B. Clarke; Replaced synonym: *Cynoglossum heynei* G. Don

---

*Cyphomattia* Boiss., Fl. Orient. 4: 272. 1875

- IPNI: Cyphomattia Boiss. -- Fl. Orient. [Boissier] 4(1): 272. 1875 [Sep-Oct 1875] (IK)
- ASSESSMENT: [*Cynoglossum* L.]
- STATUS: gen. nov.
- REFERENCE: Link
- SYNONYMY:   
  ≡ *Rindera* sect. *Cyphomattia* (Boiss.) Kusn. 1910
- BASIS: Type (designated on ING card, date 1996-02-09): *Cyphomattia lanata* (Lam.) Boiss.

---

*Cyphomattia korshinskyi* Lipsky in Trudy Imp. S.-Peterburgsk. Bot. Sada 26: 511. 1910

- IPNI: Cyphomattia korshinskyi Lipsky -- Trudy Imp. S.-Peterburgsk. Bot. Sada xxvi. 511 (1910). (IK)
- ASSESSMENT: *Cynoglossum korshinskyi* (Lipsky) Greuter & Stier
- STATUS: spec. nov.
- REFERENCE: Link
- SYNONYMY:   
  ≡ *Rindera korshinskyi* (Lipsky) O. Fedtsch. & B. Fedtsch. 1913
    
  ≡ *Cynoglossum korshinskyi* (Lipsky) Greuter & Stier 2015
- BASIS: Original material: Каратегинъ: Между Гармомъ и Немичи, вдоль по р. Сухобъ, на кам. и глин. склонахъ, пл. и ост. цвѣт. 20 іюня 1897 (*Коржинскій*). Дамбурачи, у устья Мук-су, на каменист. склонахъ, плод. и цв. 23 іюня 1879 (*Коржинскій*) [from protologue]. Syntypes presumably in LE

---

*Cyphomattia lanata* (Lam.) Boiss., Fl. Orient. 4: 272. 1875

- IPNI: Cyphomattia lanata Boiss. -- Fl. Orient. [Boissier] 4(1): 272. 1875 [Sep-Oct 1875] (IK)
- ASSESSMENT: *Cynoglossum lanatum* Lam.
- STATUS: comb. nov.
- REFERENCE: Link
- BASIS: Basionym: *Cynoglossum lanatum* Lam.

---

*Cyphomattia lanata* var. *brachyantha* (Boiss.) Boiss., Fl. Orient. 4: 273. 1875

- IPNI: absent (2014-11-05)
- ASSESSMENT: [*Cynoglossum lanatum* Lam.]
- STATUS: comb. & stat. nov. [species to variety]
- REFERENCE: Link
- BASIS: Basionym: *Mattia brachyantha* Boiss.

---

*Echinospermum borbonicum*  (Lam.) Lehm., Pl. Asperif. Nucif.: 119. 1818

- IPNI: Echinospermum borbonicum Lehm. -- Pl. Asperif. Nucif. 1: 119. 1818 [Jul-early Sep 1818] (IK)
- ASSESSMENT: [*Cynoglossum borbonicum* Bory]
- STATUS: comb. nov.
- REFERENCE: Link
- BASIS: Basionym: *Myosotis borbonica*  Lam.

---

*Echinospermum bungei* Boiss., Fl. Orient. 4: 252. 1875

- IPNI: Echinospermum bungei Boiss. -- Fl. Orient. [Boissier] 4(1): 252. 1875 [Sep-Oct 1875] (IK)
- ASSESSMENT: [*Microparacaryum intermedium* (Fresen.) Hilger & Podlech]
- STATUS: spec. nov.
- REFERENCE: Link
- SYNONYMY:   
  ≡ *Paracaryum bungei* (Boiss.) Brand 1921
    
  ≡ *Mattiastrum bungei* (Boiss.) Rech. f. & Riedl 1963
- BASIS: Original material: in Persiâ prope Scharud (Bge!) [from protologue]

---

*Echinospermum calathicarpum* Stocks in Hooker's J. Bot. Kew Gard. Misc. 4: 175. 1852

- IPNI: Echinospermum calathicarpum Stocks -- Hooker's J. Bot. Kew Gard. Misc. 4: 175. 1852 (IK)
- ASSESSMENT: [*Microparacaryum intermedium* (Fresen.) Hilger & Podlech]
- STATUS: spec. nov.
- REFERENCE: Link
- SYNONYMY:   
  ≡ *Paracaryum calathicarpum* (Stocks) Boiss. 1875
    
  ≡ *Paracaryum intermedium* var. *calathicarpum* (Stocks) Y. J. Nasir 1989
- BASIS: Original material: Upper Beloochistan. [Stocks] No. 1003

---

*Echinospermum californicum*  A. Gray in Proc. Amer. Acad. Arts 17: 225. 1882

- IPNI: Echinospermum californicum A.Gray -- Proc. Amer. Acad. Arts xvii. (1881-82) 225. (IK)
- ASSESSMENT: *Hackelia californica*  (A. Gray) I. M. Johnst.
- STATUS: spec. nov.
- REFERENCE: Link
- SYNONYMY:   
  ≡ *Cynoglossum californicum* (A. Gray) A. Gray 1886
    
  ≡ *Hackelia californica*  (A. Gray) I. M. Johnst. 1923
- BASIS: Original material: Sierra Nevada, California, from Mount Shasta southward. This was taken for Lehmann's E. diffusum, because of his description of the corolla (“Corolla alba ? magna, tubus calyce paullo longior sensim ampliatus”) ; and Californian specimens of the real E. diffusum were mixed with it. The original specimens of the latter do not have the exserted tube of the corolla which marks the present species when in blossom, as does the fruit at maturity. It is the E. nervosum of Kellogg ; but neither the leaves nor the sepals are perceptibly nervose (the former not “3-5-nerved” nor the latter “3-nerved”), so that the name would be a false one [from protologue]

---

*Echinospermum enerve*  E. Mey. ex Drège in Flora 23(2), Beilage: 180. 1843

- IPNI: Echinospermum enerve E.Mey. ex DC. -- Prodr. [A. P. de Candolle] 10: 154. 1846 [8 Apr 1846] (IK)
- ASSESSMENT: [*Cynoglossum hispidum* Thunb.]
- STATUS: spec. nov.
- REFERENCE: Link
- SYNONYMY:   
  ≡ *Cynoglossum enerve* (E. Mey. ex Drège) Turcz. ex B. D. Jacks.
- BASIS: Original material: une plante du Cap (*Echinospermum enerve* E. Mey.in Drege collect.) [from protologue]. Holotype or syntypes presumably at KW
- COMMENTS: Validated by indirect reference, through E. Meyer’s initials, to Turczaninov’s description of 1840 (see under *Cynoglossum enerve* ascribed to Turczaninov ). The author of the “Beilage”, except for the introduction, is Drège; the name is ascribed to “E. M.”; but the validating description is Turczaninov’s. Hence, the author of the name is Drège (ICN Art. 46.5).

---

*Echinospermum javanicum*  Lehm., Pl. Asperif. Nucif.: 118. 1818

- IPNI: Echinospermum javanicum Lehm. -- Pl. Asperif. Nucif. 1: 118. 1818 [Jul-early Sep 1818] (IK)
- ASSESSMENT: *Cynoglossum javanicum* (Lehm.) A. DC.
- STATUS: spec. nov.
- REFERENCE: Link
- SYNONYMY:   
  ≡ *Rochelia javanica*  (Lehm.) Roem. & Schult. 1819
    
  ≡ *Cynoglossum javanicum* (Lehm.) A. DC. 1846
- BASIS: Original material: in insula Java. (Thunberg.) (v. s.) [from protologue]

---

*Echinospermum latifolium*  Hochst. ex A. Rich., Tent. Fl. Abyss. 2: 89. 1850

- IPNI: Echinospermum latifolium Hochst. ex A.Rich. -- Tent. Fl. Abyss. 2: 89. 1850 (IK)
- ASSESSMENT: *Cynoglossopsis latifolia*  (Hochst. ex A. Rich.) Brand
- STATUS: spec. nov.
- REFERENCE: Link
- SYNONYMY:   
  ≡ *Cynoglossum hochstetteri* Vatke ex Engl. 1892
    
  ≡ *Cynoglossopsis latifolia*  (Hochst. ex A. Rich.) Brand 1931
- BASIS: Original material: [Abyssinia] in rupibus prope Mai-Mezanno, in districtu Tchélatchékanné mense augusto florens (Schimper) … pl. Schimp. Abyss., sect. iii, no 1447 [from protologue]. Holotype: P; isotypes: K (K000418897\*), M (M0188101\*), MPU (MPU002549\*), S (S12-5613\*), TUB (TUB003673\*, TUB003674\*)

---

*Echinospermum patulum*  Lehm., Pl. Asperif. Nucif.: 124. 1818

- IPNI: Echinospermum patulum Lehm. -- Pl. Asperif. Nucif. 1: 124. 1818 [Jul-early Sep 1818] (IK)
- ASSESSMENT: *Lappula patula* (Lehm.) Asch. ex Gürke
- STATUS: spec. nov.
- REFERENCE: Link
- SYNONYMY:   
  ≡ *Cynoglossum patulum* (Lehm.) E. H. L. Krause 1903
    
  ≡ *Lappula patula* (Lehm.) Asch. ex Gürke 1894
- BASIS: Original material: Based on several misapplications of *Lappula squarrosa* Retz. (1781); in deserto Caucasico-Caspico, ad Kumam et Terek nec non ad Wolgam inferiorem circa Sareptam coloniam et urbem Astrachan (v. v.) [from protologue]

---

*Echinospermum zeylanicum* (Lehm.) Lehm., Pl. Asperif. Nucif.: 116. 1818

- IPNI: Echinospermum zeylanicum (Vahl) Lehm. -- Pl. Asperif. Nucif. 1: 116. 1818 [Jul-early Sep 1818] (IK)
- ASSESSMENT: *Cynoglossum zeylanicum* (Lehm.) Brand
- STATUS: comb. nov.
- REFERENCE: Link
- BASIS: Basionym: *Myosotis zeylanica*  Lehm.; Replaced synonym: *Anchusa zeylanica* Vahl ex Hornem., non *Anchusa zeylanica* J. Jacq.

---

*Echium glaucophyllum* Jacq., Collectanea 2: 325. 1789

- IPNI: Echium glaucophyllum Jacq. -- Coll. ii. 325. (IK)
- ASSESSMENT: *Lobostemon glaucophyllus* (Jacq.) Buek
- STATUS: spec. nov.
- REFERENCE: Link
- SYNONYMY:   
  ≡ *Lobostemon glaucophyllus* (Jacq.) Buek 1837
- BASIS: Original material: Ad promontorium bonae Spei [from protologue]

---

*Echium papillosum* Thunb., Fl. Cap. 2: 8. 1818

- IPNI: Echium papillosum Thunb. -- Fl. Cap. ii. 8. (IK)
- ASSESSMENT: [*Lobostemon glaucophyllus* (Jacq.) Buek]
- STATUS: spec. nov. (or perhaps nom. illeg. [homonym of *Echium papillosum* Lehm. 1818 (Sep.-Oct.)])
- REFERENCE: Link
- SYNONYMY:   
  ≡ *Cynoglossum papillosum* (Thunb.) Thunb. 1820
- BASIS: Original material: [S. Africa], not indicated in protologue

---

*Eritrichium howardii*  (A. Gray) Rydb. in Mem. New York Bot. Gard. 1: 327. 1900

- IPNI: Eritrichium howardii Rydb. -- Mem. New York Bot. Gard. i. 327. (IK)
- ASSESSMENT: accepted
- STATUS: comb. nov.
- REFERENCE: Link
- BASIS: Basionym: *Cynoglossum howardii* A. Gray

---

*Gruvelia pusilla* A. DC. in Candolle, Prodr. 10: 119. 1846

- IPNI: Gruvelia pusilla A.DC. -- Prodr. [A. P. de Candolle] 10: 119. 1846 [8 Apr 1846] (IK)
- ASSESSMENT: *Pectocarya pusilla* (A. DC.) A. Gray
- STATUS: spec. nov.
- REFERENCE: Link
- SYNONYMY:   
  ≡ *Pectocarya pusilla* (A. DC.) A. Gray 1877
- BASIS: Original material: in Chili (Poepp. n. 276. diar.) prope Valparaiso et montem la Leona (Bert.! n. 212). Cynoglossum pauciflorum? Bert.! herb. et merc. chil. 1829. apr. p. 510. DC. mss. non Ruiz et Pav. [from protologue]. Syntypes: *Bertero 212*, G-DC (G00204600, G00204601), GH (GH00073065, GH00073066), HAL (HAL0115130), M (M0174208); *Poeppig 276*, G-DC (G00204602)

---

*Hackelia andicola* (K. Krause) Brand in Engler, Pflanzenr. 97 (IV.252): 120. 1931

- IPNI: Hackelia andicola (K.Krause) Brand -- Pflanzenr. (Engler) Borrag.-Borraginoid.-Cryptanth. 120 (1931). (IK)
- ASSESSMENT: accepted
- STATUS: comb. nov.
- REFERENCE: Link
- BASIS: Basionym: *Cynoglossum andicola* K. Krause

---

*Hackelia brachytuba* (Diels) I. M. Johnst. in J. Arnold Arbor. 18: 25. 1937

- IPNI: Hackelia brachytuba (Diels) I.M.Johnst. -- J. Arnold Arbor. 1937, xviii. 25. (IK)
- ASSESSMENT: accepted
- STATUS: comb. nov.
- REFERENCE: Link
- BASIS: Basionym: *Paracaryum brachytubum* Diels

---

*Hackelia californica*  (A. Gray) I. M. Johnst. in Contr. Gray Herb. 68: 47. 1923

- IPNI: Hackelia californica I.M.Johnst. -- Contr. Gray Herb. 68: 47. 1923 (IK)
- ASSESSMENT: accepted
- STATUS: comb. nov.
- REFERENCE: Link
- BASIS: Basionym: *Echinospermum californicum*  A. Gray

---

*Hackelia ciliata*  (Douglas ex Lehm.) I. M. Johnst. in Contr. Gray Herb. 68: 46. 1923

- IPNI: Hackelia ciliata I.M.Johnst. -- Contr. Gray Herb. 68: 46. 1923 (GCI)
- ASSESSMENT: accepted
- STATUS: comb. nov.
- REFERENCE: Link
- BASIS: Basionym: *Cynoglossum ciliatum* Douglas ex Lehm.

---

*Hackelia deflexa*  (Wahlenb.) Opiz in Berchtold & al., Oekon.-Techn. Fl. Böhm. 2(2): 147. 1839

- IPNI: Hackelia deflexa Opiz -- Bercht. & Opiz, Okon.-techn. Fl. Bohm. ii II. 147 (1838); Seznam, 49. (IK)
- ASSESSMENT: accepted
- STATUS: comb. nov.
- REFERENCE: Link
- BASIS: Basionym: *Myosotis deflexa*  Wahlenb.

---

*Hackelia hintoniorum*  (B. L. Turner) Sutorý in Novon 20: 464. 2010

- IPNI: Hackelia hintoniorum (B.L.Turner) Sutorý -- Novon 20(4): 464. 2010 [29 Nov 2010]
- ASSESSMENT: accepted
- STATUS: comb. nov.
- REFERENCE: Link
- BASIS: Basionym: *Cynoglossum hintoniorum* B. L. Turner

---

*Hackelia mexicana*  (Cham. & Schltdl.) I. M. Johnst. in Contr. Gray Herb. 68: 46. 1923

- IPNI: Hackelia mexicana I.M.Johnst. -- Contr. Gray Herb. 68: 46. 1923 (IK)
- ASSESSMENT: accepted
- STATUS: comb. nov.
- REFERENCE: Link
- BASIS: Basionym: *Cynoglossum mexicanum* Cham. & Schltdl.

---

*Hackelia parviflora* (K. Krause) Brand in Engler, Pflanzenr. 97 (IV.252): 122. 1931

- IPNI: Hackelia parviflora Brand -- Pflanzenr. (Engler) [Heft 97] 4, Fam. 252: 122. 1931 (GCI)
- ASSESSMENT: accepted
- STATUS: comb. nov.
- REFERENCE: Link
- BASIS: Basionym: *Cynoglossum parviflorum* K. Krause

---

*Hackelia revoluta*  (Ruiz & Pav.) I. M. Johnst. in Contr. Gray Herb. 68: 45. 1923

- IPNI: Hackelia revoluta I.M.Johnst. -- Contr. Gray Herb. 68: 45. 1923 (GCI)
- ASSESSMENT: accepted
- STATUS: comb. nov.
- REFERENCE: Link
- BASIS: Basionym: *Cynoglossum revolutum* Ruiz & Pav.

---

*Hackelia uncinata* (Royle ex Benth.) C. E. C. Fischer in Bull. Misc. Inform. Kew 1932: 298. 1932

- IPNI: Hackelia uncinata (Royle ex Benth.) C.E.C.Fisch. -- Bull. Misc. Inform. Kew 1932(6): 298. [27 Jul 1932] (IK)
- ASSESSMENT: accepted
- STATUS: comb. nov.
- REFERENCE: Link
- BASIS: Basionym: *Cynoglossum uncinatum* Royle ex Benth.

---

*Hackelia virginiana*  (L.) I. M. Johnst. in Contr. Gray Herb. 68: 45. 1923

- IPNI: Hackelia virginiana I.M.Johnst. -- Contr. Gray Herb. 68: 45. 1923 (IK)
- ASSESSMENT: accepted
- STATUS: comb. nov.
- REFERENCE: Link
- BASIS: Basionym: *Myosotis virginiana*  L.

---

*Kuschakewiczia* Regel & Smirn. in Trudy Imp. S.-Peterburgsk. Bot. Sada 5: 625. 1877

- IPNI: Kuschakewiczia Regel & Smirn. -- Trudy Imp. S.-Peterburgsk. Bot. Sada v. (1877) 625. (IK)
- ASSESSMENT: [*Cynoglossum* L.]
- STATUS: gen. nov.
- REFERENCE: Link
- SYNONYMY:   
  ≡ *Solenanthus*  sect. *Kuschakewiczia* (Regel & Smirn.) Zakirov 1941
- BASIS: Original type: *Kuschakewiczia turkestanica* Regel

---

*Kuschakewiczia turkestanica* Regel in Trudy Imp. S.-Peterburgsk. Bot. Sada 5: 626. 1877

- IPNI: Kuschakewiczia turkestanica Regel & Smirn. -- Trudy Imp. S.-Peterburgsk. Bot. Sada v. (1877) 626. (IK)
- ASSESSMENT: *Cynoglossum turkestanicum* (Regel) Greuter & Stier
- STATUS: spec. nov.
- REFERENCE: Link
- SYNONYMY:   
  ≡ *Solenanthus kuschakewiczii* Lipsky 1904, nom. illeg.
    
  ≡ *Solenanthus turkestanicus* (Regel) Kusn. 1913
    
  ≡ *Cynoglossum turkestanicum* (Regel) Greuter & Stier 2015
- BASIS: Original material: In Turkestania prope urbem Taschkent (Kuschakewicz), in montibus karatavicis prope Ak-su (Sewerzow) [from protologue]. – Lectotype (Popov in Komarov, Fl. SSSR 19: 647. 1953, as “tip”): Taškent, Kušakewič, LE

---

*Lappula* Moench, Methodus: 416. 1794

- IPNI: Lappula Moench -- Methodus (Moench) 416 (1794). [4 May 1794] (IK)
- ASSESSMENT: accepted
- STATUS: gen. nov.
- REFERENCE: Link
- SYNONYMY:   
  ≡ *Cynoglossum* [unranked] *Lappula* (Moench) Wallr. 1921
- BASIS: Original type: *Myosotis lappula* L.

---

*Lappula cynoglossoides*  (Lam.) Gürke in Engler & Prantl, Nat. Pflanzenfam. 4(3a): 107. 1894

- IPNI: Lappula cynoglossoides Gürke -- Nat. Pflanzenfam. [Engler & Prantl] iv. 3a (1893) 107. (IK)
- ASSESSMENT: accepted
- STATUS: comb. nov.
- REFERENCE: Link
- BASIS: Basionym: *Myosotis cynoglossoides*  Lam.
- COMMENTS: Considered the probably correct name of the heterotypic *Cynoglossum echinatum* Thunb..

---

*Lappula patula* (Lehm.) Asch. ex Gürke in Engler & Prantl, Nat. Pflanzenfam. 4(3a): 107. 1894

- IPNI: Lappula patula Asch. ex Gürke -- Nat. Pflanzenfam. [Engler & Prantl] iv. 3a (1803) 107. (IK)
- ASSESSMENT: accepted
- STATUS: comb. nov.
- REFERENCE: Link
- BASIS: Basionym: *Echinospermum patulum*  Lehm.

---

*Lappula squarrosa*  (Retz.) Dumort., Fl. Belg.: 40. 1827

- IPNI: Lappula squarrosa Dumort. -- Fl. Belg. (Dumortier) 40. 1827 (IK)
- ASSESSMENT: accepted
- STATUS: comb. nov.
- REFERENCE: Link
- BASIS: Basionym: *Myosotis squarrosa* Retz.
- COMMENTS: Considered the correct name of the heterotypic synonyms *Cynoglossum lappula* (L.) Scop. (*Cynoglossum clusii* Loisel., nom.illeg.) and *Cynoglossum lusitanicum* Fortis (non L.).

---

*Lepechiniella inconspicua* (Brand) Riedl in Oesterr. Bot. Z. 110: 517. 1963

- IPNI: Lepechiniella inconspicua (Brand) Riedl -- Oesterr. Bot. Z. 110: 517. 1963 (IK)
- ASSESSMENT: accepted
- STATUS: comb. nov.
- REFERENCE: Link
- BASIS: Basionym: *Paracaryum inconspicuum* Brand
- COMMENTS: Considered the correct name of the heterotypic synonyms *Cynoglossum lappula* (L.) Scop. (*Cynoglossum clusii* Loisel., nom.illeg.) and *Cynoglossum lusitanicum* Fortis (non L.).

---

*Lepechiniella microcarpa* (Boiss.) Riedl in Oesterr. Bot. Z. 110: 517. 1963

- IPNI: Lepechiniella microcarpa (Boiss.) Riedl -- Oesterr. Bot. Z. 110: 517. 1963 (IK)
- ASSESSMENT: accepted
- STATUS: comb. nov.
- REFERENCE: Link
- BASIS: Basionym: *Paracaryum microcarpum* Boiss.

---

*Lepechiniella minuta* (Lipsky) Popov in Komarov, Fl. SSSR 19: 402. 1953

- IPNI: Lepechiniella minuta (Lipsky) Popov -- Fl. URSS xix. 402 (Nov. 1953). (IK)
- ASSESSMENT: accepted
- STATUS: comb. nov.
- REFERENCE: Link
- BASIS: Basionym: *Paracaryum minutum* Lipsky

---

*Lepechiniella sarawschanica* (Lipsky) Popov in Komarov, Fl. SSSR 19: 392. 1953 *(‘seravschanica’)*

- IPNI: Lepechiniella seravschanica (Lipsky) Popov -- Fl. URSS xix. 391 (Nov. 1953). (IK)
- ASSESSMENT: accepted
- STATUS: comb. nov.
- REFERENCE: Link
- BASIS: Basionym: *Paracaryum sarawschanicum* Lipsky

---

*Lindelofia* Lehm. in Neue Allg. Deutsche Garten- Blumenzeitung 6: 351. 1850

- IPNI: Lindelofia Lehm. -- in Hamb. Gartenz. vi. (1850) 351. (IK)
- ASSESSMENT: [*Cynoglossum* L.]
- STATUS: gen. nov.
- REFERENCE: Link
- BASIS: Type (Riedl in Rechinger, Fl. Iranica 48: 137. 1967): *Lindelofia spectabilis* Lehm.
- COMMENTS: We agree with Mill (in Edinburgh J. Bot. 67: 143. 2010) in rejecting the previous type designation by Riedl (in Oesterr. Bot. Z. 109: 385. 1962) as ineffective, not being definitely accepted by that author. But there is no valid reason to dismiss the subsequent type designation(s), as done by Mill (l.c.).

---

*Lindelofia* sect. *Anchusophyton* Brand in Engler, Pflanzenr. 78 (IV.252): 82. 1921

- IPNI: absent (2014-11-05)
- ASSESSMENT: [*Cynoglossum* L.]
- STATUS: sect. nov.
- REFERENCE: Link
- BASIS: Original elements: *Lindelofia albida* (Wettst.) Brand, *L. lanata* (L.) Brand, *L. tubiflora* (Murb.) Brand; type not designated

---

*Lindelofia* sect. *Brandia* Popov in Komarov, Fl. SSSR 19: 627. 1953

- IPNI: absent (2014-11-05)
- STATUS: nom. inval. [sine descr. lat., ICN Art. 39.1]
- REFERENCE: Link
- BASIS: Type: see type discussion in Mill (2010) Edinburgh J. Bot. 67 (1): 141—154
- COMMENTS: Popov (l.c.) refers to “*Adelocaryum* Brand … [tantum quoad speciem primam: *A. anchusoides*]”; which means that Brand’s Latin description of *Adelocaryum* cannot be used to validate Popov’s sectional name; and neither can that of *Adelocaryum anchusoides* itself, given in the key (ICN Art. 38.11(b)).

---

*Lindelofia* sect. *Brandia* Popov ex Riedl in Rechinger, Fl. Iranica 48: 137. 1967

- IPNI: absent (2014-11-05)
- ASSESSMENT: [*Cynoglossum* L.]
- STATUS: sect. nov.
- REFERENCE: Link

---

*Lindelofia* ser. *Macrofornicatae* of Popov in Komarov, Fl. SSSR 19: 629. 1953

- IPNI: absent (2014-11-05)
- STATUS: nom. inval. [sine descr. lat., ICN Art. 39.1]
- REFERENCE: Link

---

*Lindelofia* ser. *Microfornicatae* of Popov in Komarov, Fl. SSSR 19: 633. 1953

- IPNI: absent (2014-11-05)
- STATUS: nom. inval. [sine descr. lat., ICN Art. 39.1]
- REFERENCE: Link

---

*Lindelofia* sect. *Pseudocynoglossum* of Popov in Komarov, Fl. SSSR 19: 629. 1953

- IPNI: absent (2014-11-05)
- STATUS: nom. inval. [sine descr. lat., ICN Art. 39.1]
- REFERENCE: Link

---

*Lindelofia* sect. *Trachelanthus* (Kunze) Brand in Engler, Pflanzenr. 78 (IV.252): 80. 1921

- IPNI: absent (2014-11-05)
- ASSESSMENT: [*Cynoglossum* L.]
- STATUS: comb. nov.
- REFERENCE: Link
- BASIS: Basionym: *Trachelanthus* Kunze

---

*Lindelofia albida* (Wettst.) Brand in Engler, Pflanzenr. 78 (IV.252): 83. 1921

- IPNI: Lindelofia albida Brand -- Pflanzenr. (Engler) Borrag.-Cynogloss. 83 (1921). (IK)
- ASSESSMENT: *Cynoglossum albidum* (Wettst.) Greuter & Burdet
- STATUS: comb. nov.
- REFERENCE: Link
- BASIS: Basionym: *Mattia albida*  Wettst.

---

*Lindelofia anchusoides* (Lindl.) Lehm. in Neue Allg. Deutsche Garten- Blumenzeitung 6: 352. 1850

- IPNI: Lindelofia anchusoides (Lindl.) Lehm. -- Neue Allg. Deutsche Garten- Blumenzeitung 6: 352. 1850 (IK)
- ASSESSMENT: *Cynoglossum anchusoides* Lindl.
- STATUS: comb. nov.
- REFERENCE: Link
- BASIS: Basionym: *Cynoglossum anchusoides* Lindl.

---

*Lindelofia anchusoides* subsp. *aspera* (Rech. f.) F. Sadat in Mitt. Bot. Staatssamml. München 28: 104. 1989

- IPNI: Lindelofia anchusoides (Lindl.) Lehm. subsp. aspera (Rech.f.) F.Sadat -- Mitt. Bot. Staatssamml. München 28: 104 (1989):. (IK)
- ASSESSMENT: *Cynoglossum anchusoides* subsp. *asperum* (Rech. f.) Greuter & Stier
- STATUS: comb. & stat. nov. [species to subspecies]
- REFERENCE: Link
- BASIS: Basionym: *Lindelofia aspera* Rech. f.

---

*Lindelofia anchusoides* subsp. *macrostyla* (Bunge) Kamelin in Byull. Moskovsk. Obshch. Isp. Prir., Otd. Biol. 80: 89. 1975

- IPNI: Lindelofia anchusoides (Lindl.) Lehm. subsp. macrostyla (Bunge) Kamelin -- Byull. Mosk. Obshch. Ispyt. Prir., Biol. 80(6): 89. 1975 (IK)
- ASSESSMENT: [*Cynoglossum anchusoides* Lindl.]
- STATUS: comb. & stat. nov. [species to subspecies]
- REFERENCE: Link
- BASIS: Basionym: *Cynoglossum macrostylum* Bunge

---

*Lindelofia angustifolia* (Schrenk) Brand in Engler, Pflanzenr. 78 (IV.252): 87. 1921

- IPNI: Lindelofia angustifolia Brand -- Pflanzenr. (Engler) Borrag.-Cynogloss. 87 (1921). (IK)
- ASSESSMENT: [*Cynoglossum stylosum* Kar. & Kir.]
- STATUS: comb. nov.
- REFERENCE: Link
- BASIS: Basionym: *Solenanthus angustifolius* Schrenk

---

*Lindelofia aspera* Rech. f. in Ann. Naturhist. Mus. Wien 58: 48. 1951

- IPNI: Lindelofia aspera Rech.f. -- in Ann. Naturhist. Mus. Wien lviii. 48 (1951). (IK)
- ASSESSMENT: *Cynoglossum anchusoides* subsp. *asperum* (Rech. f.) Greuter & Stier
- STATUS: spec. nov.
- REFERENCE: Link
- SYNONYMY:   
  ≡ *Lindelofia anchusoides* subsp. *aspera* (Rech. f.) F. Sadat 1989
    
  ≡ *Cynoglossum anchusoides* subsp. *asperum* (Rech. f.) Greuter & Stier 2015
- BASIS: Original material: Afghanistan, Bandar, 6000', along stream, plant 20 inches high, flowers purple-blue, 9. X. 1939. (Koelz no.14107, Typus in hb. Nation. Arbor. Beltsville, fragm. in hb. Mus. Wien) + two paratypes [from protologue]. Holotype: BARC; isotype, W (W1966-0019196, fragm.); paratype: W (W1966-0019191)

---

*Lindelofia benthamii* Hook. f., Fl. Brit. India 4: 159. 1883 *(‘Benthami’)*

- IPNI: Lindelofia benthamii Hook.f. -- Fl. Brit. India [J. D. Hooker] 4(10): 159. 1883 [Jun 1883] (IK)
- ASSESSMENT: [*Cynoglossum stylosum* Kar. & Kir.]
- STATUS: spec. nov.
- REFERENCE: Link
- BASIS: Original material: Solenanthus sp. 3, herb.Ind. Or. H. f. & T. … Kashmir and Western Tibet; alt, 11-15,000 ft., Thomson, Strachey & Winterbottom, & c. [from protologue]. Presumed syntypes: Thomson, GH (GH00097781\*), NY (NY00335505\*)

---

*Lindelofia brachystemon* (Fisch. & C. A. Mey.) Brand in Engler, Pflanzenr. 78 (IV.252): 84. 1921

- IPNI: Lindelofia brachystemon Brand -- Pflanzenr. (Engler) Borrag.-Cynogloss. 84 (1921). (IK)
- ASSESSMENT: *Cynoglossum brachystemon* (Fisch. & C. A. Mey.) Greuter & Stier
- STATUS: comb. nov.
- REFERENCE: Link
- BASIS: Basionym: *Solenanthus brachystemon* Fisch. & C. A. Mey.

---

*Lindelofia campanulata* Riedl in Biol. Skr. 13: 198. 1963

- IPNI: Lindelofia campanulata Riedl -- Biol. Skr. xiii. No. 4 (Symb. Afghan. v.) 198(1963). (IK)
- ASSESSMENT: *Cynoglossum campanulatum* (Riedl) Greuter & Stier
- STATUS: spec. nov.
- REFERENCE: Link
- SYNONYMY:   
  ≡ *Cynoglossum campanulatum* (Riedl) Greuter & Stier 2015
- BASIS: Original material: [Afghanistan], NE: Altin Djelao, bod de rivière (Lindberg 559, Typus W). Ibidem, fruct. (Lindberg 558, W) [+ photograph of type specimen] [from protologue]. Syntypes: W (W1966-0019912, W2004-0015200)

---

*Lindelofia capusii* (Franch.) Popov in Komarov, Fl. SSSR 19: 630. 1953

- IPNI: Lindelofia capusii (Franch.) Popov -- Spisok Rast. Gerb. Fl. S.S.S.R. Bot. Inst. Vsesojuzn. Akad. Nauk 12: 67. 1953, in obs.; et in Komarov, Fl. URSS, 19: 630. 1953. (IK)
- ASSESSMENT: *Cynoglossum capusii* (Franch.) Pazij
- STATUS: comb. nov.
- REFERENCE: Link
- BASIS: Basionym: *Paracaryum capusii* Franch.

---

*Lindelofia cerinthoides* (Boiss.) Brand in Engler, Pflanzenr. 78 (IV.252): 80. 1921

- IPNI: Lindelofia cerinthoides Brand -- Pflanzenr. (Engler) Borrag.-Cynogloss. 80 (1921). (IK)
- ASSESSMENT: *Cynoglossum cerinthoides* (Boiss.) Greuter & Burdet
- STATUS: comb. nov.
- REFERENCE: Link
- BASIS: Basionym: *Solenanthus cerinthoides*  Boiss.

---

*Lindelofia cerinthoides* var. *stenophylla* (Bornm.) Brand in Engler, Pflanzenr. 78 (IV.252): 82. 1921

- IPNI: absent (2014-11-05)
- ASSESSMENT: [*Cynoglossum cerinthoides* (Boiss.) Greuter & Burdet]
- STATUS: comb. nov.
- REFERENCE: Link
- BASIS: Basionym: *Trachelanthus cerinthoides* var. *stenophyllus* Bornm.

---

*Lindelofia cynoglossoides* Brand in Engler, Pflanzenr. 78 (IV.252): 88. 1921

- IPNI: Lindelofia cynoglossoides Brand -- Pflanzenr. (Engler) Borrag.-Cynogloss. 88 (1921). (IK)
- ASSESSMENT: [*Cynoglossum anchusoides* Lindl.]
- STATUS: spec. nov.
- REFERENCE: Link
- BASIS: Original material: Afghanistan (Griffith n. 5978) [from protologue]. Syntype: GH (GH00097782\*)

---

*Lindelofia eriocalycina* (Boiss. & Buhse) Brand in Engler, Pflanzenr. 78 (IV.252): 84. 1921

- IPNI: Lindelofia eriocalycina Brand -- Pflanzenr. (Engler) Borrag.-Cynogloss. 84 (1921). (IK)
- ASSESSMENT: *Cynoglossum eriocalycinum* (Boiss. & Buhse) Greuter & Stier
- STATUS: comb. nov.
- REFERENCE: Link
- BASIS: Basionym: *Solenanthus eriocalycinus* Boiss. & Buhse

---

*Lindelofia hissarica* (Lipsky) Brand in Engler, Pflanzenr. 78 (IV.252): 82. 1921

- IPNI: Lindelofia hissarica Brand -- Pflanzenr. (Engler) Borrag.-Cynogloss. 82 (1921). (IK)
- ASSESSMENT: *Cynoglossum hissaricum* (Lipsky) Greuter & Stier
- STATUS: comb. nov.
- REFERENCE: Link
- BASIS: Basionym: *Trachelanthus hissaricus* Lipsky

---

*Lindelofia kandavanensis* Bornm. & Gauba in Repert. Spec. Nov. Regni Veg. 51: 217. 1942 *(‘Lindelophia’)*

- IPNI: Lindelofia kandavanensis Bornm. & Gauba -- Repert. Spec. Nov. Regni Veg. 51: 217. 1942 (IK)
- ASSESSMENT: *Cynoglossum kandavanense* (Bornm. & Gauba) Akhani
- STATUS: spec. nov.
- REFERENCE: Link
- SYNONYMY:   
  ≡ *Cynoglossum kandavanense* (Bornm. & Gauba) Akhani 1998
- BASIS: Original material: [Iran] Elburs: Nordhänge des Kandavan, im Buschwald, 2300 m (21. VII. 1938; [Gauba] no. 1640) [from protologue]. Holotype: B (B100365354\*)

---

*Lindelofia korolkowii* (Lipsky) Brand in Engler, Pflanzenr. 78 (IV.252): 82. 1921 *(‘korolkowi’)*

- IPNI: Lindelofia korolkowii Brand -- Pflanzenr. (Engler) Borrag.-Cynogloss. 82 (1921). (IK)
- ASSESSMENT: *Cynoglossum korolkowii* (Lipsky) Greuter & Stier
- STATUS: comb. nov.
- REFERENCE: Link
- BASIS: Basionym: *Trachelanthus korolkowii* Lipsky

---

*Lindelofia kurdica* (Kotschy ex Paine) Brand in Engler, Pflanzenr. 78 (IV.252): 82. 1921

- IPNI: Lindelofia kurdica Brand -- Pflanzenr. (Engler) Borrag.-Cynogloss. 82 (1921). (IK)
- ASSESSMENT: [*Cynoglossum cerinthoides* (Boiss.) Greuter & Burdet]
- STATUS: comb. nov.
- REFERENCE: Link
- BASIS: Basionym: *Cerinthopsis kurdica* Kotschy ex Paine

---

*Lindelofia lahulensis* Brand in Repert. Spec. Nov. Regni Veg. 19: 70. 1923

- IPNI: Lindelofia lahulensis Brand -- Repert. Spec. Nov. Regni Veg. 19: 70. 1923 (IK)
- ASSESSMENT: *Pseudomertensia lahulensis* (Brand) Aswal
- STATUS: spec. nov.
- REFERENCE: Link
- SYNONYMY:   
  ≡ *Pseudomertensia lahulensis* (Brand) Aswal 1994
- BASIS: Original material: Südwestlicher Himalaya: Lahul: Kydang, an Felsen des Nyimephed, 4200 bis 4400 m ü. M.. (Heyde, im Juli 1880, Herb. Bernhard Lorenz, Zittau) [from protologue]

---

*Lindelofia lanata* (L.) Brand in Engler, Pflanzenr. 78 (IV.252): 83. 1921

- IPNI: Lindelofia lanata Brand -- Pflanzenr. (Engler) Borrag. Cynogloss. 83. 1921 (IK)
- ASSESSMENT: *Cynoglossum mathezii* Greuter & Burdet
- STATUS: comb. nov.
- REFERENCE: Link
- BASIS: Basionym: *Anchusa lanata* L.

---

*Lindelofia lanata* var. *ovatifolia* Brand in Engler, Pflanzenr. 78 (IV.252): 83. 1921

- IPNI: absent (2014-11-05)
- ASSESSMENT: [*Cynoglossum mathezii* Greuter & Burdet]
- STATUS: var. nov.
- REFERENCE: Link
- BASIS: Original material: [Algeria], Hammam Rbira (Kuegler, Herb. Haussknecht, z.T.) [from protologue]. Holotype: JE (JE00016091\*)

---

*Lindelofia longiflora* (A. DC.) Baill., Hist. Pl. 10: 378. 1890

- IPNI: Lindelofia longiflora Baill. -- Hist. Pl. (Baillon) 10: 379. 1890 [Jul-Aug 1890] ; Gurke in Engl. & Prantl, Naturl. Pflanzenfam. iv.3a (1894) 103 (IK)
- ASSESSMENT: [*Cynoglossum longifolium* (Leichtlin ex Beck & F. Abel) Greuter & Stier]
- STATUS: comb. nov.
- REFERENCE: Link
- BASIS: Basionym: *Omphalodes longiflora*  A. DC.; Replaced synonym: *Cynoglossum longiflorum* Royle ex Benth., non *Cynoglossum longiflorum* Lehm.

---

*Lindelofia longiflora* of Gürke [Engler & Prantl, Nat. Pflanzenfam. 4(3a): 103. 1894]

- IPNI: Lindelofia longiflora Baill. -- Hist. Pl. (Baillon) 10: 379. 1890 [Jul-Aug 1890] ; Gurke in Engl. & Prantl, Naturl. Pflanzenfam. iv.3a (1894) 103 (IK)
- STATUS: [isonym]
- REFERENCE: Link

---

*Lindelofia longiflora* var. *falconeri* (C. B. Clarke) Brand in Engler, Pflanzenr. 78 (IV.252): 85. 1921

- IPNI: absent (2014-11-05)
- ASSESSMENT: [*Cynoglossum longifolium* (Leichtlin ex Beck & F. Abel) Greuter & Stier]
- STATUS: comb. nov.
- REFERENCE: Link
- BASIS: Basionym: *Lindelofia spectabilis* var. *falconeri* C. B. Clarke

---

*Lindelofia longiflora* var. *levingei* (C. B. Clarke) Brand in Engler, Pflanzenr. 78 (IV.252): 85. 1921

- IPNI: absent (2014-11-05)
- ASSESSMENT: [*Cynoglossum longifolium* (Leichtlin ex Beck & F. Abel) Greuter & Stier]
- STATUS: comb. nov.
- REFERENCE: Link
- BASIS: Basionym: *Lindelofia spectabilis* var. *levingei* C. B. Clarke

---

*Lindelofia longifolia* Leichtlin ex Beck & F. Abel in Wiener Ill. Gart.-Zeitung 13: 326. 1888

- IPNI: Lindelofia longifolia Hort. -- ex Wien. Ill. Gart.-Zeit. xiii. (1888) 326. (IK)
- ASSESSMENT: *Cynoglossum longifolium* (Leichtlin ex Beck & F. Abel) Greuter & Stier
- STATUS: spec. nov.
- REFERENCE: Link
- SYNONYMY:   
  ≡ *Cynoglossum longifolium* (Leichtlin ex Beck & F. Abel) Greuter & Stier 2015
- BASIS: Original material: [cultivated plant of unknown origin introduced by Max Leichtlin through his private botanical garden in Baden-Baden, Germany] [inferred from protologue]
- COMMENTS: The name appears in a section of miscellanies (“Miscellen”) under the subtitle “Leichtlin’sche Neuheiten” (novelties from Leichtlin). We accept the names appearing in this portion without authorship as implicitly ascribed to Leichtlin, but the description must be credited to the journal editors, G. Beck von Mannagetta and Friedrich Abel. The name is likely due to a lapsus for *Lindelofia longiflora* (a combination then not yet made), but this cannot be proved. What was presumably the same plant was, in subsequent years, distributed by Leichtlin under yet another synonym, *Lindelofia spectabilis* (see e.g. Garden & Forest 6: 246. 1893; 7: 247. 1894). Even now it is occasionally grown under Leichtlin’s name (see e.g. T. Meyer).

---

*Lindelofia longipedicellata* Riedl in Biol. Skr. 13: 199. 1963

- IPNI: Lindelofia longipedicellata Riedl -- Biol. Skr. xiii. No. 4 (Symb. Afghan. v.) 199 (963). (IK)
- ASSESSMENT: *Cynoglossum longipedicellatum* (Riedl) Greuter & Stier
- STATUS: spec. nov.
- REFERENCE: Link
- SYNONYMY:   
  ≡ *Cynoglossum longipedicellatum* (Riedl) Greuter & Stier 2015
- BASIS: Original material: [Afghanistan], NE: C. Nuristan: Oberes Petsch-(Parun)Tal nördlich Wama, 1400-1800m [+ photograph of type specimen] (Scheibe 146, Typus, W, Isotypus, HAL.) [from protologue]. Syntypes, W (W1966-0020015, W2004-0015201)

---

*Lindelofia macrostyla* (Bunge) Popov in Komarov, Fl. URSS 19: 627. 1953

- IPNI: Lindelofia macrostyla (Bunge) Popov -- Spisok Rast. Gerb. Fl. S.S.S.R. Bot. Inst. Vsesojuzn. Akad. Nauk 12: 67. 1953, in obs.; et in Komarov, Fl. URSS, 19: 627. 1953. (IK)
- ASSESSMENT: [*Cynoglossum anchusoides* Lindl.]
- STATUS: comb. nov.
- REFERENCE: Link
- BASIS: Basionym: *Cynoglossum macrostylum* Bunge

---

*Lindelofia micrantha* Rech. f. & Riedl in Rechinger, Fl. Iranica 48: 139. 1967

- IPNI: Lindelofia micrantha Rech.f. & Riedl -- Fl. Iranica [Rechinger] 48: 139. 1967 (IK)
- ASSESSMENT: *Cynoglossum minutiflorum* Greuter & Stier
- STATUS: spec. nov.
- REFERENCE: Link
- SYNONYMY:   
  ≡ *Cynoglossum minutiflorum* Greuter & Stier 2015
- BASIS: Original material: Typus: RECH. 17378, W. Afghanistan: Ghazni: In monte ad occidentem jugi Sardalu inter Qarabagh et Sang-i Masha; 33°10'N 67°45'E. substr. gyps., 2600 m, 30. VI. 1962, RECH. 17378 [from protologue] Holotype: W (W1966-0019523\*)

---

*Lindelofia olgae* (Regel & Smirn.) Brand in Engler, Pflanzenr. 78 (IV.252): 87. 1921

- IPNI: Lindelofia olgae Brand -- Pflanzenr. (Engler) Borrag. Cynogloss. 87. 1921 (IK)
- ASSESSMENT: *Cynoglossum olgae* (Regel & Smirn.) Greuter & Stier
- STATUS: comb. nov.
- REFERENCE: Link
- BASIS: Basionym: *Solenanthus olgae* Regel & Smirn.

---

*Lindelofia olgae* var. *intermedia* (Lipsky) Brand in Engler, Pflanzenr. 78 (IV.252): 88. 1921

- IPNI: absent (2014-11-05)
- ASSESSMENT: [*Cynoglossum olgae* (Regel & Smirn.) Greuter & Stier]
- STATUS: comb. nov.
- REFERENCE: Link
- BASIS: Basionym: *Solenanthus olgae* var. *intermedius* Lipsky

---

*Lindelofia olgae* var. *tschimganica* (Lipsky) Brand in Engler, Pflanzenr. 78 (IV.252): 88. 1921

- IPNI: absent (2014-11-05)
- ASSESSMENT: [*Cynoglossum olgae* (Regel & Smirn.) Greuter & Stier]
- STATUS: comb. nov.
- REFERENCE: Link
- BASIS: Basionym: *Solenanthus olgae* var. *tschimganicus* Lipsky

---

*Lindelofia platycalyx* Riedl in Biol. Skr. 13: 202. 1963

- IPNI: Lindelofia platycalyx Riedl -- Biol. Skr. xiii. No. 4 (Symb. Afghan. v.) 202 (963). (IK)
- ASSESSMENT: *Cynoglossum platycalyx* (Riedl) Greuter & Stier
- STATUS: spec. nov.
- REFERENCE: Link
- SYNONYMY:   
  ≡ *Cynoglossum platycalyx* (Riedl) Greuter & Stier 2015
- BASIS: Original material: Pakistan: Shadal, Hazara, 16. VIII. 1899 (INAYAT, Typus, W) [+ drawing of type specimen] [from protologue]

---

*Lindelofia pterocarpa* (Rupr.) Popov in Komarov, Fl. SSSR 19: 638. 1953

- IPNI: Lindelofia pterocarpa (Rupr.) Popov -- Spisok Rast. Gerb. Fl. S.S.S.R. Bot. Inst. Vsesojuzn. Akad. Nauk 12: 67. 1953, in obs.; et in Komarov, Fl. URSS, 19: 638. 1953. (IK)
- ASSESSMENT: *Cynoglossum stylosum* subsp. *pterocarpum* (Rupr.) Greuter & Stier
- STATUS: comb. & stat. nov. [variety to species]
- REFERENCE: Link
- BASIS: Basionym: *Solenanthus nigricans* var. *pterocarpus* Rupr.

---

*Lindelofia spectabilis* Lehm. in Neue Allg. Deutsche Garten- Blumenzeitung 6: 352. 1850

- IPNI: Lindelofia spectabilis Lehm. -- in Hamb. Gartenz. vi. (1850) 352. (IK)
- ASSESSMENT: [*Cynoglossum longifolium* (Leichtlin ex Beck & F. Abel) Greuter & Stier]
- STATUS: nom. illeg. [superfl.]
- REFERENCE: Link
- BASIS: Replaced synonyms: *Cynoglossum longiflorum* Royle ex Benth., non *Cynoglossum longiflorum* Lehm.; *Omphalodes longiflora*  A. DC.

---

*Lindelofia spectabilis* var. *falconeri* C. B. Clarke in Hooker, Fl. Brit. India 4: 159. 1883

- IPNI: absent (2014-11-05)
- ASSESSMENT: [*Cynoglossum longifolium* (Leichtlin ex Beck & F. Abel) Greuter & Stier]
- STATUS: var. nov.
- REFERENCE: Link
- SYNONYMY:   
  ≡ *Lindelofia longiflora* var. *falconeri* (C. B. Clarke) Brand 1921
- BASIS: Original material: Kashmir, Falconer [from protologue]

---

*Lindelofia spectabilis* var. *levingei* C. B. Clarke in Hooker, Fl. Brit. India 4: 159. 1883 *(‘levingii’)*

- IPNI: absent (2014-11-05)
- ASSESSMENT: [*Cynoglossum longifolium* (Leichtlin ex Beck & F. Abel) Greuter & Stier]
- STATUS: var. nov.
- REFERENCE: Link
- SYNONYMY:   
  ≡ *Lindelofia longiflora* var. *levingei* (C. B. Clarke) Brand 1921
- BASIS: Original material: Kashmir; Pir Pingul, alt. 11,500 ft., Levinge [from protologue]
- COMMENTS: Correction of the epithet’s original spelling mandated by ICN Art. 60.7.

---

*Lindelofia stenosiphon* Rech. f. in Ann. Naturhist. Mus. Wien 58: 49. 1951

- IPNI: Lindelofia stenosiphon Rech.f. -- in Ann. Naturhist. Mus. Wien lviii. 49 (1951). (IK)
- ASSESSMENT: [*Cynoglossum dielsii* (Bornm.) Greuter & Stier]
- STATUS: spec. nov.
- REFERENCE: Link
- BASIS: Original material: Afghanistan: Kabul. 20. V. 1947 (K. Lindberg, no. 57, Typus in hb. Lindberg, fragm. in hb. Mus. Wien) [from protologue]. Isotype: W (W1966-0019905, fragm.)

---

*Lindelofia stylosa* (Kar. & Kir.) Brand in Engler, Pflanzenr. 78 (IV.252): 85. 1921

- IPNI: Lindelofia stylosa (Kar. & Kir.) Brand -- Pflanzenr. (Engler) Borrag. Cynogloss. 85. 1921 (IK)
- ASSESSMENT: *Cynoglossum stylosum* Kar. & Kir.
- STATUS: comb. nov.
- REFERENCE: Link
- BASIS: Basionym: *Cynoglossum stylosum* Kar. & Kir.

---

*Lindelofia stylosa* var. *hispida* (Regel) Brand in Engler, Pflanzenr. 78 (IV.252): 87. 1921

- IPNI: absent (2014-11-05)
- ASSESSMENT: [*Cynoglossum stylosum* Kar. & Kir.]
- STATUS: comb. nov.
- REFERENCE: Link
- BASIS: Basionym: *Solenanthus nigricans* var. *hispidus* Regel

---

*Lindelofia stylosa* var. *pterocarpa* (Rupr.) M. Pop. ex Czuk. in Flora Tadsch. SSR 7: 489. 1984

- IPNI: absent (2014-11-05)
- ASSESSMENT: *Cynoglossum stylosum* subsp. *pterocarpum* (Rupr.) Greuter & Stier
- STATUS: comb. nov.
- REFERENCE: Link
- BASIS: Basionym: *Solenanthus nigricans* var. *pterocarpus* Rupr.

---

*Lindelofia stylosa* subsp. *pterocarpa* (Rupr.) Kamelin in Novon 3: 263. 1993

- IPNI: Lindelofia stylosa (Kar. & Kir.) Brand subsp. pterocarpa (Rupr.) Kamelin -- Novon 3(3): 263. 1993 (IK)
- ASSESSMENT: *Cynoglossum stylosum* subsp. *pterocarpum* (Rupr.) Greuter & Stier
- STATUS: stat. nov. [variety to subspecies]
- REFERENCE: Link
- BASIS: Basionym: *Solenanthus nigricans* var. *pterocarpus* Rupr.

---

*Lindelofia tschimganica* (Lipsky) Popov in Bot. Mater. Gerb. Inst. Bot. Akad. Nauk Uzbeksk. S.S.R. 16: 43. 1961

- IPNI: Lindelofia tschimganica (Lipsky) Popov in Pazij -- in Not. Syst. Herb. Inst. Bot. Acad. Sci. Uzbekistan. xvi. 43 (1961). (IK)
- ASSESSMENT: [*Cynoglossum olgae* (Regel & Smirn.) Greuter & Stier]
- STATUS: comb. & stat. nov. [variety to species]
- REFERENCE: Link
- BASIS: Basionym: *Solenanthus olgae* var. *tschimganicus* Lipsky

---

*Lindelofia tubiflora* (Murb.) Brand in Engler, Pflanzenr. 78 (IV.252): 83. 1921

- IPNI: Lindelofia tubiflora Brand -- Pflanzenr. (Engler) Borrag. Cynogloss. 83. 1921 (IK)
- ASSESSMENT: *Cynoglossum tubiflorum* (Murb.) Greuter & Burdet
- STATUS: comb. nov.
- REFERENCE: Link
- BASIS: Basionym: *Solenanthus tubiflorus* Murb.

---

*Lithospermum flexuosum* Lehm., Pl. Asperif. Nucif.: 333. 1818

- IPNI: Lithospermum flexuosum Lehm. -- Pl. Asperif. Nucif. 2: 333. 1818 [Sep-Oct 1818] (IK)
- ASSESSMENT: accepted
- STATUS: nom. nov.
- REFERENCE: Link
- BASIS: Replaced synonym: *Cynoglossum muricatum* Thunb., non *Lithospermum muricatum* Ruiz & Pav. (1799)

---

*Lobostemon glaucophyllus* (Jacq.) Buek in Linnaea 11: 138. 1837

- IPNI: Lobostemon glaucophyllus Buek. -- Linnaea 11: 138. 1837 (IK)
- ASSESSMENT: accepted
- STATUS: comb. nov.
- REFERENCE: Link
- BASIS: Basionym: *Echium glaucophyllum* Jacq.
- COMMENTS: Considered the correct name of the heterotypic *Cynoglossum papillosum* (Thunb.) Thunb. (*Echium papillosum* Thunb.).

---

*Mattia* Schult., Observ. Bot.: 30,32. 1809

- IPNI: Mattia Schult. -- Observ. Bot. [Schultes] 30, 32 (1809). (IK)
- ASSESSMENT: [*Cynoglossum* L.]
- STATUS: nom. illeg. [superfl.]
- REFERENCE: Link
- BASIS: Replaced synonym: *Rindera* Pall.

---

*Mattia* Roem. & Schult., Syst. Veg. 4: 82. 1819

- IPNI: absent (2014-11-05)
- ASSESSMENT: [*Cynoglossum* L.]
- STATUS: nom. illeg. [homonym, ICN Art. 48.1]
- REFERENCE: Link
- BASIS: Three named species are included, *Mattia umbellata*, *Mattia lanata*, and *Mattia staminea*. The former should logically be regarded as the type, but has not yet been formally designated as such
- COMMENTS: The automatic type (ICN Art. 7.5) of *Rindera* Schult., *Rindera tetraspis*, is excluded by implication, being treated as a synonym of *Rindera laevigata* in *Rindera*, a separate genus (ICN Art. 48.1).

---

*Mattia* subg. *Mattiaria* Coss. in Bull. Soc. Bot. France 3: 709. 1857

- IPNI: absent (2014-11-05)
- ASSESSMENT: [*Cynoglossum* L.]
- STATUS: subg. nov.
- REFERENCE: Link
- SYNONYMY:   
  ≡ *Rindera* sect. *Mattiaria* (Coss.) Kusn. 1910
    
  ≡ *Cynoglossum* subg. *Mattiaria* (Coss.) Greuter 1981
- BASIS: Original type: *Mattia gymnandra* Coss.

---

*Mattia alapadnochiton* Vatke in Z. Gesammten Naturwiss. (Halle) 45: 126. 1875

- IPNI: Mattia alapadnochiton Vatke -- Zeitschr. Naturwiss. xlv. 126 (1875); Bornmuller in Beih. Bot. Centralbl. xxxiii. II. 176. (IK)
- ASSESSMENT: [*Cynoglossum lanatum* Lam.]
- STATUS: spec. nov.
- REFERENCE: Link
- BASIS: Original material: [Iran], Ad nives Kuh Nur Persiae australis julio 1868 legit cl. professor C. Haussknecht [from protologue]. Syntype: JE (JE00011965\*)

---

*Mattia albida*  Wettst. in Denkschr. Kaiserl. Akad. Wiss., Wien, Math.-Naturwiss. Kl. 50: 32. 1885

- IPNI: Mattia albida Wettst. ex Stapf -- Denkschr. Kaiserl. Akad. Wiss., Wien. Math.-Naturwiss. Kl. 50(2): 32. 1885 (IK)
- ASSESSMENT: *Cynoglossum albidum* (Wettst.) Greuter & Burdet
- STATUS: spec. nov.
- REFERENCE: Link
- SYNONYMY:   
  ≡ *Rindera albida* (Wettst.) Kusn. 1910
    
  ≡ *Lindelofia albida* (Wettst.) Brand 1921
    
  ≡ *Cynoglossum albidum* (Wettst.) Greuter & Burdet 1981
- BASIS: Original material: [Iran], In montibus Karaghan prope Schurab (11.V.) [Polak] [from protologue] Presumed holotype: WU (WU0072783); Isotype: US (US00664122\*)

---

*Mattia angustifolia* (Willd.) G. Don, Gen. Hist. 4: 310. 1837–1838

- IPNI: Mattia angustifolia G.Don -- Gen. Hist. iv. 310. (IK)
- ASSESSMENT: [*Cynoglossum racemosum* Schreb.]
- STATUS: comb. nov.
- REFERENCE: Link
- BASIS: Basionym: *Cynoglossum angustifolium* Willd.

---

*Mattia aucheri* A. DC. in Candolle, Prodr. 10: 169. 1846

- IPNI: Mattia aucheri DC. & A.DC. -- Prodr. [A. P. de Candolle] 10: 169. 1846 [8 Apr 1846] (IK)
- ASSESSMENT: *Cynoglossum aucheri* (A. DC.) Greuter & Burdet
- STATUS: spec. nov.
- REFERENCE: Link
- SYNONYMY:   
  ≡ *Paracaryum aucheri* (A. DC.) Boiss. 1849
    
  ≡ *Mattiastrum aucheri* (A. DC.) Brand 1915
    
  ≡ *Cynoglossum aucheri* (A. DC.) Greuter & Burdet 1981
- BASIS: Original material: in Asia Minori circa Moglah legit cl. Aucher! Cynoglossum lanatum Auch.! pl. exs. N. 908 et 2285. (v. s. a cl. inv.) [from protologue]. Syntypes: *Aucher-Eloy 908*, G-DC (G00202105\*); *Aucher-Eloy 2285* (G00202108\*); isosyntype: MPU (MPU013011\*)

---

*Mattia brachyantha* Boiss., Diagn. Pl. Orient. 11: 127. 1849

- IPNI: Mattia brachyantha Boiss. -- Diagn. Pl. Orient. ser. 1, 11: 127. 1849 [Mar-Apr 1849] (IK)
- ASSESSMENT: [*Cynoglossum lanatum* Lam.]
- STATUS: spec. nov.
- REFERENCE: Link
- SYNONYMY:   
  ≡ *Cyphomattia lanata* var. *brachyantha* (Boiss.) Boiss. 1875
    
  ≡ *Mattia lanata* var. *brachyantha* (Boiss.) Bornm. 1906
- BASIS: Original material: In Armeniâ? vel Persiâ? *Aucher* No 4976 [from protologue]. Syntype: P (P04083559)

---

*Mattia bracteata* of Orphanides [Boiss., Fl. Orient. 4: 274. 1875]

- IPNI: Mattia bracteata Orph. ex Boiss. -- Fl. Orient. [Boissier] 4(1): 274. 1875 [Sep-Oct 1875] (IK)
- STATUS: nom. inval. [pro syn. sub *Mattia graeca*]
- REFERENCE: Link
- COMMENTS: Later validated as *Rindera graeca* var. *bracteata* Boiss. & Orph. ex Kusn..

---

*Mattia bungei* Boiss., Fl. Orient. 4: 274. 1875

- IPNI: Mattia bungei Boiss. -- Fl. Orient. [Boissier] 4(1): 274. 1875 [Sep-Oct 1875] (IK)
- ASSESSMENT: *Cynoglossum bungei* (Boiss.) Greuter & Stier
- STATUS: spec. nov.
- REFERENCE: Link
- SYNONYMY:   
  ≡ *Rindera bungei* (Boiss.) Gürke 1894
    
  ≡ *Bilegnum bungei* (Boiss.) Brand 1915
    
  ≡ *Cynoglossum bungei* (Boiss.) Greuter & Stier 2015
- BASIS: Original material: in montibus Persiae bor.-orientalis prope Schahrud (Bge.) [from protologue]. Holotype: G-BOIS
- COMMENTS: Not to be confused with *Echinospermum bungei* Boiss. ≡ *Paracaryum bungei* (Boiss.) Brand, based on a different Bunge gathering from the same area.

---

*Mattia caespitosa* A. DC. in Candolle, Prodr. 10: 168. 1846

- IPNI: Mattia caespitosa A.DC. -- Prodr. [A. P. de Candolle] 10: 168. 1846 [8 Apr 1846] (IK)
- ASSESSMENT: *Cynoglossum caespitosum* (A. DC.) Greuter & Burdet
- STATUS: spec. nov.
- REFERENCE: Link
- SYNONYMY:   
  ≡ *Rindera caespitosa* (A. DC.) Gürke 1894
    
  ≡ *Cynoglossum caespitosum* (A. DC.) Greuter & Burdet 1981
- BASIS: Original material: in Cappadocia ad Euphratem legit cl. Aucher! *Cynoglossum umbellatum* Auch.! pl. exs. n. 2280 et 2282 [from protologue]. Syntypes: *Aucher-Eloy 2280*, G-DC (G00202113), *Aucher-Eloy 2280*, G-DC (G00202112), MPU (MPU013007)

---

*Mattia canescens* A. DC. in Candolle, Prodr. 10: 168. 1846

- IPNI: Mattia canescens A.DC. -- Prodr. [A. P. de Candolle] 10: 168. 1846 [8 Apr 1846] (IK)
- ASSESSMENT: [*Cynoglossum lanatum* Lam.]
- STATUS: spec. nov.
- REFERENCE: Link
- SYNONYMY:   
  ≡ *Rindera lanata* var. *canescens* (A. DC.) Kusn. 1849
    
  ≡ *Rindera canescens* (A. DC.) Bunge 1851
    
  ≡ *Mattia lanata* var. *canescens* (A. DC.) Kusn. ex. Bornm. 1937
- BASIS: Original material: in Oriente (Michx.) inter Kermancha et Amadan (Oliv. et Brug). *Rindera tetraspis* Lher. herb. [from protologue]. Syntypes: *Michaux*, C-DC (G00202115\*), *Olivier & Bruguière*, G-DC (G00202114\*), P (P04083550\*)

---

*Mattia columnae* (Ten.) G. Don, Gen. Hist. 4: 310. 1837–1838

- IPNI: Mattia columnae G.Don -- Gen. Hist. 4: 310. [1837-1838] (IK)
- ASSESSMENT: *Cynoglossum columnae* Ten.
- STATUS: comb. nov.
- REFERENCE: Link
- BASIS: Basionym: *Cynoglossum columnae* Ten.

---

*Mattia corymbiformis* DC. & A. DC. in Candolle, Prodr. 10: 169. 1846

- IPNI: Mattia corymbiformis DC. & A.DC. -- Prodr. [A. P. de Candolle] 10: 169. 1846 [8 Apr 1846] (IK)
- ASSESSMENT: *Cynoglossum corymbiforme* (DC. & A. DC.) Greuter & Burdet
- STATUS: spec. nov.
- REFERENCE: Link
- SYNONYMY:   
  ≡ *Paracaryum corymbiforme* (DC. & A. DC.) Boiss. 1849
    
  ≡ *Mattiastrum corymbiforme* (DC. & A. DC.) Brand 1915
    
  ≡ *Cynoglossum corymbiforme* (DC. & A. DC.) Greuter & Burdet 1981
- BASIS: Original material: in Armeniâ. Cynogl. glastifolium Auch. pl. exs. n. 2292 (v. s. a cl. inv.) [from protologue]. Holotype: G-DC (G00202110\*); isotypes: G(G00236144\*, G00236145\*), GH (GH00097549\*), MPU (MPU013008\*)

---

*Mattia corymbiformis* var. *polystachya* DC. & A. DC. in Candolle, Prodr. 10: 169. 1846

- IPNI: absent (2014-11-05)
- ASSESSMENT: [*Cynoglossum corymbiforme* (DC. & A. DC.) Greuter & Burdet]
- STATUS: var. nov.
- REFERENCE: Link
- SYNONYMY:   
  ≡ *Paracaryum corymbiforme* var. *polystachyum* (DC. & A. DC.) Tchich. 1860
- BASIS: Original material: In Armeniâ etiam legit cl. Aucher pl. exs. 2329. (v. s. a cl. inv.) [from protologue]. Holotype: G-DC (G00202109\*)

---

*Mattia cristata* (Schreb.) G. Don, Gen. Hist. 4: 310. 1837–1838

- IPNI: Mattia cristata G.Don -- Gen. Hist. iv. 310. (IK)
- ASSESSMENT: *Cynoglossum cristatum* Schreb.
- STATUS: comb. nov.
- REFERENCE: Link
- BASIS: Basionym: *Cynoglossum cristatum* Schreb.

---

*Mattia emarginata* (Lam.) Roem. & Schult., Syst. Veg. 4: 83, 765. 1819

- IPNI: Mattia emarginata Roem. & Schult. -- Syst. Veg., ed. 15 bis [Roemer & Schultes] 4: 83, 765. 1819 (IK)
- ASSESSMENT: [*Cynoglossum racemosum* Schreb.]
- STATUS: comb. nov.
- REFERENCE: Link
- BASIS: Basionym: *Cynoglossum emarginatum* Lam.

---

*Mattia eriantha* K. Koch ex Ledeb., Fl. Ross. 3: 173. 1847

- IPNI: Mattia eriantha Ledeb. -- Fl. Ross. (Ledeb.) 3(1,8): 173. 1847 [Oct 1847] (IK)
- ASSESSMENT: *Cynoglossum regium* (S. G. Gmel.) Greuter & Stier
- STATUS: nom. illeg. [superfl.]
- REFERENCE: Link
- BASIS: Replaced synonym: *Symphytum regium*  S. G. Gmel.

---

*Mattia glastifolia* (Willd.) G. Don, Gen. Hist. 4: 310. 1837–1838

- IPNI: Mattia glastifolia G.Don -- Gen. Hist. iv. 310. (IK)
- ASSESSMENT: *Cynoglossum glastifolium* Willd.
- STATUS: comb. nov.
- REFERENCE: Link
- BASIS: Basionym: *Cynoglossum glastifolium* Willd.

---

*Mattia graeca* A. DC. in Candolle, Prodr. 10: 168. 1846

- IPNI: Mattia graeca A.DC. -- Prodr. [A. P. de Candolle] 10: 168. 1846 [8 Apr 1846] (IK)
- ASSESSMENT: *Cynoglossum graecum* (A. DC.) Greuter & Burdet
- STATUS: spec. nov.
- REFERENCE: Link
- SYNONYMY:   
  ≡ *Rindera graeca* (A. DC.) Boiss. & Heldr. 1846
    
  ≡ *Cynoglossum graecum* (A. DC.) Greuter & Burdet 1981
- BASIS: Original material: in cacumine 6000 ped. alto montis Kronion Graeciae (Heldr.!). Rindera Graeca Boiss. et Heldr.! pl. exs. anno 1844 [from protologue]. Holotype: G-DC (G00202111\*)

---

*Mattia gymnandra* Coss. in Bull. Soc. Bot. France 3: 708. 1857

- IPNI: Mattia gymnandra Coss. -- Bull. Soc. Bot. France 3: 708. 1857 [1856 publ. 1857] (IK)
- ASSESSMENT: *Cynoglossum gymnandrum* (Coss.) Greuter & Burdet
- STATUS: spec. nov.
- REFERENCE: Link
- SYNONYMY:   
  ≡ *Rindera gymnandra* (Coss.) Gürke 1894
    
  ≡ *Cynoglossum gymnandrum* (Coss.) Greuter & Burdet 1981
- BASIS: Original material: Coss. ap. Bourgeau *pl. Alger. exsicc.* n. 24 c (1856); in provincia Algeriensi: in montium Djurdjura occidentalium verticibus *Tizi Tsennent et Tamegout*, circiter a 1600 metris, … comitante amicissimo H. de la Perraudière inventa [from protologue]

---

*Mattia himalayensis* Klotzsch in Klotzsch & Garcke, Bot. Ergebn. Reise Waldemar: 94. 1862

- IPNI: Mattia himalayensis Klotzsch -- Bot. Ergebn. Reise Waldemar [Klotzsch & Garcke] 94. 1862 [Jan 1862] (IK)
- ASSESSMENT: *Cynoglossum himalayense* (Klotzsch) Greuter & Stier
- STATUS: spec. nov.
- REFERENCE: Link
- SYNONYMY:   
  ≡ *Paracaryum himalayense* (Klotzsch) C. B. Clarke 1885
    
  ≡ *Mattiastrum himalayense*  (Klotzsch) Brand 1915
    
  ≡ *Cynoglossum himalayense* (Klotzsch) Greuter & Stier 2015
- BASIS: Original material: Tafel 64; von Dr. Hoffmeister im Himalaya entdeckt [from protologue]. Original illustration:  Klotzsch & Garcke, Bot. Ergebn. Reise Waldemar: t. 64

---

*Mattia incana* Ledeb., Fl. Ross. 3: 173. 1847

- IPNI: Mattia incana Ledeb. -- Fl. RoM. iii. 173. (IK)
- ASSESSMENT: *Cynoglossum incanum* (Ledeb.) Greuter & Burdet
- STATUS: spec. nov.
- REFERENCE: Link
- SYNONYMY:   
  ≡ *Paracaryum incanum* (Ledeb.) Boiss. 1875
    
  ≡ *Mattiastrum incanum* (Ledeb.) Brand 1915
    
  ≡ *Cynoglossum incanum* (Ledeb.) Greuter & Burdet 1981
- BASIS: Original material: In provinciis caucasicis versus fines turcicas (Nordm. pl. exs.) [from protologue]

---

*Mattia laevigata* of Schultes, Observ. Bot.: 31. 1809

- IPNI: Mattia laevigata Schult. -- Obs. But. 81. (IK)
- STATUS: no name [ICN Art. 35.2]
- REFERENCE: Link

---

*Mattia lanata* of Schultes, Observ. Bot.: 31. 1809

- IPNI: Mattia lanata Schult. -- Observ. Bot. [Schultes] 31. 1809 (IK)
- STATUS: no name [ICN Art. 35.2]
- REFERENCE: Link

---

*Mattia lanata* (Lam.) Roem. & Schult., Syst. Veg. 4: 82. 1819

- IPNI: absent (2014-11-05)
- ASSESSMENT: *Cynoglossum lanatum* Lam.
- STATUS: comb. nov.
- REFERENCE: Link
- BASIS: Basionym: *Cynoglossum lanatum* Lam.

---

*Mattia lanata* var. *brachyantha* (Boiss.) Bornm. in Beih. Bot. Centralbl., Abt. 2, 20: 194. 1906

- IPNI: absent (2014-11-05)
- ASSESSMENT: [*Cynoglossum lanatum* Lam.]
- STATUS: comb. nov.
- REFERENCE: Link
- BASIS: Basionym: *Mattia brachyantha* Boiss.

---

*Mattia lanata* var. *canescens* (A. DC.) Kusn. ex. Bornm. in Repert. Spec. Nov. Regni Veg. 41: 327. 1937

- IPNI: absent (2014-11-05)
- ASSESSMENT: [*Cynoglossum lanatum* Lam.]
- STATUS: comb. nov.
- REFERENCE: Link
- BASIS: Basionym: *Mattia canescens* A. DC.

---

*Mattia lanata* var. *cyanoptera* Bornm. in Beih. Bot. Centralbl., Abt. 2, 20: 194. 1906

- IPNI: absent (2014-11-05)
- ASSESSMENT: [*Cynoglossum lanatum* Lam.]
- STATUS: var. nov.
- REFERENCE: Link
- SYNONYMY:   
  ≡ *Rindera lanata* f. *cyanoptera* (Bornm.) Brand 1921
- BASIS: Original material: [Turkey] extra fines Persiae: Cappadociae ad radices montis Argaei (Bornm. pl. exsicc. Anatoliae orient. no. 2829) [from protologue]. Syntypes: B (B100365435\*, B100365436\*), JE (JE00011961\*), PH (PH00017093\*)

---

*Mattia lanata* var. *detonsa* Bornm. in Beih. Bot. Centralbl., Abt. 2, 20: 194. 1906

- IPNI: absent (2014-11-05)
- ASSESSMENT: [*Cynoglossum lanatum* Lam.]
- STATUS: var. nov.
- REFERENCE: Link
- SYNONYMY:   
  ≡ *Rindera lanata* var. *detonsa* (Bornm.) Bornm. 1911
    
  ≡ *Rindera lanata* subvar. *detonsa* (Bornm.) Brand 1921
- BASIS: Original material: Inter Hamadan et Tebris in monte Takhti-Soleiman; VI. 1898, [Strauss] [from protologue]. Syntypes: B (B100365434\*), JE (JE00011960\*)

---

*Mattia lanata* var. *euryptera* Bornm. in Beih. Bot. Centralbl., Abt. 2, 20: 194. 1906

- IPNI: absent (2014-11-05)
- ASSESSMENT: [*Cynoglossum lanatum* Lam.]
- STATUS: var. nov.
- REFERENCE: Link
- SYNONYMY:   
  ≡ *Rindera lanata* f. *euryptera* (Bornm.) Brand 1921
- BASIS: Original material: Sintenis exsicc. (no. 2357; prope Egin. VI. 1890 lect.). Extra fines Persiae: Kurdistania, Egin [from protologue]. Syntypes: JE (JE, JE00011964\*), P (P00622865\*, P00622866\*)
- COMMENTS: Bornmüller seemingly ascribes the name to Haussknecht, but at the end adds: “pro specie”, therefore crediting Haussknecht with the species name (never published), not with the varietal name.

---

*Mattia lanata* var. *macrophylla* Bornm. in Beih. Bot. Centralbl., Abt. 2, 20: 194. 1906

- IPNI: absent (2014-11-05)
- ASSESSMENT: [*Cynoglossum lanatum* Lam.]
- STATUS: var. nov.
- REFERENCE: Link
- SYNONYMY:   
  ≡ *Rindera lanata* f. *macrophylla* (Bornm.) Brand 1921
- BASIS: Original material: Chomeïn, in motosis; VII. 1896, [*Strauss*] [from protologue]. Syntypes: B (B100365431\*), JE (JE00011962\*, JE00011963\*)
- COMMENTS: Bornmüller seemingly ascribes the name to Haussknecht, but then adds: (spec.), therefore crediting Haussknecht with the species name (never published), not with the varietal name.

---

*Mattia lanata* var. *punctata* (A. DC.) Bornm. in Beih. Bot. Centralbl., Abt. 2, 20: 194. 1906

- IPNI: absent (2014-11-05)
- ASSESSMENT: [*Cynoglossum tetraspis* (Pall.) Greuter & Burdet]
- STATUS: comb. & stat. nov. [species to variety]
- REFERENCE: Link
- BASIS: Basionym: *Mattia punctata* A. DC.

---

*Mattia lanata* var. *stenophylla* Bornm. in Beih. Bot. Centralbl., Abt. 2, 20: 194. 1906

- IPNI: absent (2014-11-05)
- ASSESSMENT: [*Cynoglossum lanatum* Lam.]
- STATUS: var. nov.
- REFERENCE: Link
- SYNONYMY:   
  ≡ *Rindera lanata* var. *stenophylla* (Bornm.) Bornm. 1911
- BASIS: Original material: Sultanabad, in montosis, VI. 1890. – In monte Raswend, prope Asna; 4. VI. 1892 et VI. 1897; – In monte Schahsinde; VI. 1897. Prope Chomeïn; VI. 1896. In monte Schuturun-kuh; 1899. Hamadan, in latere meridionali alpium Elwend; VI. 1902 et 16. VI. 1895, [*Strauss*] [from protologue]. Syntype: Hamadan, 6.1902, B (B100250760\*)

---

*Mattia lateriflora* (Lam.) G. Don, Gen. Hist. 4: 310. 1837–1838 *(‘lateriflorum’)*

- IPNI: Mattia lateriflorum G.Don -- Gen. Hist. iv. 310. (IK)
- ASSESSMENT: *Pectocarya lateriflora*  (Lam.) A. DC.
- STATUS: comb. nov.
- REFERENCE: Link
- BASIS: Basionym: *Cynoglossum lateriflorum* Lam.

---

*Mattia leptophylla* A. DC. in Candolle, Prodr. 10: 170. 1846

- IPNI: Mattia leptophylla DC. & A.DC. -- Prodr. [A. P. de Candolle] 10: 170. 1846 [8 Apr 1846] (IK)
- ASSESSMENT: *Cynoglossum leptophyllum* (A. DC.) Greuter & Burdet
- STATUS: spec. nov.
- REFERENCE: Link
- SYNONYMY:   
  ≡ *Paracaryum leptophyllum* (A. DC.) Boiss. 1849
    
  ≡ *Mattiastrum leptophyllum* (A. DC.) Brand 1915
    
  ≡ *Cynoglossum leptophyllum* (A. DC.) Greuter & Burdet 1981
- BASIS: Original material: in Armenia. *Cynogl. angustifolium* Auch. pl. exs. n. 2382 et 2281 (v. s. a cl. inv.) [from protologue]. Syntypes: *Aucher-Eloy* 2382, G-DC (G00202101\*); *Aucher-Eloy* 2281, G-DC (G00202102), isosyntypes: BM (BM001014423\*), E (E00284806\*), G (G00236072\*, G00236073\*), MPU (MPU013009\*)

---

*Mattia magellensis* (Ten.) G. Don, Gen. Hist. 4: 310. 1837–1838 *(‘magellense’)*

- IPNI: Mattia magellensis G.Don -- Gen. Hist. iv. 310. (IK)
- ASSESSMENT: *Cynoglossum magellense*  Ten.
- STATUS: comb. nov.
- REFERENCE: Link
- BASIS: Basionym: *Cynoglossum magellense*  Ten.

---

*Mattia oblongifolia* Popov ex Baranov in Izv. Turkestansk. Otd. Russk. Geogr. Obshch. 17: 20, 33. 1925

- IPNI: Mattia oblongifolia Popov ex Baranov -- J. Turkest. Branch Russ. Geogr. Soc. 1924, xvii. 20, 33 (1925), nomen. (IK)
- STATUS: nom. inval. [nom. nud.]
- REFERENCE: Link

---

*Mattia pilosa* (Ruiz & Pav.) G. Don, Gen. Hist. 4: 310. 1837–1838

- IPNI: Mattia pilosa G.Don -- Gen. Hist. iv. 310. (IK)
- ASSESSMENT: [*Pectocarya lateriflora*  (Lam.) A. DC.]
- STATUS: comb. nov.
- REFERENCE: Link
- BASIS: Basionym: *Cynoglossum pilosum* Ruiz & Pav.

---

*Mattia punctata* A. DC. in Candolle, Prodr. 10: 167. 1846

- IPNI: Mattia punctata A.DC. -- Prodr. [A. P. de Candolle] 10: 167. 1846 [8 Apr 1846] (IK)
- ASSESSMENT: [*Cynoglossum tetraspis* (Pall.) Greuter & Burdet]
- STATUS: spec. nov.
- REFERENCE: Link
- SYNONYMY:   
  ≡ *Mattia lanata* var. *punctata* (A. DC.) Bornm. 1906
    
  ≡ *Rindera lanata* var. *punctata* (A. DC.) Kusn. 1910
    
  ≡ *Rindera lanata* subvar. *punctata* (A. DC.) Brand 1921
- BASIS: Original material: in Oriente ad Antab, in monte Tauro, et in Persiae montibus excelsis legit cl. Aucher. *Cynoglossum laevigatum* Auch. n. 1921 et 2291. (v. s.) “Cynoglossum laevigatum” Auch. [from protologue]. Syntypes: *Aucher-Eloy 1921*, G-DC (G00202116\*); *Aucher-Eloy 2291*, G-DC (G00202117\*), isosyntypes: MPU (MPU013010\*), P (P04083635\*)

---

*Mattia schlumbergeri* Boiss., Fl. Orient. 4: 274. 1875

- IPNI: Mattia schlumbergeri Boiss. -- Fl. Orient. [Boissier] 4(1): 274. 1875 [Sep-Oct 1875] (IK)
- ASSESSMENT: *Cynoglossum schlumbergeri* (Boiss.) Greuter & Burdet
- STATUS: spec. nov.
- REFERENCE: Link
- SYNONYMY:   
  ≡ *Rindera schlumbergeri* (Boiss.) Gürke 1894
    
  ≡ *Cynoglossum schlumbergeri* (Boiss.) Greuter & Burdet 1981
- BASIS: Original material: in Libano ubi cl. Schlumberger legit vere 1872 [from protologue]

---

*Mattia schmidtii* Heldr. in Ann. Sci. Nat., Bot., ser. 4, 13: 382. 1860

- IPNI: Mattia schmidtii Heldr. -- Ann. Sci. Nat., Bot. sér. 4, 13: 382. 1860 (IK)
- ASSESSMENT: [*Cynoglossum graecum* (A. DC.) Greuter & Burdet]
- STATUS: spec. nov.
- REFERENCE: Link
- BASIS: Original material: [Greece], in rupestribus calcareis reg. superioris montis Dirphyis Eubœæ (m. Delphi hod.) alt. 3780 ped. supra mare, ubi floriferam legit cl. 20 Maii 1860 cl. et am. J.-F. Jul. Schmidt [from protologue]

---

*Mattia staminea* (Desf.) Roem. & Schult., Syst. Veg. 4: 82, 764. 1819

- IPNI: Mattia staminea Roem. & Schult. -- Syst. Veg., ed. 15 bis [Roemer & Schultes] 4: 82, 764. 1819 (IK)
- ASSESSMENT: *Cynoglossum stamineum* Desf.
- STATUS: comb. nov.
- REFERENCE: Link
- BASIS: Basionym: *Cynoglossum stamineum* Desf.

---

*Mattia umbellata* (Waldst. & Kit.) Schult., Observ. Bot.: 32. 1809

- IPNI: Mattia umbellata Schult. -- Observ. Bot. [Schultes] 32. 1809 (IK)
- ASSESSMENT: *Cynoglossum umbellatum* Waldst. & Kit.
- STATUS: comb. nov.
- REFERENCE: Link
- BASIS: Basionym: *Cynoglossum umbellatum* Waldst. & Kit.

---

*Mattia umbellata* of K. Koch in Linnaea 17: 302. 1844

- IPNI: Mattia umbellata K.Koch -- Linnaea 17: 302. 1844 (IK)
- STATUS: [isonym]
- REFERENCE: Link

---

*Mattiastrum* (Boiss.) Brand in Repert. Spec. Nov. Regni Veg. 14: 150. 1915

- IPNI: Mattiastrum Brand -- Repert. Spec. Nov. Regni Veg. 14: 150. 1915 (IK)
- ASSESSMENT: [*Cynoglossum* L.]
- STATUS: comb. & stat. nov. [section to genus]
- REFERENCE: Link
- BASIS: Basionym: *Paracaryum* sect. *Mattiastrum* Boiss.

---

*Mattiastrum* subsect. *Annua* Riedl in Rechinger, Fl. Iranica 48: 121. 1967

- IPNI: absent (2014-11-05)
- ASSESSMENT: [*Cynoglossum* L.]
- STATUS: subsect. nov.
- REFERENCE: Link
- BASIS: Original type: *Mattiastrum tibeticum* (C. B. Clarke) Brand

---

*Mattiastrum* subsect. *Caespitosa* Riedl in Rechinger, Fl. Iranica 48: 117. 1967

- IPNI: absent (2014-11-05)
- ASSESSMENT: [*Cynoglossum* L.]
- STATUS: subsect. nov.
- REFERENCE: Link
- BASIS: Original type: *Mattiastrum multicaule* Rech. f. & Riedl

---

*Mattiastrum* subsect. *Exalata* Riedl in Rechinger, Fl. Iranica 48: 122. 1967

- IPNI: absent (2014-11-05)
- ASSESSMENT: [*Cynoglossum* L.]
- STATUS: subsect. nov.
- REFERENCE: Link
- BASIS: Original type: *Mattiastrum polyanthum* Riedl

---

*Mattiastrum* sect. *Macromattiastrum* Brand in Repert. Spec. Nov. Regni Veg. 14: 150. 1915

- IPNI: absent (2014-11-05)
- ASSESSMENT: [*Cynoglossum* L.]
- STATUS: sect. nov.
- REFERENCE: Link
- SYNONYMY:   
  ≡ *Paracaryum* sect. *Macromattiastrum* (Brand) Popov 1953
- BASIS: Original elements: *Mattiastrum aucheri* (A. DC.) Brand, *M. velutinum* (Post) Brand, *M. glastifolium* (Willd.) Brand, *M. racemosum* (Schreb.) Brand, *M. corymbiforme* (DC. & A. DC) Brand, *M. incanum* (Ledeb.) Brand, *M. longipes* (Boiss.) Brand, *M. erysimifolium* (Boiss.) Brand , *M. ancyritanum* (Boiss.) Brand, *M. calycinum* (Boiss. & Balansa) Brand, *M. leptophyllum* (A. DC.) Brand, *Cynoglossum angustifolium* Willd., *C. emarginatum* Lam., *Paracaryum azureum* Boiss. & Heldr., *P. erigerifolium* Schott & Koschy ex Boiss., *Omphalodes pontica* K. Koch; type not designated,

---

*Mattiastrum* subg. *Mediomattiastrum* Riedl in Rechinger, Fl. Iranica 48: 124. 1967

- IPNI: absent (2014-11-05)
- ASSESSMENT: [*Cynoglossum* L.]
- STATUS: subg. nov.
- REFERENCE: Link
- BASIS: Original type: *Mattiastrum dielsii* Bornm.

---

*Mattiastrum* sect. *Modestomattiastrum* Brand in Repert. Spec. Nov. Regni Veg. 14: 152. 1915

- IPNI: absent (2014-11-05)
- ASSESSMENT: [*Cynoglossum* L.]
- STATUS: sect. nov.
- REFERENCE: Link
- SYNONYMY:   
  ≡ *Mattiastrum* subg. *Modestomattiastrum* (Brand) Riedl 1967
    
  ≡ *Paracaryum* sect. *Modestomattiastrum* (Brand) Popov 1953
    
  ≡ *Paracaryum* subg. *Modestomattiastrum* (Brand) R. R. Mill 1977
- BASIS: Type (Riedl in Rechinger, Fl. Iranica 48: 112. 1967): *Mattiastrum modestum* (Boiss. & Hausskn.) Brand

---

*Mattiastrum* subg. *Modestomattiastrum* (Brand) Riedl in Rechinger, Fl. Iranica 48: 112. 1967

- IPNI: absent (2014-11-05)
- ASSESSMENT: [*Cynoglossum* L.]
- STATUS: stat. nov. [section to subgenus]
- REFERENCE: Link
- BASIS: Basionym: *Mattiastrum* sect. *Modestomattiastrum* Brand

---

*Mattiastrum acrocladum* Rech. f. & Riedl in Biol. Skr. 13: 207. 1963

- IPNI: Mattiastrum acrocladum Rech.f. & Riedl -- Biol. Skr. xiii. No. 4 (Symb. Afghan. v.) 207(1963). (IK)
- ASSESSMENT: [*Cynoglossum himalayense* (Klotzsch) Greuter & Stier]
- STATUS: spec. nov.
- REFERENCE: Link
- BASIS: Original material: [Afghanistan], NE: Nuristan: Minjan, Miyan Deh, 2550m (*Edelberg 1451*, Typus, W, Isotypus, C) [from protologue]. Holotype: W (W1966-0019885); isotype: C (C10008749\*)

---

*Mattiastrum amani* Rech. f. in Ann. Naturhist. Mus. Wien 58: 50. 1951

- IPNI: Mattiastrum amani Rech.f. -- in Ann. Naturhist. Mus. Wien lviii. 50 (1951). (IK)
- ASSESSMENT: *Cynoglossum amani* (Rech. f.) Greuter & Burdet
- STATUS: spec. nov.
- REFERENCE: Link
- SYNONYMY:   
  ≡ *Paracaryum amani* (Rech. f.) R. R. Mill 1977
    
  ≡ *Cynoglossum amani* (Rech. f.) Greuter & Burdet 1981
- BASIS: Original material: Syria borealis: Mons Amanus, 5000', VI. 1906 (Haradjian no. 740, Typus in herb. Delessert) [from protologue]. Holotype: G (G00177875\*)

---

*Mattiastrum ancyritanum* (Boiss.) Brand in Repert. Spec. Nov. Regni Veg. 14: 152. 1915

- IPNI: Mattiastrum ancyritanum Brand -- Repert. Spec. Nov. Regni Veg. 14: 152. 1915 (IK)
- ASSESSMENT: *Cynoglossum ancyritanum* (Boiss.) Greuter & Burdet
- STATUS: comb. nov.
- REFERENCE: Link
- BASIS: Basionym: *Paracaryum ancyritanum* Boiss.

---

*Mattiastrum artvinense* (R. R. Mill) Valdés in Willdenowia 41: 312. 2011

- IPNI: Mattiastrum artvinense (R.R.Mill) Valdés -- Willdenowia 41(2): 312. 2011 [20 Dec 2011]
- ASSESSMENT: *Cynoglossum artvinense* (R. R. Mill) Greuter & Burdet
- STATUS: comb. nov.
- REFERENCE: Link
- BASIS: Basionym: *Paracaryum artvinense* R. R. Mill

---

*Mattiastrum asperum* (Stocks) Brand in Repert. Spec. Nov. Regni Veg. 14: 153. 1915

- IPNI: Mattiastrum asperum Brand -- Repert. Spec. Nov. Regni Veg. 14: 153. 1915 (IK)
- ASSESSMENT: *Cynoglossum asperum* (Stocks) Greuter & Stier
- STATUS: comb. nov.
- REFERENCE: Link
- BASIS: Basionym: *Paracaryum asperum* Stocks

---

*Mattiastrum aucheri* (A. DC.) Brand in Repert. Spec. Nov. Regni Veg. 14: 150. 1915

- IPNI: Mattiastrum aucheri Brand -- Repert. Spec. Nov. Regni Veg. 14: 150. 1915 (IK)
- ASSESSMENT: *Cynoglossum aucheri* (A. DC.) Greuter & Burdet
- STATUS: comb. nov.
- REFERENCE: Link
- BASIS: Basionym: *Mattia aucheri* A. DC.

---

*Mattiastrum badghysii* F. Sadat in Mitt. Bot. Staatssamml. München 28: 67. 1989

- IPNI: Mattiastrum badghysii F.Sadat -- Mitt. Bot. Staatssamml. München 28: 67 (1989). (IK)
- ASSESSMENT: *Cynoglossum badghysii* (F. Sadat) Greuter & Stier
- STATUS: spec. nov.
- REFERENCE: Link
- SYNONYMY:   
  ≡ *Cynoglossum badghysii* (F. Sadat) Greuter & Stier 2015
- BASIS: Original material: Holotypus: Afghanistan, Prov. Badghis: Djawand (Joand), 64/08 E - 35/04 N, 1000m, Juni 1950, Neubauer 431 (Hb. PODL.) [from protologue]. Holotype: MSB (MSB002970\*)

---

*Mattiastrum brandianum* of Bornmüller in Magyar Bot. Lapok 30: 71. 1931

- IPNI: absent (2014-11-05)
- STATUS: nom. inval. [nom. prov., ICN Art. 36.1 (b)]
- REFERENCE: Link
- COMMENTS: Described, in German, in a discussion but named only provisionally.

---

*Mattiastrum bungei* (Boiss.) Rech. f. & Riedl in Biol. Skr. 13: 209. 1963

- IPNI: Mattiastrum bungei (Boiss.) Rech.f. & Riedl -- Biol. Skr. xiii. No. 4 (Symb. Afghan. v.) 209 (963), cum descr. emend. (IK)
- ASSESSMENT: [*Microparacaryum intermedium* (Fresen.) Hilger & Podlech]
- STATUS: comb. nov.
- REFERENCE: Link
- BASIS: Basionym: *Echinospermum bungei* Boiss.

---

*Mattiastrum calycinum* (Boiss. & Balansa) Brand in Repert. Spec. Nov. Regni Veg. 14: 152. 1915

- IPNI: Mattiastrum calycinum Brand -- Repert. Spec. Nov. Regni Veg. 14: 152. 1915 (IK)
- ASSESSMENT: *Cynoglossum argaeum* Greuter & Burdet
- STATUS: comb. nov.
- REFERENCE: Link
- BASIS: Basionym: *Paracaryum calycinum* Boiss. & Balansa

---

*Mattiastrum cappadocicum* (Boiss. & Balansa) Brand in Repert. Spec. Nov. Regni Veg. 14: 155. 1915

- IPNI: Mattiastrum cappadocicum Brand -- Repert. Spec. Nov. Regni Veg. 14: 155. 1915 (IK)
- ASSESSMENT: *Cynoglossum caesareum* Greuter & Burdet
- STATUS: comb. nov.
- REFERENCE: Link
- BASIS: Basionym: *Paracaryum cappadocicum* Boiss. & Balansa

---

*Mattiastrum corymbiforme* (DC. & A. DC.) Brand in Repert. Spec. Nov. Regni Veg. 14: 151. 1915

- IPNI: Mattiastrum corymbiforme Brand -- Repert. Spec. Nov. Regni Veg. 14: 151. 1915 (IK)
- ASSESSMENT: *Cynoglossum corymbiforme* (DC. & A. DC.) Greuter & Burdet
- STATUS: comb. nov.
- REFERENCE: Link
- BASIS: Basionym: *Mattia corymbiformis* DC. & A. DC.

---

*Mattiastrum crista-galli* Rech. f. & Riedl in Biol. Skr. 13: 207. 1963

- IPNI: Mattiastrum crista-galli Rech.f. & Riedl -- Biol. Skr. xiii. No. 4 (Symb. Afghan. v.) 207 (963). (IK)
- ASSESSMENT: *Cynoglossum crista-galli* (Rech. f. & Riedl) Greuter & Stier
- STATUS: spec. nov.
- REFERENCE: Link
- SYNONYMY:   
  ≡ *Paracaryum crista-galli* (Rech. f. & Riedl) Kamelin & Raenko 1985
    
  ≡ *Cynoglossum crista-galli* (Rech. f. & Riedl) Greuter & Stier 2015
- BASIS: Original material: Fig. 158. Persia: E: Khorasan: Kuh-e Nishapur, Darreh Abshar supra Akhlomat, 1600-1800m (RECHINGER f. [& al] 4502, Typus, W) [+ 1 paratype] [from protologue]. Holotype: W (W 1966-0019587\*; see protologue, fig. 158); isotypes: E (E00284798\*),G (G00177873\*), M (M0174164\*), MO (MO-150758\*), S (S-G-3971\*), US (US00110908\*)

---

*Mattiastrum cristatum* (Schreb.) Brand in Repert. Spec. Nov. Regni Veg. 14: 154. 1915

- IPNI: Mattiastrum cristatum Brand -- Repert. Spec. Nov. Regni Veg. 14: 154. 1915 (IK)
- ASSESSMENT: *Cynoglossum cristatum* Schreb.
- STATUS: comb. nov.
- REFERENCE: Link
- BASIS: Basionym: *Cynoglossum cristatum* Schreb.

---

*Mattiastrum cristatum* subsp. *carduchorum* (R. R. Mill) Valdés in Willdenowia 41: 312. 2011

- IPNI: Mattiastrum cristatum Brand subsp. carduchorum (R.R.Mill) Valdés -- Willdenowia 41(2): 312. 2011 [20 Dec 2011]
- ASSESSMENT: *Cynoglossum cristatum* subsp. *carduchorum* (R. R. Mill) Greuter & Burdet
- STATUS: comb. nov.
- REFERENCE: Link
- BASIS: Basionym: *Paracaryum cristatum* subsp. *carduchorum* R. R. Mill

---

*Mattiastrum cristatum* var. *paropamisi* Brand in Engler, Pflanzenr. 78 (IV.252): 62. 1921

- IPNI: absent (2014-11-05)
- ASSESSMENT: *Cynoglossum* sp.
- STATUS: var. nov.
- REFERENCE: Link
- BASIS: Original material: Badchis: Kette des Paropamisus, sehr gemein auf den Hügeln und Abhängen bis zu 1700 m ü. M. (Aitchison n. 382, Herb. Boissier) [from protologue]. Holotype: G-BOIS; isotype: GH (GH00097547\*), K, LE

---

*Mattiastrum cynoglossoides* Rech. f. & Riedl in Rechinger, Fl. Iranica 48: 123. 1967

- IPNI: Mattiastrum cynoglossoides Rech.f. & Riedl -- Fl. Iranica [Rechinger] 48: 123. 1967 (IK)
- ASSESSMENT: *Cynoglossum cynoglossoides* (Rech. f. & Riedl) Greuter & Stier
- STATUS: spec. nov.
- REFERENCE: Link
- SYNONYMY:   
  ≡ *Paracaryum cynoglossoides* (Rech. f. & Riedl) Khat. 1994
    
  ≡ *Cynoglossum cynoglossoides* (Rech. f. & Riedl) Greuter & Stier 2015
- BASIS: Original material: Tab. 26. Typus: Rech 17688-b, W. Afghanistan: E: Ghazni: Okak, NE altoplanitei Dasht-e Nawar, 33°50' N, 67°55'E, 3000 m, 4. VII. 1962, Rech. 17688-b [+ 1 paratype] [from protologue]. Holotype: W (W 1966-0019528; see protologue, t. 26)

---

*Mattiastrum densum* Rech. f. & Riedl in Oesterr. Bot. Z. 110: 519. 1963

- IPNI: Mattiastrum densum Rech.f. & Riedl -- Oesterr. Bot. Z. 110: 519. 1963 (IK)
- ASSESSMENT: *Cynoglossum densum* (Rech. f. & Riedl) Greuter & Stier
- STATUS: spec. nov.
- REFERENCE: Link
- SYNONYMY:   
  ≡ *Paracaryum densum* (Rech. f. & Riedl) D. Heller 1986
    
  ≡ *Cynoglossum densum* (Rech. f. & Riedl) Greuter & Stier 2015
- BASIS: Original material: Abb. 4. Iraq: Distr. Diyala, ad confines Persiae. In collibus conglomeraticis ab oppido Mandala ca. 10 km orientem versus, ad ripam dextram fluvii, 2. VI. 1957. K. H. RECHINGER 12800 (Holotypus, W) [from protologue]. Holotype: W (W1966-0019351; see protologue, fig. 4\*)

---

*Mattiastrum dielsii* Bornm. in Bot. Jahrb. Syst. 66: 236. 1934

- IPNI: Mattiastrum dielsii Bornm. -- Bot. Jahrb. Syst. 66(2): 236. 1934 [25 Jan 1934] (IK)
- ASSESSMENT: *Cynoglossum dielsii* (Bornm.) Greuter & Stier
- STATUS: spec. nov.
- REFERENCE: Link
- SYNONYMY:   
  ≡ *Cynoglossum dielsii* (Bornm.) Greuter & Stier 2015
- BASIS: Original material: [Afghanistan], Dar-Ul-Aman, auf dem Berge Kuh-i-Gorächt, c. 1800-2000 m (V. 1927; [Manger] nr. 21) [from protologue]. Possible isotype: JE (JE00011977\*)

---

*Mattiastrum dieterlei* F. Sadat in Mitt. Bot. Staatssamml. München 28: 88. 1989

- IPNI: Mattiastrum dieterlei F.Sadat -- Mitt. Bot. Staatssamml. München 28: 88 (1989). (IK)
- ASSESSMENT: *Cynoglossum dieterlei* (F. Sadat) Greuter & Stier
- STATUS: spec. nov.
- REFERENCE: Link
- SYNONYMY:   
  ≡ *Cynoglossum dieterlei* (F. Sadat) Greuter & Stier 2015
- BASIS: Original material: Holotypus: [Afghanistan], Prov. Bamian: Band-i-Amir, Kohe Jak Ruya, 3500 m, 26.6.1970, *A. Dieterle 595* (Hb. PODL.!), Abb. 8 d-e; 10 b [+ 3 paratypes] [from protologue]. Holotype: MSB (MSB002968\*; see protologue, fig. 10 b\*)

---

*Mattiastrum emiri* (Popov) Czerep., Sosud. Rast. SSSR: 116. 1981

- IPNI: Mattiastrum emiri (Popov) Czerep. -- Sosud. Rast. SSSR 116 (1981):. (IK)
- ASSESSMENT: [*Cynoglossum himalayense* (Klotzsch) Greuter & Stier]
- STATUS: comb. nov.
- REFERENCE: Link
- BASIS: Basionym: *Paracaryum emiri* Popov

---

*Mattiastrum erysimifolium* (Boiss.) Brand in Repert. Spec. Nov. Regni Veg. 14: 152. 1915

- IPNI: Mattiastrum erysimifolium Brand -- Repert. Spec. Nov. Regni Veg. 14: 152. 1915 (IK)
- ASSESSMENT: *Cynoglossum erysimifolium* (Boiss.) Greuter & Burdet
- STATUS: comb. nov.
- REFERENCE: Link
- BASIS: Basionym: *Paracaryum erysimifolium* Boiss.

---

*Mattiastrum flaviflorum* Rech. f. & Riedl in Rechinger, Fl. Iranica 48: 122. 1967

- IPNI: Mattiastrum flaviflorum Rech.f. & Riedl -- Fl. Iranica [Rechinger] 48: 122. 1967 (IK)
- ASSESSMENT: *Cynoglossum flaviflorum* (Rech. f. & Riedl) Greuter & Stier
- STATUS: spec. nov.
- REFERENCE: Link
- SYNONYMY:   
  ≡ *Cynoglossum flaviflorum* (Rech. f. & Riedl) Greuter & Stier 2015
- BASIS: Original material: Tab. 19, fig. 8. Typus: RECH. 18446, W. Afghanistan: C: Bamian: Band-i Amir, ad lacum Band-i Zolfikar, 2900 m, 15. 7. 1962, RECH. 18446 [+ 6 paratypes] [HOLO: W; ISO: G, M fide Sadat 1989: 92] [from protologue]. Syntypes: W (W1966-0019530\*, W1966-0019531\*, W1966-0019532\*); isosyntypes: G (G00177795\*), M (M0174162\*)

---

*Mattiastrum formosum* Rech. f. & Riedl in Rechinger, Fl. Iranica 48: 119. 1967

- IPNI: Mattiastrum formosum Rech.f. & Riedl -- Fl. Iranica [Rechinger] 48: 119. 1967 (IK)
- ASSESSMENT: *Cynoglossum rechingeri* Greuter & Stier
- STATUS: spec. nov.
- REFERENCE: Link
- SYNONYMY:   
  ≡ *Cynoglossum rechingeri* Greuter & Stier 2015
- BASIS: Original material: Tab. 23; tab. 45, fig. 2. Typus: RECH. 31422, W. Afghanistan: E: Kabul: In declivibus australibus jugi Salang, 2700-3300 m, substr. granit., 25. VI. 65, RECH. 31422 [+ 1 paratype] [HOLO: W; ISO: G, K, M fide Sadat 1989: 90] [from protologue]. Holotype: W (W1966-0019669; see protologue, t. 23); isotypes: B (B100365405\*), C (C10008751\*), G (G00177796\*), GZU (GZU000272977\*), LD (LD1217701\*), M (M0174732\*), MO (MO-176907\*), MSB (MSB002969\*), S (S-G-3972\*), US (US00110909\*)

---

*Mattiastrum glastifolium*  (Willd.) Brand in Repert. Spec. Nov. Regni Veg. 14: 151. 1915

- IPNI: Mattiastrum glastifolium Brand -- Repert. Spec. Nov. Regni Veg. 14: 151. 1915 (IK)
- ASSESSMENT: *Cynoglossum glastifolium* Willd.
- STATUS: comb. nov.
- REFERENCE: Link
- BASIS: Basionym: *Cynoglossum glastifolium* Willd.

---

*Mattiastrum gorganicum*  Riedl in Rechinger, Fl. Iranica 48: 111. 1967

- IPNI: Mattiastrum gorganicum Riedl -- Fl. Iranica [Rechinger] 48: 111. 1967 (IK)
- ASSESSMENT: [*Cynoglossum turcomanicum* (Bornm. & Sint.) Greuter & Stier]
- STATUS: spec. nov.
- REFERENCE: Link
- SYNONYMY:   
  ≡ *Paracaryum gorganicum* (Riedl) D. Heller 1987
- BASIS: Original material: Tab. 21. Typus: SHAR. 5351-E, W. Persia: E: Khor.: Bojnurd: “Ansagarthe”, SHAR. 5351-E [from protologue]

---

*Mattiastrum gracile* (Czerniak.) Czerep., Sosud. Rast. SSSR: 116. 1981

- IPNI: Mattiastrum gracile (Czerniak.) Czerep. -- Sosud. Rast. SSSR 116 (1981):. (IK)
- ASSESSMENT: [*Cynoglossum turcomanicum* (Bornm. & Sint.) Greuter & Stier]
- STATUS: comb. nov.
- REFERENCE: Link
- BASIS: Basionym: *Paracaryum gracile* Czerniak.

---

*Mattiastrum heratense* Rech. f. & Riedl in Biol. Skr. 13: 210. 1963

- IPNI: Mattiastrum heratense Rech.f. & Riedl -- Biol. Skr. xiii. No. 4 (Symb. Afghan. v.) 210(1963). (IK)
- ASSESSMENT: *Cynoglossum heratense* (Rech. f. & Riedl) Greuter & Stier
- STATUS: spec. nov.
- REFERENCE: Link
- SYNONYMY:   
  ≡ *Paracaryum heratense*  (Rech. f. & Riedl) Kamelin 1975
    
  ≡ *Cynoglossum heratense* (Rech. f. & Riedl) Greuter & Stier 2015
- BASIS: Original material: Fig. 161. [Afghanistan], SW: Herat to Shin Dand, 1400m (Koeie 3937, Typus, W, Isotypus, C) [+ 1 paratype] [from protologue]. Holotype: W (W1966-0019869\*; see protologue, fig. 161); isotype: C (C10008750\*)

---

*Mattiastrum himalayense*  (Klotzsch) Brand in Repert. Spec. Nov. Regni Veg. 14: 156. 1915

- IPNI: Mattiastrum himalayense Brand -- Repert. Spec. Nov. Regni Veg. 14: 156. 1915 (IK)
- ASSESSMENT: *Cynoglossum himalayense* (Klotzsch) Greuter & Stier
- STATUS: comb. nov.
- REFERENCE: Link
- BASIS: Basionym: *Mattia himalayensis* Klotzsch

---

*Mattiastrum himalayense*  subsp. *fallax* Rech. f. & Riedl in Rechinger, Fl. Iranica 48: 117. 1967

- IPNI: absent (2014-11-05)
- ASSESSMENT: [*Cynoglossum heratense* (Rech. f. & Riedl) Greuter & Stier]
- STATUS: subsp. nov.
- REFERENCE: Link
- SYNONYMY:   
  ≡ *Mattiastrum himalayense*  var. *fallax* (Rech. f. & Riedl) Kazmi 1971
- BASIS: Original material: Tab. 22. Typus: HDG. & WDB. 4719, BE [recte: BG]. Afghanistan: C: Bareki Cheidan ad viam versus Band-e Amir ducentem, 2800 m, HDG. & WDB. 4719 [+ 5 paratypes] [from protologue]. Isotype: E (E00313423\*)

---

*Mattiastrum himalayense*  var. *fallax* (Rech. f. & Riedl) Kazmi in J. Arnold Arbor. 52: 128. 1971

- IPNI: Mattiastrum himalayense Brand var. fallax (Rech.f. & Riedl) Kazmi -- J. Arnold Arbor. 52(1): 128. 1971 (IK)
- ASSESSMENT: [*Cynoglossum heratense* (Rech. f. & Riedl) Greuter & Stier]
- STATUS: stat. nov. [subspecies to variety]
- REFERENCE: Link
- BASIS: Basionym: *Mattiastrum himalayense*  subsp. *fallax* Rech. f. & Riedl

---

*Mattiastrum honigbergeri* Rech. f. in Ann. Naturhist. Mus. Wien 55: 14. 1947

- IPNI: Mattiastrum honigbergeri Rech.f. -- in Ann. Naturhist. Mus. Wien lv. 14 (1947). (IK)
- ASSESSMENT: [*Cynoglossum asperum* (Stocks) Greuter & Stier]
- STATUS: spec. nov.
- REFERENCE: Link
- BASIS: Original material: Afghanistan: Kabul (Honigberger, Typus im Herb. Mus. Wien [W]) [from protologue]

---

*Mattiastrum howardii* Kazmi in J. Arnold Arbor. 52: 129. 1971

- IPNI: Mattiastrum howardii Kazmi -- J. Arnold Arbor. 52(1): 129. 1971 (IK)
- ASSESSMENT: [*Cynoglossum anchusoides* Lindl.]
- STATUS: spec. nov.
- REFERENCE: Link
- BASIS: Original material: Type: West Pakistan: Gilgit Agency, Dist. Astore, 7800 ft., 25. 7. 1892, *J. F. Duthie s.n.* (BM-holotype, E-isotype) [+ 2 paratypes] [from protologue]. Holotype: BM (BM001014420\*); isotype: E (E00288387\*)

---

*Mattiastrum incanum* (Ledeb.) Brand in Repert. Spec. Nov. Regni Veg. 14: 151. 1915

- IPNI: Mattiastrum incanum Brand -- Repert. Spec. Nov. Regni Veg. 14: 151. 1915 (IK)
- ASSESSMENT: *Cynoglossum incanum* (Ledeb.) Greuter & Burdet
- STATUS: comb. nov.
- REFERENCE: Link
- BASIS: Basionym: *Mattia incana* Ledeb.

---

*Mattiastrum karakoricum* Podlech & F. Sadat in Mitt. Bot. Staatssamml. München 27: 65. 1988

- IPNI: Mattiastrum karakoricum Podlech & F.Sadat -- Mitt. Bot. Staatssamml. München 27: 65 (1988). (IK)
- ASSESSMENT: *Cynoglossum karakoricum* (Podlech & F. Sadat) Greuter & Stier
- STATUS: spec. nov.
- REFERENCE: Link
- SYNONYMY:   
  ≡ *Cynoglossum karakoricum* (Podlech & F. Sadat) Greuter & Stier 2015
- BASIS: Original material: Holotypus: Pakistan, Hunza Karakorum: Naltar Tal, 2600 m, lichter Fichten-Kiefernwald, 10.8.1983, leg. *A. Bruhn* (Herb. PODLECH) [from protologue] Holotype: MSB (MSB003151\*)

---

*Mattiastrum karataviense*  (Pavlov ex Popov) Czerep., Sosud. Rast. SSSR: 116. 1981

- IPNI: Mattiastrum karataviense (Pavlov ex Popov) Czerep. -- Sosud. Rast. SSSR 116 (1981):. (IK)
- ASSESSMENT: *Cynoglossum karataviense* (Pavlov ex Popov) Greuter & Stier
- STATUS: comb. nov.
- REFERENCE: Link
- BASIS: Basionym: *Paracaryum karataviense* Pavlov ex Popov

---

*Mattiastrum kurdistanicum* Brand in Repert. Spec. Nov. Regni Veg. 14: 154. 1915

- IPNI: Mattiastrum kurdistanicum Brand -- Repert. Spec. Nov. Regni Veg. 14: 154. 1915 (IK)
- ASSESSMENT: *Cynoglossum kurdistanicum* (Brand) Greuter & Burdet
- STATUS: spec. nov.
- REFERENCE: Link
- SYNONYMY:   
  ≡ *Paracaryum kurdistanicum* (Brand) R. R. Mill 1977
    
  ≡ *Cynoglossum kurdistanicum* (Brand) Greuter & Burdet 1981
- BASIS: Original material: Kurdistan: Diarbekir: Karadjadagh, in Gebüschen. Blühend im Mai. (Sintensis no. 710, als *Paracaryum longipes?*) [from protologue]. Syntypes: BR (BR0000006967963\*), E (E00296270\*), G (G00226405\*), JE (JE00011691\*), LD (LD1217821\*, LD1217881\*), WU (WU 0069893)

---

*Mattiastrum lambertianum* (C. B. Clarke) Brand in Engler, Pflanzenr. 78 (IV.252): 61. 1921

- IPNI: Mattiastrum lambertianum Brand -- Pflanzenr. (Engler) Borrag.-Cynogloss. 61 (1921). (IK)
- ASSESSMENT: *Cynoglossum lambertianum* (C. B. Clarke) Greuter & Stier
- STATUS: comb. nov.
- REFERENCE: Link
- BASIS: Basionym: *Paracaryum lambertianum* C. B. Clarke

---

*Mattiastrum lamprocarpum* (Boiss.) Brand in Repert. Spec. Nov. Regni Veg. 14: 154. 1915

- IPNI: Mattiastrum lamprocarpum Brand -- Repert. Spec. Nov. Regni Veg. 14: 154. 1915 (IK)
- ASSESSMENT: *Cynoglossum lamprocarpum* (Boiss.) Greuter & Burdet
- STATUS: comb. nov.
- REFERENCE: Link
- BASIS: Basionym: *Paracaryum lamprocarpum* Boiss.

---

*Mattiastrum lamprocarpum* var. *warburgianum* Evenari in Bull. Soc. bot. Genève 31: 359. 1940 *(‘Warburgiana’)*

- IPNI: absent (2014-11-05)
- ASSESSMENT: [*Cynoglossum lamprocarpum* (Boiss.) Greuter & Burdet]
- STATUS: var. nov.
- REFERENCE: Link
- BASIS: Original material: H. [mont Hermon]: [*Aaronsohn*] 7096, Mezra'a, 27. VI. 1906 (fl. et fr.). Deux exemplaires de l’Herbier Boissier, “inter Rasheyia et Damas” et “ad Souk Wadi Barrada” [from protologue]

---

*Mattiastrum latiflorum* Rech. f. & Riedl in Biol. Skr. 13: 214. 1963

- IPNI: Mattiastrum latiflorum Rech.f. & Riedl -- Biol. Skr. xiii. No. 4 (Symb. Afghan. v.) 214 (963). (IK)
- ASSESSMENT: [*Cynoglossum flaviflorum* (Rech. f. & Riedl) Greuter & Stier]
- STATUS: spec. nov.
- REFERENCE: Link
- BASIS: Original material: Fig. 165; [Afghanistan] C: Koh-i Baba, 3300 m, 24.VII.1948 (KOEIE 2641, Typus, W) [+ 1 paratype] [from protologue]. Holotype: W (W1966-0019866; see protologue, fig. 165)

---

*Mattiastrum laxiflorum* (Trautv.) Czerep., Sosud. Rast. SSSR: 116. 1981

- IPNI: Mattiastrum laxiflorum (Trautv.) Czerep. -- Sosud. Rast. SSSR 116 (1981):. (IK)
- ASSESSMENT: *Cynoglossum laxiflorum* (Trautv.) Greuter & Burdet
- STATUS: comb. nov.
- REFERENCE: Link
- BASIS: Basionym: *Paracaryum laxiflorum* Trautv.

---

*Mattiastrum leptophyllum* (A. DC.) Brand in Repert. Spec. Nov. Regni Veg. 14: 152. 1915

- IPNI: Mattiastrum leptophyllum Brand -- Repert. Spec. Nov. Regni Veg. 14: 152. 1915 (IK)
- ASSESSMENT: *Cynoglossum leptophyllum* (A. DC.) Greuter & Burdet
- STATUS: comb. nov.
- REFERENCE: Link
- BASIS: Basionym: *Mattia leptophylla* A. DC.

---

*Mattiastrum lithospermifolium* (Lam.) Brand in Repert. Spec. Nov. Regni Veg. 14: 155. 1915

- IPNI: Mattiastrum lithospermifolium (Lam.) Brand -- Repert. Spec. Nov. Regni Veg. 14(10/15): 155. 1915 [31 Dec 1915] (IK)
- ASSESSMENT: *Cynoglossum lithospermifolium* Lam.
- STATUS: comb. nov.
- REFERENCE: Link
- BASIS: Basionym: *Cynoglossum lithospermifolium* Lam.

---

*Mattiastrum lithospermifolium* subsp. *cariense* (Boiss.) Valdés in Willdenowia 41: 312. 2011

- IPNI: Mattiastrum lithospermifolium (Lam.) Brand subsp. cariense (Boiss.) Valdés -- Willdenowia 41(2): 312. 2011 [20 Dec 2011]
- ASSESSMENT: *Cynoglossum lithospermifolium* subsp. *cariense* (Boiss.) Greuter & Burdet
- STATUS: comb. nov.
- REFERENCE: Link
- BASIS: Basionym: *Omphalodes cariensis* Boiss.

---

*Mattiastrum longipes*  (Boiss. & Balansa) Brand in Repert. Spec. Nov. Regni Veg. 14: 152. 1915

- IPNI: Mattiastrum longipes Brand -- Repert. Spec. Nov. Regni Veg. 14: 152. 1915 (IK)
- ASSESSMENT: *Cynoglossum longipes* (Boiss. & Balansa) Greuter & Burdet
- STATUS: comb. nov.
- REFERENCE: Link
- BASIS: Basionym: *Paracaryum longipes* Boiss. & Balansa

---

*Mattiastrum luristanicum* (Nábělek) Bornm. in Magyar Bot. Lapok 30: 71. 1931

- IPNI: absent (2014-11-05)
- ASSESSMENT: *Cynoglossum luristanicum* (Nábělek) Greuter & Stier
- STATUS: comb. nov.
- REFERENCE: Link
- BASIS: Basionym: *Paracaryum luristanicum* Nábělek

---

*Mattiastrum luristanicum* of Riedl in Rechinger, Fl. Iranica 48: 111. 1967

- IPNI: Mattiastrum luristanicum (Nábělek) Riedl -- Fl. Iranica [Rechinger] 48: 111. 1967 (IK)
- STATUS: [isonym]
- REFERENCE: Link

---

*Mattiastrum minutum* (Lipsky) Brand in Repert. Spec. Nov. Regni Veg. 14: 156. 1915

- IPNI: Mattiastrum minutum Brand -- Repert. Spec. Nov. Regni Veg. 14: 156. 1915 (IK)
- ASSESSMENT: *Lepechiniella minuta* (Lipsky) Popov
- STATUS: comb. nov.
- REFERENCE: Link
- BASIS: Basionym: *Paracaryum minutum* Lipsky

---

*Mattiastrum modestum* (Boiss. & Hausskn.) Brand in Repert. Spec. Nov. Regni Veg. 14: 154. 1915

- IPNI: Mattiastrum modestum Brand -- Repert. Spec. Nov. Regni Veg. 14: 154. 1915 (IK)
- ASSESSMENT: *Cynoglossum modestum* (Boiss. & Hausskn.) Greuter & Stier
- STATUS: comb. nov.
- REFERENCE: Link
- BASIS: Basionym: *Paracaryum modestum* Boiss. & Hausskn.

---

*Mattiastrum montbretii* Riedl in Oesterr. Bot. Z. 110: 520. 1963

- IPNI: Mattiastrum montbretii Riedl -- Oesterr. Bot. Z. 110: 520. 1963 (IK)
- ASSESSMENT: *Cynoglossum montbretii* (Riedl) Greuter & Burdet
- STATUS: spec. nov.
- REFERENCE: Link
- SYNONYMY:   
  ≡ *Paracaryum montbretii* (Riedl) R. R. Mill 1978
    
  ≡ *Cynoglossum montbretii* (Riedl) Greuter & Burdet 1981
- BASIS: Original material: Armenia turcica: Erzerum, 1834. MONTBRET 2544 (sub *Cynoglosso*, Holotypus, W) [from protologue]. Holotype: W (W0018882\*)

---

*Mattiastrum multicaule* Rech. f. & Riedl in Biol. Skr. 13: 216. 1963

- IPNI: Mattiastrum multicaule Rech.f. & Riedl -- Biol. Skr. xiii. No. 4 (Symb. Afghan. v.) 216(1963). (IK)
- ASSESSMENT: *Cynoglossum multicaule* (Rech. f. & Riedl) Greuter & Stier
- STATUS: spec. nov.
- REFERENCE: Link
- SYNONYMY:   
  ≡ *Cynoglossum multicaule* (Rech. f. & Riedl) Greuter & Stier 2015
- BASIS: Original material: Fig. 166; [Afghanistan], C: Pandjvai, Coteau, 14.IV.1958 (LINDBERG 380, Typus, W) [from protologue]. Holotype: W (W1966-0019911\*; see protologue, fig. 166)

---

*Mattiastrum nigrum*  Riedl in Rechinger, Fl. Iranica 48: 118. 1967

- IPNI: Mattiastrum nigrum Riedl -- Fl. Iranica [Rechinger] 48: 118. 1967 (IK)
- ASSESSMENT: *Cynoglossum nigrum* (Riedl) Greuter & Stier
- STATUS: spec. nov.
- REFERENCE: Link
- SYNONYMY:   
  ≡ *Paracaryum nigrum* (Riedl) D. Heller 1986
    
  ≡ *Cynoglossum nigrum* (Riedl) Greuter & Stier 2015
- BASIS: Original material: Tab. 45, fig. 1. Typus: HDG. & WDB. 5430 (Holotypus BE [recte: BG]) Afghanistan: Parvan: In valle Panjshir, in declivibus occidentalibus jugi Anjuman, 3900 m, 22.7.1962, HDG. & WDB. 5430 [+ 2 paratypes] from protologue]. Holotype: BG; isotype: E (E00253324\*)

---

*Mattiastrum paphlagonicum* Bornm. in Magyar Bot. Lapok 30: 69. 1931

- IPNI: Mattiastrum paphlagonicum Bornm. -- Magyar Bot. Lapok 1931, xxx. 69. (IK)
- ASSESSMENT: *Cynoglossum paphlagonicum* (Bornm.) Greuter & Burdet
- STATUS: spec. nov.
- REFERENCE: Link
- SYNONYMY:   
  ≡ *Paracaryum paphlagonicum* (Bornm.) R. R. Mill 1977
    
  ≡ *Cynoglossum paphlagonicum* (Bornm.) Greuter & Burdet 1981
- BASIS: Original material: [Turkey], Paphlagonia austr.: In regionis aridae planitie et in collibus glareosis sterilibus prope Koč-hissar alt. 1000 m sm, 23. VI. 1929 BORNM. No. 14414 [from protologue]. Syntypes: B (B100365404\*), BM (BM001014421\*), E (E00296263\*), G (G00236075\*), GH (GH00097550\*), JE (JE00011692\*, JE00011693\*), S (S-G-3973\*)

---

*Mattiastrum polyanthum* Rech. f. & Riedl in Rechinger, Fl. Iranica 48: 123. 1967

- IPNI: Mattiastrum polyanthum Rech.f. & Riedl -- Fl. Iranica [Rechinger] 48: 123. 1967 (IK)
- ASSESSMENT: *Cynoglossum polyanthum* (Rech. f. & Riedl) Greuter & Stier
- STATUS: spec. nov.
- REFERENCE: Link
- SYNONYMY:   
  ≡ *Paracaryum polyanthum* (Rech. f. & Riedl) Khat. 1994
    
  ≡ *Cynoglossum polyanthum* (Rech. f. & Riedl) Greuter & Stier 2015
- BASIS: Original material: Tab. 19, fig. 9; tab. 26). Typus: RECH. 17688, W. Afghanistan: E: Ghazni: Okak, NE altoplanitiei Dasht-i Nawar, 32°50'N 67°55'E, 3000 m, 4. VII. 1962, RECH. 17688-a [from protologue]. Holotype: W (W1966-0019529); isotypes: G, (G00236078\*) M (M0174168\*)

---

*Mattiastrum polycarpum* Rech. f. in Ann. Naturhist. Mus. Wien 58: 51. 1951

- IPNI: Mattiastrum polycarpum Rech.f. -- in Ann. Naturhist. Mus. Wien lviii. 51 (1951). (IK)
- ASSESSMENT: *Cynoglossum polycarpum* (Rech. f.) Greuter & Burdet
- STATUS: spec. nov.
- REFERENCE: Link
- SYNONYMY:   
  ≡ *Paracaryum polycarpum* (Rech. f.) R. R. Mill 1977
    
  ≡ *Cynoglossum polycarpum* (Rech. f.) Greuter & Burdet 1981
- BASIS: Original material: Syria borealis: Akher Dagh, 6000', VII. 1907 (Haradjian no. 1602, Typus in hb. Delessert) [from protologue]. Holotype: G (G00236146\*); isotype: E (E00287043\*)

---

*Mattiastrum pygmaeum* Rech. f. in Ann. Naturhist. Mus. Wien 58: 52. 1951

- IPNI: Mattiastrum pygmaeum Rech.f. -- in Ann. Naturhist. Mus. Wien lviii. 52 (1951). (IK)
- ASSESSMENT: *Cynoglossum pygmaeum* (Rech. f.) Greuter & Stier
- STATUS: spec. nov.
- REFERENCE: Link
- SYNONYMY:   
  ≡ *Paracaryum pygmaeum* (Rech. f.) D. Heller 1986
    
  ≡ *Cynoglossum pygmaeum* (Rech. f.) Greuter & Stier 2015
- BASIS: Original material: Persiae prov. Gorgan (Asterabad) : Montes Shavar, in saxosis calc. supra Ostameidan, 3500 m, 28. VII. 1948 (Rech. fil. no. 6022, Typus in hb. Mus. Wien) [from protologue]. Holotype: W (W1966-0019603\*)

---

*Mattiastrum racemosum* (Schreb.) Brand in Repert. Spec. Nov. Regni Veg. 14: 151. 1915

- IPNI: Mattiastrum racemosum Brand -- Repert. Spec. Nov. Regni Veg. 14: 151. 1915 (IK)
- ASSESSMENT: *Cynoglossum racemosum* Schreb.
- STATUS: comb. nov.
- REFERENCE: Link
- BASIS: Basionym: *Cynoglossum racemosum* Schreb.

---

*Mattiastrum reuteri* (Boiss. & Hausskn.) Brand in Repert. Spec. Nov. Regni Veg. 14: 155. 1915

- IPNI: Mattiastrum reuteri Brand -- Repert. Spec. Nov. Regni Veg. 14: 155. 1915 (IK)
- ASSESSMENT: *Cynoglossum reuteri* (Boiss. & Hausskn.) Greuter & Burdet
- STATUS: comb. nov.
- REFERENCE: Link
- BASIS: Basionym: *Paracaryum reuteri* Boiss. & Hausskn.

---

*Mattiastrum sarawschanicum* (Lipsky) Brand in Repert. Spec. Nov. Regni Veg. 14: 155. 1915

- IPNI: Mattiastrum sarawschanicum Brand -- Repert. Spec. Nov. Regni Veg. 14: 155. 1915 (IK)
- ASSESSMENT: *Lepechiniella sarawschanica* (Lipsky) Popov
- STATUS: comb. nov.
- REFERENCE: Link
- BASIS: Basionym: *Paracaryum sarawschanicum* Lipsky

---

*Mattiastrum scabridum* Rech. f. in Ann. Naturhist. Mus. Wien 58: 51. 1951

- IPNI: Mattiastrum scabridum Rech.f. -- in Ann. Naturhist. Mus. Wien lviii. 51 (1951). (IK)
- ASSESSMENT: [*Cynoglossum racemosum* Schreb.]
- STATUS: spec. nov.
- REFERENCE: Link
- SYNONYMY:   
  ≡ *Paracaryum racemosum* var. *scabridum* (Rech. f.) R. R. Mill 1977
- BASIS: Original material: Syria borealis: Mons Amanus, 5000', VII. 1906 (Haradjian no. 566, Typus in hb. Delessert) [from protologue]. Holotype: G

---

*Mattiastrum sessiliflorum*  Rech. f. & Riedl in Rechinger, Fl. Iranica 48: 125. 1967

- IPNI: Mattiastrum sessiliflorum Rech.f. & Riedl -- Fl. Iranica [Rechinger] 48: 125. 1967 (IK)
- ASSESSMENT: *Cynoglossum sessiliflorum* (Rech. f. & Riedl) Greuter & Stier
- STATUS: spec. nov.
- REFERENCE: Link
- SYNONYMY:   
  ≡ *Cynoglossum sessiliflorum* (Rech. f. & Riedl) Greuter & Stier 2015
- BASIS: Original material: Tab. 19, fig. 11; tab. 27. Typus: RECH. 17385, W. Afghanistan: E: Ghazni: In monte ad occidentem jugi Sardalu, inter Qarabagh et Sang-i Masha, substr. gyps., 2600 m, 30. VI. 1962, RECH. 17385 [from protologue]. Holotype: W (W1966-0019533); isotypes: B (B100365403\*), G (G00177624\*), K (), LD (LD1210761\*), M (M0174167\*), MO (MO-176909\*)

---

*Mattiastrum shepardii* (Post & Beauverd) Valdés in Willdenowia 41: 312. 2011

- IPNI: Mattiastrum shepardii (Post & Beauverd) Valdés -- Willdenowia 41(2): 312. 2011 [20 Dec 2011]
- ASSESSMENT: *Cynoglossum shepardii* (Post & Beauverd) Greuter & Burdet
- STATUS: comb. nov.
- REFERENCE: Link
- BASIS: Basionym: *Paracaryum shepardii* Post & Beauverd

---

*Mattiastrum stenolophum* (Boiss. & Balansa) Brand in Repert. Spec. Nov. Regni Veg. 14: 155. 1915

- IPNI: Mattiastrum stenolophum Brand -- Repert. Spec. Nov. Regni Veg. 14: 155. 1915 (IK)
- ASSESSMENT: *Cynoglossum stenolophum* (Boiss. & Balansa) Greuter & Burdet
- STATUS: comb. nov.
- REFERENCE: Link
- BASIS: Basionym: *Paracaryum stenolophum* Boiss. & Balansa

---

*Mattiastrum straussii*  (Hausskn. ex. Bornm.) Brand in Repert. Spec. Nov. Regni Veg. 14: 155. 1915

- IPNI: Mattiastrum straussii Brand -- Repert. Spec. Nov. Regni Veg. 14: 155. 1915 (IK)
- ASSESSMENT: [*Cynoglossum modestum* (Boiss. & Hausskn.) Greuter & Stier]
- STATUS: comb. nov.
- REFERENCE: Link
- BASIS: Basionym: *Paracaryum straussii* Hausskn. ex Bornm.

---

*Mattiastrum subscaposum* Rech. f. & Riedl in Rechinger, Fl. Iranica 48: 120. 1967

- IPNI: Mattiastrum subscaposum Rech.f. & Riedl -- Fl. Iranica [Rechinger] 48: 120. 1967 (IK)
- ASSESSMENT: *Cynoglossum subscaposum* (Rech. f. & Riedl) Greuter & Stier
- STATUS: spec. nov.
- REFERENCE: Link
- SYNONYMY:   
  ≡ *Cynoglossum subscaposum* (Rech. f. & Riedl) Greuter & Stier 2015
- BASIS: Original material: Tab. 19, fig. 6; tab. 24. Typus: RECH. 16926, W. Afghanistan: E: Kabul: In faucibus Tang-i Gharu inter Kabul et Sarobi, substr. gneiss, 1400-1500 m, 17. VI. 1962, RECH. 16926 [+ 2 paratypes] [from protologue]. Holotype: W (see protologue, t. 24)

---

*Mattiastrum thomsonii* (C. B. Clarke) Kazmi in J. Arnold Arbor. 52: 130. 1971

- IPNI: Mattiastrum thomsonii (C.B.Clarke) Kazmi -- J. Arnold Arbor. 52(1): 130. 1971 (IK)
- ASSESSMENT: *Cynoglossum thomsonii* (C. B. Clarke) I. M. Johnst.
- STATUS: comb. nov.
- REFERENCE: Link
- BASIS: Basionym: *Paracaryum thomsonii* C. B. Clarke

---

*Mattiastrum tibeticum* (C. B. Clarke) Brand in Repert. Spec. Nov. Regni Veg. 14: 156. 1915

- IPNI: Mattiastrum tibeticum Brand -- Repert. Spec. Nov. Regni Veg. 14: 156. 1915 (IK)
- ASSESSMENT: [*Microparacaryum intermedium* (Fresen.) Hilger & Podlech]
- STATUS: comb. nov.
- REFERENCE: Link
- BASIS: Basionym: *Paracaryum tibeticum* C. B. Clarke

---

*Mattiastrum tibeticum* var. *schlagintweitii* Brand in Engler, Pflanzenr. 78 (IV.252): 66. 1921

- IPNI: absent (2014-11-05)
- ASSESSMENT: [*Microparacaryum intermedium* (Fresen.) Hilger & Podlech]
- STATUS: var. nov.
- REFERENCE: Link
- BASIS: Original material: Tibet, Prov. Balti, auf dem Wege von Hùshe zum Sospor-Gletscher, Schlagintweit no. 6006 (Herb. Boissier) [from protologue]

---

*Mattiastrum trinervium* (Duthie) Brand in Engler, Pflanzenr. 78 (IV.252): 64. 1921

- IPNI: Mattiastrum trinervium Brand -- Pflanzenr. (Engler) Borrag.-Cynogloss. 64 (1921). (IK)
- ASSESSMENT: *Cynoglossum trinervium* (Duthie) Greuter & Stier
- STATUS: comb. nov.
- REFERENCE: Link
- BASIS: Basionym: *Paracaryum trinervium*  Duthie

---

*Mattiastrum turcomanicum*  (Bornm. & Sint.) Brand in Repert. Spec. Nov. Regni Veg. 14: 155. 1915

- IPNI: Mattiastrum turcomanicum Brand -- Repert. Spec. Nov. Regni Veg. 14: 155. 1915 (IK)
- ASSESSMENT: *Cynoglossum turcomanicum* (Bornm. & Sint.) Greuter & Stier
- STATUS: comb. nov.
- REFERENCE: Link
- BASIS: Basionym: *Paracaryum turcomanicum* Bornm. & Sint.

---

*Mattiastrum velutinum* (Post) Brand in Repert. Spec. Nov. Regni Veg. 14: 151. 1915

- IPNI: Mattiastrum velutinum Brand -- Repert. Spec. Nov. Regni Veg. 14: 151. 1915 (IK)
- ASSESSMENT: [*Cynoglossum schlumbergeri* (Boiss.) Greuter & Burdet]
- STATUS: comb. nov.
- REFERENCE: Link
- BASIS: Basionym: *Paracaryum velutinum* Post

---

*Memoremea scorpioides* (Haenke) Otero & al. in Phytotaxa 173 (4): 266. 2014

- IPNI: Memoremea scorpioides (Haenke) A.Otero, Jim.Mejías, Valcárcel & P.Vargas -- Phytotaxa 173(4): 266. 2014 [2 Jul 2014] [epublished]
- ASSESSMENT: accepted
- STATUS: comb. nov.
- REFERENCE: Link
- BASIS: Basionym: *Cynoglossum scorpioides* Haenke

---

*Mertensia lindelofioides*  Rech. f. & Riedl in Biol. Skr. 13: 233. 1963

- IPNI: Mertensia lindelofioides Rech.f. & Riedl -- Biol. Skr. xiii. No. 4 (Symb. Afghan. v.) 233 (963). (IK)
- ASSESSMENT: [*Cynoglossum olgae* (Regel & Smirn.) Greuter & Stier]
- STATUS: spec. nov.
- REFERENCE: Link
- SYNONYMY:   
  ≡ *Pseudomertensia lindelofioides*  (Rech. f. & Riedl) Riedl 1967
- BASIS: Original material: [Afghanistan] NE: Chiva, Paturage, 22.VII.1959 (LINDBERG 710, Typus, W). Salehlang, Pandjir, 7.VII.1960 (LINDBERG, W). Holotype: W (W1970-0012790\*)

---

*Microparacaryum* (Popov ex Riedl) Hilger & Podlech in Pl. Syst. Evol. 148: 302. 1985

- IPNI: Microparacaryum (Popov ex Riedl) Hilger & Podlech -- Pl. Syst. Evol. 148(3-4): 302. 1985 (IK)
- ASSESSMENT: accepted
- STATUS: nom. & stat. nov. [section to genus]
- REFERENCE: Link
- BASIS: Basionym: *Paracaryum* sect. *Microparacaryum* Popov ex Riedl

---

*Microparacaryum intermedium* (Fresen.) Hilger & Podlech in Pl. Syst. Evol. 148: 302. 1985

- IPNI: Microparacaryum intermedium (Fresen.) Hilger & Podlech -- Pl. Syst. Evol. 148(3-4): 302. 1985 (IK)
- ASSESSMENT: accepted
- STATUS: comb. nov.
- REFERENCE: Link
- BASIS: Basionym: *Cynoglossum intermedium* Fresen.

---

*Microparacaryum intermedium* f. *stellatum* (Riedl) Hilger & Podlech in Biodivers. Data J. [hoc loco]. 2015

- ASSESSMENT: accepted
- STATUS: stat. nov. [species via variety to forma]
- BASIS: Basionym: *Paracaryum stellatum* Riedl

---

*Microparacaryum salsum* (Boiss.) Hilger & Podlech in Pl. Syst. Evol. 148: 305. 1985

- IPNI: Microparacaryum salsum (Boiss.) Hilger & Podlech -- Pl. Syst. Evol. 148(3-4): 305. 1985 (IK)
- ASSESSMENT: accepted
- STATUS: comb. nov.
- REFERENCE: Link
- BASIS: Basionym: *Paracaryum salsum* Boiss.

---

*Moltkia coerulea*  (Willd.) Lehm. in Neue Schriften Naturf. Ges. Halle 3(2): 6. 1817.

- IPNI: Moltkia coerulea Lehm. -- in Neue Schr. Naturf. Ges. Halle iii. II. (1817) 6. (IK)
- ASSESSMENT: accepted
- STATUS: comb. nov.
- REFERENCE: Link
- BASIS: Basionym: *Onosma coerulea*  Willd.

---

*Moltkia libanotica*  Zucc. in Abh. Math.-Phys. Cl. Königl. Bayer. Akad. Wiss. 3: 246. 1843 *(‘Moltkea’)*

- IPNI: Moltkia libanotica Zucc. -- Abh. Math.-Phys. Cl. Königl. Bayer. Akad. Wiss. 3: 246, t. 3. f. 2. 1843 [1837-1843 publ. 1843] (IK)
- ASSESSMENT: [*Cynoglossum stamineum* Desf.]
- STATUS: spec. nov.
- REFERENCE: Link
- BASIS: Original material: locis glareosis ad lacum Baical in Sibiria (v. s.) [from protologue]. Original specimens: MEL?; S (S12-8154\*)

---

*Moltkia neubaueri* Rech. f. in Ann. Naturhist. Mus. Wien 58: 57. 1951

- IPNI: Moltkia neubaueri Rech.f. -- Ann. Naturhist. Mus. Wien Iviii. 57 (1951). (IK)
- ASSESSMENT: *Cynoglossum neubaueri* (Rech. f.) Greuter & Stier
- STATUS: spec. nov.
- REFERENCE: Link
- SYNONYMY:   
  ≡ *Rindera neubaueri* (Rech. f.) Rech. f. & Riedl 1963
    
  ≡ *Cynoglossum neubaueri* (Rech. f.) Greuter & Stier 2015
- BASIS: Original material: Afghanistan: Zentraler Gebirgsstock, Baraki Nawor, Hesaredschad, an Schneeflecken, 11. V. 1949 (Neubauer, no. 835) [from protologue]

---

*Myosotidium hortensia*  (Decne.) Baill., Hist. Pl. 10: 383. 1890

- IPNI: Myosotidium hortensia (Decne.) Baill. -- Hist. Pl. (Baillon) 10: 383, adnot. 1. 1890 [Jul-Aug 1890] (IK)
- ASSESSMENT: accepted
- STATUS: comb. nov.
- REFERENCE: Link
- BASIS: Basionym: *Myosotis hortensia*  Decne.
- COMMENTS: Considered the correct name of the heterotypic synonym *Cynoglossum nobile* Hook. f. 1858. Heenan & Schönberger (in New Zealand J. Bot. 47: 124) consider *hortensia* a three-ending adjective and “correct” Baillon’s *Myosotidium hortensia* to *M. ‘hortensium’*. They err. The epithet *Hortensia*, also a well known generic name, is a noun in apposition and does not take the gender of the generic name (ICN Art. 23.5); if proof be needed, in the basionym protologue it is written with a capital initial letter.

---

*Myosotidium nobile*  (Hook. f.) Hook. in Bot. Mag.: ad t. 5137. 1859

- IPNI: Myosotidium nobile (Hook.f.) Hook. -- Bot. Mag. 85: t. 5137. 1859 [1 Sep 1859] (IK)
- ASSESSMENT: [*Myosotidium hortensia*  (Decne.) Baill.]
- STATUS: comb. nov.
- REFERENCE: Link
- BASIS: Basionym: *Cynoglossum nobile* Hook. f.

---

*Myosotis borbonica*  Lam., Tabl. Encycl. 1: 396. 1791

- IPNI: Myosotis borbonica Lam. -- Tabl. Encycl. i. 396. (IK)
- ASSESSMENT: [*Cynoglossum borbonicum* Bory]
- STATUS: spec. nov.
- REFERENCE: Link
- SYNONYMY:   
  ≡ *Echinospermum borbonicum*  (Lam.) Lehm. 1818
    
  ≡ *Rochelia borbonica*  (Lam.) Roem. & Schult. 1819
- BASIS: Original material: information: Ex insula Borboniae. Commers. Herb. [from protologue]. Syntypes: P (P00417670\*, P00417671\*), P-LA
- COMMENTS: Belonging to *Cynoglossum* and a taxonomic synonym of *Cynoglossum borbonicum* Bory (1804), the publication of which prevents its transfer to that genus.

---

*Myosotis cynoglossoides*  Lam., Tabl. Encycl. 1: 396. 1792

- IPNI: Myosotis cynoglossoides Lam. -- Tabl. Encycl. i. 396. (IK)
- ASSESSMENT: *Lappula cynoglossoides*  (Lam.) Gürke
- STATUS: spec. nov.
- REFERENCE: Link
- SYNONYMY:   
  ≡ *Lappula cynoglossoides*  (Lam.) Gürke
- BASIS: Original material: E Cap. B. Spei. D. Sonnerat [from protologue]. Holotype presumably in P-LA

---

*Myosotis deflexa*  Wahlenb. in Kongl. Vetensk. Acad. Nya Handl. 31: 113. 1810

- IPNI: Myosotis deflexa Wahlenb. -- Kongl. Vetensk. Acad. Nya Handl. xxxi. (1810) 113. f. 4. (IK)
- ASSESSMENT: *Hackelia deflexa*  (Wahlenb.) Opiz
- STATUS: spec. nov.
- REFERENCE: Link
- SYNONYMY:   
  ≡ *Cynoglossum deflexum* (Wahlenb.) Roth 1827
    
  ≡ *Hackelia deflexa*  (Wahlenb.) Opiz 1839
- BASIS: Original material: Lapponia, år 1800 i Tramsens Fögderi af Norska Nordland vid Ballsfjorden uppom Merta gård. och 1807 på berget Njarnmats söder om Quickjocks prestgård i Luleå-Lappmark [from protologue], and plants cultivated in Uppsala and Stockholm botanic gardens; original illustration: t. 4. Lectotype (Moberg & Nilsson in Nordic J. Bot. 11: 292. 1991): “Myosotis reflexa, Norrska Nordland vid Balsfjord d. 18 Juli 1800” [Wahlenberg’s handwriting] (UPS)

---

*Myosotis hortensia*  Decne. in Delessert, Icon. Select. Pl. 5: 42. 1846

- IPNI: Myosotis hortensia Decne. -- Icon. Select. Pl. 5: 42 (t. 99). 1846 [Jun 1846] (IK)
- ASSESSMENT: *Myosotidium hortensia*  (Decne.) Baill.
- STATUS: spec. nov.
- REFERENCE: Link
- SYNONYMY:   
  ≡ *Myosotidium hortensia*  (Decne.) Baill.
- BASIS: Original material: Tab. CXIX. in insula Chatam legit Cl. navarchus Cecile. Lectotype (Heenan & Schönberger in New Zealand J. Bot. 47: 124. 2009, as “holotype”): Myosotis hortensia Dne., Borraginee, fl. bleues, Ile Chatam, dans les sables maritimes, Obre 1838, P (P00622862\*)

---

*Myosotis javanica*  of Thunberg [Lehmann in Neue Schriften Naturf. Ges. Halle 3: 21. 1817]

- IPNI: Myosotis javanica Sw. ex Lehm. -- in Neue Schr. Naturf. Ges. Halle iii. II. (1817) 21. (IK)
- STATUS: nom. inval. [nom. nud.]
- REFERENCE: Link
- COMMENTS: Subsequently published as *Echinospermum javanicum*  Lehm. (1818); see also *Cynoglossum javanicum* of Thunberg.

---

*Myosotis lappula*  L., Sp. Pl.: 131. 1753

- IPNI: Myosotis lappula L. -- Sp. Pl. 1: 131. 1753 [1 May 1753] (IK)
- ASSESSMENT: [*Lappula squarrosa*  (Retz.) Dumort.]
- STATUS: spec. nov.
- REFERENCE: Link
- SYNONYMY:   
  ≡ *Cynoglossum lappula* (L.) Scop. 1771
    
  ≡ *Cynoglossum clusii* Loisel. 1819, nom. illeg.
- BASIS: Original material: in Europae argillosis, nudis, ruderatis [from protologue]. Lectotype (Selvi in Taxon 53: 803. 2004): Herb. Linn. No. 180.9 (LINN\*)

---

*Myosotis squarrosa* Retz., Observ. Bot. 2: 9. 1781

- IPNI: Myosotis squarrosa Retz. -- Observ. Bot. (Retzius) ii. 9. (IK)
- ASSESSMENT: *Lappula squarrosa*  (Retz.) Dumort.
- STATUS: spec. nov.
- REFERENCE: Link
- SYNONYMY:   
  ≡ *Lappula squarrosa*  (Retz.) Dumort. 1827
- BASIS: Original material: None indicated in protologue; descibed from cultivated plants

---

*Myosotis virginiana*  L., Sp. Pl.: 131. 1753

- IPNI: Myosotis virginiana L. -- Sp. Pl. 1: 131. 1753 [1 May 1753] (IK)
- ASSESSMENT: *Hackelia virginiana*  (L.) I. M. Johnst.
- STATUS: spec. nov.
- REFERENCE: Link
- SYNONYMY:   
  ≡ *Hackelia virginiana*  (L.) I. M. Johnst. 1923
- BASIS: Original material: Myosotis seminibus hispidis, foliis lanceolato-ovatis. Gron. virg. 19. … in Virginia [from protologue]. Lectotype (Wells in Taxon 53: 803. 2004): Clayton 111, BM (BM000038161\*)
- COMMENTS: Wells (in schedis lectotypi) has unaccountably identified the Clayton specimen as *Cynoglossum virginicum* L. (1767), and that error has made its way into online databases such as the Linnean Plant Name Typification Project’s and JStore’s of Global Plants. Fortunately, the specimen itself represents the *Hackelia* species to which the name has been traditionally attached, not the superficially similar *Cynoglossum* species. See also *Cynoglossum morisonii* DC..

---

*Myosotis zeylanica*  Lehm. in Neue Schriften Naturf. Ges. Halle 3: 20. 1817

- IPNI: Myosotis zeylanica Sw. ex Lehm. -- in Neue Schr. Naturf. Ges. Halle iii. II. (1817) 20. (IK)
- ASSESSMENT: *Cynoglossum zeylanicum* (Lehm.) Brand
- STATUS: nom. nov.
- REFERENCE: Link
- BASIS: Replaced synonym: *Anchusa zeylanica* Vahl ex Hornem., non *Anchusa zeylanica* J. Jacq.
- COMMENTS: Subsequently published as *Echinospermum zeylanicum* (Lehm.) Lehm. (1818 ); see also *Cynoglossum ceilanicum* of Thunberg, nom nud.

---

*Nihon japonicum* (Thunb.) Otero & al. in Phytotaxa 173 (4): 266. 2014

- IPNI: Nihon japonicum (Thunb.) A.Otero, Jim.Mejías, Valcárcel & P.Vargas -- Phytotaxa 173(4): 266. 2014 [2 Jul 2014] [epublished]
- ASSESSMENT: accepted
- STATUS: comb. nov.
- REFERENCE: Link
- BASIS: Basionym: *Cynoglossum japonicum* Thunb.

---

*Omphalodes* sect. *Paracaryum* A. DC., Prodr. 10: 159. 1846

- IPNI: absent (2014-11-06)
- ASSESSMENT: [*Cynoglossum* L.]
- STATUS: sect. nov.
- REFERENCE: Link
- SYNONYMY:   
  ≡ *Paracaryum* (A. DC.) Boiss. 1849
- BASIS: Type (Kazmi in J. Arnold Arbor. 52: 120. 1971): *Omphalodes rugulosa*  DC.

---

*Omphalodes* sect. *Pseudanchusa* A. DC., Prodr. 10: 158. 1846

- IPNI: absent (2014-11-06)
- ASSESSMENT: [*Cynoglossum* L.]
- STATUS: sect. nov.
- REFERENCE: Link
- BASIS: Original type: Omphalodes longiflora A. DC.

---

*Omphalodes brassicifolia*  (Lag.) Sweet in Hort. Brit.: 293. 1826

- IPNI: Omphalodes brassicifolia Sweet -- Hort. Brit. [Sweet] 293. 1826 (IK)
- ASSESSMENT: accepted
- STATUS: comb. nov.
- REFERENCE: Link
- BASIS: Basionym: *Cynoglossum brassicifolium* Lag.

---

*Omphalodes cappadocica*  (Willd.) DC., Prodr. 10: 161. 1846

- IPNI: Omphalodes cappadocica DC. -- Prodr. [A. P. de Candolle] 10: 161. 1846 [8 Apr 1846] (IK)
- ASSESSMENT: accepted
- STATUS: comb. nov.
- REFERENCE: Link
- BASIS: Basionym: *Cynoglossum cappadocicum* Willd.

---

*Omphalodes cariensis* Boiss., Diagn. Pl. Orient., ser. 1, 4: 41. 1844

- IPNI: Omphalodes cariensis Boiss. -- Diagn. Pl. Orient. ser. 1, 4: 41. 1844 [Jun 1844] (IK)
- ASSESSMENT: *Cynoglossum lithospermifolium* subsp. *cariense* (Boiss.) Greuter & Burdet
- STATUS: spec. nov.
- REFERENCE: Link
- SYNONYMY:   
  ≡ *Paracaryum cariense* (Boiss.) Boiss. 1849
    
  ≡ *Paracaryum lithospermifolium* subsp. *cariense* (Boiss.) R. R. Mill 1977
    
  ≡ *Cynoglossum lithospermifolium* subsp. *cariense* (Boiss.) Greuter & Burdet 1981
    
  ≡ *Mattiastrum lithospermifolium* subsp. *cariense* (Boiss.) Valdés 2011
- BASIS: Original material: *Omphalodes myosotoides* Boiss. in pl. Cariens. Pinardi; in pinguibus regionis alpinae *Cadmi* orientalis ubi legi Jun. 1842, in *Cariâ* interiori Pinard 1843 [from protologue]. Syntypes: 1842, *Boissier*, G-BOIS (G00330309\*), G-DC (G00205801\*, G00205802\*); 1843, Pinard, G (G00236074\*), G-BOIS (G00330308\*), G-DC (G00205808\*)

---

*Omphalodes cristata*  (Schreb.) Schrank in Denkschr. Bayer. Akad. Wiss. 3: 221 1812

- IPNI: Omphalodes cristata Schrank -- Denkschr. Akad. Muench. iii. (1812) 221. (IK)
- ASSESSMENT: *Cynoglossum cristatum* Schreb.
- STATUS: comb. nov.
- REFERENCE: Link
- BASIS: Basionym: *Cynoglossum cristatum* Schreb.

---

*Omphalodes glochidiata* Bunge in Mém. Acad. Imp. Sci. St.-Pétersbourg Divers Savans, 7: 413. 1851

- IPNI: Omphalodes glochidiata Bunge -- Mém. Acad. Imp. Sci. St.-Pétersbourg Divers Savans vii. (1847) 413. (IK)
- ASSESSMENT: [*Microparacaryum intermedium* (Fresen.) Hilger & Podlech]
- STATUS: spec. nov.
- REFERENCE: Link
- SYNONYMY:   
  ≡ *Paracaryum glochidiatum* (Bunge) Riedl 1970, non Benth. & Hook. f. ex C. B. Clarke 1883
- BASIS: Original material: An den Granitfelsen bei Bakali. 27. April, in den Lehmgründen des südlichen Kisilkum 29. April 1842 (florens et fructibus infimis vix maturescentibus) [*Lehmann*] [from protologue]. Holotype or lectotype (Riedl in Anz. Österr. Akad. Wiss., Math.-Naturwiss. Kl. 106: 8. 1970): LE

---

*Omphalodes hirsuta*  DC., Prodr. 10: 159. 1846

- IPNI: Omphalodes hirsuta DC. -- Prodr. [A. P. de Candolle] 10: 159. 1846 [8 Apr 1846] (IK)
- ASSESSMENT: *Cynoglossum paracaryum* Greuter & Burdet
- STATUS: spec. nov.
- REFERENCE: Link
- SYNONYMY:   
  ≡ *Paracaryum hirsutum* (DC.) Boiss. 1849
    
  ≡ *Cynoglossum paracaryum* Greuter & Burdet 1981
- BASIS: Original material: in Armenia (Auch. 1836 n. 1936), in monte Tauro (Auch. 1837 n. 2232 [recte: 2283]) [from protologue]. Lectotype (Mill in Davis, Fl. Turkey 6: 298. 1978): *Aucher-Eloy 2283*, G-DC (G00200168\*); isolectotypes: G (G00096141\*), G-BOIS (G00150250\*); paralectotype: *Aucher-Eloy 1936*, G-DC (G00200167\*)

---

*Omphalodes linifolia*  (L.) Moench, Methodus: 419. 1794

- IPNI: Omphalodes linifolia Moench -- Methodus (Moench) 419. 1794 [4 May 1794] (IK)
- ASSESSMENT: accepted
- STATUS: comb. nov.
- REFERENCE: Link
- BASIS: Basionym: *Cynoglossum linifolium* L.

---

*Omphalodes linifolia*  var. *caerulescens* (DC.) DC., Prodr. 10: 161. 1846 *(‘coerulescens’)*

- IPNI: absent (2014-11-06)
- ASSESSMENT: [*Omphalodes linifolia*  (L.) Moench]
- STATUS: comb. nov.
- REFERENCE: Link
- BASIS: Basionym: *Cynoglossum linifolium* var. *caerulescens* DC.

---

*Omphalodes littoralis*  Lehm. in Mag. Neuesten Entdeck. Gesammten Naturk. Ges. Naturf. Freunde Berlin 8: 98. 1818

- IPNI: Omphalodes littoralis Lehm. -- Mag. Neuesten Entdeck. Gesammten Naturk. Ges. Naturf. Freunde Berlin 8: 97 (-99). 1818 (IK)
- ASSESSMENT: accepted
- STATUS: spec. nov.
- REFERENCE: Link
- SYNONYMY:   
  ≡ *Cynoglossum littorale* (Lehm.) Spreng. 1824
- BASIS: Original material: in littoribus Galliae occidentalis. In einigen Herbarien französischer Botaniker unter dem Namen *Cynoglossum linifolium* gesehen, und … erhalten [from protologue]

---

*Omphalodes longiflora*  A. DC. in Candolle, Prodr. 10: 158. 1846

- IPNI: Omphalodes longiflora DC. -- Prodr. [A. P. de Candolle] 10: 158. 1846 [8 Apr 1846] (IK)
- ASSESSMENT: [*Cynoglossum longifolium* (Leichtlin ex Beck & F. Abel) Greuter & Stier]
- STATUS: nom. nov. [ICN Art. 58.1]
- REFERENCE: Link
- BASIS: Replaced synonym: *Cynoglossum longiflorum* Royle ex Benth., non *Cynoglossum longiflorum* Lehm.

---

*Omphalodes lusitanica*  (L.) Schrank in Denkschr. Bayer. Akad. Wiss. 3: 221. 1812

- IPNI: Omphalodes lusitanica Schrank -- Denkschr. Akad. Muench. iii. (1812) 221. (IK)
- ASSESSMENT: Omphalodes sp. (nomen dubium)
- STATUS: comb. nov.
- REFERENCE: Link
- BASIS: Basionym: *Cynoglossum lusitanicum* L.
[truncated: 172,361 more chars]
